# Congenital Anomalies in a Neonate With Partial Monosomy of Chromosome 21 q Arm: A Case Report

**DOI:** 10.7759/cureus.98751

**Published:** 2025-12-08

**Authors:** Praisy Joy, Sudeshna Datta, Sanjukta Sahoo, Tapas Kumar Som, Prabhas R Tripathy, Manisha R Gaikwad, Pavithra Ayyanar, Sunil K Raghav

**Affiliations:** 1 Department of Anatomy, All India Institute of Medical Sciences, Bhubaneswar, Bhubaneswar, IND; 2 Immunogenomics and Systems Biology Laboratory, Institute of Life Sciences, Bhubaneswar, IND; 3 Department of Neonatology, All India Institute of Medical Sciences, Bhubaneswar, Bhubaneswar, IND; 4 Department of Pathology and Laboratory Medicine, All India Institute of Medical Sciences, Bhubaneswar, Bhubaneswar, IND

**Keywords:** 21q deletion, congenital anomalies (ca), next generation sequencing (ngs), partial 21 monosomy, ring chromosome 21, structural variants (sv), whole genome sequencing (wgs)

## Abstract

Congenital anomalies are a major cause of infant morbidity and mortality. Whole-genome sequencing provides a potential tool for solving diagnostic dilemmas in such instances. We report an interesting case of a neonate from India with holoprosencephaly and multiple associated anomalies, including dysmorphic features and ventricular septal defect. Karyotype analysis revealed mitotic instability. Both ring chromosome 21 and deletions in the q arm of chromosome 21 were observed in the metaphase spreads. To characterize the genomic abnormality more precisely, whole genome sequencing was performed, which identified a partial monosomy involving the 21q22.11-q22.3 region. This case demonstrates how whole-genome sequencing effectively identifies copy number variations and complex structural abnormalities, providing valuable insights into the genetic basis of congenital abnormalities.

## Introduction

A congenital anomaly (CA) is defined as “a structural, functional, behavioural, or metabolic abnormality present during the intrauterine period that may be detected prenatally or at birth” [1]. CAs profoundly impact the health and well-being of individuals, families, healthcare systems, and communities, as they cause early miscarriage and fetal and infant death. They can be classified as major or minor anomalies according to the International Classification of Disease guidelines by the World Health Organization [2]. Major anomalies affect the infant’s life expectancy and physical or social functions, whereas minor anomalies have little or no impact on health [2]. Although CAs occur globally, its burden is higher in developing countries [3]. In India, the incidence ranges from 0.84% to 2.9%, making it the fourth leading cause (8.6%) of neonatal mortality and the sixth leading cause of under-five mortality [4]. Common anomalies are neural tube defects, congenital heart diseases, cleft lip and palate, and limb abnormalities. With the emergence of 4D ultrasound and advances in sequencing techniques, the trend in detection has increased over the years. There is a genetic aetiology in 30-40% of such cases.

Methods to understand the aetiology of CA include karyotyping, fluorescence in situ hybridisation (FISH), comparative genomic hybridization (CGH) array, and next-generation sequencing (NGS) techniques. In recent times, NGS is one of the most powerful methods for the diagnosis of congenital abnormalities and rare Mendelian disorders. It is widely used to detect single-nucleotide variants (SNVs) and structural variants (SVs) [5]. SVs are large genomic alterations of at least 50 nucleotides or larger. They are generally classified as unbalanced gains or losses of DNA (copy-number variants, CNVs) or balanced rearrangements (inversions and translocations) [5]. Complex SVs disrupt large genomic regions and account for germline and somatic disorders [6].

Here, we present a case of a neonate with multiple congenital anomalies and evidence of unstable mitotic behaviour. Cytogenetic analysis was found to have a mosaic pattern, with 10 metaphases demonstrating a ring chromosome 21 and 15 metaphases with a deletion of the long arm of chromosome 21. For further exploration of the underlying genetic aetiology, whole genome sequencing (WGS) was performed. WGS detected a partial monosomy of approximately 15.2 Mb on chromosome 21, extending from 21q22.11-q22.3 (chr21:31,118,415-46,286,297, hg38).

## Case presentation

Materials and methods

Ethical Clearance

Patient was enrolled in an institutional review board-approved research study (IEC Number: T/IM-NF/Anatomy/23/44). The procedures followed were in accordance with the ethical standards of the institution’s committee on human research and were in keeping with international standards.

Whole Genome Sequencing and Preprocessing

WGS was performed using the Nextera DNA Flex (Illumina, San Diego, CA, USA) library prep kit according to the manufacturer’s guidelines. Following library preparation and quantification, we performed paired-end sequencing (150 bp) of the library using the Novaseq 6000 sequencing instrument (Illumina). The raw BCL files were demultiplexed, and the quality of the fastq files was checked using the FASTQC tool (https://www.bioinformatics.babraham.ac.uk/projects/fastqc/). Adapter sequences and low-quality bases were trimmed using fastp (v0.39) to minimize sequencing errors [7]. High-quality paired-end reads were then aligned to the hg38 human reference genome using the BWA-MEM aligner (v0.7.17) [8]. Picard MarkDuplicates tool (2.26.5) (http://broadinstitute.github.io/picard) was used to mark the PCR duplicate reads, which were excluded from downstream analysis. Base quality score recalibration and variant calling were performed using GATK (v4.1.2.0) according to GATK Best Practices recommendations [9-11].

Identification of CNVs and Gene Prioritization Analysis

DELLY’s (v1.0.3) [12] read-depth profile analysis was used for the detection of CNVs. We used two R packages (R Foundation for Statistical Computing, Vienna, Austria) to annotate the genes in the CNV region: org.Hs.eg.db [13] and AnnotationDbi [14]. We mapped the RefSeq EntrezID to chromosomal cytobands, gene symbols, Online Mendelian Inheritance in Man (OMIM) identifiers, and gene biotypes using various org.Hs.eg.db package functions. Then, using a custom R script, we filtered for chromosome 21 and merged all the above variables.

Identification and Annotation of SVs

For SV calling and genotyping, we used three callers: DELLY [12], MANTA (v1.6.0) [15] and smoove (https://github.com/brentp/smoove?tab=readme-ov-file) with default parameters to ensure concurrent and accurate SV calling. For all three callers, SVs with split-read support of ≤ 3 were filtered out. The identified SVs were merged using the SURVIVOR (v 1.0.7) [16]-merge function within a 1 kb breakpoint window, considering SV. Only SVs detected by all three methods and ≥50 bp in length were retained; variants >1 Mb were excluded due to detection limitations in short-read sequencing. We first annotated SVs with population-level allele frequency (AF) information using the SVAFotate tool with “-f 0.8, -a best mis -c 0” parameters [17]. SVAFotate aggregates and combines allele frequencies from multiple population datasets, such as CCDG [18], gnomAD [5], 1000G [19], and TOPMed [20]. To identify rare and unique SVs in our dataset, we excluded common SVs (Max_AF >= 0.01). For functional annotation, we used AnnotSV (v3.4.4) [21] with the GRCh38 build of the human genome. The resulting output file includes gene-based annotations, information on repeats, genic intolerance, and overlapping features from various databases such as the Database of Genomic Variants [22], 1000 Genomes [19], gnomAD [5], ExAC databases [23], disease-based annotation from OMIM [24], pathogenic features from dbVar [25], Exomiser score [26] and American College of Medical Genetics and Genomics (ACMG) categories [27]. We extracted the GnomAD pLI (probability of being loss-of-function intolerant) scores from annotSV results to understand the gene constraint of the identified SVs in the genomic regions. pLI is a score that indicates the probability of a gene being intolerant to a loss-of-function (LoF) variation. A gene with a pLI score >= 0.9 is considered extremely LoF intolerant. We used the VarElect, a free online phenotype-dependent variant prioritizer for screening genes in the CNV region. We queried all genes present in the CNV region with disease phenotypes of the patient and exported the result in Excel (Microsoft, Redmond, WA, USA) [28].

Functional Enrichment Analysis

Gene ontology (GO) analyses for the genes impacted with SVs were performed in R using the package ClusterProfiler [29]. The plots for the paper were created using GGPLOT2 package in R. The karyotype plot and visualization of reciprocal deletion and duplication were created using the karyoplotR [30] and Gviz [31] packages, respectively.

Clinical Details

**Figure 1:**
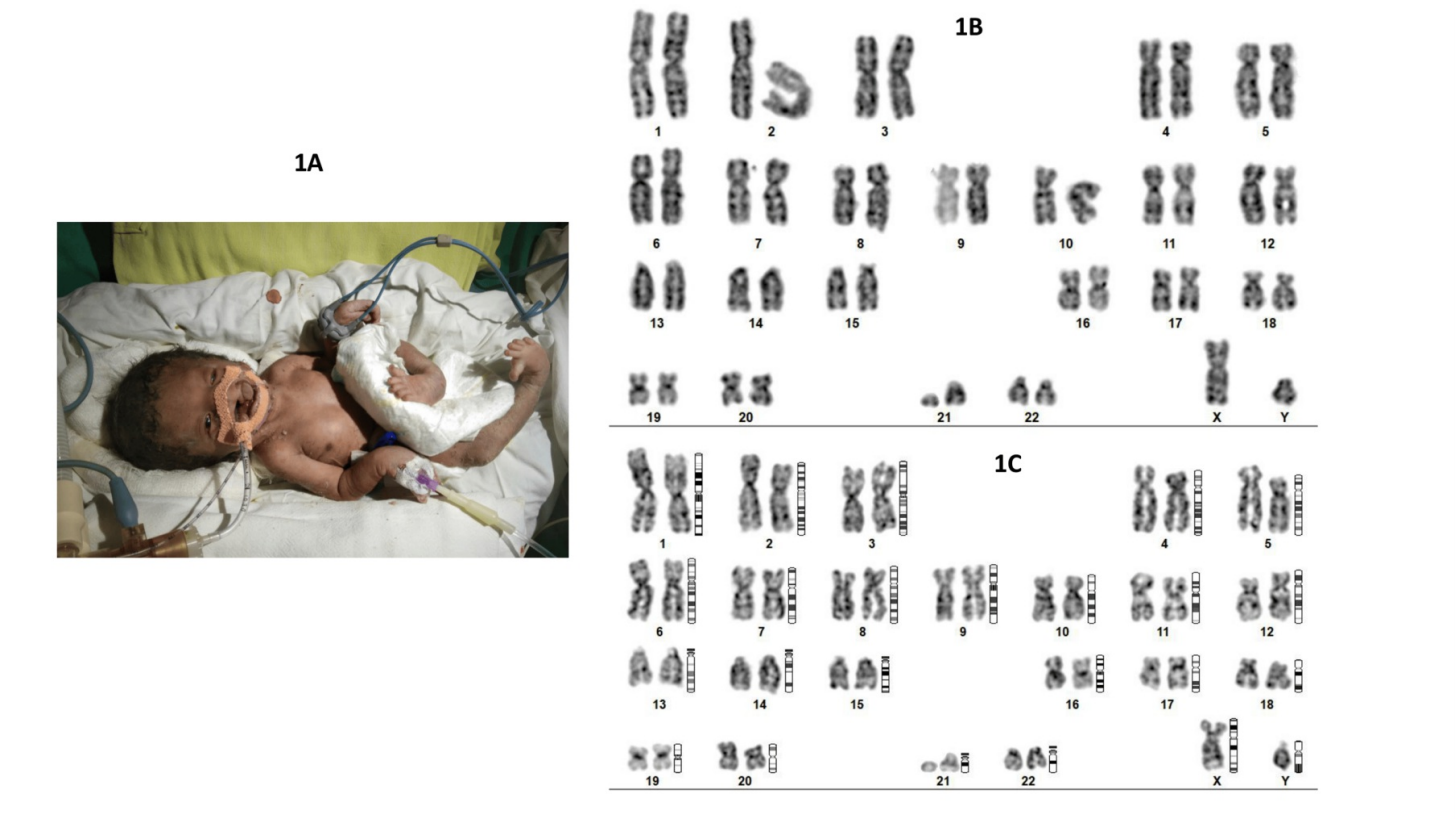
Cytogenetic analysis reveals r(21) and deletion of q arm A) The neonate in the NICU. Dysmorphism in the form of microophthalmia, hypertelorism, wide spaced nipples, pectus excavatum, club foot and big toe is seen in this photo. B) Karyotype showing 21 q deletion. C) Karyotype showing ring chromosome in chromosome 21.

A one-day-old male baby was admitted to the neonatal intensive care unit (NICU) with multiple anomalies. The baby was born to a nonconsanguineously married couple who had a previous spontaneous abortion during early pregnancy. This was their second pregnancy and the antenatal period was unremarkable. Ultrasound scans done during the last trimester of pregnancy showed hydrocephalus. Quickening was not felt. The baby was 1.340 kg at birth, born by normal vaginal delivery at 37 weeks. The baby did not cry at birth. APGAR Score at 1 and 5 minutes was 2 and 3. The baby had severe perinatal asphyxia. Initially, bag and mask ventilation was done, and since there were no respiratory efforts, the baby was intubated and started on a ventilator. Blood gas analysis was not done. The baby passed away on the same day seven hours after birth due to multiple congenital anomalies. On examination, the baby had microphthalmia, hypertelorism, corneal clouding, micrognathia, bilateral anotia, central cleft lip (harelip) and cleft palate, arthrogryposis (fixed flexion deformity), bilateral congenital talipes equinovarus (CTEV), wide-spaced nipple, pectus excavatum, scoliosis, genu recurvatum, and bilateral large extended big toe (Figure 1A). However, there were no polydactyly or scalp defects. The baby had a ventricular septal defect on the echocardiogram. A neurosonogram showed holoprosencephaly. Karyotype was done which had a resolution of 9 Mb at the Department of Anatomy, All India Institute of Medical Sciences (AIIMS), Bhubaneswar. Karyotyping showed mos 46, X, r(21) (::p13->q22::) / 46, XY, del(21) (q?) [59%,41%] (Figure 1B, 1C). There was unstable mitotic behaviour that was noted. We wanted to do whole-genome sequencing to clear the diagnostic dilemma. The cause of death was severe perinatal asphyxia (hypoxic-ischemic encephalopathy (HIE) grade 3). Then, the fetal autopsy done in the Department of Pathology, AIIMS, Bhubaneswar, showed holoprosencephaly. No other internal anomalies were noted.

Results

WGS Identifies Partial Monosomy in Chromosome 21

**Figure 2:**
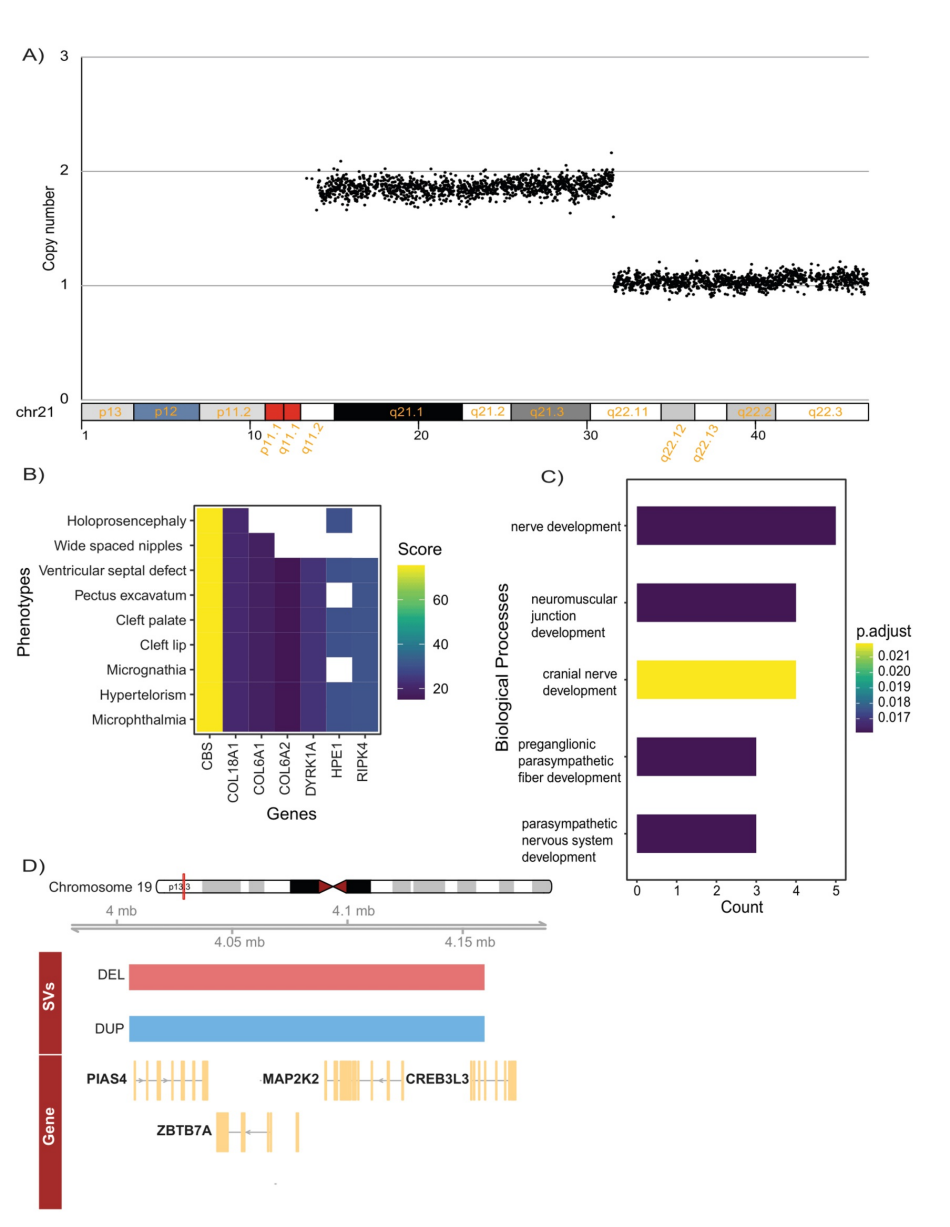
Whole genome analysis uncovered partial monosomy of chromosome 21 and reciprocal deletion and duplication on chromosome 19 A) Figure showing copy number variation (CNV) - Deletion of 21q in one arm of chromosome 21. B) Gene-phenotype plot for the genes observed in the CNV region. Legend denotes the VarElect score. C) Gene ontology (GO) BP enrichment analysis for the genes affected by structural variants (SVs). P-values were calculated using the non-parametric Wilcoxon rank-sum test, and adjusted p-values were calculated based on the Bonferroni correction procedure. D) Visualization of reciprocal deletion and duplication on chromosome 19. Red is for deletion, and blue is for duplication.

CNV analysis revealed a 15.2 Mb partial monosomy in the long arm of chromosome 21, extending from 21q22.11-q22.3 (chr21:31,118,415 - 46,286,297, hg38) (Figure 2A). The number of genes spanning this region is 505. Among them, the majority are non-coding RNAs (41.19%), followed by protein-coding genes (37.23%) and pseudogenes (15.84%) (Appendix 1 panel A, Appendix 2). Of the protein-coding genes, 19.6% are associated with OMIM disease phenotype (Appendix 2). Notably, *KCNJ6, PCNT2 *are involved in brain morphogenesis [32], while HPE1 is associated with holoprosencephaly [33]. Deletions in *SYNJ1, ITSN1, SLC5A3/SMIT1, and KCNE2*, located in the distal part of 21q22.11, are associated with neurobehavioral symptoms [34,35], whereas deletions in the critical region 21q22.11, such as *KCNE1* and *DSCR1*, lead to congenital heart defects [35].

Using VarElect [28] for phenotype-driven gene prioritization, we identified 89 genes associated with patient’s clinical features (Appendix 3). Applying a score threshold of ≥15, we identified seven top candidates, with the *CBS *gene showing the highest score of 75.41. The gene encodes cystathionine beta-synthase, and mutations in which cause homocystinuria, a multisystemic autosomal recessive disorder of the connective tissue, muscles, central nervous system (CNS), and cardiovascular system [36]. Pectus excavatum is common in homocystinuria patients, and it is also present in our case. Although CBS was phenotypically relevant, no pathogenic mutations were detected. Additionally,* COL6A1 *and *COL6A2* (Bethlem myopathy genes) and DYRK1A (linked to intellectual disability syndrome and ocular anomalies) [37-40] were identified as key candidates.

SV Analysis Identifies Reciprocal Deletion and Duplication

In addition to CNV analysis, we identified 2,380 SVs using split-read, read-pair, and assembly-based approaches (Methods). The majority were deletions (94.51%), followed by duplications (2.3%), inversions (1.93%), and translocations (1.01%) (Appendix 1 panel B). Chromosome 21 exhibited the least number of SVs among all chromosomes, limited to deletions and insertions (Appendix 1 panel C). To identify patient-specific variants, SVs were annotated with AF from multiple cohorts and classified as rare (Max_AF < 0.01; n = 70) or unique (Max_AF = 0; n = 93). Of them, 119 SVs were predicted to affect 106 genes across coding, untranslated region (UTR), and intronic regions (Appendix 4).

GO analysis of these genes indicated enrichment in the biological processes associated with nervous system development (Figure 2C). Gene constraint analysis (gnomAD pLI ≥ 0.9) identified 18 SVs. Among these SVs, we found a reciprocal deletion and duplication on chromosome 19 (chr19:4,005,230-4,159,441) comprising three genes: *MAP2K2, PIAS4, ZBTB7A* (Figure 2D, 2E). *MAP2K2*, a key kinase in the RAS/MAPK pathway, is implicated in cardio-facio-cutaneous (CFC) syndrome [41]. *ZBTB7A* is a transcriptional repressor and is linked to developmental delay and neurological defects [42], and PIAS4 encodes a RING finger protein which interacts with the androgen receptor, is associated with abnormal head size phenotypes (macrocephaly or microcephaly) [43]. However, the effects of the reciprocal deletion and duplication on the patient phenotype remain unknown. 

## Discussion

21q deletion syndrome (ORPHA:574) is a rare chromosomal anomaly (less than 1 in 1000000 births) characterized by the loss of variable portions of a segment of the long arm of chromosome 21 leading to an increased risk of birth defects, developmental delay and intellectual deficit. The phenotypes range from mild to severe depending on the location and size of the area deleted.

In this report, we present a complex case with multiple congenital anomalies and early neonatal death, characterized by mosaic chromosomal abnormalities involving chromosome 21. Conventional cytogenetic analysis revealed a mosaic karyotype consisting of cells with ring morphology and others with deletion of the long arm of chromosome 21; mos 46, X, r21 (::p13->q22::) / 46, XY, del21 (q?) [59%,41%]. Ring chromosomes (RCs) are rare structural chromosomal anomalies with an estimated incidence of 1 in 50,000 live births [44]. RCs are found in 10-12% of cases involving chromosomes 18, 20, and X, and in 5-9% of cases involving chromosomes 13, 14, 15, 21, 22, and Y [44]. Autosomal RCs are usually unstable and may lead to different mosaic genetic constitutions [45]. Similar to the phenotypes observed in our case, a previous study has reported microcephaly, mild asymmetry of the face, depressed nasal bridge, squint in both eyes, flat occiput, large ears, broad trunk, wide nipple, and pectus excavatum in a 29-month-old patient. Cytogenetic analysis revealed a mosaic karyotype mos 46,XX,r21(p11.1q22.1?2)/45,XX, del21/46,XX [46].

To further elucidate the structural abnormalities, WGS was performed. CNV analysis revealed a terminal deletion of 15.2 Mb on chromosome 21q22.11-q22.3 confirming the long arm deletion from cytogenetic analysis. However, we could not detect the ring chromosome or its breakpoint. This observation infers that the ring chromosome found might be an unstable rearrangement, with a progressive loss of distal genetic material occurring through mitotic divisions.

Chromosome 21q deletions present a wide phenotypic spectrum from mild to severe. Studies have reported three deletion regions. Region 1 extended from centromere to 31.2 Mb encompassing 50 genes, region 2 extended from 31.2 Mb to 36 Mb encompassing 80 genes causing a very severe phenotype and region 3 extended from 36 Mb and 37.5 Mb to telomere encompassing 130 genes causing a mild phenotype [35,47]. In our study, we report a partial monosomy of 15.2 Mb extending from 21q22.11-21q22.3. Here we observe deletions in region 2 and region 3, and similar to the findings of severe phenotypes associated with deletions in these regions [35,47] our patient too demonstrated lower birth weight, microphthalmia, hypertelorism, micrognathia, central cleft lip (harelip) and cleft palate, arthrogryposis, wide spaced nipple, pectus excavatum, bilateral large extended big toe, ventricular septal defect and holoprosencephaly. Previous studies have established that several genes within the deleted 21q22.11-q22.3 region are critical to neurodevelopmental and cardiac phenotypes [32-35]. In this study, we utilized VarElect, a phenotype-based gene prioritization tool, and found 89 genes to be directly associated with the patient's clinical features. Among the top-scoring genes (score ≥15) were *CBS, RIPK4, HPE1, and DYRK1A*. For instance, CBS is involved in homocysteine metabolism, and its disruption leads to homocystinuria, often associated with pectus excavatum, a feature also observed in our case. Despite its strong association with the clinical phenotype, we did not find any pathogenic mutations in the gene. The exact effect of these SVs on the phenotype of the patient is still unknown; their overall effect, together with the CNV at chromosome 21, could play a role in the clinical presentation.

Moon and Yum described an unusual case involving a newborn who initially showed signs consistent with HIE, such as low muscle tone, breathing problems, trouble feeding, and persistent seizures within the first day of life [48]. Brain MRI demonstrated widespread abnormal signals, suggesting possible perinatal asphyxia. Nevertheless, the lack of definite hypoxic events around birth and the atypical clinical progression led to further testing. Chromosome analysis identified a de novo ring chromosome 21 with a terminal deletion, confirming a genetic disorder rather than a brain injury caused by lack of oxygen. This case highlights the need to consider genetic causes in newborns with unexplained neurological symptoms, especially when imaging resembles HIE. The authors point out that ring chromosome 21 can produce a range of symptoms, including severe neurological problems at birth. This report contributes to the limited knowledge on newborn presentations of ring chromosome 21 and underscores the risk of misdiagnosis in cases that mimic acquired perinatal brain injury [48]. Our case also had a history of severe perinatal asphyxia suggestive of HIE.

## Conclusions

This report details a case of ring chromosome 21 exhibiting a severe phenotype, characterized by a 15.2 Mb deletion along with reciprocal deletion and duplication events on chromosome 19, as identified through whole genome sequencing. We have done the cytogenetic and molecular characterisation to delineate the cause and dilemma in the diagnosis of this case with multiple congenital anomalies. 21q deletions have a wide phenotypic spectrum of presentation. Genetic counselling and prenatal diagnosis will go a long way to help families with such disorders.

## Figures and Tables

**Figure 3:**
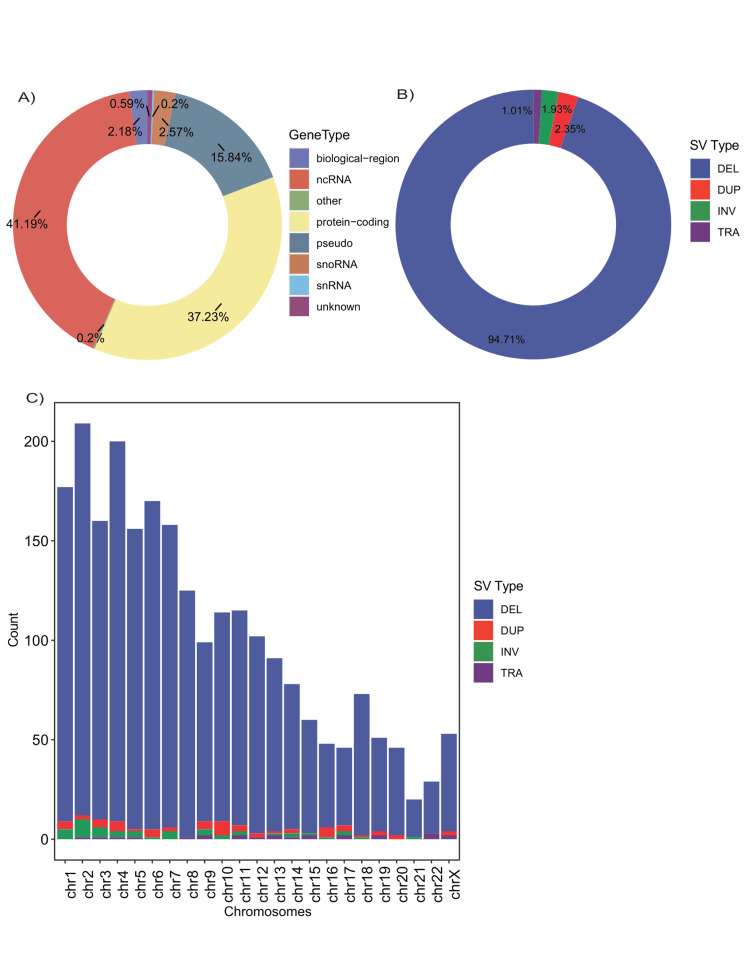
Distribution of structural variants A) Pie plot showing the percentage of genes in the copy-number variant (CNV) region belonging to various feature type. B) Pie plot showing the percentage of total structural variants (SVs) discovered. DEL, DUP, INV, TRA denote deletion, duplication, inversion and translocations. c) Stacked bar plot showing the total number of each type of SVs discovered across chromosomes.

## Appendices

Appendix 1

Appendix 2 

**Table 1:** Genes annotated in the copy-number variant (CNV) region

EntrezID	Cytoband	GeneSymbol	GeneType	Gene/Locus_MIM_number	Phenotype	Phenotype_MIM_number
539	21q22.11	ATP5PO	protein-coding	600828	Mitochondrial complex V (ATP synthase) deficiency, nuclear type 7	620359
757	21q22.11	TMEM50B	protein-coding	617894		
2618	21q22.11	GART	protein-coding	138440		
3454	21q22.11	IFNAR1	protein-coding	107450	Immunodeficiency 106, susceptibility to viral infections	619935
3455	21q22.11	IFNAR2	protein-coding	602376	{Hepatitis B virus, susceptibility to}	610424
3460	21q22.11	IFNGR2	protein-coding	147569	Immunodeficiency 28, mycobacteriosis	614889
3588	21q22.11	IL10RB	protein-coding	123889	{Hepatitis B virus, susceptibility to}	610424
6453	21q22.11	ITSN1	protein-coding	602442		
6526	21q22.11	SLC5A3	protein-coding	600444		
6647	21q22.11	SOD1	protein-coding	105400		
6651	21q22.11	SON	protein-coding	182465	ZTTK syndrome	617140
7074	21q22.11	TIAM1	protein-coding	600687	Neurodevelopmental disorder with language delay and seizures	619908
7339	21q22.11	UBE3AP2	pseudo			
8867	21q22.11	SYNJ1	protein-coding	604297	Developmental and epileptic encephalopathy 53	617389
9073	21q22.11	CLDN8	protein-coding	611231		
9875	21q22.11	URB1	protein-coding	608865		
9946	21q22.11	CRYZL1	protein-coding	603920		
9992	21q22.11	KCNE2	protein-coding	603796	Atrial fibrillation, familial, 4	611493
10215	21q22.11	OLIG2	protein-coding	606386		
29980	21q22.11	DONSON	protein-coding	251230		
30811	21q22.11	HUNK	protein-coding	606532		
54022	21q22.11	RPS5P3	pseudo			
54048	21q22.11	HMGN1P2	pseudo			
54065	21q22.11	SMIM11	protein-coding			
54067	21q22.11	C21orf62-AS1	ncRNA			
54069	21q22.11	MIS18A	protein-coding	618137		
54099	21q22.11	FBXW11P1	pseudo			
54943	21q22.11	DNAJC28	protein-coding			
56245	21q22.11	C21orf62	protein-coding			
56246	21q22.11	MRAP	protein-coding	607398		
56683	21q22.11	CFAP298	protein-coding	615494	Ciliary dyskinesia, primary, 26	615500
57466	21q22.11	SCAF4	protein-coding	616023	Fliedner-Zweier syndrome	620511
59271	21q22.11	EVA1C	protein-coding			
64968	21q22.11	MRPS6	protein-coding	611973		
84996	21q22.11	URB1-AS1	ncRNA			
94104	21q22.11	PAXBP1	protein-coding	617621		
114036	21q22.11	LINC00310	ncRNA			
116448	21q22.11	OLIG1	protein-coding	606385		
140258	21q22.11	KRTAP13-1	protein-coding	608718		
140290	21q22.11	TCP10L	protein-coding	608365		
150051	21q22.11	TIAM1-AS1	ncRNA			
254950	21q22.11	KRTAP15-1	protein-coding			
266919	21q22.11	LINC00307	ncRNA			
284827	21q22.11	KRTAP13-4	protein-coding			
337878	21q22.11	KRTAP7-1	protein-coding			
337879	21q22.11	KRTAP8-1	protein-coding			
337880	21q22.11	KRTAP11-1	protein-coding	600064		
337882	21q22.11	KRTAP19-1	protein-coding			
337959	21q22.11	KRTAP13-2	protein-coding			
337960	21q22.11	KRTAP13-3	protein-coding			
337963	21q22.11	KRTAP23-1	protein-coding			
337964	21q22.11	KRTAP13-5P	pseudo			
337965	21q22.11	KRTAP13-6P	pseudo			
337966	21q22.11	KRTAP6-1	protein-coding			
337967	21q22.11	KRTAP6-2	protein-coding			
337968	21q22.11	KRTAP6-3	protein-coding			
337969	21q22.11	KRTAP19-2	protein-coding			
337970	21q22.11	KRTAP19-3	protein-coding			
337971	21q22.11	KRTAP19-4	protein-coding			
337972	21q22.11	KRTAP19-5	protein-coding			
337973	21q22.11	KRTAP19-6	protein-coding			
337974	21q22.11	KRTAP19-7	protein-coding			
337975	21q22.11	KRTAP20-1	protein-coding			
337976	21q22.11	KRTAP20-2	protein-coding			
337977	21q22.11	KRTAP21-1	protein-coding			
337978	21q22.11	KRTAP21-2	protein-coding			
337979	21q22.11	KRTAP22-1	protein-coding			
337980	21q22.11	KRTAP8-2P	pseudo			
337981	21q22.11	KRTAP8-3P	pseudo			
337982	21q22.11	KRTAP19-9P	pseudo			
337983	21q22.11	KRTAP19-10P	pseudo			
337984	21q22.11	KRTAP19-11P	pseudo			
337985	21q22.11	KRTAP20-3	protein-coding			
337986	21q22.11	KRTAP21-4P	pseudo			
388818	21q22.11	KRTAP26-1	protein-coding			
391279	21q22.11	OR7E23P	pseudo			
643803	21q22.11	KRTAP24-1	protein-coding	618927		
643812	21q22.11	KRTAP27-1	protein-coding			
677846	21q22.11	SNORA80A	snoRNA			
728299	21q22.11	KRTAP19-8	protein-coding			
728409	21q22.11	LINC01548	ncRNA			
100128766	21q22.11	RPL8P2	pseudo			
100129168	21q22.11	MTRES1P2	pseudo			
100130240	21q22.11	RPS5P2	pseudo			
100131268	21q22.11	TPT1P1	pseudo			
100131902	21q22.11	KRTAP25-1	protein-coding			
100151640	21q22.11	EXOSC3P1	pseudo			
100151643	21q22.11	KRTAP20-4	protein-coding			
100151645	21q22.11	USF1P1	pseudo			
100288287	21q22.11	KRTAP22-2	protein-coding			
100288323	21q22.11	KRTAP21-3	protein-coding			
100288432	21q22.11	IL10RB-DT	ncRNA			
100422891	21q22.11	MIR4327	ncRNA			
100499261	21q22.11	BTF3P6	pseudo			
100506215	21q22.11	PAXBP1-AS1	ncRNA			
100506334	21q22.11	LINC00649	ncRNA			
100551499	21q22.11	LINC00159	ncRNA			
100873732	21q22.11	RNA5SP490	pseudo			
100874202	21q22.11	MIS18A-AS1	ncRNA			
101928078	21q22.11	LOC101928078	pseudo			
101928107	21q22.11	LOC101928107	ncRNA			
101928126	21q22.11	LOC101928126	ncRNA			
101930746	21q22.11	LINC00945	ncRNA			
102465248	21q22.11	MIR6501	ncRNA			
102724449	21q22.11	SOD1-DT	ncRNA			
102724502	21q22.11	LINC01690	ncRNA			
105372772	21q22.11	LOC105372772	ncRNA			
105372773	21q22.11	LOC105372773	ncRNA			
105372774	21q22.11	LOC105372774	ncRNA			
105372775	21q22.11	LOC105372775	ncRNA			
105372776	21q22.11	LOC105372776	ncRNA			
105372777	21q22.11	LOC105372777	ncRNA			
105372779	21q22.11	MRAP-AS1	ncRNA			
105372787	21q22.11	LOC105372787	ncRNA			
105372789	21q22.11	LOC105372789	ncRNA			
105372790	21q22.11	LOC105372790	ncRNA			
105372791	21q22.11	LOC105372791	ncRNA			
105377136	21q22.11	LOC105377136	ncRNA			
106144524	21q22.11	HUNK-AS1	ncRNA			
106479491	21q22.11	RN7SL740P	pseudo			
107985488	21q22.11	LOC107985488	ncRNA			
110091775	21q22.11	CFAP298-TCP10L	protein-coding			
124900469	21q22.11	LOC124900469	snoRNA			
124900470	21q22.11	LOC124900470	snoRNA			
124905008	21q22.11	LOC124905008	ncRNA			
124905009	21q22.11	LOC124905009	ncRNA			
124905010	21q22.11	LOC124905010	ncRNA			
124905011	21q22.11	LOC124905011	ncRNA			
124905012	21q22.11	LOC124905012	ncRNA			
124905013	21q22.11	LOC124905013	ncRNA			
127882475	21q22.11	IFNAR2-IL10RB	protein-coding			
861	21q22.12	RUNX1	protein-coding	151385	Leukemia, acute myeloid	601626
873	21q22.12	CBR1	protein-coding	114830		
874	21q22.12	CBR3	protein-coding	603608		
1827	21q22.12	RCAN1	protein-coding	602917		
3753	21q22.12	KCNE1	protein-coding	176261	Jervell and Lange-Nielsen syndrome 2	612347
8410	21q22.12	RPS9P1	pseudo			
8488	21q22.12	RPL3P1	pseudo			
8489	21q22.12	RPL23AP3	pseudo			
9980	21q22.12	DOP1B	protein-coding	604803		
23515	21q22.12	MORC3	protein-coding	610078		
54021	21q22.12	SRSF9P1	pseudo			
54024	21q22.12	RPS26P1	pseudo			
54025	21q22.12	RPS20P1	pseudo			
54026	21q22.12	RPL34P3	pseudo			
54031	21q22.12	RIMKLBP1	pseudo			
54036	21q22.12	PPP1R2P2	pseudo			
54064	21q22.12	LINC00160	ncRNA			
54093	21q22.12	SETD4	protein-coding			
54102	21q22.12	CLIC6	protein-coding	615321		
80215	21q22.12	RUNX1-IT1	ncRNA			
266693	21q22.12	EZH2P1	pseudo			
387315	21q22.12	MTND2P1	pseudo			
388820	21q22.12	SMIM34	protein-coding			
728556	21q22.12	MEMO1P1	pseudo			
768219	21q22.12	MIR802	ncRNA	616090		
100133286	21q22.12	CBR1-AS1	ncRNA			
100506385	21q22.12	LINC01426	ncRNA			
100506403	21q22.12	LOC100506403	ncRNA			
100506428	21q22.12	CBR3-AS1	ncRNA			
100996609	21q22.12	LINC01436	ncRNA			
101241877	21q22.12	ATP5MFP1	pseudo			
101928147	21q22.12	C21orf140	protein-coding			
101928269	21q22.12	LOC101928269	ncRNA			
105369295	21q22.12	SETD4-AS1	ncRNA			
105369306	21q22.12	LOC105369306	ncRNA			
105372793	21q22.12	LOC105372793	ncRNA			
106481493	21q22.12	RNU6-992P	pseudo			
107985515	21q22.12	LOC107985515	ncRNA			
112267915	21q22.12	LOC112267915	ncRNA			
124900475	21q22.12	LOC124900475	snoRNA			
124905014	21q22.12	RUNX1-AS1	ncRNA			
124905015	21q22.12	LOC124905015	ncRNA			
124905016	21q22.12	LOC124905016	ncRNA			
124905072	21q22.12	LOC124905072	snoRNA			
8208	21q22.12-q22.13	CHAF1B	protein-coding	601245		
1859	21q22.13	DYRK1A	protein-coding	600855	Intellectual developmental disorder, autosomal dominant 7	614104
3141	21q22.13	HLCS	protein-coding	253270		
3763	21q22.13	KCNJ6	protein-coding	600877	Keppen-Lubinsky syndrome	614098
6493	21q22.13	SIM2	protein-coding	600892		
7267	21q22.13	TTC3	protein-coding	602259		
10281	21q22.13	DSCR4	ncRNA	604829		
10311	21q22.13	VPS26C	protein-coding	605298		
23562	21q22.13	CLDN14	protein-coding	605608	Deafness, autosomal recessive 29	614035
51227	21q22.13	PIGP	protein-coding	605938	Developmental and epileptic encephalopathy 55	617599
53820	21q22.13	RIPPLY3	protein-coding	609892		
54035	21q22.13	PSMD4P1	pseudo			
84677	21q22.13	DSCR8	ncRNA	613396		
257203	21q22.13	DSCR9	ncRNA			
259234	21q22.13	DSCR10	ncRNA			
359737	21q22.13	MRPL20P1	pseudo			
503646	21q22.13	DPRXP5	pseudo			
100188792	21q22.13	MAFD3	unknown			
100288590	21q22.13	LOC100288590	pseudo			
100873733	21q22.13	RNA5SP491	pseudo			
100874006	21q22.13	TTC3-AS1	ncRNA			
100874060	21q22.13	SPATA20P1	pseudo			
100874294	21q22.13	HLCS-IT1	ncRNA			
100874327	21q22.13	DSCR4-IT1	ncRNA			
101928368	21q22.13	LOC101928368	ncRNA			
105369301	21q22.13	CLDN14-AS1	ncRNA			
105369305	21q22.13	LOC105369305	ncRNA			
105369308	21q22.13	LOC105369308	ncRNA			
105372797	21q22.13	LOC105372797	ncRNA			
105372798	21q22.13	LOC105372798	ncRNA			
105372799	21q22.13	KCNJ6-AS1	ncRNA			
105372801	21q22.13	LOC105372801	ncRNA			
106479873	21q22.13	RNU6-696P	pseudo			
106481108	21q22.13	RN7SL678P	pseudo			
107985492	21q22.13	LOC107985492	ncRNA			
108281168	21q22.13	LOC108281168	biological-region			
122455337	21q22.13	HLCS-AS1	ncRNA			
124905017	21q22.13	LOC124905017	ncRNA			
124905018	21q22.13	LOC124905018	ncRNA			
124905019	21q22.13	LOC124905019	ncRNA			
124905020	21q22.13	LOC124905020	ncRNA			
124905070	21q22.13	LOC124905070	snoRNA			
128193292	21q22.13	LNCTSI	ncRNA			
3772	21q22.13-q22.2	KCNJ15	protein-coding	602106		
100188797	21q22.13-qter	BMND6	unknown			
1826	21q22.2	DSCAM	protein-coding	602523		
2078	21q22.2	ERG	protein-coding	165080	Lymphatic malformation 14	620602
2114	21q22.2	ETS2	protein-coding	164740		
3150	21q22.2	HMGN1	protein-coding	163920		
5121	21q22.2	PCP4	protein-coding	601629		
6450	21q22.2	SH3BGR	protein-coding	602230		
7485	21q22.2	GET1	protein-coding	602915		
8624	21q22.2	PSMG1	protein-coding	605296		
10317	21q22.2	B3GALT5	protein-coding	604066		
54014	21q22.2	BRWD1	protein-coding	617824	Ciliary dyskinesia, primary, 51	620438
114041	21q22.2	B3GALT5-AS1	ncRNA			
150082	21q22.2	LCA5L	protein-coding			
150084	21q22.2	IGSF5	protein-coding	610638		
191585	21q22.2	PLAC4	ncRNA	613770		
282569	21q22.2	BACE2-IT1	ncRNA			
284835	21q22.2	LINC00323	ncRNA			
391282	21q22.2	RPL23AP12	pseudo			
400866	21q22.2	LINC00114	ncRNA	611723		
400867	21q22.2	ETS2-AS1	ncRNA			
692146	21q22.2	RPS26P4	pseudo			
729056	21q22.2	JCADP1	pseudo			
100420924	21q22.2	RNF6P1	pseudo			
100421629	21q22.2	METTL21AP1	pseudo			
100423023	21q22.2	MIR3197	ncRNA			
100431168	21q22.2	MYL6P2	pseudo			
100506492	21q22.2	DSCAM-AS1	ncRNA			
100616148	21q22.2	MIR4760	ncRNA			
100861429	21q22.2	YRDCP3	pseudo			
100862727	21q22.2	TIMM9P2	pseudo			
100873797	21q22.2	RPSAP64	pseudo			
100874093	21q22.2	BRWD1-AS1	ncRNA			
100874326	21q22.2	DSCAM-IT1	ncRNA			
100874428	21q22.2	SNRPGP13	pseudo			
101060045	21q22.2	PCBP2P1	pseudo			
101928398	21q22.2	LOC101928398	ncRNA			
101928435	21q22.2	LINC01700	ncRNA			
102466972	21q22.2	MIR6508	ncRNA			
102724678	21q22.2	LINC01423	ncRNA			
102724740	21q22.2	LINC02940	ncRNA			
103091865	21q22.2	BRWD1-AS2	ncRNA			
105369294	21q22.2	LOC105369294	ncRNA			
105372802	21q22.2	LOC105372802	ncRNA			
105372804	21q22.2	LOC105372804	ncRNA			
106480743	21q22.2	BRWD1-IT1	ncRNA			
106865373	21q22.2	GET1-SH3BGR	protein-coding			
107548109	21q22.2	LOC107548109	biological-region			
107548111	21q22.2	LOC107548111	biological-region			
107985480	21q22.2	LOC107985480	ncRNA			
107985484	21q22.2	LINC02943	ncRNA			
107985499	21q22.2	LOC107985499	ncRNA			
107985500	21q22.2	LOC107985500	ncRNA			
107985513	21q22.2	LOC107985513	ncRNA			
108281150	21q22.2	LOC108281150	biological-region			
111099028	21q22.2	LOC111099028	biological-region			
124900473	21q22.2	LOC124900473	snoRNA			
124905066	21q22.2	LOC124905066	pseudo			
124905074	21q22.2	LOC124905074	snoRNA			
25825	21q22.2-q22.3	BACE2	protein-coding	605668		
104	21q22.3	ADARB1	protein-coding	601218	Neurodevelopmental disorder with hypotonia, microcephaly, and seizures	618862
326	21q22.3	AIRE	protein-coding	240300		
754	21q22.3	PTTG1IP	protein-coding	603784		
755	21q22.3	CFAP410	protein-coding	602271		
875	21q22.3	CBS	protein-coding	236200		
1291	21q22.3	COL6A1	protein-coding	120220	Bethlem myopathy 1A	158810
1292	21q22.3	COL6A2	protein-coding	120240	?Myosclerosis, congenital	255600
1409	21q22.3	CRYAA	protein-coding	123580	Cataract 9, multiple types	604219
1476	21q22.3	CSTB	protein-coding	254800		
1637	21q22.3	DCR	other	190685	Down syndrome	190685
3244	21q22.3	HPE1	unknown			
3275	21q22.3	PRMT2	protein-coding	601961		
3689	21q22.3	ITGB2	protein-coding	116920		
4047	21q22.3	LSS	protein-coding	600909	Alopecia-intellectual disability syndrome 4	618840
4599	21q22.3	MX1	protein-coding	147150		
4600	21q22.3	MX2	protein-coding	147890		
4731	21q22.3	NDUFV3	protein-coding	602184		
5116	21q22.3	PCNT	protein-coding	210720		
5152	21q22.3	PDE9A	protein-coding	602973		
5211	21q22.3	PFKL	protein-coding	171860	Hemolytic anemia due to phosphofructokinase deficiency	
5316	21q22.3	PKNOX1	protein-coding	602100		
5822	21q22.3	PWP2	protein-coding	601475		
6285	21q22.3	S100B	protein-coding	176990		
6573	21q22.3	SLC19A1	protein-coding	600424	?Megaloblastic anemia, folate-responsive	601775
6612	21q22.3	SUMO3	protein-coding	602231		
7031	21q22.3	TFF1	protein-coding	113710		
7032	21q22.3	TFF2	protein-coding	182590		
7033	21q22.3	TFF3	protein-coding	600633		
7109	21q22.3	TRAPPC10	protein-coding	602103	Neurodevelopmental disorder with microcephaly, short stature, and speech delay	620027
7113	21q22.3	TMPRSS2	protein-coding	602060		
7226	21q22.3	TRPM2	protein-coding	603749		
7307	21q22.3	U2AF1	protein-coding	191317		
7327	21q22.3	UBE2G2	protein-coding	603124		
8209	21q22.3	GATD3	protein-coding	601659		
8566	21q22.3	PDXK	protein-coding	179020	Neuropathy, hereditary motor and sensory, type VIC, with optic atrophy	618511
8568	21q22.3	RRP1	protein-coding	610653		
8888	21q22.3	MCM3AP	protein-coding	603294	Peripheral neuropathy, autosomal recessive, with or without impaired intellectual development	618124
9619	21q22.3	ABCG1	protein-coding	603076		
10785	21q22.3	WDR4	protein-coding	605924	Galloway-Mowat syndrome 6	618347
10841	21q22.3	FTCD	protein-coding	229100		
11077	21q22.3	HSF2BP	protein-coding	604554	Premature ovarian failure 19	619245
23076	21q22.3	RRP1B	protein-coding	610654		
23181	21q22.3	DIP2A	protein-coding	607711		
23275	21q22.3	POFUT2	protein-coding	610249		
23308	21q22.3	ICOSLG	protein-coding	605717	?Immunodeficiency 119	620825
25966	21q22.3	C2CD2	protein-coding	617581		
29947	21q22.3	DNMT3L	protein-coding	606588		
49854	21q22.3	ZBTB21	protein-coding	616485		
53347	21q22.3	UBASH3A	protein-coding	605736		
54020	21q22.3	SLC37A1	protein-coding	608094		
54027	21q22.3	RPL31P1	pseudo			
54029	21q22.3	RPL23AP4	pseudo			
54039	21q22.3	PCBP3	protein-coding	608502		
54043	21q22.3	MYL6P1	pseudo			
54045	21q22.3	IMMTP1	pseudo			
54049	21q22.3	H2AZP1	pseudo			
54058	21q22.3	C21orf58	protein-coding			
54059	21q22.3	YBEY	protein-coding	617461		
54082	21q22.3	TSPEAR-AS1	ncRNA			
54084	21q22.3	TSPEAR	protein-coding	612920	?Deafness, autosomal recessive 98	614861
54089	21q22.3	LINC00112	ncRNA			
54090	21q22.3	LINC00111	ncRNA			
54097	21q22.3	FAM3B	protein-coding	608617		
54101	21q22.3	RIPK4	protein-coding	214350		
54145	21q22.3	H2BC12L	protein-coding			
56894	21q22.3	AGPAT3	protein-coding	614794		
63977	21q22.3	PRDM15	protein-coding	617692		
64699	21q22.3	TMPRSS3	protein-coding	601072		
80781	21q22.3	COL18A1	protein-coding	120328	Glaucoma, primary closed-angle	618880
81543	21q22.3	LRRC3	protein-coding	617620		
84221	21q22.3	SPATC1L	protein-coding	612412		
84536	21q22.3	LINC01547	ncRNA			
85395	21q22.3	SLX9	protein-coding			
89765	21q22.3	RSPH1	protein-coding	609314	Ciliary dyskinesia, primary, 24	615481
89766	21q22.3	UMODL1	protein-coding	613859		
90625	21q22.3	ERVH48-1	protein-coding			
114038	21q22.3	LINC00313	ncRNA			
114042	21q22.3	LINC00334	ncRNA			
114043	21q22.3	TSPEAR-AS2	ncRNA			
114044	21q22.3	MCM3AP-AS1	ncRNA			
150094	21q22.3	SIK1	protein-coding	605705	Developmental and epileptic encephalopathy 30	616341
150135	21q22.3	LINC00479	ncRNA			
150142	21q22.3	ZNF295-AS1	ncRNA			
150147	21q22.3	UMODL1-AS1	ncRNA			
246704	21q22.3	LINC00315	ncRNA			
284836	21q22.3	LINC00319	ncRNA			
284837	21q22.3	AATBC	ncRNA			
343819	21q22.3	MRPL51P2	pseudo			
353323	21q22.3	KRTAP12-2	protein-coding			
353332	21q22.3	KRTAP12-1	protein-coding			
353333	21q22.3	KRTAP10-10	protein-coding			
378825	21q22.3	PICSAR	ncRNA	617191		
378832	21q22.3	COL18A1-AS1	ncRNA			
386672	21q22.3	KRTAP10-4	protein-coding			
386674	21q22.3	KRTAP10-6	protein-coding			
386675	21q22.3	KRTAP10-7	protein-coding			
386676	21q22.3	KRTAP10-9	protein-coding			
386677	21q22.3	KRTAP10-1	protein-coding			
386678	21q22.3	KRTAP10-11	protein-coding			
386679	21q22.3	KRTAP10-2	protein-coding			
386680	21q22.3	KRTAP10-5	protein-coding			
386681	21q22.3	KRTAP10-8	protein-coding			
386682	21q22.3	KRTAP10-3	protein-coding			
386683	21q22.3	KRTAP12-3	protein-coding			
386684	21q22.3	KRTAP12-4	protein-coding			
386685	21q22.3	KRTAP10-12	protein-coding			
387492	21q22.3	DSTNP1	pseudo			
387494	21q22.3	RPL18AP2	pseudo			
388830	21q22.3	LINC00316	ncRNA			
642852	21q22.3	LINC00205	ncRNA			
727699	21q22.3	LINC00163	ncRNA	610259		
727701	21q22.3	LINC00165	ncRNA			
728039	21q22.3	SSR4P1	pseudo			
100126693	21q22.3	LINC00322	ncRNA			
100129027	21q22.3	PCBP3-AS1	ncRNA			
100505746	21q22.3	ITGB2-AS1	ncRNA			
100847013	21q22.3	MIR5692B	ncRNA			
100861507	21q22.3	FTCD-AS1	ncRNA			
100861510	21q22.3	LRRC3-DT	ncRNA			
100862692	21q22.3	DIP2A-IT1	ncRNA			
100873205	21q22.3	MTCO1P3	pseudo			
100873206	21q22.3	MTND5P1	pseudo			
100873332	21q22.3	MTND6P21	pseudo			
100873734	21q22.3	RNA5SP492	pseudo			
100873880	21q22.3	TMEM97P1	pseudo			
100874224	21q22.3	KRTAP10-13P	pseudo			
100874236	21q22.3	COL18A1-AS2	ncRNA			
100874510	21q22.3	PSMA6P3	pseudo			
101928212	21q22.3	LOC101928212	ncRNA			
101928233	21q22.3	LINC01671	ncRNA			
101928255	21q22.3	LOC101928255	ncRNA			
101928284	21q22.3	PDE9A-AS1	ncRNA			
101928311	21q22.3	LINC01668	ncRNA			
101928369	21q22.3	LOC101928369	ncRNA			
101928399	21q22.3	LINC01679	ncRNA			
101928607	21q22.3	TRPM2-AS	ncRNA			
101928689	21q22.3	LINC01424	ncRNA			
101928745	21q22.3	BNAT1	ncRNA			
101928796	21q22.3	LOC101928796	ncRNA			
101930094	21q22.3	RSPH1-DT	ncRNA			
102464825	21q22.3	MIR6070	ncRNA			
102465488	21q22.3	MIR6814	ncRNA			
102465489	21q22.3	MIR6815	ncRNA			
102723380	21q22.3	LOC102723380	ncRNA			
102724800	21q22.3	LOC102724800	ncRNA			
102725065	21q22.3	LOC102725065	ncRNA			
105372808	21q22.3	PCSEAT	ncRNA			
105372809	21q22.3	LOC105372809	ncRNA			
105372812	21q22.3	LOC105372812	ncRNA			
105372813	21q22.3	LOC105372813	ncRNA			
105372814	21q22.3	LOC105372814	ncRNA			
105372815	21q22.3	LOC105372815	ncRNA			
105372817	21q22.3	LOC105372817	ncRNA			
105372821	21q22.3	LOC105372821	ncRNA			
105372824	21q22.3	LOC105372824	ncRNA			
105372825	21q22.3	LOC105372825	ncRNA			
105372826	21q22.3	LOC105372826	ncRNA			
105372829	21q22.3	LOC105372829	ncRNA			
105372832	21q22.3	LOC105372832	ncRNA			
105372833	21q22.3	DNMT3L-AS1	ncRNA			
105372836	21q22.3	LOC105372836	ncRNA			
105372839	21q22.3	LOC105372839	ncRNA			
105372840	21q22.3	LINC01694	ncRNA			
105372841	21q22.3	LOC105372841	ncRNA			
105372842	21q22.3	LOC105372842	ncRNA			
105377137	21q22.3	LINC03039	ncRNA			
105377138	21q22.3	LINC01678	ncRNA			
105377139	21q22.3	LOC105377139	ncRNA			
106480072	21q22.3	RNU6-1150P	pseudo			
106481303	21q22.3	RNU6-396P	pseudo			
106481452	21q22.3	RNU6-859P	pseudo			
106481542	21q22.3	RNU6-1149P	pseudo			
106481742	21q22.3	FRGCA	ncRNA			
106635524	21q22.3	SNORA91	snoRNA			
107075260	21q22.3	MTCYBP21	pseudo			
107985478	21q22.3	LOC107985478	ncRNA			
107985485	21q22.3	LOC107985485	ncRNA			
107985502	21q22.3	LOC107985502	ncRNA			
107985504	21q22.3	LOC107985504	ncRNA			
107987299	21q22.3	LOC107987299	ncRNA			
107987300	21q22.3	LOC107987300	ncRNA			
107987303	21q22.3	LOC107987303	ncRNA			
108251799	21q22.3	LOC108251799	biological-region			
108254685	21q22.3	LOC108254685	biological-region			
108281139	21q22.3	LOC108281139	biological-region			
108281151	21q22.3	LOC108281151	biological-region			
109029533	21q22.3	LOC109029533	biological-region			
109623452	21q22.3	SNORD159	snoRNA			
111099027	21q22.3	LOC111099027	biological-region			
111216286	21q22.3	LINC02575	ncRNA			
112268487	21q22.3	KRTAP12-5P	pseudo			
122152369	21q22.3	COL6A2-DT	ncRNA			
124900467	21q22.3	LOC124900467	protein-coding			
124900472	21q22.3	LOC124900472	snoRNA			
124905021	21q22.3	LOC124905021	ncRNA			
124905022	21q22.3	LOC124905022	ncRNA			
124905023	21q22.3	LOC124905023	ncRNA			
124905024	21q22.3	LOC124905024	ncRNA			
124905025	21q22.3	LOC124905025	ncRNA			
124905026	21q22.3	LOC124905026	ncRNA			
124905027	21q22.3	LOC124905027	protein-coding			
124905028	21q22.3	LOC124905028	ncRNA			
124905029	21q22.3	LOC124905029	ncRNA			
124905030	21q22.3	LOC124905030	ncRNA			
124905031	21q22.3	LOC124905031	ncRNA			
124905032	21q22.3	LOC124905032	ncRNA			
124905033	21q22.3	LOC124905033	ncRNA			
124905034	21q22.3	LOC124905034	ncRNA			
124905035	21q22.3	LOC124905035	ncRNA			
124905036	21q22.3	HEPFAL	ncRNA			
124905037	21q22.3	LOC124905037	ncRNA			
124905038	21q22.3	LOC124905038	ncRNA			
124905039	21q22.3	LOC124905039	ncRNA			
124905040	21q22.3	LOC124905040	ncRNA			
124905041	21q22.3	LOC124905041	ncRNA			
124905042	21q22.3	LOC124905042	ncRNA			
124905043	21q22.3	LOC124905043	ncRNA			
124905044	21q22.3	LOC124905044	ncRNA			
124905045	21q22.3	LOC124905045	ncRNA			
124905046	21q22.3	LOC124905046	ncRNA			
124905058	21q22.3	LOC124905058	ncRNA			
124905060	21q22.3	LOC124905060	pseudo			
124905062	21q22.3	LOC124905062	snRNA			
124905068	21q22.3	LOC124905068	snoRNA			
124905069	21q22.3	LOC124905069	snoRNA			
128092249	21q22.3	LOC128092249	protein-coding			
130890644	21q22.3	MX1-AS1	ncRNA			

Appendix 3 

**Table 2:** Gene-phenotype correlation list from VarElect

Symbol	Description	Category	Matched Phenotypes	Matched Phenotypes Scount	Global Rank (Total Genes 12465)	-LOG10(P)	Score	Average Disease Causing Likelihood		
CBS	Cystathionine Beta-Synthase	Protein Coding	Microphthalmia, Hypertelorism, Micrognathia, Cleft lip, Cleft palate, Pectus excavatum, Ventricular septal defect, Wide\-spaced nipples, Holoprosencephaly	9	54	2.810805	75.41	0.547264		
RIPK4	Receptor Interacting Serine/Threonine Kinase 4	Protein Coding	Microphthalmia, Hypertelorism, Micrognathia, Cleft lip, Cleft palate, Ventricular septal defect, Wide\-spaced nipples	7	294	2.074851	30.71	0.540287		
HPE1	Holoprosencephaly 1, Alobar	Genetic Locus	Microphthalmia, Hypertelorism, Cleft lip, Cleft palate, Ventricular septal defect, Holoprosencephaly	6	269	2.113446	29.97	0		
DYRK1A	Dual Specificity Tyrosine Phosphorylation Regulated Kinase 1A	Protein Coding	Microphthalmia, Hypertelorism, Micrognathia, Cleft lip, Cleft palate, Pectus excavatum, Ventricular septal defect	7	486	1.856562	23.69	0.810935		
COL18A1	Collagen Type XVIII Alpha 1 Chain	Protein Coding	Microphthalmia, Hypertelorism, Micrognathia, Cleft lip, Cleft palate, Pectus excavatum, Ventricular septal defect, Wide\-spaced nipples, Holoprosencephaly	9	702	1.696861	21.51	0.062167		
COL6A1	Collagen Type VI Alpha 1 Chain	Protein Coding	Microphthalmia, Hypertelorism, Micrognathia, Cleft lip, Cleft palate, Pectus excavatum, Ventricular septal defect, Wide\-spaced nipples	8	817	1.630977	18.43	0.052352		
COL6A2	Collagen Type VI Alpha 2 Chain	Protein Coding	Microphthalmia, Hypertelorism, Micrognathia, Cleft lip, Cleft palate, Pectus excavatum, Ventricular septal defect	7	957	1.562287	15.37	0.068567		
SOD1	Superoxide Dismutase 1	Protein Coding	Microphthalmia, Hypertelorism, Micrognathia, Cleft lip, Cleft palate, Ventricular septal defect, Holoprosencephaly	7	1024	1.532899	14.66	0.774798		
LSS	Lanosterol Synthase	Protein Coding	Microphthalmia, Hypertelorism, Cleft lip, Cleft palate, Ventricular septal defect, Holoprosencephaly	6	1106	1.499443	12.67	0.484029		
PCNT	Pericentrin	Protein Coding	Microphthalmia, Hypertelorism, Micrognathia, Cleft lip, Cleft palate, Pectus excavatum, Ventricular septal defect, Holoprosencephaly	8	1382	1.402691	12.57	0.003366		
SLC19A1	Solute Carrier Family 19 Member 1	Protein Coding	Cleft lip, Cleft palate, Ventricular septal defect, Holoprosencephaly	4	926	1.576588	11.9	0.336461		
CSTB	Cystatin B	Protein Coding	Microphthalmia, Hypertelorism, Micrognathia, Cleft lip, Cleft palate, Pectus excavatum, Ventricular septal defect, Wide\-spaced nipples, Holoprosencephaly	9	1635	1.329681	11.6	0.733491		
DONSON	DNA Replication Fork Stabilization Factor DONSON	Protein Coding	Microphthalmia, Micrognathia, Cleft lip, Cleft palate	4	961	1.560475	11.57	0.404012		
RUNX1	RUNX Family Transcription Factor 1	Protein Coding	Hypertelorism, Micrognathia, Cleft lip, Cleft palate, Pectus excavatum, Ventricular septal defect	6	1287	1.43362	11.34	0.491055		
TRAPPC10	Trafficking Protein Particle Complex Subunit 10	Protein Coding	Microphthalmia, Hypertelorism, Micrognathia, Cleft lip, Cleft palate, Ventricular septal defect, Holoprosencephaly	7	1459	1.379143	11.2	0.50591		
SIK1	Salt Inducible Kinase 1	Protein Coding	Hypertelorism, Micrognathia, Cleft lip, Cleft palate, Ventricular septal defect	5	1241	1.449427	10.64	0.269487		
IFNAR1	Interferon Alpha And Beta Receptor Subunit 1	Protein Coding	Microphthalmia, Hypertelorism, Micrognathia, Cleft lip, Cleft palate, Pectus excavatum, Ventricular septal defect, Holoprosencephaly	8	1883	1.268348	9.9	0.343348		
S100B	S100 Calcium Binding Protein B	Protein Coding	Microphthalmia, Hypertelorism, Micrognathia, Cleft lip, Cleft palate, Pectus excavatum, Ventricular septal defect	7	2008	1.240435	8.69	0.728072		
KCNE1	Potassium Voltage-Gated Channel Subfamily E Regulatory Subunit 1	Protein Coding	Microphthalmia, Hypertelorism, Micrognathia, Cleft lip, Cleft palate, Pectus excavatum, Ventricular septal defect, Wide\-spaced nipples	8	2178	1.205141	8.58	0.531162		
CFAP410	Cilia And Flagella Associated Protein 410	Protein Coding	Hypertelorism, Micrognathia, Cleft lip, Cleft palate, Pectus excavatum	5	1806	1.286481	8.09	0.192971		
WDR4	WD Repeat Domain 4	Protein Coding	Microphthalmia, Hypertelorism, Micrognathia, Cleft lip, Cleft palate	5	1843	1.277673	7.96	0.248393		
SON	SON DNA And RNA Binding Protein	Protein Coding	Hypertelorism, Micrognathia, Cleft lip, Cleft palate, Ventricular septal defect	5	1978	1.246972	7.48	0.594756		
SYNJ1	Synaptojanin 1	Protein Coding	Microphthalmia, Hypertelorism, Micrognathia, Cleft lip, Cleft palate, Ventricular septal defect, Wide\-spaced nipples	7	2392	1.164437	7.42	0.428713		
SIM2	SIM BHLH Transcription Factor 2	Protein Coding	Microphthalmia, Hypertelorism, Cleft lip, Cleft palate, Ventricular septal defect, Holoprosencephaly	6	2192	1.202358	7.37	0.487994		
OLIG2	Oligodendrocyte Transcription Factor 2	Protein Coding	Hypertelorism, Micrognathia, Cleft lip, Cleft palate, Pectus excavatum, Ventricular septal defect	6	2210	1.198806	7.33	0		
KCNE2	Potassium Voltage-Gated Channel Subfamily E Regulatory Subunit 2	Protein Coding	Microphthalmia, Hypertelorism, Micrognathia, Cleft lip, Cleft palate, Pectus excavatum, Ventricular septal defect, Wide\-spaced nipples	8	2935	1.07559	6.57	0.592285		
PRDM15	PR/SET Domain 15	Protein Coding	Microphthalmia, Hypertelorism, Cleft lip, Cleft palate, Ventricular septal defect, Holoprosencephaly	6	2683	1.114578	6.2	0.573968		
DNMT3L	DNA Methyltransferase 3 Like	Protein Coding	Microphthalmia, Hypertelorism, Micrognathia, Cleft lip, Cleft palate, Ventricular septal defect, Holoprosencephaly	7	3551	0.992848	5.07	0.253846		
HMGN1	High Mobility Group Nucleosome Binding Domain 1	Protein Coding	Hypertelorism, Micrognathia, Cleft lip, Cleft palate, Pectus excavatum	5	3014	1.064055	5.06	0.247919		
HUNK	Hormonally Up-Regulated Neu-Associated Kinase	Protein Coding	Microphthalmia, Hypertelorism, Cleft lip, Cleft palate, Pectus excavatum, Wide\-spaced nipples	6	3624	0.98401	4.62	0.324749		
GART	Phosphoribosylglycinamide Formyltransferase, Phosphoribosylglycinamide Synthetase, Phosphoribosylaminoimidazole Synthetase	Protein Coding	Hypertelorism, Micrognathia, Cleft lip, Cleft palate	4	3120	1.049044	4.38	0.296779		
SCAF4	SR-Related CTD Associated Factor 4	Protein Coding	Hypertelorism, Micrognathia, Pectus excavatum, Ventricular septal defect	4	3580	0.989316	3.8	0.768482		
MCM3AP	Minichromosome Maintenance Complex Component 3 Associated Protein	Protein Coding	Hypertelorism, Micrognathia, Cleft lip, Cleft palate, Ventricular septal defect	5	4022	0.938757	3.75	0.498954		
ZBTB21	Zinc Finger And BTB Domain Containing 21	Protein Coding	Hypertelorism, Cleft lip, Cleft palate, Ventricular septal defect	4	3670	0.978533	3.7	0.536187		
HLCS	Holocarboxylase Synthetase	Protein Coding	Hypertelorism, Micrognathia, Cleft lip, Cleft palate, Ventricular septal defect	5	4201	0.919846	3.57	0.636247		
SMIM11	Small Integral Membrane Protein 11	Protein Coding	Microphthalmia, Cleft lip, Cleft palate, Ventricular septal defect	4	4006	0.940488	3.38	0.61613		
CRYZL1	Crystallin Zeta Like 1	Protein Coding	Hypertelorism, Micrognathia, Cleft lip, Cleft palate, Ventricular septal defect	5	4423	0.897482	3.33	0.620438		
PIGP	Phosphatidylinositol Glycan Anchor Biosynthesis Class P	Protein Coding	Cleft lip, Cleft palate, Ventricular septal defect	3	3578	0.989558	3.29	0.216345		
TSPEAR	Thrombospondin Type Laminin G Domain And EAR Repeats	Protein Coding	Microphthalmia, Hypertelorism, Micrognathia, Cleft palate, Ventricular septal defect	5	4640	0.876681	3.16	0.266227		
ITSN1	Intersectin 1	Protein Coding	Cleft lip, Cleft palate, Ventricular septal defect	3	4175	0.922542	2.79	0.664135		
SUMO3	Small Ubiquitin Like Modifier 3	Protein Coding	Microphthalmia, Cleft lip, Cleft palate	3	4337	0.906009	2.66	0.67305		
TMPRSS2	Transmembrane Serine Protease 2	Protein Coding	Hypertelorism, Micrognathia, Pectus excavatum, Ventricular septal defect, Wide\-spaced nipples	5	5394	0.811288	2.53	0.315989		
IFNGR2	Interferon Gamma Receptor 2	Protein Coding	Microphthalmia, Hypertelorism, Micrognathia, Cleft palate, Holoprosencephaly	5	5438	0.807759	2.49	0.402133		
TMPRSS3	Transmembrane Serine Protease 3	Protein Coding	Cleft lip, Cleft palate, Pectus excavatum	3	4759	0.865683	2.33	0.158363		
URB1	URB1 Ribosome Biogenesis Homolog	Protein Coding	Cleft palate, Pectus excavatum	2	4555	0.88471	2.06	0		
KCNJ6	Potassium Inwardly Rectifying Channel Subfamily J Member 6	Protein Coding	Hypertelorism, Micrognathia, Cleft palate	3	5464	0.805688	1.91	0.883012		
CRYAA	Crystallin Alpha A	Protein Coding	Microphthalmia, Cleft palate	2	5047	0.840165	1.8	0.7352		
FTCD	Formimidoyltransferase Cyclodeaminase	Protein Coding	Cleft lip, Cleft palate	2	5194	0.827697	1.71	0.162659		
ERG	ETS Transcription Factor ERG	Protein Coding	Hypertelorism, Micrognathia, Pectus excavatum, Ventricular septal defect	4	6396	0.73729	1.69	0.830623		
ATP5PO	ATP Synthase Peripheral Stalk Subunit OSCP	Protein Coding	Hypertelorism, Micrognathia, Ventricular septal defect	3	6608	0.723129	1.35	0.334732		
MORC3	MORC Family CW-Type Zinc Finger 3	Protein Coding	Micrognathia, Cleft palate	2	5943	0.769193	1.33	0.807403		
MX1	MX Dynamin Like GTPase 1	Protein Coding	Micrognathia, Cleft palate	2	5943	0.769193	1.33	0.43731		
CLDN14	Claudin 14	Protein Coding	Hypertelorism, Micrognathia, Cleft palate	3	6658	0.719855	1.32	0.534441		
SETD4	SET Domain Containing 4	Protein Coding	Micrognathia, Cleft palate, Ventricular septal defect	3	6687	0.717967	1.3	0.547486		
TFF2	Trefoil Factor 2	Protein Coding	Microphthalmia, Micrognathia	2	6063	0.760511	1.28	0.36645		
U2AF1	U2 Small Nuclear RNA Auxiliary Factor 1	Protein Coding	Hypertelorism, Micrognathia, Cleft palate	3	7346	0.677148	1.25	0.77858		
AIRE	Autoimmune Regulator	Protein Coding	Hypertelorism, Pectus excavatum, Ventricular septal defect	3	7462	0.670343	1.21	0.259796		
H2BC12L	H2B Clustered Histone 12 Like	Protein Coding	Microphthalmia, Hypertelorism, Micrognathia, Cleft palate	4	8048	0.637511	1.09	0		
IMMTP1	Inner Membrane Mitochondrial Protein Pseudogene 1	Pseudogene	Microphthalmia, Hypertelorism, Micrognathia, Cleft palate	4	8048	0.637511	1.09	0		
CHAF1B	Chromatin Assembly Factor 1 Subunit B	Protein Coding	Microphthalmia, Hypertelorism, Micrognathia, Cleft palate	4	8048	0.637511	1.09	0.55132		
BACE2	Beta-Secretase 2	Protein Coding	Cleft palate, Ventricular septal defect	2	6643	0.720834	1.08	0.848639		
ADARB1	Adenosine Deaminase RNA Specific B1	Protein Coding	Cleft palate, Ventricular septal defect, Holoprosencephaly	3	7905	0.645297	1.01	0.865561		
GATD3	Glutamine Amidotransferase Class 1 Domain Containing 3	Protein Coding	Hypertelorism, Cleft lip, Ventricular septal defect	3	8025	0.638754	0.96	0.308967		
TIAM1	TIAM Rac1 Associated GEF 1	Protein Coding	Ventricular septal defect	1	7491	0.668659	0.69	0.460805		
DSCAM	DS Cell Adhesion Molecule	Protein Coding	Hypertelorism, Ventricular septal defect	2	8565	0.610471	0.66	0.741066		
RCAN1	Regulator Of Calcineurin 1	Protein Coding	Ventricular septal defect	1	7616	0.661472	0.65	0.663669		
FAM3B	FAM3 Metabolism Regulating Signaling Molecule B	Protein Coding	Cleft lip, Cleft palate	2	8924	0.592639	0.55	0.32568		
LINC01671	Long Intergenic Non-Protein Coding RNA 1671	RNA Gene	Cleft lip	1	9245	0.577292	0.35	0		
PDE9A	Phosphodiesterase 9A	Protein Coding	Cleft lip	1	9245	0.577292	0.35	0.666175		
ITGB2	Integrin Subunit Beta 2	Protein Coding	Microphthalmia, Micrognathia	2	10463	0.523542	0.32	0.714501		
PWP2	PWP2 Small Subunit Processome Component	Protein Coding	Hypertelorism, Holoprosencephaly	2	10479	0.522879	0.32	0.241039		
DCR	Down Syndrome Chromosome Region	Genetic Locus	Ventricular septal defect	1	9847	0.549895	0.31	0		
MIR802	MicroRNA 802	RNA Gene	Ventricular septal defect	1	9847	0.549895	0.31	0		
VPS26C	VPS26 Endosomal Protein Sorting Factor C	Protein Coding	Ventricular septal defect	1	10025	0.542114	0.29	0.712495		
DSCR4	Down Syndrome Critical Region 4	RNA Gene	Ventricular septal defect	1	10025	0.542114	0.29	0.595025		
SPATC1L	Spermatogenesis And Centriole Associated 1 Like	Protein Coding	Microphthalmia, Hypertelorism	2	10771	0.510943	0.27	0.119095		
POFUT2	Protein O-Fucosyltransferase 2	Protein Coding	Cleft lip	1	10688	0.514302	0.2	0.434408		
ETS2	ETS Proto-Oncogene 2, Transcription Factor	Protein Coding	Hypertelorism	1	10756	0.511548	0.19	0.656116		
IFNAR2	Interferon Alpha And Beta Receptor Subunit 2	Protein Coding	Cleft palate	1	10775	0.510781	0.19	0.422986		
IGSF5	Immunoglobulin Superfamily Member 5	Protein Coding	Cleft lip	1	11076	0.498816	0.14	0.107325		
KCNJ6-AS1	KCNJ6 Antisense RNA 1	RNA Gene	Micrognathia	1	11108	0.497563	0.13	0		
UBASH3A	Ubiquitin Associated And SH3 Domain Containing A	Protein Coding	Microphthalmia	1	11130	0.496703	0.13	0.271878		
KCNJ15	Potassium Inwardly Rectifying Channel Subfamily J Member 15	Protein Coding	Microphthalmia	1	11130	0.496703	0.13	0.434616		
TRPM2	Transient Receptor Potential Cation Channel Subfamily M Member 2	Protein Coding	Holoprosencephaly	1	11469	0.483673	0.11	0.128253		
ICOSLG	Inducible T Cell Costimulator Ligand	Protein Coding	Micrognathia	1	11516	0.481897	0.11	0.271789		
DIP2A	Disco Interacting Protein 2 Homolog A	Protein Coding	Micrognathia	1	11639	0.477283	0.1	0.607098		
SLC37A1	Solute Carrier Family 37 Member 1	Protein Coding	Hypertelorism	1	11856	0.46926	0.1	0.468879		
KRTAP10-12	Keratin Associated Protein 10-12	Protein Coding	Hypertelorism	1	11856	0.46926	0.1	0.110913		
TSPEAR-AS1	TSPEAR Antisense RNA 1	RNA Gene	Microphthalmia	1	12292	0.453576	0.07	0		

Appendix 4 

**Table 3:** Functional annotation of structural variants

AnnotSV_ID	SV_chrom	SV_start	SV_end	SV_length	SV_type	Samples_ID	ID	REF	ALT	QUAL	FILTER	INFO	FORMAT	ILS_AP001	ILS_AP001_1	ILS_AP001_2	Annotation_mode	CytoBand	Gene_name	Closest_left	Closest_right	Gene_count	Tx	Tx_version	Tx_start	Tx_end	Overlapped_tx_length	Overlapped_CDS_length	Overlapped_CDS_percent	Frameshift	Exon_count	Location	Location2	Dist_nearest_SS	Nearest_SS_type	Intersect_start	Intersect_end	RE_gene	P_gain_phen	P_gain_hpo	P_gain_source	P_gain_coord	P_loss_phen	P_loss_hpo	P_loss_source	P_loss_coord	P_ins_phen	P_ins_hpo	P_ins_source	P_ins_coord	po_P_gain_phen	po_P_gain_hpo	po_P_gain_source	po_P_gain_coord	po_P_gain_percent	po_P_loss_phen	po_P_loss_hpo	po_P_loss_source	po_P_loss_coord	po_P_loss_percent	P_snvindel_nb	P_snvindel_phen	B_gain_source	B_gain_coord	B_gain_AFmax	B_loss_source	B_loss_coord	B_loss_AFmax	B_ins_source	B_ins_coord	B_ins_AFmax	B_inv_source	B_inv_coord	B_inv_AFmax	po_B_gain_allG_source	po_B_gain_allG_coord	po_B_gain_someG_source	po_B_gain_someG_coord	po_B_loss_allG_source	po_B_loss_allG_coord	po_B_loss_someG_source	po_B_loss_someG_coord	GC_content_left	GC_content_right	Repeat_coord_left	Repeat_type_left	Repeat_coord_right	Repeat_type_right	Gap_left	Gap_right	SegDup_left	SegDup_right	ENCODE_blacklist_left	ENCODE_blacklist_characteristics_left	ENCODE_blacklist_right	ENCODE_blacklist_characteristics_right	ACMG	HI	TS	DDD_HI_percent	ExAC_delZ	ExAC_dupZ	ExAC_cnvZ	ExAC_synZ	ExAC_misZ	GenCC_disease	GenCC_moi	GenCC_classification	GenCC_pmid	NCBI_gene_ID	OMIM_ID	OMIM_phenotype	OMIM_inheritance	OMIM_morbid	OMIM_morbid_candidate	LOEUF_bin	GnomAD_pLI	ExAC_pLI	Exomiser_gene_pheno_score	Human_pheno_evidence	Mouse_pheno_evidence	Fish_pheno_evidence	AnnotSV_ranking_score	AnnotSV_ranking_criteria	ACMG_class	ILS_AP001_2
12_107608877_107608984_DEL_1	12	1.08E+08	1.08E+08	-106	DEL	ILS_AP001,ILS_AP001_1,ILS_AP001_2	MantaDEL:22826:0:0:0:0:0	TTAAAATAAAATAAAATAAATAAAATAAAATAAAATAAAATAAAATAAAATAAAATAAAATAAAATAAATAAAATAAAATATAAAATAAAATATAAAATAAAATAAA	T	780	PASS	SUPP=3;SUPP_VEC=111;SVLEN=-106;SVTYPE=DEL;SVMETHOD=SURVIVOR1.0.7;CHR2=12;END=107608983;CIPOS=0,28;CIEND=0,1;STRANDS=+;Max_AF=0;Max_Het=0;Max_HomAlt=0;Max_PopMax_AF=0;CCDG_Count=0;Best_CCDG_Mismatch_ID=186910_1;Best_CCDG_Mismatch_OFP=0.00934579;Best_CCDG_Mismatch_SVTYPE=BND;Best_CCDG_Mismatch_AF=0.1459;Best_CCDG_Mismatch_Het=0;Best_CCDG_Mismatch_HomAlt=0;SV_Cov=1;CCDG_SV_Cov=0;TOPMed_Count=0;Best_TOPMed_ID=DEL_12:107608906-107608976;Best_TOPMed_OFP=0.672897;Best_TOPMed_AF=0.170773;Best_TOPMed_Het=29656;Best_TOPMed_HomAlt=3748;Best_TOPMed_PopMax_AF=0.383399;Best_TOPMed_Mismatch_ID=INV_12:107505762-107866958;Best_TOPMed_Mismatch_OFP=0.000296236;Best_TOPMed_Mismatch_SVTYPE=INV;Best_TOPMed_Mismatch_AF=4e-06;Best_TOPMed_Mismatch_Het=1;Best_TOPMed_Mismatch_HomAlt=0;TOPMed_SV_Cov=0.672897;ThousG_Count=0;ThousG_SV_Cov=0;gnomAD_Count=0;Best_gnomAD_ID=gnomAD-SV_v3_DEL_chr12_b0743d0d;Best_gnomAD_OFP=0.186736;Best_gnomAD_AF=0.132533;Best_gnomAD_Het=15993;Best_gnomAD_HomAlt=305;Best_gnomAD_PopMax_AF=0.283157;Best_gnomAD_Mismatch_ID=gnomAD-SV_v3_INS_chr12_5965628a;Best_gnomAD_Mismatch_OFP=0.141663;Best_gnomAD_Mismatch_SVTYPE=INS;Best_gnomAD_Mismatch_AF=8.9e-05;Best_gnomAD_Mismatch_Het=11;Best_gnomAD_Mismatch_HomAlt=0;gnomAD_SV_Cov=1	GT:PSV:LN:DR:ST:QV:TY:ID:RAL:AAL:CO	0/1:NA:107:0,0:+:780:DEL:DEL00038933:NA:NA:chr12_107608877-chr12_107608984	0/1:NA:106:0,0:+:694:DEL:MantaDEL_22826_0_0_0_0_0:TTAAAATAAAATAAAATAAATAAAATAAAATAAAATAAAATAAAATAAAATAAAATAAAATAAAATAAATAAAATAAAATATAAAATAAAATATAAAATAAAATAAA:T:chr12_107608877-chr12_107608983	0/1:NA:78:0,0:+:380:DEL:7348:NA:NA:chr12_107608905-chr12_107608983	split	q23.3	ABTB3				NM_001018072	2	1.07E+08	1.08E+08	108	0	0	no	17	intron4-intron4	CDS	1205	3'	1.08E+08	1.08E+08																																			IMH	12:40539485-113039119	0.1121																																				121551									0						full=3	0/1
10_1560472_1560587_DEL_1	10	1560472	1560587	-114	DEL	ILS_AP001,ILS_AP001_1,ILS_AP001_2	MantaDEL:36333:0:0:0:0:0	CACCCTGGGTCGTCACACACGCGTGAACACACGGGGGGCACCCTGGGTCGTCACACACGCGTGAACACACGGGGGGCACCCTGGGTCGTCACACACGCGTGAACACACGGGGGGT	C	802	PASS	SUPP=3;SUPP_VEC=111;SVLEN=-114;SVTYPE=DEL;SVMETHOD=SURVIVOR1.0.7;CHR2=10;END=1560586;CIPOS=0,0;CIEND=-40,1;STRANDS=+;Max_AF=0;Max_Het=0;Max_HomAlt=0;Max_PopMax_AF=0;CCDG_Count=0;Best_CCDG_ID=143996;Best_CCDG_OFP=0.652174;Best_CCDG_AF=0.644;Best_CCDG_Het=0;Best_CCDG_HomAlt=0;Best_CCDG_PopMax_AF=0;Best_CCDG_Mismatch_ID=461311;Best_CCDG_Mismatch_OFP=0.000281707;Best_CCDG_Mismatch_SVTYPE=DUP;Best_CCDG_Mismatch_AF=6.839e-05;Best_CCDG_Mismatch_Het=0;Best_CCDG_Mismatch_HomAlt=0;SV_Cov=1;CCDG_SV_Cov=0.652174;TOPMed_Count=0;Best_TOPMed_ID=DEL_10:1560473-1560546;Best_TOPMed_OFP=0.652174;Best_TOPMed_AF=0.796027;Best_TOPMed_Het=36576;Best_TOPMed_HomAlt=63698;Best_TOPMed_PopMax_AF=0.945155;Best_TOPMed_Mismatch_ID=DUP_10:1544002-1577700;Best_TOPMed_Mismatch_OFP=0.00341246;Best_TOPMed_Mismatch_SVTYPE=DUP;Best_TOPMed_Mismatch_AF=7e-06;Best_TOPMed_Mismatch_Het=2;Best_TOPMed_Mismatch_HomAlt=0;TOPMed_SV_Cov=1;ThousG_Count=0;Best_ThousG_ID=HGSV_13251;Best_ThousG_OFP=0.652174;Best_ThousG_AF=0.622854;Best_ThousG_Het=1628;Best_ThousG_HomAlt=1181;Best_ThousG_PopMax_AF=0.742928;ThousG_SV_Cov=0.652174;gnomAD_Count=0;Best_gnomAD_ID=gnomAD-SV_v3_DEL_chr10_cf2eea75;Best_gnomAD_OFP=0.652174;Best_gnomAD_AF=0.501124;Best_gnomAD_Het=45144;Best_gnomAD_HomAlt=7973;Best_gnomAD_PopMax_AF=0.584797;Best_gnomAD_Mismatch_ID=gnomAD-SV_v3_DUP_chr10_8c7c0436;Best_gnomAD_Mismatch_OFP=0.733823;Best_gnomAD_Mismatch_SVTYPE=DUP;Best_gnomAD_Mismatch_AF=0.044062;Best_gnomAD_Mismatch_Het=4474;Best_gnomAD_Mismatch_HomAlt=9;gnomAD_SV_Cov=1	GT:PSV:LN:DR:ST:QV:TY:ID:RAL:AAL:CO	1/1:NA:115:0,0:+:480:DEL:DEL00030007:NA:NA:chr10_1560472-chr10_1560587	1/1:NA:114:0,0:+:802:DEL:MantaDEL_36333_0_0_0_0_0:CACCCTGGGTCGTCACACACGCGTGAACACACGGGGGGCACCCTGGGTCGTCACACACGCGTGAACACACGGGGGGCACCCTGGGTCGTCACACACGCGTGAACACACGGGGGGT:C:chr10_1560472-chr10_1560586	1/1:NA:74:0,0:+:561:DEL:5864:NA:NA:chr10_1560472-chr10_1560546	split	p15.3	ADARB2				NM_018702	4	1177312	1737525	116	0	0	no	10	intron1-intron1	CDS	176463	5'	1560471	1560587																																																															43.56	-1.24883	-2.53125	-2.42223	0.97635	2.868325					105						1	8.56E-01	7.38E-01	0						full=3	1/1
11_26289563_26289683_DEL_1	11	26289563	26289683	-119	DEL	ILS_AP001,ILS_AP001_1,ILS_AP001_2	MantaDEL:28029:0:1:0:0:0	TACATATACACATATATCCTATATGTGTGTATATGTACATATACACACATATATTCTATATGTGTGTATATGTACATATACACACATATATTCTATATGTGTGTATATGTACATATACAC	T	534	PASS	SUPP=3;SUPP_VEC=111;SVLEN=-119;SVTYPE=DEL;SVMETHOD=SURVIVOR1.0.7;CHR2=11;END=26289682;CIPOS=0,7;CIEND=-35,1;STRANDS=+;Max_AF=0;Max_Het=0;Max_HomAlt=0;Max_PopMax_AF=0;CCDG_Count=0;Best_CCDG_Mismatch_ID=552441;Best_CCDG_Mismatch_OFP=0.512821;Best_CCDG_Mismatch_SVTYPE=INV;Best_CCDG_Mismatch_AF=0.0002393;Best_CCDG_Mismatch_Het=0;Best_CCDG_Mismatch_HomAlt=0;SV_Cov=1;CCDG_SV_Cov=0;TOPMed_Count=0;Best_TOPMed_ID=DEL_11:26168982-26306759;Best_TOPMed_OFP=0.00087096;Best_TOPMed_AF=4e-06;Best_TOPMed_Het=1;Best_TOPMed_HomAlt=0;Best_TOPMed_PopMax_AF=1.20192e-05;Best_TOPMed_Mismatch_ID=DUP_11:25900270-26725298;Best_TOPMed_Mismatch_OFP=0.000145449;Best_TOPMed_Mismatch_SVTYPE=DUP;Best_TOPMed_Mismatch_AF=7e-06;Best_TOPMed_Mismatch_Het=2;Best_TOPMed_Mismatch_HomAlt=0;TOPMed_SV_Cov=1;ThousG_Count=0;Best_ThousG_ID=HGSV_22611;Best_ThousG_OFP=0.666667;Best_ThousG_AF=0.012901;Best_ThousG_Het=80;Best_ThousG_HomAlt=1;Best_ThousG_PopMax_AF=0.030354;Best_ThousG_Mismatch_ID=HGSV_21276;Best_ThousG_Mismatch_OFP=1.75885e-06;Best_ThousG_Mismatch_SVTYPE=INV;Best_ThousG_Mismatch_AF=0.413492;Best_ThousG_Mismatch_Het=2648;Best_ThousG_Mismatch_HomAlt=0;ThousG_SV_Cov=1;gnomAD_Count=0;Best_gnomAD_ID=gnomAD-SV_v3_DEL_chr11_c1e64f6d;Best_gnomAD_OFP=0.701754;Best_gnomAD_AF=0.00694;Best_gnomAD_Het=867;Best_gnomAD_HomAlt=2;Best_gnomAD_PopMax_AF=0.024629;Best_gnomAD_Mismatch_ID=gnomAD-SV_v3_DUP_chr11_2e30ec4e;Best_gnomAD_Mismatch_OFP=0.00388489;Best_gnomAD_Mismatch_SVTYPE=DUP;Best_gnomAD_Mismatch_AF=8e-06;Best_gnomAD_Mismatch_Het=1;Best_gnomAD_Mismatch_HomAlt=0;gnomAD_SV_Cov=1	GT:PSV:LN:DR:ST:QV:TY:ID:RAL:AAL:CO	0/1:NA:120:0,0:+:342:DEL:DEL00034436:NA:NA:chr11_26289563-chr11_26289683	0/1:NA:119:0,0:+:534:DEL:MantaDEL_28029_0_1_0_0_0:TACATATACACATATATCCTATATGTGTGTATATGTACATATACACACATATATTCTATATGTGTGTATATGTACATATACACACATATATTCTATATGTGTGTATATGTACATATACAC:T:chr11_26289563-chr11_26289682	0/1:NA:77:0,0:+:333:DEL:6464:NA:NA:chr11_26289570-chr11_26289647	split	p14.2	ANO3				NM_001313726	2	26188807	26663289	121	0	0	no	28	intron1-intron1	CDS	19961	3'	26289562	26289683																													dbVar;gnomAD-SV_v3_DEL_chr11_f71dd9b5	chr11:8142635-35706287;chr11:26289521-26290235	0.048				IMH	11:1621661-71538522	0.0179																										26.37				0.115654	2.632122	dystonia 24	AD	Strong;Supportive	23200863;23200863[PMID];24151159;24442708;25356970;25847575;32116979;33388357;34307749	63982	610110	Dystonia 24, 615034 (3) AD	AD	yes		4	1.25E-17	4.09E-13	0						full=1	0/1
9_94833891_94833964_DEL_1	9	94833891	94833964	-72	DEL	ILS_AP001,ILS_AP001_1,ILS_AP001_2	MantaDEL:210906:0:1:0:0:0	TGAGAGTATTATTTTTTGCAACTCGTAAATCATTAATGGATCTAGGCAATGATCATTTTGTGTTTCCTAATGG	T	780	PASS	SUPP=3;SUPP_VEC=111;SVLEN=-72;SVTYPE=DEL;SVMETHOD=SURVIVOR1.0.7;CHR2=9;END=94833963;CIPOS=0,3;CIEND=0,1;STRANDS=+;Max_AF=0.008931;Max_Het=1691;Max_HomAlt=116;Max_PopMax_AF=0.024843;CCDG_Count=0;Best_CCDG_Mismatch_ID=433601_2;Best_CCDG_Mismatch_OFP=0.0136986;Best_CCDG_Mismatch_SVTYPE=BND;Best_CCDG_Mismatch_AF=0.00701;Best_CCDG_Mismatch_Het=0;Best_CCDG_Mismatch_HomAlt=0;SV_Cov=1;CCDG_SV_Cov=0;TOPMed_Count=1;Best_TOPMed_ID=DEL_9:94833892-94833963;Best_TOPMed_OFP=1;Best_TOPMed_AF=0.006613;Best_TOPMed_Het=1691;Best_TOPMed_HomAlt=0;Best_TOPMed_PopMax_AF=0.0105446;Best_TOPMed_Mismatch_ID=INV_9:94710576-96645135;Best_TOPMed_Mismatch_OFP=3.77347e-05;Best_TOPMed_Mismatch_SVTYPE=INV;Best_TOPMed_Mismatch_AF=4e-06;Best_TOPMed_Mismatch_Het=1;Best_TOPMed_Mismatch_HomAlt=0;TOPMed_SV_Cov=1;ThousG_Count=1;Best_ThousG_ID=HGSV_163726;Best_ThousG_OFP=0.958904;Best_ThousG_AF=0.003122;Best_ThousG_Het=16;Best_ThousG_HomAlt=2;Best_ThousG_PopMax_AF=0.01183;ThousG_SV_Cov=0.958904;gnomAD_Count=1;Best_gnomAD_ID=gnomAD-SV_v3_DEL_chr9_19fb53af;Best_gnomAD_OFP=0.958904;Best_gnomAD_AF=0.008931;Best_gnomAD_Het=894;Best_gnomAD_HomAlt=116;Best_gnomAD_PopMax_AF=0.024843;Best_gnomAD_Mismatch_ID=gnomAD-SV_v3_CPX_chr9_d1c64a8f;Best_gnomAD_Mismatch_OFP=0.00498668;Best_gnomAD_Mismatch_SVTYPE=CPX;Best_gnomAD_Mismatch_AF=8e-06;Best_gnomAD_Mismatch_Het=1;Best_gnomAD_Mismatch_HomAlt=0;gnomAD_SV_Cov=1	GT:PSV:LN:DR:ST:QV:TY:ID:RAL:AAL:CO	0/1:NA:73:0,0:+:780:DEL:DEL00028776:NA:NA:chr9_94833891-chr9_94833964	0/1:NA:72:0,0:+:448:DEL:MantaDEL_210906_0_1_0_0_0:TGAGAGTATTATTTTTTGCAACTCGTAAATCATTAATGGATCTAGGCAATGATCATTTTGTGTTTCCTAATGG:T:chr9_94833891-chr9_94833963	0/1:NA:69:0,0:+:404:DEL:5645:NA:NA:chr9_94833894-chr9_94833963	split	q22.32	AOPEP				XM_011519130	3	94726698	94875323	74	0	0	no	6	intron5-intron5	CDS	32888	5'	94833890	94833964																										dbVar	chr9:94691779-95015950	0.01							IMH	9:65437055-134286391;9:80897170-125694547;9:89671548-130954998;9:94308786-96947962	0.4714																																				84909	619600	Dystonia 31, 619565 (3) AR	AR	yes					0						full=3	0/1
11_941537_942183_DEL_1	11	941537	942183	-645	DEL	ILS_AP001,ILS_AP001_1,ILS_AP001_2	MantaDEL:25802:0:1:0:1:0	CTCCCAAAGTGCTGGGGTTACAGGCATGAGCCACTGTGGCTGGCTGCTTTCTTATTCTTTTTTTTTTTTGAGATGGAGTCTCACTCTGTTGCCCAGGCTGGAGTGCAGTGGCGTGATCTCGGCTCACTGCAATCTCTGACTCCCAGGTTCAAGCGATTCTCCCACCTCAGCCTCCCAAGTAGCTGGGACTACAGGCATGCACCACCACGCCCGGCTAATTTTTGTATTTTTAGTAGACATGGGGTTTTGCCATGTGGCTAGGCTGGTGTTGAACTCCTGATCTCAAGTGATCTGGTGGCCTTGGCCTCCCAAAGTGGTGGGATTACAAGCATGAACCACCATGCCCGGCCTCTCATGCTTAAATGAAGTAGTAATACCTGACCTTTTATTTTTTATCTATTTATATTTGAGATGGAGTCTCGCTCTGTCACCCAGGCTGGAGTGCAGTAGCGTGATCTTGGCTCACTGCAACCTCCGCCTCCCAGGTTCACTCCATTCTCCTGCCTCAGCCTCCTGAGTAGCTGGGACTACAGGCGCCCACCACCATGCCCGGCTAATTTTTTGTATTTTTAGTAGAGAGGGGGGTTTCACCCTGTTAGCCAGGATGGTCTCGATCTCCTGACCTCGTGATCTGCCCGCCTCGGCT	C	803	PASS	SUPP=3;SUPP_VEC=111;SVLEN=-645;SVTYPE=DEL;SVMETHOD=SURVIVOR1.0.7;CHR2=11;END=942182;CIPOS=0,33;CIEND=0,1;STRANDS=+;Max_AF=0.004036;Max_Het=503;Max_HomAlt=1;Max_PopMax_AF=0.005769;CCDG_Count=1;Best_CCDG_ID=158981;Best_CCDG_OFP=0.948916;Best_CCDG_AF=3.42e-05;Best_CCDG_Het=0;Best_CCDG_HomAlt=0;Best_CCDG_PopMax_AF=0;Best_CCDG_Mismatch_ID=465260;Best_CCDG_Mismatch_OFP=0.0522442;Best_CCDG_Mismatch_SVTYPE=DUP;Best_CCDG_Mismatch_AF=6.839e-05;Best_CCDG_Mismatch_Het=0;Best_CCDG_Mismatch_HomAlt=0;SV_Cov=1;CCDG_SV_Cov=0.948916;TOPMed_Count=0;Best_TOPMed_Mismatch_ID=DUP_11:857950-949631;Best_TOPMed_Mismatch_OFP=0.00704602;Best_TOPMed_Mismatch_SVTYPE=DUP;Best_TOPMed_Mismatch_AF=4e-06;Best_TOPMed_Mismatch_Het=1;Best_TOPMed_Mismatch_HomAlt=0;TOPMed_SV_Cov=0;ThousG_Count=1;Best_ThousG_ID=HGSV_21064;Best_ThousG_OFP=0.948916;Best_ThousG_AF=0.000156;Best_ThousG_Het=1;Best_ThousG_HomAlt=0;Best_ThousG_PopMax_AF=0.000832;Best_ThousG_Mismatch_ID=HGSV_21049;Best_ThousG_Mismatch_OFP=0.00345867;Best_ThousG_Mismatch_SVTYPE=DUP;Best_ThousG_Mismatch_AF=0.000156;Best_ThousG_Mismatch_Het=1;Best_ThousG_Mismatch_HomAlt=0;ThousG_SV_Cov=1;gnomAD_Count=2;Best_gnomAD_ID=gnomAD-SV_v3_DEL_chr11_938ba06e;Best_gnomAD_OFP=0.890093;Best_gnomAD_AF=0.000169;Best_gnomAD_Het=19;Best_gnomAD_HomAlt=1;Best_gnomAD_PopMax_AF=0.00161;Best_gnomAD_Mismatch_ID=gnomAD-SV_v3_DUP_chr11_2a0f5d05;Best_gnomAD_Mismatch_OFP=0.0523501;Best_gnomAD_Mismatch_SVTYPE=DUP;Best_gnomAD_Mismatch_AF=0.000119;Best_gnomAD_Mismatch_Het=15;Best_gnomAD_Mismatch_HomAlt=0;gnomAD_SV_Cov=0.995356	GT:PSV:LN:DR:ST:QV:TY:ID:RAL:AAL:CO	0/1:NA:646:20,6:+:803:DEL:DEL00033964:NA:NA:chr11_941537-chr11_942183	0/1:NA:645:0,0:+:370:DEL:MantaDEL_25802_0_1_0_1_0:CTCCCAAAGTGCTGGGGTTACAGGCATGAGCCACTGTGGCTGGCTGCTTTCTTATTCTTTTTTTTTTTTGAGATGGAGTCTCACTCTGTTGCCCAGGCTGGAGTGCAGTGGCGTGATCTCGGCTCACTGCAATCTCTGACTCCCAGGTTCAAGCGATTCTCCCACCTCAGCCTCCCAAGTAGCTGGGACTACAGGCATGCACCACCACGCCCGGCTAATTTTTGTATTTTTAGTAGACATGGGGTTTTGCCATGTGGCTAGGCTGGTGTTGAACTCCTGATCTCAAGTGATCTGGTGGCCTTGGCCTCCCAAAGTGGTGGGATTACAAGCATGAACCACCATGCCCGGCCTCTCATGCTTAAATGAAGTAGTAATACCTGACCTTTTATTTTTTATCTATTTATATTTGAGATGGAGTCTCGCTCTGTCACCCAGGCTGGAGTGCAGTAGCGTGATCTTGGCTCACTGCAACCTCCGCCTCCCAGGTTCACTCCATTCTCCTGCCTCAGCCTCCTGAGTAGCTGGGACTACAGGCGCCCACCACCATGCCCGGCTAATTTTTTGTATTTTTAGTAGAGAGGGGGGTTTCACCCTGTTAGCCAGGATGGTCTCGATCTCCTGACCTCGTGATCTGCCCGCCTCGGCT:C:chr11_941537-chr11_942182	0/1:NA:612:0,0:+:278:DEL:6370:NA:NA:chr11_941570-chr11_942182	split	p15.5	AP2A2				NM_001242837	2	925869	1012240	647	0	0	no	22	intron1-intron1	CDS	15448	5'	941536	942183																										dbVar	chr11:851409-1025480;chr11:857076-1025480;chr11:876861-979340;chr11:924172-1011626;chr11:939003-951343	0.01																																			72.44	0.24731	-0.33825	-0.15288	0.071349	4.232065	schizophrenia		Limited	23042115	161						0	1.0000e	1.00E+00	0						full=3	0/1
22_44860912_44860969_DEL_1	22	44860912	44860969	-56	DEL	ILS_AP001,ILS_AP001_1,ILS_AP001_2	MantaDEL:95612:0:1:0:0:0	TTGGAGAATGTCACTTCTTGGTTTTACAACAATTTTTTTTCTCCCTGTTGCTTGACA	T	653	PASS	SUPP=3;SUPP_VEC=111;SVLEN=-56;SVTYPE=DEL;SVMETHOD=SURVIVOR1.0.7;CHR2=22;END=44860968;CIPOS=0,1;CIEND=0,1;STRANDS=+;Max_AF=0.000781;Max_Het=18;Max_HomAlt=0;Max_PopMax_AF=0.004274;CCDG_Count=0;Best_CCDG_ID=308768;Best_CCDG_OFP=0.00374237;Best_CCDG_AF=3.419e-05;Best_CCDG_Het=0;Best_CCDG_HomAlt=0;Best_CCDG_PopMax_AF=0;Best_CCDG_Mismatch_ID=504733;Best_CCDG_Mismatch_OFP=0.00542289;Best_CCDG_Mismatch_SVTYPE=DUP;Best_CCDG_Mismatch_AF=3.419e-05;Best_CCDG_Mismatch_Het=0;Best_CCDG_Mismatch_HomAlt=0;SV_Cov=1;CCDG_SV_Cov=1;TOPMed_Count=0;Best_TOPMed_ID=DEL_22:44858643-44861351;Best_TOPMed_OFP=0.0210332;Best_TOPMed_AF=4e-06;Best_TOPMed_Het=1;Best_TOPMed_HomAlt=0;Best_TOPMed_PopMax_AF=6.26535e-06;Best_TOPMed_Mismatch_ID=INV_22:44757699-44872616;Best_TOPMed_Mismatch_OFP=0.000496002;Best_TOPMed_Mismatch_SVTYPE=INV;Best_TOPMed_Mismatch_AF=4e-06;Best_TOPMed_Mismatch_Het=1;Best_TOPMed_Mismatch_HomAlt=0;TOPMed_SV_Cov=1;ThousG_Count=1;Best_ThousG_ID=HGSV_96575;Best_ThousG_OFP=0.982456;Best_ThousG_AF=0.000781;Best_ThousG_Het=5;Best_ThousG_HomAlt=0;Best_ThousG_PopMax_AF=0.004274;Best_ThousG_Mismatch_ID=HGSV_96574;Best_ThousG_Mismatch_OFP=0.00901471;Best_ThousG_Mismatch_SVTYPE=CPX;Best_ThousG_Mismatch_AF=0.000156;Best_ThousG_Mismatch_Het=1;Best_ThousG_Mismatch_HomAlt=0;ThousG_SV_Cov=0.982456;gnomAD_Count=1;Best_gnomAD_ID=gnomAD-SV_v3_DEL_chr22_e53f8373;Best_gnomAD_OFP=0.982456;Best_gnomAD_AF=0.000143;Best_gnomAD_Het=18;Best_gnomAD_HomAlt=0;Best_gnomAD_PopMax_AF=0.0042;Best_gnomAD_Mismatch_ID=gnomAD-SV_v3_DUP_chr22_abddc20b;Best_gnomAD_Mismatch_OFP=0.00712411;Best_gnomAD_Mismatch_SVTYPE=DUP;Best_gnomAD_Mismatch_AF=1.7e-05;Best_gnomAD_Mismatch_Het=2;Best_gnomAD_Mismatch_HomAlt=0;gnomAD_SV_Cov=1	GT:PSV:LN:DR:ST:QV:TY:ID:RAL:AAL:CO	0/1:NA:57:0,0:+:653:DEL:DEL00062432:NA:NA:chr22_44860912-chr22_44860969	1/1:NA:56:0,0:+:486:DEL:MantaDEL_95612_0_1_0_0_0:TTGGAGAATGTCACTTCTTGGTTTTACAACAATTTTTTTTCTCCCTGTTGCTTGACA:T:chr22_44860912-chr22_44860968	0/1:NA:55:0,0:+:272:DEL:11382:NA:NA:chr22_44860913-chr22_44860968	split	q13.31	ARHGAP8				NM_001017526	2	44752574	44862784	58	0	0	no	13	intron12-intron12	CDS	1077	5'	44860911	44860969																													dbVar	chr22:44820754-44869341;chr22:44837184-44861234	0.01				IMH	22:44479419-47655346	0.2479																										79.74	-2.62354	0.465679	-1.01906							23779						8	8.52E-28		0.5199		Cleft lip;Cleft palate;Hypertelorism;Micrognathia;Microphthalmia;cataract				full=3	0/1
8_144279080_144279163_DEL_1	8	1.44E+08	1.44E+08	-82	DEL	ILS_AP001,ILS_AP001_1,ILS_AP001_2	MantaDEL:224320:0:0:0:0:0	TGACCACCAAGGGCCATGACCGTCACGGGCCACGACCGCCATGGGCCATGACCCCCATGGGCCTCAAGACCACCACAGGCCAC	T	790	PASS	SUPP=3;SUPP_VEC=111;SVLEN=-82;SVTYPE=DEL;SVMETHOD=SURVIVOR1.0.7;CHR2=8;END=144279162;CIPOS=0,8;CIEND=-19,1;STRANDS=+;Max_AF=0;Max_Het=0;Max_HomAlt=0;Max_PopMax_AF=0;CCDG_Count=0;Best_CCDG_Mismatch_ID=425360_1;Best_CCDG_Mismatch_OFP=0.0120482;Best_CCDG_Mismatch_SVTYPE=BND;Best_CCDG_Mismatch_AF=0.5376;Best_CCDG_Mismatch_Het=0;Best_CCDG_Mismatch_HomAlt=0;SV_Cov=1;CCDG_SV_Cov=0;TOPMed_Count=0;Best_TOPMed_ID=DEL_8:144279089-144279143;Best_TOPMed_OFP=0.674699;Best_TOPMed_AF=0.549235;Best_TOPMed_Het=67184;Best_TOPMed_HomAlt=39319;Best_TOPMed_PopMax_AF=0.624534;Best_TOPMed_Mismatch_ID=DUP_8:144257901-144309152;Best_TOPMed_Mismatch_OFP=0.00161942;Best_TOPMed_Mismatch_SVTYPE=DUP;Best_TOPMed_Mismatch_AF=4e-06;Best_TOPMed_Mismatch_Het=1;Best_TOPMed_Mismatch_HomAlt=0;TOPMed_SV_Cov=1;ThousG_Count=0;ThousG_SV_Cov=0;gnomAD_Count=0;Best_gnomAD_ID=gnomAD-SV_v3_DEL_chr8_77f5991f;Best_gnomAD_OFP=0.674699;Best_gnomAD_AF=0.412819;Best_gnomAD_Het=41699;Best_gnomAD_HomAlt=4427;Best_gnomAD_PopMax_AF=0.463041;Best_gnomAD_Mismatch_ID=gnomAD-SV_v3_DUP_chr8_4fc86a05;Best_gnomAD_Mismatch_OFP=0.244346;Best_gnomAD_Mismatch_SVTYPE=DUP;Best_gnomAD_Mismatch_AF=0.5;Best_gnomAD_Mismatch_Het=3;Best_gnomAD_Mismatch_HomAlt=0;gnomAD_SV_Cov=1	GT:PSV:LN:DR:ST:QV:TY:ID:RAL:AAL:CO	1/1:NA:83:0,0:+:780:DEL:DEL00027468:NA:NA:chr8_144279080-chr8_144279163	1/1:NA:82:0,0:+:790:DEL:MantaDEL_224320_0_0_0_0_0:TGACCACCAAGGGCCATGACCGTCACGGGCCACGACCGCCATGGGCCATGACCCCCATGGGCCTCAAGACCACCACAGGCCAC:T:chr8_144279080-chr8_144279162	1/1:NA:55:0,0:+:502:DEL:5409:NA:NA:chr8_144279088-chr8_144279143	split	q24.3	BOP1				NM_015201	5	1.44E+08	1.44E+08	84	0	0	no	16	intron2-intron2	CDS	2775	3'	1.44E+08	1.44E+08																																																															71.45				0.350937	0.12491					23246						7	9.28E-02	5.27E-01	0						full=3	1/1
17_52039252_52049002_DEL_1	17	52039252	52049002	-9749	DEL	ILS_AP001,ILS_AP001_1,ILS_AP001_2	MantaDEL:52948:0:1:0:0:0	A		1620	PASS	SUPP=3;SUPP_VEC=111;SVLEN=-9749;SVTYPE=DEL;SVMETHOD=SURVIVOR1.0.7;CHR2=17;END=52049001;CIPOS=0,2;CIEND=0,1;STRANDS=+;Max_AF=0.009991;Max_Het=710;Max_HomAlt=18;Max_PopMax_AF=0.052137;CCDG_Count=1;Best_CCDG_ID=239784;Best_CCDG_OFP=0.999795;Best_CCDG_AF=0.0008206;Best_CCDG_Het=0;Best_CCDG_HomAlt=0;Best_CCDG_PopMax_AF=0;Best_CCDG_Mismatch_ID=568590;Best_CCDG_Mismatch_OFP=0.000523327;Best_CCDG_Mismatch_SVTYPE=INV;Best_CCDG_Mismatch_AF=0.2957;Best_CCDG_Mismatch_Het=0;Best_CCDG_Mismatch_HomAlt=0;SV_Cov=1;CCDG_SV_Cov=0.999795;TOPMed_Count=5;Best_TOPMed_ID=DEL_17:52039255-52049004;Best_TOPMed_OFP=0.999487;Best_TOPMed_AF=0.002648;Best_TOPMed_Het=710;Best_TOPMed_HomAlt=18;Best_TOPMed_PopMax_AF=0.0457731;Best_TOPMed_Mismatch_ID=DUP_17:51966479-52066348;Best_TOPMed_Mismatch_OFP=0.0976259;Best_TOPMed_Mismatch_SVTYPE=DUP;Best_TOPMed_Mismatch_AF=4e-06;Best_TOPMed_Mismatch_Het=1;Best_TOPMed_Mismatch_HomAlt=0;TOPMed_SV_Cov=1;ThousG_Count=1;Best_ThousG_ID=HGSV_62744;Best_ThousG_OFP=0.999795;Best_ThousG_AF=0.009991;Best_ThousG_Het=62;Best_ThousG_HomAlt=1;Best_ThousG_PopMax_AF=0.052137;Best_ThousG_Mismatch_ID=HGSV_62408;Best_ThousG_Mismatch_OFP=0.000448843;Best_ThousG_Mismatch_SVTYPE=INV;Best_ThousG_Mismatch_AF=0.067926;Best_ThousG_Mismatch_Het=435;Best_ThousG_Mismatch_HomAlt=0;ThousG_SV_Cov=0.999795;gnomAD_Count=1;Best_gnomAD_ID=gnomAD-SV_v3_DEL_chr17_7b229558;Best_gnomAD_OFP=0.999795;Best_gnomAD_AF=0.002197;Best_gnomAD_Het=273;Best_gnomAD_HomAlt=2;Best_gnomAD_PopMax_AF=0.046867;Best_gnomAD_Mismatch_ID=gnomAD-SV_v3_DUP_chr17_1f0cd36f;Best_gnomAD_Mismatch_OFP=0.0976259;Best_gnomAD_Mismatch_SVTYPE=DUP;Best_gnomAD_Mismatch_AF=8e-06;Best_gnomAD_Mismatch_Het=1;Best_gnomAD_Mismatch_HomAlt=0;gnomAD_SV_Cov=1	GT:PSV:LN:DR:ST:QV:TY:ID:RAL:AAL:CO	0/1:NA:9750:19,15:+:1620:DEL:DEL00051442:NA:NA:chr17_52039252-chr17_52049002	0/1:NA:9749:0,0:+:829:DEL:MantaDEL_52948_0_1_0_0_0:NA:NA:chr17_52039252-chr17_52049001	0/1:NA:9747:0,0:+:650:DEL:9547:NA:NA:chr17_52039254-chr17_52049001	split	q21.33	CA10				NM_001082533	1	51630312	52159801	9751	0	0	no	10	intron3-intron3	CDS	23316	5'	52039251	52049002																													1000g;dbVar;esv3640777	chr17:52039118-52052439;chr17:52039251-52049003;17:52039252-52049003;17:52039252-52049003	0.0496				IMH	17:34039916-62479846;17:34040041-62479166;17:34040143-62479546;17:34040242-62479120;17:34040341-62478867;17:34040432-62479253;17:46268845-67992342;17:46287445-64926623;17:46291987-64922991	0.2957																										4.4	1.125011	0.861137	1.183256	-0.11718	1.13949					56934						2	5.67E-01	9.73E-01	0.4271			Microphthalmia;eye decreased size, abnormal			full=1	0/1
10_18141430_18162954_DUP_1	10	18141430	18162954	21524	DUP	ILS_AP001,ILS_AP001_1,ILS_AP001_2	MantaDUP:TANDEM:37963:0:1:0:0:0	T		1080	PASS	SUPP=3;SUPP_VEC=111;SVLEN=21524;SVTYPE=DUP;SVMETHOD=SURVIVOR1.0.7;CHR2=10;END=18162954;CIPOS=0,1;CIEND=0,0;STRANDS=-;Max_AF=0;Max_Het=0;Max_HomAlt=0;Max_PopMax_AF=0;CCDG_Count=0;Best_CCDG_Mismatch_ID=145959;Best_CCDG_Mismatch_OFP=0.00673635;Best_CCDG_Mismatch_SVTYPE=DEL;Best_CCDG_Mismatch_AF=0.000171;Best_CCDG_Mismatch_Het=0;Best_CCDG_Mismatch_HomAlt=0;SV_Cov=1;CCDG_SV_Cov=0;TOPMed_Count=0;Best_TOPMed_ID=DUP_10:17607298-18187851;Best_TOPMed_OFP=0.0370766;Best_TOPMed_AF=7e-06;Best_TOPMed_Het=2;Best_TOPMed_HomAlt=0;Best_TOPMed_PopMax_AF=1.25661e-05;Best_TOPMed_Mismatch_ID=DEL_10:18135902-18166700;Best_TOPMed_Mismatch_OFP=0.698864;Best_TOPMed_Mismatch_SVTYPE=DEL;Best_TOPMed_Mismatch_AF=4e-06;Best_TOPMed_Mismatch_Het=1;Best_TOPMed_Mismatch_HomAlt=0;TOPMed_SV_Cov=1;ThousG_Count=0;Best_ThousG_Mismatch_ID=HGSV_14289;Best_ThousG_Mismatch_OFP=0.00268136;Best_ThousG_Mismatch_SVTYPE=INV;Best_ThousG_Mismatch_AF=0.000156;Best_ThousG_Mismatch_Het=1;Best_ThousG_Mismatch_HomAlt=0;ThousG_SV_Cov=0;gnomAD_Count=0;Best_gnomAD_ID=gnomAD-SV_v3_DUP_chr10_a46e6116;Best_gnomAD_OFP=0.0219146;Best_gnomAD_AF=8e-06;Best_gnomAD_Het=1;Best_gnomAD_HomAlt=0;Best_gnomAD_PopMax_AF=3e-05;Best_gnomAD_Mismatch_ID=gnomAD-SV_v3_DEL_chr10_59bf6ec9;Best_gnomAD_Mismatch_OFP=0.115819;Best_gnomAD_Mismatch_SVTYPE=DEL;Best_gnomAD_Mismatch_AF=8e-06;Best_gnomAD_Mismatch_Het=1;Best_gnomAD_Mismatch_HomAlt=0;gnomAD_SV_Cov=1	GT:PSV:LN:DR:ST:QV:TY:ID:RAL:AAL:CO	0/1:NA:21523:41,8:-:1080:DUP:DUP00030417:NA:NA:chr10_18141431-chr10_18162954	0/1:NA:21524:0,0:-:470:DUP:MantaDUP_TANDEM_37963_0_1_0_0_0:NA:NA:chr10_18141430-chr10_18162954	0/1:NA:21524:0,0:-:212:DUP:5946:NA:NA:chr10_18141430-chr10_18162954	split	p12.33	CACNB2				XM_047425725	1	18140423	18518968	21525	93	11	no	10	intron1-intron2	CDS	573	5'	18141429	18162954																																																															18.41	0.627254	-0.88115	-0.42823	-1.11349	0.425036	Brugada syndrome 1;Brugada syndrome 4;hypertrophic cardiomyopathy;short QT syndrome	AD	Disputed Evidence;Limited	17224476;19358333;20817017;22584458;22840528;25637381;30662450	783	600003	Brugada syndrome 4, 611876 (3) AD	AD	yes		3	2.67E-06	1.82E-03	0						full=3	0/1
2_44409095_44409184_DEL_1	2	44409095	44409184	-88	DEL	ILS_AP001,ILS_AP001_1,ILS_AP001_2	MantaDEL:174423:0:0:0:0:0	ATATATATATATATATATATATATATATATATATATATATATATATATATATATATATATATATATATATATATATATATATATATATG	A	1140	PASS	SUPP=3;SUPP_VEC=111;SVLEN=-88;SVTYPE=DEL;SVMETHOD=SURVIVOR1.0.7;CHR2=2;END=44409183;CIPOS=0,5;CIEND=-15,1;STRANDS=+;Max_AF=0;Max_Het=0;Max_HomAlt=0;Max_PopMax_AF=0;CCDG_Count=0;Best_CCDG_ID=271444;Best_CCDG_OFP=0.00477493;Best_CCDG_AF=3.419e-05;Best_CCDG_Het=0;Best_CCDG_HomAlt=0;Best_CCDG_PopMax_AF=0;Best_CCDG_Mismatch_ID=271446_1;Best_CCDG_Mismatch_OFP=0.011236;Best_CCDG_Mismatch_SVTYPE=BND;Best_CCDG_Mismatch_AF=0.636;Best_CCDG_Mismatch_Het=0;Best_CCDG_Mismatch_HomAlt=0;SV_Cov=1;CCDG_SV_Cov=1;TOPMed_Count=0;Best_TOPMed_ID=DEL_2:44391632-44410277;Best_TOPMed_OFP=0.00477289;Best_TOPMed_AF=3.2e-05;Best_TOPMed_Het=9;Best_TOPMed_HomAlt=0;Best_TOPMed_PopMax_AF=0.000107787;Best_TOPMed_Mismatch_ID=INV_2:44403211-44419415;Best_TOPMed_Mismatch_OFP=0.00549179;Best_TOPMed_Mismatch_SVTYPE=INV;Best_TOPMed_Mismatch_AF=4e-06;Best_TOPMed_Mismatch_Het=1;Best_TOPMed_Mismatch_HomAlt=0;TOPMed_SV_Cov=1;ThousG_Count=0;Best_ThousG_ID=HGSV_77537;Best_ThousG_OFP=0.775281;Best_ThousG_AF=0.437305;Best_ThousG_Het=1391;Best_ThousG_HomAlt=703;Best_ThousG_PopMax_AF=0.627962;Best_ThousG_Mismatch_ID=HGSV_77475;Best_ThousG_Mismatch_OFP=6.63578e-07;Best_ThousG_Mismatch_SVTYPE=INS;Best_ThousG_Mismatch_AF=0.000468;Best_ThousG_Mismatch_Het=3;Best_ThousG_Mismatch_HomAlt=0;ThousG_SV_Cov=0.775281;gnomAD_Count=0;Best_gnomAD_ID=gnomAD-SV_v3_DEL_chr2_ac5169c0;Best_gnomAD_OFP=0.775281;Best_gnomAD_AF=0.424911;Best_gnomAD_Het=38541;Best_gnomAD_HomAlt=6924;Best_gnomAD_PopMax_AF=0.550847;gnomAD_Mismatches=gnomAD-SV_v3_DUP_chr2_a7918d70,gnomAD-SV_v3_DUP_chr2_d33e7116,gnomAD-SV_v3_DUP_chr2_a1ef2047;gnomAD_Mismatches_Count=3;gnomAD_Mismatch_SVTYPEs=DUP;Best_gnomAD_Mismatch_ID=gnomAD-SV_v3_DUP_chr2_a1ef2047;Best_gnomAD_Mismatch_OFP=0.828365;Best_gnomAD_Mismatch_SVTYPE=DUP;Best_gnomAD_Mismatch_AF=0.000226;Best_gnomAD_Mismatch_Het=20;Best_gnomAD_Mismatch_HomAlt=4;gnomAD_SV_Cov=1	GT:PSV:LN:DR:ST:QV:TY:ID:RAL:AAL:CO	1/1:NA:89:0,0:+:1140:DEL:DEL00006084:NA:NA:chr2_44409095-chr2_44409184	1/1:NA:88:0,0:+:732:DEL:MantaDEL_174423_0_0_0_0_0:ATATATATATATATATATATATATATATATATATATATATATATATATATATATATATATATATATATATATATATATATATATATATG:A:chr2_44409095-chr2_44409183	1/1:NA:68:0,0:+:699:DEL:1226:NA:NA:chr2_44409100-chr2_44409168	split	p21	CAMKMT				XM_047445879	1	44361946	44715353	90	0	0	no	6	intron3-intron3	CDS	18789	5'	44409094	44409184																										dbVar;gnomAD-SV_v3_DUP_chr2_d33e7116	chr2:44405812-44507018;chr2:44409080-44409188	0.0624	dbVar	chr2:44278237-44413378;chr2:44391631-44410269	0.01				IMH	2:21055811-76514955;2:32873521-53185851	0.1623																										16.48	-1.41796	-1.07939	-1.18635	-0.0005	-0.70187					79823						8	1.38E-15	5.15E-13	0.2756	2p21 microdeletion syndrome;Cleft palate;Depressed nasal bridge;Frontal bossing;Micrognathia					full=1	1/1
11_64341843_64341924_DEL_1	11	64341843	64341924	-80	DEL	ILS_AP001,ILS_AP001_1,ILS_AP001_2	MantaDEL:30764:0:1:0:0:0	GGTTGTGGTCTGAGGTCTTGGGCCATCAGTGATGTCACAACCAGATGGCCCAAGACCCCAGACCACAACCCCATGTCTGGT	G	480	PASS	SUPP=3;SUPP_VEC=111;SVLEN=-80;SVTYPE=DEL;SVMETHOD=SURVIVOR1.0.7;CHR2=11;END=64341923;CIPOS=0,8;CIEND=0,1;STRANDS=+;Max_AF=0.000519;Max_Het=65;Max_HomAlt=0;Max_PopMax_AF=0.000951;CCDG_Count=0;Best_CCDG_ID=166951;Best_CCDG_OFP=0.00442405;Best_CCDG_AF=3.419e-05;Best_CCDG_Het=0;Best_CCDG_HomAlt=0;Best_CCDG_PopMax_AF=0;Best_CCDG_Mismatch_ID=166953_2;Best_CCDG_Mismatch_OFP=0.0123457;Best_CCDG_Mismatch_SVTYPE=BND;Best_CCDG_Mismatch_AF=0.06302;Best_CCDG_Mismatch_Het=0;Best_CCDG_Mismatch_HomAlt=0;SV_Cov=1;CCDG_SV_Cov=1;TOPMed_Count=0;Best_TOPMed_ID=DEL_11:59466655-66168743;Best_TOPMed_OFP=1.20858e-05;Best_TOPMed_AF=4e-06;Best_TOPMed_Het=1;Best_TOPMed_HomAlt=0;Best_TOPMed_PopMax_AF=6.26354e-06;Best_TOPMed_Mismatch_ID=DUP_11:60405002-68536600;Best_TOPMed_Mismatch_OFP=9.96114e-06;Best_TOPMed_Mismatch_SVTYPE=DUP;Best_TOPMed_Mismatch_AF=4e-06;Best_TOPMed_Mismatch_Het=1;Best_TOPMed_Mismatch_HomAlt=0;TOPMed_SV_Cov=1;ThousG_Count=0;Best_ThousG_Mismatch_ID=HGSV_24190;Best_ThousG_Mismatch_OFP=2.06875e-06;Best_ThousG_Mismatch_SVTYPE=INV;Best_ThousG_Mismatch_AF=0.488601;Best_ThousG_Mismatch_Het=3129;Best_ThousG_Mismatch_HomAlt=0;ThousG_SV_Cov=0;gnomAD_Count=1;Best_gnomAD_ID=gnomAD-SV_v3_DEL_chr11_5ab5afbb;Best_gnomAD_OFP=0.901235;Best_gnomAD_AF=0.000519;Best_gnomAD_Het=65;Best_gnomAD_HomAlt=0;Best_gnomAD_PopMax_AF=0.000951;Best_gnomAD_Mismatch_ID=gnomAD-SV_v3_DUP_chr11_4e711591;Best_gnomAD_Mismatch_OFP=0.00309149;Best_gnomAD_Mismatch_SVTYPE=DUP;Best_gnomAD_Mismatch_AF=1.7e-05;Best_gnomAD_Mismatch_Het=0;Best_gnomAD_Mismatch_HomAlt=1;gnomAD_SV_Cov=1	GT:PSV:LN:DR:ST:QV:TY:ID:RAL:AAL:CO	0/1:NA:81:0,0:+:480:DEL:DEL00035331:NA:NA:chr11_64341843-chr11_64341924	0/1:NA:80:0,0:+:280:DEL:MantaDEL_30764_0_1_0_0_0:GGTTGTGGTCTGAGGTCTTGGGCCATCAGTGATGTCACAACCAGATGGCCCAAGACCCCAGACCACAACCCCATGTCTGGT:G:chr11_64341843-chr11_64341923	0/1:NA:72:0,0:+:197:DEL:6678:NA:NA:chr11_64341851-chr11_64341923	split	q13.1	CCDC88B				NM_032251	6	64340203	64357534	82	0	0	no	27	intron7-intron7	CDS	69	3'	64341842	64341924																																			IMH	11:1621661-71538522;11:49193342-89651496;11:49211577-89635120;11:49243027-89597882;11:49358811-89347590;11:49403038-89297530;11:49586833-89037283;11:49629782-88995227;11:49736709-88892563	0.4958																										77.95	0.163641	0.700084	0.590085	0.461565	0.989825					283234						2	1.40E-09	9.29E-04	0						full=3	0/1
3_112357974_112367723_DUP_1	3	1.12E+08	1.12E+08	9742	DUP	ILS_AP001,ILS_AP001_1,ILS_AP001_2	MantaDUP:TANDEM:164788:0:1:0:0:0	A		881	PASS	SUPP=3;SUPP_VEC=111;SVLEN=9742;SVTYPE=DUP;SVMETHOD=SURVIVOR1.0.7;CHR2=3;END=112367716;CIPOS=0,1;CIEND=0,7;STRANDS=-;Max_AF=0;Max_Het=0;Max_HomAlt=0;Max_PopMax_AF=0;CCDG_Count=0;Best_CCDG_ID=508907;Best_CCDG_OFP=0.00262431;Best_CCDG_AF=3.419e-05;Best_CCDG_Het=0;Best_CCDG_HomAlt=0;Best_CCDG_PopMax_AF=0;Best_CCDG_Mismatch_ID=584333;Best_CCDG_Mismatch_OFP=0.00205123;Best_CCDG_Mismatch_SVTYPE=INV;Best_CCDG_Mismatch_AF=6.839e-05;Best_CCDG_Mismatch_Het=0;Best_CCDG_Mismatch_HomAlt=0;SV_Cov=1;CCDG_SV_Cov=1;TOPMed_Count=0;Best_TOPMed_ID=DUP_3:112266702-112474400;Best_TOPMed_OFP=0.046909;Best_TOPMed_AF=4.7e-05;Best_TOPMed_Het=13;Best_TOPMed_HomAlt=0;Best_TOPMed_PopMax_AF=8.29431e-05;Best_TOPMed_Mismatch_ID=INV_3:111765091-113399244;Best_TOPMed_Mismatch_OFP=0.0059621;Best_TOPMed_Mismatch_SVTYPE=INV;Best_TOPMed_Mismatch_AF=4e-06;Best_TOPMed_Mismatch_Het=1;Best_TOPMed_Mismatch_HomAlt=0;TOPMed_SV_Cov=1;ThousG_Count=0;ThousG_SV_Cov=0;gnomAD_Count=0;Best_gnomAD_Mismatch_ID=gnomAD-SV_v3_DEL_chr3_846bc145;Best_gnomAD_Mismatch_OFP=0.0410551;Best_gnomAD_Mismatch_SVTYPE=DEL;Best_gnomAD_Mismatch_AF=0.000135;Best_gnomAD_Mismatch_Het=17;Best_gnomAD_Mismatch_HomAlt=0;gnomAD_SV_Cov=0	GT:PSV:LN:DR:ST:QV:TY:ID:RAL:AAL:CO	0/1:NA:9741:33,10:-:881:DUP:DUP00010845:NA:NA:chr3_112357975-chr3_112367716	0/1:NA:9742:0,0:-:666:DUP:MantaDUP_TANDEM_164788_0_1_0_0_0:NA:NA:chr3_112357974-chr3_112367716	0/1:NA:9749:0,0:-:155:DUP:2231:NA:NA:chr3_112357974-chr3_112367723	split	q13.2	CD200				NM_001365853	1	1.12E+08	1.12E+08	4839	8	1	yes	6	intron5-txEnd	CDS-3'UTR	3569	3'	1.12E+08	1.12E+08																													gnomAD-SV_v3_DEL_chr3_9b8d4286	chr3:11110259-145805573	0.0446				IMH	3:30036454-134858252;3:72502419-189537676;3:75447628-125901392	0.239																										65.55	0.220641	0.039405	0.154051	-0.07105	0.270677					4345						2	4.14E-01	1.60E-01	0.1903		Holoprosencephaly;abnormal microglial cell morphology				full=3	0/1
6_118691123_118692764_DEL_1	6	1.19E+08	1.19E+08	-1639	DEL	ILS_AP001,ILS_AP001_1,ILS_AP001_2	MantaDEL:127453:1:2:0:0:0	A		1613	PASS	SUPP=3;SUPP_VEC=111;SVLEN=-1639;SVTYPE=DEL;SVMETHOD=SURVIVOR1.0.7;CHR2=6;END=118692764;CIPOS=-2,0;CIEND=-9,0;STRANDS=+;Max_AF=0.002853;Max_Het=306;Max_HomAlt=0;Max_PopMax_AF=0.004399;CCDG_Count=0;Best_CCDG_ID=383030;Best_CCDG_OFP=0.04009;Best_CCDG_AF=3.419e-05;Best_CCDG_Het=0;Best_CCDG_HomAlt=0;Best_CCDG_PopMax_AF=0;Best_CCDG_Mismatch_ID=524510;Best_CCDG_Mismatch_OFP=0.741746;Best_CCDG_Mismatch_SVTYPE=DUP;Best_CCDG_Mismatch_AF=0.4692;Best_CCDG_Mismatch_Het=0;Best_CCDG_Mismatch_HomAlt=0;SV_Cov=1;CCDG_SV_Cov=1;TOPMed_Count=0;Best_TOPMed_ID=DEL_6:118690006-118698985;Best_TOPMed_OFP=0.182608;Best_TOPMed_AF=7.1e-05;Best_TOPMed_Het=20;Best_TOPMed_HomAlt=0;Best_TOPMed_PopMax_AF=0.000118978;Best_TOPMed_Mismatch_ID=INV_6:118677651-118746246;Best_TOPMed_Mismatch_OFP=0.0239078;Best_TOPMed_Mismatch_SVTYPE=INV;Best_TOPMed_Mismatch_AF=4e-06;Best_TOPMed_Mismatch_Het=1;Best_TOPMed_Mismatch_HomAlt=0;TOPMed_SV_Cov=1;ThousG_Count=0;Best_ThousG_ID=HGSV_136958;Best_ThousG_OFP=0.182669;Best_ThousG_AF=0.000156;Best_ThousG_Het=1;Best_ThousG_HomAlt=0;Best_ThousG_PopMax_AF=0.000789;Best_ThousG_Mismatch_ID=HGSV_136959;Best_ThousG_Mismatch_OFP=0.741746;Best_ThousG_Mismatch_SVTYPE=DUP;Best_ThousG_Mismatch_AF=0.446284;Best_ThousG_Mismatch_Het=456;Best_ThousG_Mismatch_HomAlt=1201;ThousG_SV_Cov=1;gnomAD_Count=1;Best_gnomAD_ID=gnomAD-SV_v3_DEL_chr6_730c34b1;Best_gnomAD_OFP=0.811881;Best_gnomAD_AF=0.002853;Best_gnomAD_Het=306;Best_gnomAD_HomAlt=0;Best_gnomAD_PopMax_AF=0.004399;Best_gnomAD_Mismatch_ID=gnomAD-SV_v3_DUP_chr6_0ee6261a;Best_gnomAD_Mismatch_OFP=0.539753;Best_gnomAD_Mismatch_SVTYPE=DUP;Best_gnomAD_Mismatch_AF=0.039098;Best_gnomAD_Mismatch_Het=3931;Best_gnomAD_Mismatch_HomAlt=5;gnomAD_SV_Cov=1	GT:PSV:LN:DR:ST:QV:TY:ID:RAL:AAL:CO	0/1:NA:1632:27,18:+:1613:DEL:DEL00020616:NA:NA:chr6_118691123-chr6_118692755	0/1:NA:1639:0,0:+:579:DEL:MantaDEL_127453_1_2_0_0_0:NA:NA:chr6_118691125-chr6_118692764	0/1:NA:1639:0,0:+:584:DEL:4087:NA:NA:chr6_118691125-chr6_118692764	split	q22.31	CEP85L				NM_001178035	2	1.18E+08	1.19E+08	1642	0	0	no	14	intron1-intron1	5'UTR	17271	5'	1.19E+08	1.19E+08																										CMRI:142_pbsv.SPLIT.DUP.1935;IMH;dbVar	chr6:118470570-118861466;chr6:118563166-118770310;6:118690556-118692765;6:118690557-118692765	0.4692	dbVar	chr6:118563166-118770310;chr6:118690042-118699122;chr6:118690263-118693049	0.01																																44.27	-0.87242	-2.53125	-2.47163	-1.75564	-0.84951	lissencephaly 10;lissencephaly due to LIS1 mutation	AD	Definitive;Strong	32097629;32097630;33096394	387119	618865	Lissencephaly 10, 618873 (3) AD	AD	yes		3	4.54E-09	4.96E-03	0.3527	Microphthalmia;Optic nerve hypoplasia;Posterior-predominant lissencephaly-broad flat pons and medulla-midline crossing defects syndrome					full=1	0/1
2_119659621_119660920_DEL_1	2	1.2E+08	1.2E+08	-1298	DEL	ILS_AP001,ILS_AP001_1,ILS_AP001_2	MantaDEL:179317:0:1:0:0:0	G		1245	PASS	SUPP=3;SUPP_VEC=111;SVLEN=-1298;SVTYPE=DEL;SVMETHOD=SURVIVOR1.0.7;CHR2=2;END=119660919;CIPOS=0,3;CIEND=0,1;STRANDS=+;Max_AF=0;Max_Het=0;Max_HomAlt=0;Max_PopMax_AF=0;CCDG_Count=0;Best_CCDG_ID=279347;Best_CCDG_OFP=0.00104813;Best_CCDG_AF=3.419e-05;Best_CCDG_Het=0;Best_CCDG_HomAlt=0;Best_CCDG_PopMax_AF=0;Best_CCDG_Mismatch_ID=497353;Best_CCDG_Mismatch_OFP=0.00373295;Best_CCDG_Mismatch_SVTYPE=DUP;Best_CCDG_Mismatch_AF=3.419e-05;Best_CCDG_Mismatch_Het=0;Best_CCDG_Mismatch_HomAlt=0;SV_Cov=1;CCDG_SV_Cov=1;TOPMed_Count=0;Best_TOPMed_ID=DEL_2:119592602-119681700;Best_TOPMed_OFP=0.0145791;Best_TOPMed_AF=4e-06;Best_TOPMed_Het=1;Best_TOPMed_HomAlt=0;Best_TOPMed_PopMax_AF=0.00010661;Best_TOPMed_Mismatch_ID=DUP_2:119610102-119662000;Best_TOPMed_Mismatch_OFP=0.0250289;Best_TOPMed_Mismatch_SVTYPE=DUP;Best_TOPMed_Mismatch_AF=4e-06;Best_TOPMed_Mismatch_Het=1;Best_TOPMed_Mismatch_HomAlt=0;TOPMed_SV_Cov=1;ThousG_Count=0;ThousG_Mismatches=HGSV_81537;ThousG_Mismatches_Count=1;ThousG_Mismatch_SVTYPEs=DUP;Best_ThousG_Mismatch_ID=HGSV_81537;Best_ThousG_Mismatch_OFP=0.916725;Best_ThousG_Mismatch_SVTYPE=DUP;Best_ThousG_Mismatch_AF=0.7297;Best_ThousG_Mismatch_Het=1131;Best_ThousG_Mismatch_HomAlt=1771;ThousG_SV_Cov=0;gnomAD_Count=0;Best_gnomAD_ID=gnomAD-SV_v3_DEL_chr2_b8d7b14a;Best_gnomAD_OFP=0.00289823;Best_gnomAD_AF=1.6e-05;Best_gnomAD_Het=2;Best_gnomAD_HomAlt=0;Best_gnomAD_PopMax_AF=0.000161;Best_gnomAD_Mismatch_ID=gnomAD-SV_v3_INS_chr2_11e51f62;Best_gnomAD_Mismatch_OFP=0.00623557;Best_gnomAD_Mismatch_SVTYPE=INS;Best_gnomAD_Mismatch_AF=0.000676;Best_gnomAD_Mismatch_Het=81;Best_gnomAD_Mismatch_HomAlt=2;gnomAD_SV_Cov=1	GT:PSV:LN:DR:ST:QV:TY:ID:RAL:AAL:CO	0/1:NA:1299:21,12:+:1245:DEL:DEL00007394:NA:NA:chr2_119659621-chr2_119660920	0/1:NA:1298:0,0:+:848:DEL:MantaDEL_179317_0_1_0_0_0:NA:NA:chr2_119659621-chr2_119660919	0/1:NA:1295:0,0:+:521:DEL:1472:NA:NA:chr2_119659624-chr2_119660919	split	q14.2	CFAP221				XM_006712353	4	1.2E+08	1.2E+08	703	0	0	no	25	intron24-txEnd	3'UTR	549	3'	1.2E+08	1.2E+08																										dbVar	chr2:119496017-119698511;chr2:119607424-119746424	0.01	dbVar;gnomAD-SV_v3_DEL_chr2_8881e883	chr2:106014408-123234450;chr2:119062422-123333424	0.2151				IMH	2:65359567-213949290;2:94555625-132220070	0.3179																																primary ciliary dyskinesia	AD;AR	Limited;Moderate;Supportive	31636325[PMID]	200373									0						full=1	0/1
9_132421610_132421687_DEL_1	9	1.32E+08	1.32E+08	-76	DEL	ILS_AP001,ILS_AP001_1,ILS_AP001_2	MantaDEL:213820:0:0:0:0:0	TGAGAATCCTGGGTTCTCATGCTCTGGTTTCTCTTCAAATCGTATAAATCTTTCGCCTTTTACTAAAGATTTCTGTG	T	1020	PASS	SUPP=3;SUPP_VEC=111;SVLEN=-76;SVTYPE=DEL;SVMETHOD=SURVIVOR1.0.7;CHR2=9;END=132421686;CIPOS=0,16;CIEND=0,1;STRANDS=+;Max_AF=0;Max_Het=0;Max_HomAlt=0;Max_PopMax_AF=0;CCDG_Count=0;Best_CCDG_ID=437382;Best_CCDG_OFP=0.792208;Best_CCDG_AF=0.9596;Best_CCDG_Het=0;Best_CCDG_HomAlt=0;Best_CCDG_PopMax_AF=0;Best_CCDG_Mismatch_ID=537439;Best_CCDG_Mismatch_OFP=0.00224949;Best_CCDG_Mismatch_SVTYPE=DUP;Best_CCDG_Mismatch_AF=3.419e-05;Best_CCDG_Mismatch_Het=0;Best_CCDG_Mismatch_HomAlt=0;SV_Cov=0.792208;CCDG_SV_Cov=0.792208;TOPMed_Count=0;Best_TOPMed_Mismatch_ID=DUP_9:132104708-132425204;Best_TOPMed_Mismatch_OFP=0.000240251;Best_TOPMed_Mismatch_SVTYPE=DUP;Best_TOPMed_Mismatch_AF=4e-06;Best_TOPMed_Mismatch_Het=1;Best_TOPMed_Mismatch_HomAlt=0;TOPMed_SV_Cov=0;ThousG_Count=0;Best_ThousG_ID=HGSV_165893;Best_ThousG_OFP=0.792208;Best_ThousG_AF=0.927835;Best_ThousG_Het=424;Best_ThousG_HomAlt=2758;Best_ThousG_PopMax_AF=0.968454;ThousG_SV_Cov=0.792208;gnomAD_Count=0;Best_gnomAD_ID=gnomAD-SV_v3_DEL_chr9_d530eb63;Best_gnomAD_OFP=0.792208;Best_gnomAD_AF=0.731839;Best_gnomAD_Het=33391;Best_gnomAD_HomAlt=29444;Best_gnomAD_PopMax_AF=0.78125;Best_gnomAD_Mismatch_ID=gnomAD-SV_v3_DUP_chr9_494359e1;Best_gnomAD_Mismatch_OFP=0.00224949;Best_gnomAD_Mismatch_SVTYPE=DUP;Best_gnomAD_Mismatch_AF=8e-06;Best_gnomAD_Mismatch_Het=1;Best_gnomAD_Mismatch_HomAlt=0;gnomAD_SV_Cov=0.792208	GT:PSV:LN:DR:ST:QV:TY:ID:RAL:AAL:CO	1/1:NA:77:0,0:+:1020:DEL:DEL00029746:NA:NA:chr9_132421610-chr9_132421687	1/1:NA:76:0,0:+:999:DEL:MantaDEL_213820_0_0_0_0_0:TGAGAATCCTGGGTTCTCATGCTCTGGTTTCTCTTCAAATCGTATAAATCTTTCGCCTTTTACTAAAGATTTCTGTG:T:chr9_132421610-chr9_132421686	1/1:NA:60:0,0:+:827:DEL:5815:NA:NA:chr9_132421626-chr9_132421686	split	q34.13	CFAP77				XM_011518671	2	1.32E+08	1.33E+08	78	0	0	no	4	intron1-intron1	CDS	11143	5'	1.32E+08	1.32E+08																																			IMH	9:65437055-134286391	0.2414																																				389799									0						full=3	1/1
8_93405628_93405764_DEL_1	8	93405628	93405764	-123	DEL	ILS_AP001,ILS_AP001_1,ILS_AP001_2	MantaDEL:184904:1:1:0:0:0	TATATATATATATATATATATATATATGTATATATATATATATATGTATATATATATATATATGTATATATATATATATATGTATATATATATATATATATATATGTGTATATATATATATATA	TG	840	PASS	SUPP=3;SUPP_VEC=111;SVLEN=-123;SVTYPE=DEL;SVMETHOD=SURVIVOR1.0.7;CHR2=8;END=93405764;CIPOS=-13,9;CIEND=-14,0;STRANDS=+;Max_AF=0;Max_Het=0;Max_HomAlt=0;Max_PopMax_AF=0;CCDG_Count=0;Best_CCDG_Mismatch_ID=112287_1;Best_CCDG_Mismatch_OFP=0.00806452;Best_CCDG_Mismatch_SVTYPE=BND;Best_CCDG_Mismatch_AF=0.0006497;Best_CCDG_Mismatch_Het=0;Best_CCDG_Mismatch_HomAlt=0;SV_Cov=1;CCDG_SV_Cov=0;TOPMed_Count=0;Best_TOPMed_ID=DEL_8:93375834-93553436;Best_TOPMed_OFP=0.000698182;Best_TOPMed_AF=7e-06;Best_TOPMed_Het=2;Best_TOPMed_HomAlt=0;Best_TOPMed_PopMax_AF=0.000213174;Best_TOPMed_Mismatch_ID=INV_8:93056502-93610923;Best_TOPMed_Mismatch_OFP=0.000223656;Best_TOPMed_Mismatch_SVTYPE=INV;Best_TOPMed_Mismatch_AF=4e-06;Best_TOPMed_Mismatch_Het=1;Best_TOPMed_Mismatch_HomAlt=0;TOPMed_SV_Cov=1;ThousG_Count=0;Best_ThousG_Mismatch_ID=HGSV_153347;Best_ThousG_Mismatch_OFP=2.18579e-06;Best_ThousG_Mismatch_SVTYPE=CTX;Best_ThousG_Mismatch_AF=0.000156;Best_ThousG_Mismatch_Het=1;Best_ThousG_Mismatch_HomAlt=0;ThousG_SV_Cov=0;gnomAD_Count=0;Best_gnomAD_ID=gnomAD-SV_v3_DEL_chr8_4c808af8;Best_gnomAD_OFP=0.172709;Best_gnomAD_AF=0.014802;Best_gnomAD_Het=1343;Best_gnomAD_HomAlt=0;Best_gnomAD_PopMax_AF=0.034483;gnomAD_Mismatches=gnomAD-SV_v3_DUP_chr8_25e7e38e;gnomAD_Mismatches_Count=1;gnomAD_Mismatch_SVTYPEs=DUP;Best_gnomAD_Mismatch_ID=gnomAD-SV_v3_DUP_chr8_25e7e38e;Best_gnomAD_Mismatch_OFP=0.813172;Best_gnomAD_Mismatch_SVTYPE=DUP;Best_gnomAD_Mismatch_AF=0.001429;Best_gnomAD_Mismatch_Het=180;Best_gnomAD_Mismatch_HomAlt=0;gnomAD_SV_Cov=1	GT:PSV:LN:DR:ST:QV:TY:ID:RAL:AAL:CO	0/1:NA:123:0,0:+:840:DEL:DEL00026665:NA:NA:chr8_93405628-chr8_93405751	0/1:NA:123:0,0:+:632:DEL:MantaDEL_184904_1_1_0_0_0:TATATATATATATATATATATATATATGTATATATATATATATATGTATATATATATATATATGTATATATATATATATATGTATATATATATATATATATATATGTGTATATATATATATATA:TG:chr8_93405641-chr8_93405764	0/1:NA:100:0,0:+:425:DEL:5227:NA:NA:chr8_93405650-chr8_93405750	split	q22.1	CIBAR1-DT				NR_033858	1	93346466	93700433	137	0	0	no	11	intron7-intron7	UTR	14404	3'	93405627	93405764																																																																									642924									0						full=3	0/1
5_79750489_79753717_INV_1	5	79750489	79753717	3228	INV	ILS_AP001,ILS_AP001_1,ILS_AP001_2	MantaBND:136633:0:1:0:0:0:0	T	T]chr5:79753717]	1140	PASS	SUPP=3;SUPP_VEC=111;SVLEN=3228;SVTYPE=INV;SVMETHOD=SURVIVOR1.0.7;CHR2=5;END=79753717;CIPOS=0,4;CIEND=0,0;STRANDS=;Max_AF=0;Max_Het=0;Max_HomAlt=0;Max_PopMax_AF=0;CCDG_Count=0;Best_CCDG_ID=592532;Best_CCDG_OFP=0.000123398;Best_CCDG_AF=3.419e-05;Best_CCDG_Het=0;Best_CCDG_HomAlt=0;Best_CCDG_PopMax_AF=0;Best_CCDG_Mismatch_ID=359580_1;Best_CCDG_Mismatch_OFP=0.000309693;Best_CCDG_Mismatch_SVTYPE=BND;Best_CCDG_Mismatch_AF=0.05519;Best_CCDG_Mismatch_Het=0;Best_CCDG_Mismatch_HomAlt=0;SV_Cov=1;CCDG_SV_Cov=1;TOPMed_Count=0;Best_TOPMed_ID=INV_5:79227384-80480748;Best_TOPMed_OFP=0.00257626;Best_TOPMed_AF=4e-06;Best_TOPMed_Het=1;Best_TOPMed_HomAlt=0;Best_TOPMed_PopMax_AF=6.25986e-06;Best_TOPMed_Mismatch_ID=DEL_5:79752602-79756800;Best_TOPMed_Mismatch_OFP=0.0920003;Best_TOPMed_Mismatch_SVTYPE=DEL;Best_TOPMed_Mismatch_AF=4e-06;Best_TOPMed_Mismatch_Het=1;Best_TOPMed_Mismatch_HomAlt=0;TOPMed_SV_Cov=1;ThousG_Count=0;Best_ThousG_ID=HGSV_123838;Best_ThousG_OFP=9.76245e-05;Best_ThousG_AF=0.000156;Best_ThousG_Het=1;Best_ThousG_HomAlt=0;Best_ThousG_PopMax_AF=0;Best_ThousG_Mismatch_ID=HGSV_124085;Best_ThousG_Mismatch_OFP=0.583906;Best_ThousG_Mismatch_SVTYPE=CPX;Best_ThousG_Mismatch_AF=0.081798;Best_ThousG_Mismatch_Het=478;Best_ThousG_Mismatch_HomAlt=23;ThousG_SV_Cov=1;gnomAD_Count=0;Best_gnomAD_ID=gnomAD-SV_v3_INV_chr5_29b515bc;Best_gnomAD_OFP=0.000123398;Best_gnomAD_AF=8e-06;Best_gnomAD_Het=1;Best_gnomAD_HomAlt=0;Best_gnomAD_PopMax_AF=3e-05;gnomAD_Mismatches=gnomAD-SV_v3_CPX_chr5_dbd26d18;gnomAD_Mismatches_Count=1;gnomAD_Mismatch_SVTYPEs=CPX;Best_gnomAD_Mismatch_ID=gnomAD-SV_v3_CPX_chr5_dbd26d18;Best_gnomAD_Mismatch_OFP=0.938936;Best_gnomAD_Mismatch_SVTYPE=CPX;Best_gnomAD_Mismatch_AF=0.014205;Best_gnomAD_Mismatch_Het=1776;Best_gnomAD_Mismatch_HomAlt=0;gnomAD_SV_Cov=1	GT:PSV:LN:DR:ST:QV:TY:ID:RAL:AAL:CO	0/1:NA:3228:28,9::1140:INV:INV00016940:NA:NA:chr5_79750489-chr5_79753717	0/1:NA:3228:0,0::336:INV:MantaBND_136633_0_1_0_0_0_0:NA:NA:chr5_79750489-chr5_79753717	0/1:NA:3224:0,0::260:INV:3323_1:NA:NA:chr5_79750493-chr5_79753717	split	q14.1	CMYA5				NM_153610	5	79689835	79800222	3229	119	0	yes	13	intron5-intron6	CDS	923	5'	79750488	79753717																										dbVar	chr5:79531454-79772168;chr5:79672056-79773955	0.01	dbVar	chr5:79531454-79772168;chr5:79672056-79773955	0.01				IMH	5:24758176-102357714;5:40738518-84864090;5:58813595-102383104	0.0576																										77.74	-0.42279	0.206259	-0.01032	-0.72954	-2.63571					202333						4	1.35E-54	3.81E-43	0.377		Holoprosencephaly;abnormal cerebellum morphology				full=NA	0/1
10_123888363_123889122_DEL_1	10	1.24E+08	1.24E+08	-758	DEL	ILS_AP001,ILS_AP001_1,ILS_AP001_2	MantaDEL:46419:0:1:0:0:0	GAACCCCTCACTCCCACCTCAGTGCCTCATACATAGCATTGCATCATCCACCCAGTGAAGCAAAGTACAGAAGTTTGCATTAAACACTCCAGGCCACAGTCCAATGTTCCTTCCTATGTAAGTGCCTTCCTAAGATATTCCAAAACAATCCAATGCAGAGATAAGTGCTTCTATTACACCACGAGCAGCATATAGATCAAGCCGCTACTTAAAGTAGAAAAAAAATAATTTTCTCCTAGTGTAGTTTAACTGCACAAAACTGTCACTGATTCCCCTAACTAAGTTCACGCTTAAACCAGTCTTCCCACTTTGGTCCAGGGCAGAGGATTCTGCTGAGCAAACACCAAAATAAAAAAGGACAGGAGTTAAAAATATGAGATAAATGTCACACTTGTCTTGTCATGGTTGGGGAGAGCTCTAAAAAAGCCCCACAGAATTCATTAGAAACACAGCTGACATGGTAGCAGGTCTGCAGGCAGCTGTCCCAACAGGCCTCTGATGTGAGTCTCTAGATCTTCCGAAGACGTGTTTTCAGTATGGGGCTTGGAGATCCCGGTGGATGCCAGGGCTGCGGTGAACTGGGCAATGTGTCCTGCAGGAGCAGAGGCTAGGCACCGAATCAACAGGCTCCCCCATGGCCTGTCTCTACCTGCAAGATGGCCTTCCAAAACCAAATGAGCTTTTTCTTGCAAATTCAGGTTACACTCTGATTTCTCCTCCAGAGACCCCATTCCAAGAGTGGCAAGAGCTGTTTCTCCA	G	1560	PASS	SUPP=3;SUPP_VEC=111;SVLEN=-758;SVTYPE=DEL;SVMETHOD=SURVIVOR1.0.7;CHR2=10;END=123889121;CIPOS=0,1;CIEND=0,1;STRANDS=+;Max_AF=3.419e-05;Max_Het=3;Max_HomAlt=0;Max_PopMax_AF=0.000454;CCDG_Count=1;Best_CCDG_ID=156990;Best_CCDG_OFP=0.998682;Best_CCDG_AF=3.419e-05;Best_CCDG_Het=0;Best_CCDG_HomAlt=0;Best_CCDG_PopMax_AF=0;SV_Cov=1;CCDG_SV_Cov=0.998682;TOPMed_Count=0;Best_TOPMed_ID=DEL_10:121934616-128427106;Best_TOPMed_OFP=0.000116904;Best_TOPMed_AF=4e-06;Best_TOPMed_Het=1;Best_TOPMed_HomAlt=0;Best_TOPMed_PopMax_AF=6.28844e-06;Best_TOPMed_Mismatch_ID=DUP_10:123775202-124006800;Best_TOPMed_Mismatch_OFP=0.0032772;Best_TOPMed_Mismatch_SVTYPE=DUP;Best_TOPMed_Mismatch_AF=4e-06;Best_TOPMed_Mismatch_Het=1;Best_TOPMed_Mismatch_HomAlt=0;TOPMed_SV_Cov=1;ThousG_Count=0;ThousG_SV_Cov=0;gnomAD_Count=1;Best_gnomAD_ID=gnomAD-SV_v3_DEL_chr10_028858dd;Best_gnomAD_OFP=0.998682;Best_gnomAD_AF=2.4e-05;Best_gnomAD_Het=3;Best_gnomAD_HomAlt=0;Best_gnomAD_PopMax_AF=0.000454;Best_gnomAD_Mismatch_ID=gnomAD-SV_v3_DUP_chr10_ad8d2382;Best_gnomAD_Mismatch_OFP=0.0484087;Best_gnomAD_Mismatch_SVTYPE=DUP;Best_gnomAD_Mismatch_AF=8e-06;Best_gnomAD_Mismatch_Het=1;Best_gnomAD_Mismatch_HomAlt=0;gnomAD_SV_Cov=1	GT:PSV:LN:DR:ST:QV:TY:ID:RAL:AAL:CO	0/1:NA:759:16,13:+:1560:DEL:DEL00033705:NA:NA:chr10_123888363-chr10_123889122	0/1:NA:758:0,0:+:898:DEL:MantaDEL_46419_0_1_0_0_0:GAACCCCTCACTCCCACCTCAGTGCCTCATACATAGCATTGCATCATCCACCCAGTGAAGCAAAGTACAGAAGTTTGCATTAAACACTCCAGGCCACAGTCCAATGTTCCTTCCTATGTAAGTGCCTTCCTAAGATATTCCAAAACAATCCAATGCAGAGATAAGTGCTTCTATTACACCACGAGCAGCATATAGATCAAGCCGCTACTTAAAGTAGAAAAAAAATAATTTTCTCCTAGTGTAGTTTAACTGCACAAAACTGTCACTGATTCCCCTAACTAAGTTCACGCTTAAACCAGTCTTCCCACTTTGGTCCAGGGCAGAGGATTCTGCTGAGCAAACACCAAAATAAAAAAGGACAGGAGTTAAAAATATGAGATAAATGTCACACTTGTCTTGTCATGGTTGGGGAGAGCTCTAAAAAAGCCCCACAGAATTCATTAGAAACACAGCTGACATGGTAGCAGGTCTGCAGGCAGCTGTCCCAACAGGCCTCTGATGTGAGTCTCTAGATCTTCCGAAGACGTGTTTTCAGTATGGGGCTTGGAGATCCCGGTGGATGCCAGGGCTGCGGTGAACTGGGCAATGTGTCCTGCAGGAGCAGAGGCTAGGCACCGAATCAACAGGCTCCCCCATGGCCTGTCTCTACCTGCAAGATGGCCTTCCAAAACCAAATGAGCTTTTTCTTGCAAATTCAGGTTACACTCTGATTTCTCCTCCAGAGACCCCATTCCAAGAGTGGCAAGAGCTGTTTCTCCA:G:chr10_123888363-chr10_123889121	0/1:NA:757:0,0:+:794:DEL:6318:NA:NA:chr10_123888364-chr10_123889121	split	q26.13	CPXM2				NM_198148	3	1.24E+08	1.24E+08	760	0	0	no	14	intron1-intron1	CDS	2233	5'	1.24E+08	1.24E+08																													dbVar	chr10:121962383-124521347	0.01																																55.19	-0.63744	0.863002	0.269837	1.988612	0.415814					119587						5	5.49E-13	1.83E-10	0						full=1	0/1
19_4005348_4159440_DEL_1	19	4005348	4159440	-154090	DEL	ILS_AP001,ILS_AP001_1,ILS_AP001_2	MantaDEL:79083:0:1:0:0:0	C		1740	PASS	SUPP=3;SUPP_VEC=111;SVLEN=-154090;SVTYPE=DEL;SVMETHOD=SURVIVOR1.0.7;CHR2=19;END=4159440;CIPOS=-2,0;CIEND=-5,0;STRANDS=+;Max_AF=0;Max_Het=0;Max_HomAlt=0;Max_PopMax_AF=0;CCDG_Count=0;Best_CCDG_ID=253950;Best_CCDG_OFP=0.113861;Best_CCDG_AF=6.839e-05;Best_CCDG_Het=0;Best_CCDG_HomAlt=0;Best_CCDG_PopMax_AF=0;Best_CCDG_Mismatch_ID=490213;Best_CCDG_Mismatch_OFP=0.150976;Best_CCDG_Mismatch_SVTYPE=DUP;Best_CCDG_Mismatch_AF=3.419e-05;Best_CCDG_Mismatch_Het=0;Best_CCDG_Mismatch_HomAlt=0;SV_Cov=0.735014;CCDG_SV_Cov=0.290815;TOPMed_Count=0;Best_TOPMed_ID=DEL_19:4130566-4146337;Best_TOPMed_OFP=0.102362;Best_TOPMed_AF=4e-06;Best_TOPMed_Het=1;Best_TOPMed_HomAlt=0;Best_TOPMed_PopMax_AF=0.000108719;Best_TOPMed_Mismatch_ID=INV_19:3851264-4364229;Best_TOPMed_Mismatch_OFP=0.300392;Best_TOPMed_Mismatch_SVTYPE=INV;Best_TOPMed_Mismatch_AF=4e-06;Best_TOPMed_Mismatch_Het=1;Best_TOPMed_Mismatch_HomAlt=0;TOPMed_SV_Cov=0.400108;ThousG_Count=0;Best_ThousG_ID=HGSV_70074;Best_ThousG_OFP=0.0204879;Best_ThousG_AF=0.000312;Best_ThousG_Het=2;Best_ThousG_HomAlt=0;Best_ThousG_PopMax_AF=0.00112;Best_ThousG_Mismatch_ID=HGSV_70075;Best_ThousG_Mismatch_OFP=0.148371;Best_ThousG_Mismatch_SVTYPE=DUP;Best_ThousG_Mismatch_AF=0.000312;Best_ThousG_Mismatch_Het=2;Best_ThousG_Mismatch_HomAlt=0;ThousG_SV_Cov=0.0925492;gnomAD_Count=0;Best_gnomAD_ID=gnomAD-SV_v3_DEL_chr19_5ba69aa0;Best_gnomAD_OFP=0.172119;Best_gnomAD_AF=6.3e-05;Best_gnomAD_Het=8;Best_gnomAD_HomAlt=0;Best_gnomAD_PopMax_AF=0.000493;Best_gnomAD_Mismatch_ID=gnomAD-SV_v3_DUP_chr19_9114b2c5;Best_gnomAD_Mismatch_OFP=0.367091;Best_gnomAD_Mismatch_SVTYPE=DUP;Best_gnomAD_Mismatch_AF=8e-06;Best_gnomAD_Mismatch_Het=1;Best_gnomAD_Mismatch_HomAlt=0;gnomAD_SV_Cov=0.733638	GT:PSV:LN:DR:ST:QV:TY:ID:RAL:AAL:CO	0/1:NA:154087:26,11:+:1740:DEL:DEL00054230:NA:NA:chr19_4005348-chr19_4159435	0/1:NA:154090:0,0:+:999:DEL:MantaDEL_79083_0_1_0_0_0:NA:NA:chr19_4005350-chr19_4159440	0/1:NA:154090:0,0:+:650:DEL:10046:NA:NA:chr19_4005350-chr19_4159440	split	p13.3	CREB3L3				NM_001271996	2	4153630	4173054	5810	457	33	yes	10	txStart-intron3	5'UTR-CDS	223	3'	4153630	4159440								dbVar:nssv18787865	19:4156976-4157315															3	Hypertriglyceridemia_2										IMH	19:1692676-41507766	0.0103																										82.61	-0.25549	-1.46872	-1.15338	0.144667	-0.01045	hypertriglyceridemia;hypertriglyceridemia 1;hypertriglyceridemia 2	AD	Limited;Strong	21666694;24503134;26427795	84699	611998	Hypertriglyceridemia 2, 619324 (3) AD	AD	yes		7	8.06E-14	1.59E-09	0						full=5	0/1
19_4005230_4159441_DUP_1	19	4005230	4159441	154171	DUP	ILS_AP001,ILS_AP001_1,ILS_AP001_2	MantaDUP:TANDEM:79083:0:1:1:0:0	T		999	PASS	SUPP=3;SUPP_VEC=111;SVLEN=154171;SVTYPE=DUP;SVMETHOD=SURVIVOR1.0.7;CHR2=19;END=4159423;CIPOS=-22,1;CIEND=0,18;STRANDS=-;Max_AF=0;Max_Het=0;Max_HomAlt=0;Max_PopMax_AF=0;CCDG_Count=0;Best_CCDG_ID=490213;Best_CCDG_OFP=0.150896;Best_CCDG_AF=3.419e-05;Best_CCDG_Het=0;Best_CCDG_HomAlt=0;Best_CCDG_PopMax_AF=0;Best_CCDG_Mismatch_ID=253950;Best_CCDG_Mismatch_OFP=0.113801;Best_CCDG_Mismatch_SVTYPE=DEL;Best_CCDG_Mismatch_AF=6.839e-05;Best_CCDG_Mismatch_Het=0;Best_CCDG_Mismatch_HomAlt=0;SV_Cov=0.97591;CCDG_SV_Cov=0.202644;TOPMed_Count=0;Best_TOPMed_ID=DUP_19:4084740-4226078;Best_TOPMed_OFP=0.255974;Best_TOPMed_AF=4e-06;Best_TOPMed_Het=1;Best_TOPMed_HomAlt=0;Best_TOPMed_PopMax_AF=6.35898e-06;Best_TOPMed_Mismatch_ID=INV_19:3851264-4364229;Best_TOPMed_Mismatch_OFP=0.30055;Best_TOPMed_Mismatch_SVTYPE=INV;Best_TOPMed_Mismatch_AF=4e-06;Best_TOPMed_Mismatch_Het=1;Best_TOPMed_Mismatch_HomAlt=0;TOPMed_SV_Cov=0.741834;ThousG_Count=0;Best_ThousG_ID=HGSV_70075;Best_ThousG_OFP=0.14813;Best_ThousG_AF=0.000312;Best_ThousG_Het=2;Best_ThousG_HomAlt=0;Best_ThousG_PopMax_AF=0.002041;Best_ThousG_Mismatch_ID=HGSV_70074;Best_ThousG_Mismatch_OFP=0.0204771;Best_ThousG_Mismatch_SVTYPE=DEL;Best_ThousG_Mismatch_AF=0.000312;Best_ThousG_Mismatch_Het=2;Best_ThousG_Mismatch_HomAlt=0;ThousG_SV_Cov=0.285162;gnomAD_Count=0;Best_gnomAD_ID=gnomAD-SV_v3_DUP_chr19_9114b2c5;Best_gnomAD_OFP=0.36671;Best_gnomAD_AF=8e-06;Best_gnomAD_Het=1;Best_gnomAD_HomAlt=0;Best_gnomAD_PopMax_AF=7.9e-05;Best_gnomAD_Mismatch_ID=gnomAD-SV_v3_DEL_chr19_5ba69aa0;Best_gnomAD_Mismatch_OFP=0.172029;Best_gnomAD_Mismatch_SVTYPE=DEL;Best_gnomAD_Mismatch_AF=6.3e-05;Best_gnomAD_Mismatch_Het=8;Best_gnomAD_Mismatch_HomAlt=0;gnomAD_SV_Cov=0.960771	GT:PSV:LN:DR:ST:QV:TY:ID:RAL:AAL:CO	0/1:NA:154170:21,8:-:600:DUP:DUP00054229:NA:NA:chr19_4005253-chr19_4159423	0/1:NA:154171:0,0:-:999:DUP:MantaDUP_TANDEM_79083_0_1_1_0_0:NA:NA:chr19_4005252-chr19_4159423	0/1:NA:154211:0,0:-:106:DUP:10045:NA:NA:chr19_4005230-chr19_4159441	split	p13.3	CREB3L3				NM_001271996	2	4153630	4173054	5811	457	33	yes	10	txStart-intron3	5'UTR-CDS	222	3'	4153630	4159441								dbVar:nssv18787865	19:4156976-4157315															3	Hypertriglyceridemia_2										IMH	19:1692676-41507766	0.0103																										82.61	-0.25549	-1.46872	-1.15338	0.144667	-0.01045	hypertriglyceridemia;hypertriglyceridemia 1;hypertriglyceridemia 2	AD	Limited;Strong	21666694;24503134;26427795	84699	611998	Hypertriglyceridemia 2, 619324 (3) AD	AD	yes		7	8.06E-14	1.59E-09	0						full=3	0/1
10_66739832_66748834_DEL_1	10	66739832	66748834	-9001	DEL	ILS_AP001,ILS_AP001_1,ILS_AP001_2	MantaDEL:41772:0:1:0:0:0	A		900	PASS	SUPP=3;SUPP_VEC=111;SVLEN=-9001;SVTYPE=DEL;SVMETHOD=SURVIVOR1.0.7;CHR2=10;END=66748833;CIPOS=0,4;CIEND=0,1;STRANDS=+;Max_AF=0;Max_Het=0;Max_HomAlt=0;Max_PopMax_AF=0;CCDG_Count=0;Best_CCDG_ID=151651;Best_CCDG_OFP=0.152246;Best_CCDG_AF=3.419e-05;Best_CCDG_Het=0;Best_CCDG_HomAlt=0;Best_CCDG_PopMax_AF=0;Best_CCDG_Mismatch_ID=550410;Best_CCDG_Mismatch_OFP=0.00023818;Best_CCDG_Mismatch_SVTYPE=INV;Best_CCDG_Mismatch_AF=3.419e-05;Best_CCDG_Mismatch_Het=0;Best_CCDG_Mismatch_HomAlt=0;SV_Cov=1;CCDG_SV_Cov=1;TOPMed_Count=0;Best_TOPMed_ID=DEL_10:66743635-66747408;Best_TOPMed_OFP=0.419351;Best_TOPMed_AF=4e-06;Best_TOPMed_Het=1;Best_TOPMed_HomAlt=0;Best_TOPMed_PopMax_AF=0.000108861;Best_TOPMed_Mismatch_ID=INV_10:66737797-66745382;Best_TOPMed_Mismatch_OFP=0.451163;Best_TOPMed_Mismatch_SVTYPE=INV;Best_TOPMed_Mismatch_AF=4e-06;Best_TOPMed_Mismatch_Het=1;Best_TOPMed_Mismatch_HomAlt=0;TOPMed_SV_Cov=1;ThousG_Count=0;Best_ThousG_ID=HGSV_17158;Best_ThousG_OFP=0.141815;Best_ThousG_AF=0.000156;Best_ThousG_Het=1;Best_ThousG_HomAlt=0;Best_ThousG_PopMax_AF=0.000832;ThousG_SV_Cov=1;gnomAD_Count=0;Best_gnomAD_ID=gnomAD-SV_v3_DEL_chr10_ae68f6a5;Best_gnomAD_OFP=0.573931;Best_gnomAD_AF=8e-06;Best_gnomAD_Het=1;Best_gnomAD_HomAlt=0;Best_gnomAD_PopMax_AF=1.7e-05;Best_gnomAD_Mismatch_ID=gnomAD-SV_v3_DUP_chr10_bb431596;Best_gnomAD_Mismatch_OFP=0.113538;Best_gnomAD_Mismatch_SVTYPE=DUP;Best_gnomAD_Mismatch_AF=8e-06;Best_gnomAD_Mismatch_Het=1;Best_gnomAD_Mismatch_HomAlt=0;gnomAD_SV_Cov=1	GT:PSV:LN:DR:ST:QV:TY:ID:RAL:AAL:CO	0/1:NA:9002:19,11:+:900:DEL:DEL00032380:NA:NA:chr10_66739832-chr10_66748834	0/1:NA:9001:0,0:+:408:DEL:MantaDEL_41772_0_1_0_0_0:NA:NA:chr10_66739832-chr10_66748833	0/1:NA:8997:0,0:+:389:DEL:6102:NA:NA:chr10_66739836-chr10_66748833	split	q21.3	CTNNA3				XM_017016157	3	65912522	66838602	9003	0	0	no	12	intron3-intron3	CDS	17429	5'	66739831	66748834																													dbVar;gnomAD-SV_v3_DEL_chr10_a13546d2;gnomAD-SV_v3_DEL_chr10_e433cf8f;gnomAD-SV_v3_DEL_chr10_f70efaa2	chr10:37996824-76847693;chr10:53764413-94620402;chr10:59459006-109812369;chr10:66401619-66756961;chr10:66507333-66750567;chr10:66558659-66899003;chr10:66627986-66764657;chr10:66653518-66833431;chr10:66689895-66832247;chr10:66691061-66754539	0.3941																														0	0	2.58	-2.62354	-1.83693	-2.47163	-0.81711	-2.50548	arrhythmogenic right ventricular cardiomyopathy;arrhythmogenic right ventricular dysplasia 13	AD	Limited	21254927;23136403;28202948;28416588;30847666;32880476	29119	607667	Arrhythmogenic right ventricular dysplasia 13, 615616 (3) AD	AD	yes		4	1.02E-12	6.54E-08	0						full=1	0/1
1_46934266_46938413_DEL_1	1	46934266	46938413	-4146	DEL	ILS_AP001,ILS_AP001_1,ILS_AP001_2	MantaDEL:192794:0:1:0:0:0	A		368	PASS	SUPP=3;SUPP_VEC=111;SVLEN=-4146;SVTYPE=DEL;SVMETHOD=SURVIVOR1.0.7;CHR2=1;END=46938412;CIPOS=0,4;CIEND=0,1;STRANDS=+;Max_AF=0;Max_Het=0;Max_HomAlt=0;Max_PopMax_AF=0;CCDG_Count=0;Best_CCDG_Mismatch_ID=456535;Best_CCDG_Mismatch_OFP=0.0351945;Best_CCDG_Mismatch_SVTYPE=DUP;Best_CCDG_Mismatch_AF=3.419e-05;Best_CCDG_Mismatch_Het=0;Best_CCDG_Mismatch_HomAlt=0;SV_Cov=1;CCDG_SV_Cov=0;TOPMed_Count=0;Best_TOPMed_Mismatch_ID=INV_1:46904533-46976957;Best_TOPMed_Mismatch_OFP=0.0572584;Best_TOPMed_Mismatch_SVTYPE=INV;Best_TOPMed_Mismatch_AF=1.4e-05;Best_TOPMed_Mismatch_Het=4;Best_TOPMed_Mismatch_HomAlt=0;TOPMed_SV_Cov=0;ThousG_Count=0;Best_ThousG_Mismatch_ID=HGSV_3133;Best_ThousG_Mismatch_OFP=0.0351945;Best_ThousG_Mismatch_SVTYPE=DUP;Best_ThousG_Mismatch_AF=0.000312;Best_ThousG_Mismatch_Het=0;Best_ThousG_Mismatch_HomAlt=1;ThousG_SV_Cov=0;gnomAD_Count=0;Best_gnomAD_ID=gnomAD-SV_v3_DEL_chr1_c9a27eb5;Best_gnomAD_OFP=0.0505233;Best_gnomAD_AF=8e-06;Best_gnomAD_Het=1;Best_gnomAD_HomAlt=0;Best_gnomAD_PopMax_AF=1.7e-05;Best_gnomAD_Mismatch_ID=gnomAD-SV_v3_DUP_chr1_536cca67;Best_gnomAD_Mismatch_OFP=0.0351945;Best_gnomAD_Mismatch_SVTYPE=DUP;Best_gnomAD_Mismatch_AF=8e-06;Best_gnomAD_Mismatch_Het=1;Best_gnomAD_Mismatch_HomAlt=0;gnomAD_SV_Cov=1	GT:PSV:LN:DR:ST:QV:TY:ID:RAL:AAL:CO	0/1:NA:4147:15,10:+:364:DEL:DEL00002018:NA:NA:chr1_46934266-chr1_46938413	0/1:NA:4146:0,0:+:333:DEL:MantaDEL_192794_0_1_0_0_0:NA:NA:chr1_46934266-chr1_46938412	0/1:NA:4142:0,0:+:368:DEL:402:NA:NA:chr1_46934270-chr1_46938412	split	p33	CYP4A11				NM_000778	4	46929187	46941476	4148	803	51	yes	12	intron1-exon8	CDS	90	5'	46934265	46938413																										dbVar	chr1:46851047-46968877;chr1:46897535-46950292;chr1:46906324-47107665	0.01	gnomAD-SV_v3_DEL_chr1_cf5f2df8	chr1:13203398-167621949	0.1676				IMH	1:7601938-50381771;1:10187873-186857210;1:16798094-234784887;1:31594523-94732184;1:36241392-73134343;1:41598779-54316740;1:46855517-47110532;1:46855922-47110120;1:46928737-47150547	0.4698																										80.74				1.525402	-1.04546					1579						8	1.09E-24	1.57E-15	0						full=3	0/1
7_158845413_158849948_DEL_1	7	1.59E+08	1.59E+08	-4534	DEL	ILS_AP001,ILS_AP001_1,ILS_AP001_2	MantaDEL:117616:0:1:0:0:0	A		840	PASS	SUPP=3;SUPP_VEC=111;SVLEN=-4534;SVTYPE=DEL;SVMETHOD=SURVIVOR1.0.7;CHR2=7;END=158849947;CIPOS=0,5;CIEND=0,1;STRANDS=+;Max_AF=0.000468;Max_Het=3;Max_HomAlt=0;Max_PopMax_AF=0.002496;CCDG_Count=0;Best_CCDG_Mismatch_ID=531469;Best_CCDG_Mismatch_OFP=0.0204096;Best_CCDG_Mismatch_SVTYPE=DUP;Best_CCDG_Mismatch_AF=6.839e-05;Best_CCDG_Mismatch_Het=0;Best_CCDG_Mismatch_HomAlt=0;SV_Cov=1;CCDG_SV_Cov=0;TOPMed_Count=0;Best_TOPMed_ID=DEL_7:158840783-158851228;Best_TOPMed_OFP=0.434096;Best_TOPMed_AF=4e-06;Best_TOPMed_Het=1;Best_TOPMed_HomAlt=0;Best_TOPMed_PopMax_AF=0.00010879;TOPMed_Mismatches=INV_7:158845422-158849948;TOPMed_Mismatches_Count=1;TOPMed_Mismatch_SVTYPEs=INV;Best_TOPMed_Mismatch_ID=INV_7:158845422-158849948;Best_TOPMed_Mismatch_OFP=0.998015;Best_TOPMed_Mismatch_SVTYPE=INV;Best_TOPMed_Mismatch_AF=3.9e-05;Best_TOPMed_Mismatch_Het=11;Best_TOPMed_Mismatch_HomAlt=0;TOPMed_SV_Cov=1;ThousG_Count=1;Best_ThousG_ID=HGSV_150660;Best_ThousG_OFP=0.998897;Best_ThousG_AF=0.000468;Best_ThousG_Het=3;Best_ThousG_HomAlt=0;Best_ThousG_PopMax_AF=0.002496;Best_ThousG_Mismatch_ID=HGSV_150654;Best_ThousG_Mismatch_OFP=0.00957005;Best_ThousG_Mismatch_SVTYPE=DUP;Best_ThousG_Mismatch_AF=0.000312;Best_ThousG_Mismatch_Het=2;Best_ThousG_Mismatch_HomAlt=0;ThousG_SV_Cov=0.998897;gnomAD_Count=1;Best_gnomAD_ID=gnomAD-SV_v3_DEL_chr7_be27f839;Best_gnomAD_OFP=0.998897;Best_gnomAD_AF=2.4e-05;Best_gnomAD_Het=3;Best_gnomAD_HomAlt=0;Best_gnomAD_PopMax_AF=0.000682;Best_gnomAD_Mismatch_ID=gnomAD-SV_v3_DUP_chr7_f232bfce;Best_gnomAD_Mismatch_OFP=0.0346691;Best_gnomAD_Mismatch_SVTYPE=DUP;Best_gnomAD_Mismatch_AF=8e-06;Best_gnomAD_Mismatch_Het=1;Best_gnomAD_Mismatch_HomAlt=0;gnomAD_SV_Cov=1	GT:PSV:LN:DR:ST:QV:TY:ID:RAL:AAL:CO	0/1:NA:4535:24,11:+:840:DEL:DEL00025162:NA:NA:chr7_158845413-chr7_158849948	0/1:NA:4534:0,0:+:527:DEL:MantaDEL_117616_0_1_0_0_0:NA:NA:chr7_158845413-chr7_158849947	0/1:NA:4529:0,0:+:401:DEL:4931:NA:NA:chr7_158845418-chr7_158849947	split	q36.3	DYNC2I1				NM_001350914	2	1.59E+08	1.59E+08	4536	0	0	no	25	intron1-intron1	5'UTR	5914	5'	1.59E+08	1.59E+08																										dbVar	chr7:158644451-159005913;chr7:158779308-159164309;chr7:158804459-158851414;chr7:158827353-158934388	0.01	dbVar	chr7:158815335-158864408	0.01																														30								Jeune syndrome;short rib-polydactyly syndrome, Verma-Naumoff type;short-rib thoracic dysplasia 8 with or without polydactyly	AR	Strong;Supportive	23910462;23910462[PMID];26874042;28454995;29068549;29271569;9068549	55112	615462	Short-rib thoracic dysplasia 8 with or without polydactyly, 615503 (3) AR	AR						0.6436	Cleft lip;Cleft palate;Cleft upper lip;Micrognathia;Short rib-polydactyly syndrome, Verma-Naumoff type					full=3	0/1
7_158840077_158850185_INV_1	7	1.59E+08	1.59E+08	10108	INV	ILS_AP001,ILS_AP001_1,ILS_AP001_2	MantaBND:117616:2:3:0:0:0:1	A	A]chr7:158850185]	984	PASS	SUPP=3;SUPP_VEC=111;SVLEN=10108;SVTYPE=INV;SVMETHOD=SURVIVOR1.0.7;CHR2=7;END=158850185;CIPOS=0,4;CIEND=0,0;STRANDS=;Max_AF=3.9e-05;Max_Het=11;Max_HomAlt=0;Max_PopMax_AF=0.00120297;CCDG_Count=0;Best_CCDG_Mismatch_ID=531469;Best_CCDG_Mismatch_OFP=0.0454953;Best_CCDG_Mismatch_SVTYPE=DUP;Best_CCDG_Mismatch_AF=6.839e-05;Best_CCDG_Mismatch_Het=0;Best_CCDG_Mismatch_HomAlt=0;SV_Cov=1;CCDG_SV_Cov=0;TOPMed_Count=1;Best_TOPMed_ID=INV_7:158840078-158850185;Best_TOPMed_OFP=1;Best_TOPMed_AF=3.9e-05;Best_TOPMed_Het=11;Best_TOPMed_HomAlt=0;Best_TOPMed_PopMax_AF=0.00120297;TOPMed_Mismatches=DEL_7:158840783-158851228;TOPMed_Mismatches_Count=1;TOPMed_Mismatch_SVTYPEs=DEL;Best_TOPMed_Mismatch_ID=DEL_7:158840783-158851228;Best_TOPMed_Mismatch_OFP=0.837386;Best_TOPMed_Mismatch_SVTYPE=DEL;Best_TOPMed_Mismatch_AF=4e-06;Best_TOPMed_Mismatch_Het=1;Best_TOPMed_Mismatch_HomAlt=0;TOPMed_SV_Cov=1;ThousG_Count=0;Best_ThousG_Mismatch_ID=HGSV_150659;Best_ThousG_Mismatch_OFP=0.479591;Best_ThousG_Mismatch_SVTYPE=DEL;Best_ThousG_Mismatch_AF=0.000468;Best_ThousG_Mismatch_Het=3;Best_ThousG_Mismatch_HomAlt=0;ThousG_SV_Cov=0;gnomAD_Count=0;gnomAD_Mismatches=gnomAD-SV_v3_DEL_chr7_700d536c;gnomAD_Mismatches_Count=1;gnomAD_Mismatch_SVTYPEs=DEL;Best_gnomAD_Mismatch_ID=gnomAD-SV_v3_DEL_chr7_700d536c;Best_gnomAD_Mismatch_OFP=0.837706;Best_gnomAD_Mismatch_SVTYPE=DEL;Best_gnomAD_Mismatch_AF=8e-06;Best_gnomAD_Mismatch_Het=1;Best_gnomAD_Mismatch_HomAlt=0;gnomAD_SV_Cov=0	GT:PSV:LN:DR:ST:QV:TY:ID:RAL:AAL:CO	0/1:NA:10108:24,7::984:INV:INV00025160:NA:NA:chr7_158840077-chr7_158850185	0/1:NA:10108:0,0::585:INV:MantaBND_117616_2_3_0_0_0_1:NA:NA:chr7_158840077-chr7_158850185	0/1:NA:10104:0,0::317:INV:4932_1:NA:NA:chr7_158840081-chr7_158850185	split	q36.3	DYNC2I1				NM_001350914	2	1.59E+08	1.59E+08	10109	0	0	no	25	intron1-intron1	5'UTR	578	5'	1.59E+08	1.59E+08																										dbVar	chr7:158644451-159005913;chr7:158779308-159164309;chr7:158804459-158851414;chr7:158827353-158934388	0.01	dbVar	chr7:158815335-158864408	0.01																														30								Jeune syndrome;short rib-polydactyly syndrome, Verma-Naumoff type;short-rib thoracic dysplasia 8 with or without polydactyly	AR	Strong;Supportive	23910462;23910462[PMID];26874042;28454995;29068549;29271569;9068549	55112	615462	Short-rib thoracic dysplasia 8 with or without polydactyly, 615503 (3) AR	AR						0.6436	Cleft lip;Cleft palate;Cleft upper lip;Micrognathia;Short rib-polydactyly syndrome, Verma-Naumoff type					full=NA	0/1
10_92864682_92873535_DEL_1	10	92864682	92873535	-8852	DEL	ILS_AP001,ILS_AP001_1,ILS_AP001_2	MantaDEL:43772:0:1:0:0:0	G		1080	PASS	SUPP=3;SUPP_VEC=111;SVLEN=-8852;SVTYPE=DEL;SVMETHOD=SURVIVOR1.0.7;CHR2=10;END=92873534;CIPOS=0,5;CIEND=0,1;STRANDS=+;Max_AF=0.003122;Max_Het=133;Max_HomAlt=5;Max_PopMax_AF=0.0202;CCDG_Count=1;Best_CCDG_ID=154088;Best_CCDG_OFP=0.999435;Best_CCDG_AF=0.00106;Best_CCDG_Het=0;Best_CCDG_HomAlt=0;Best_CCDG_PopMax_AF=0;Best_CCDG_Mismatch_ID=550410;Best_CCDG_Mismatch_OFP=0.000234237;Best_CCDG_Mismatch_SVTYPE=INV;Best_CCDG_Mismatch_AF=3.419e-05;Best_CCDG_Mismatch_Het=0;Best_CCDG_Mismatch_HomAlt=0;SV_Cov=1;CCDG_SV_Cov=0.999435;TOPMed_Count=2;Best_TOPMed_ID=DEL_10:92864688-92873540;Best_TOPMed_OFP=0.998758;Best_TOPMed_AF=0.000507;Best_TOPMed_Het=133;Best_TOPMed_HomAlt=5;Best_TOPMed_PopMax_AF=0.0151186;Best_TOPMed_Mismatch_ID=DUP_10:92834902-92875100;Best_TOPMed_Mismatch_OFP=0.220224;Best_TOPMed_Mismatch_SVTYPE=DUP;Best_TOPMed_Mismatch_AF=4e-06;Best_TOPMed_Mismatch_Het=1;Best_TOPMed_Mismatch_HomAlt=0;TOPMed_SV_Cov=0.999435;ThousG_Count=1;Best_ThousG_ID=HGSV_18630;Best_ThousG_OFP=0.959779;Best_ThousG_AF=0.003122;Best_ThousG_Het=20;Best_ThousG_HomAlt=0;Best_ThousG_PopMax_AF=0.016639;Best_ThousG_Mismatch_ID=HGSV_18631;Best_ThousG_Mismatch_OFP=0.00587372;Best_ThousG_Mismatch_SVTYPE=INS;Best_ThousG_Mismatch_AF=0.001093;Best_ThousG_Mismatch_Het=7;Best_ThousG_Mismatch_HomAlt=0;ThousG_SV_Cov=1;gnomAD_Count=1;Best_gnomAD_ID=gnomAD-SV_v3_DEL_chr10_3841b527;Best_gnomAD_OFP=0.986737;Best_gnomAD_AF=0.000722;Best_gnomAD_Het=89;Best_gnomAD_HomAlt=1;Best_gnomAD_PopMax_AF=0.0202;Best_gnomAD_Mismatch_ID=gnomAD-SV_v3_DUP_chr10_3d69cad9;Best_gnomAD_Mismatch_OFP=0.092979;Best_gnomAD_Mismatch_SVTYPE=DUP;Best_gnomAD_Mismatch_AF=8e-06;Best_gnomAD_Mismatch_Het=1;Best_gnomAD_Mismatch_HomAlt=0;gnomAD_SV_Cov=1	GT:PSV:LN:DR:ST:QV:TY:ID:RAL:AAL:CO	0/1:NA:8853:17,9:+:1080:DEL:DEL00032978:NA:NA:chr10_92864682-chr10_92873535	0/1:NA:8852:0,0:+:485:DEL:MantaDEL_43772_0_1_0_0_0:NA:NA:chr10_92864682-chr10_92873534	0/1:NA:8847:0,0:+:392:DEL:6204:NA:NA:chr10_92864687-chr10_92873534	split	q23.33	EXOC6				XM_017016347	3	92872843	93059490	692	38	1	yes	22	txStart-intron1	5'UTR-CDS	223	5'	92872843	92873535																													1000g;dbVar;gnomAD-SV_v3_DEL_chr10_244783f4;gnomAD-SV_v3_DEL_chr10_e433cf8f;gnomAD-SV_v3_DEL_chr10_f048c376;gnomAD-SV_v3_DEL_chr10_f70efaa2	chr10:53764413-94620402;chr10:59459006-109812369;chr10:90655870-109812371;chr10:90661220-109812370;chr10:92864611-92873535;10:92864612-92873535	0.4959																																7.76	0.185137	0.111528	0.181862	-0.397	0.598347					54536						3	2.44E-07	2.82E-02	0						full=3	0/1
6_5606319_5609855_DEL_1	6	5606319	5609855	-3517	DEL	ILS_AP001,ILS_AP001_1,ILS_AP001_2	MantaDEL:39071:0:1:0:0:0	C		954	PASS	SUPP=3;SUPP_VEC=111;SVLEN=-3517;SVTYPE=DEL;SVMETHOD=SURVIVOR1.0.7;CHR2=6;END=5609855;CIPOS=-19,3;CIEND=-3,0;STRANDS=+;Max_AF=0.005464;Max_Het=170;Max_HomAlt=7;Max_PopMax_AF=0.029118;CCDG_Count=0;Best_CCDG_Mismatch_ID=369898_2;Best_CCDG_Mismatch_OFP=0.000284252;Best_CCDG_Mismatch_SVTYPE=BND;Best_CCDG_Mismatch_AF=0.001094;Best_CCDG_Mismatch_Het=0;Best_CCDG_Mismatch_HomAlt=0;SV_Cov=1;CCDG_SV_Cov=0;TOPMed_Count=2;Best_TOPMed_ID=DEL_6:5606321-5609856;Best_TOPMed_OFP=0.994628;Best_TOPMed_AF=0.000633;Best_TOPMed_Het=170;Best_TOPMed_HomAlt=4;Best_TOPMed_PopMax_AF=0.0172301;Best_TOPMed_Mismatch_ID=INV_6:5606188-5607137;Best_TOPMed_Mismatch_OFP=0.191295;Best_TOPMed_Mismatch_SVTYPE=INV;Best_TOPMed_Mismatch_AF=1.1e-05;Best_TOPMed_Mismatch_Het=3;Best_TOPMed_Mismatch_HomAlt=0;TOPMed_SV_Cov=1;ThousG_Count=1;Best_ThousG_ID=HGSV_130286;Best_ThousG_OFP=0.994628;Best_ThousG_AF=0.005464;Best_ThousG_Het=21;Best_ThousG_HomAlt=7;Best_ThousG_PopMax_AF=0.029118;ThousG_SV_Cov=1;gnomAD_Count=1;Best_gnomAD_ID=gnomAD-SV_v3_DEL_chr6_3bb97935;Best_gnomAD_OFP=0.994628;Best_gnomAD_AF=0.000864;Best_gnomAD_Het=101;Best_gnomAD_HomAlt=4;Best_gnomAD_PopMax_AF=0.021108;Best_gnomAD_Mismatch_ID=gnomAD-SV_v3_DUP_chr6_ce5e12be;Best_gnomAD_Mismatch_OFP=0.0118053;Best_gnomAD_Mismatch_SVTYPE=DUP;Best_gnomAD_Mismatch_AF=8e-06;Best_gnomAD_Mismatch_Het=1;Best_gnomAD_Mismatch_HomAlt=0;gnomAD_SV_Cov=1	GT:PSV:LN:DR:ST:QV:TY:ID:RAL:AAL:CO	0/1:NA:3511:18,10:+:954:DEL:DEL00018600:NA:NA:chr6_5606341-chr6_5609852	0/1:NA:3517:0,0:+:416:DEL:MantaDEL_39071_0_1_0_0_0:NA:NA:chr6_5606338-chr6_5609855	0/1:NA:3536:0,0:+:332:DEL:3665:NA:NA:chr6_5606319-chr6_5609855	split	p25.1	FARS2				XM_047418086	1	5249933	5771583	3537	0	0	no	7	intron5-intron5	CDS	3313	3'	5606318	5609855																										dbVar	chr6:5578125-5645911	0.01	dbVar	chr6:5562842-5641064;chr6:5606319-5609855	0.01																														30	0	44.7	-2.62354	-0.67047	-1.79433	-0.69919	0.096804	Leigh syndrome;combined oxidative phosphorylation defect type 14;hereditary spastic paraplegia 77	AR	Limited;Moderate;Strong;Supportive	22499341;22499341[PMID];22833457;25851414;26553276;26553276[PMID];27549011;27652284;29126765	10667	611592	Combined oxidative phosphorylation deficiency 14, 614946 (3) AR;Spastic paraplegia 77, AR, 617046 (3) AR	AR	yes		6	9.30E-09	4.54E-06	0.409	Autosomal recessive spastic paraplegia type 77;Cleft palate;Macrodontia of permanent maxillary central incisor;Micrognathia;Retrognathia					full=1	0/1
10_13652960_13653018_DEL_1	10	13652960	13653018	-57	DEL	ILS_AP001,ILS_AP001_1,ILS_AP001_2	MantaDEL:37558:0:0:0:0:0	ACAGAGGGCAGAGCTGGAATTCCAGCTCCCTCCAGCTGTCAGACTTTGGGCCAGGCAC	A	720	PASS	SUPP=3;SUPP_VEC=111;SVLEN=-57;SVTYPE=DEL;SVMETHOD=SURVIVOR1.0.7;CHR2=10;END=13653017;CIPOS=0,0;CIEND=0,1;STRANDS=+;Max_AF=0.00921;Max_Het=374;Max_HomAlt=11;Max_PopMax_AF=0.049085;CCDG_Count=0;Best_CCDG_Mismatch_ID=145531_1;Best_CCDG_Mismatch_OFP=0.0172414;Best_CCDG_Mismatch_SVTYPE=BND;Best_CCDG_Mismatch_AF=0.001368;Best_CCDG_Mismatch_Het=0;Best_CCDG_Mismatch_HomAlt=0;SV_Cov=1;CCDG_SV_Cov=0;TOPMed_Count=1;Best_TOPMed_ID=DEL_10:13652961-13653017;Best_TOPMed_OFP=1;Best_TOPMed_AF=0.001071;Best_TOPMed_Het=257;Best_TOPMed_HomAlt=6;Best_TOPMed_PopMax_AF=0.0312274;Best_TOPMed_Mismatch_ID=DUP_10:13580086-13654763;Best_TOPMed_Mismatch_OFP=0.000776657;Best_TOPMed_Mismatch_SVTYPE=DUP;Best_TOPMed_Mismatch_AF=1.4e-05;Best_TOPMed_Mismatch_Het=4;Best_TOPMed_Mismatch_HomAlt=0;TOPMed_SV_Cov=1;ThousG_Count=1;Best_ThousG_ID=HGSV_14079;Best_ThousG_OFP=1;Best_ThousG_AF=0.00921;Best_ThousG_Het=39;Best_ThousG_HomAlt=10;Best_ThousG_PopMax_AF=0.049085;ThousG_SV_Cov=1;gnomAD_Count=1;Best_gnomAD_ID=gnomAD-SV_v3_DEL_chr10_2288408b;Best_gnomAD_OFP=0.865672;Best_gnomAD_AF=0.003142;Best_gnomAD_Het=374;Best_gnomAD_HomAlt=11;Best_gnomAD_PopMax_AF=0.034968;Best_gnomAD_Mismatch_ID=gnomAD-SV_v3_DUP_chr10_f8fe9e87;Best_gnomAD_Mismatch_OFP=0.000776657;Best_gnomAD_Mismatch_SVTYPE=DUP;Best_gnomAD_Mismatch_AF=8e-06;Best_gnomAD_Mismatch_Het=1;Best_gnomAD_Mismatch_HomAlt=0;gnomAD_SV_Cov=1	GT:PSV:LN:DR:ST:QV:TY:ID:RAL:AAL:CO	0/1:NA:58:0,0:+:720:DEL:DEL00030300:NA:NA:chr10_13652960-chr10_13653018	0/1:NA:57:0,0:+:660:DEL:MantaDEL_37558_0_0_0_0_0:ACAGAGGGCAGAGCTGGAATTCCAGCTCCCTCCAGCTGTCAGACTTTGGGCCAGGCAC:A:chr10_13652960-chr10_13653017	0/1:NA:57:0,0:+:293:DEL:5923:NA:NA:chr10_13652960-chr10_13653017	split	p13	FRMD4A				NM_001318338	2	13643705	13707567	59	0	0	no	14	intron12-intron12	CDS	985	3'	13652959	13653018																										dbVar	chr10:13543727-13697859;chr10:13580085-13654763	0.01																																			21.52	0.79038	-0.06336	0.267812	-0.26748	3.274066	severe intellectual disability-corpus callosum agenesis-facial dysmorphism-cerebellar ataxia syndrome	AR	Limited;Strong;Supportive	25388005;25388005[PMID];30214071;34869127	55691	616305	?Corpus callosum, agenesis of, with facial anomalies and cerebellar ataxia, 616819 (3) AR	AR		yes	0	1.0000e	1.00E+00	0.4303	Cleft lip;Severe intellectual disability-corpus callosum agenesis-facial dysmorphism-cerebellar ataxia syndrome;Thick lower lip vermilion	Cleft lip;Cleft palate;Hypertelorism;Micrognathia;Microphthalmia;abnormal retina blood vessel morphology				full=3	0/1
14_66758452_66758602_DEL_1	14	66758452	66758602	-149	DEL	ILS_AP001,ILS_AP001_1,ILS_AP001_2	MantaDEL:75072:0:1:0:0:0	TCAGAGGCTTGTCTAAGGATCCCCAGTAAAAGGGAGCCATCGTTTGAAGCTACAGTTGCGTGACCATTTTGAGTTTGATGGCCTGAAGGCGAGAAGAGAGAAACTGGGTTATTAGAAGACATGTATCAAAATGAAAAAAGGGGGTAAGGA	T	840	PASS	SUPP=3;SUPP_VEC=111;SVLEN=-149;SVTYPE=DEL;SVMETHOD=SURVIVOR1.0.7;CHR2=14;END=66758601;CIPOS=0,3;CIEND=0,1;STRANDS=+;Max_AF=0.000624;Max_Het=27;Max_HomAlt=0;Max_PopMax_AF=0.00411;CCDG_Count=1;Best_CCDG_ID=206074;Best_CCDG_OFP=0.98;Best_CCDG_AF=0.0003419;Best_CCDG_Het=0;Best_CCDG_HomAlt=0;Best_CCDG_PopMax_AF=0;Best_CCDG_Mismatch_ID=562107;Best_CCDG_Mismatch_OFP=4.203e-06;Best_CCDG_Mismatch_SVTYPE=INV;Best_CCDG_Mismatch_AF=3.419e-05;Best_CCDG_Mismatch_Het=0;Best_CCDG_Mismatch_HomAlt=0;SV_Cov=1;CCDG_SV_Cov=1;TOPMed_Count=0;Best_TOPMed_ID=DEL_14:66756635-66760354;Best_TOPMed_OFP=0.0403117;Best_TOPMed_AF=8.9e-05;Best_TOPMed_Het=25;Best_TOPMed_HomAlt=0;Best_TOPMed_PopMax_AF=0.000319966;Best_TOPMed_Mismatch_ID=DUP_14:66721655-66768709;Best_TOPMed_Mismatch_OFP=0.00318769;Best_TOPMed_Mismatch_SVTYPE=DUP;Best_TOPMed_Mismatch_AF=4e-06;Best_TOPMed_Mismatch_Het=1;Best_TOPMed_Mismatch_HomAlt=0;TOPMed_SV_Cov=1;ThousG_Count=1;Best_ThousG_ID=HGSV_46304;Best_ThousG_OFP=0.98;Best_ThousG_AF=0.000624;Best_ThousG_Het=4;Best_ThousG_HomAlt=0;Best_ThousG_PopMax_AF=0.003328;ThousG_SV_Cov=0.98;gnomAD_Count=1;Best_gnomAD_ID=gnomAD-SV_v3_DEL_chr14_21c34aba;Best_gnomAD_OFP=0.98;Best_gnomAD_AF=0.000215;Best_gnomAD_Het=27;Best_gnomAD_HomAlt=0;Best_gnomAD_PopMax_AF=0.00411;Best_gnomAD_Mismatch_ID=gnomAD-SV_v3_INV_chr14_53e4a3ab;Best_gnomAD_Mismatch_OFP=3.31211e-05;Best_gnomAD_Mismatch_SVTYPE=INV;Best_gnomAD_Mismatch_AF=2.4e-05;Best_gnomAD_Mismatch_Het=3;Best_gnomAD_Mismatch_HomAlt=0;gnomAD_SV_Cov=1	GT:PSV:LN:DR:ST:QV:TY:ID:RAL:AAL:CO	0/1:NA:150:0,0:+:840:DEL:DEL00042430:NA:NA:chr14_66758452-chr14_66758602	0/1:NA:149:0,0:+:585:DEL:MantaDEL_75072_0_1_0_0_0:TCAGAGGCTTGTCTAAGGATCCCCAGTAAAAGGGAGCCATCGTTTGAAGCTACAGTTGCGTGACCATTTTGAGTTTGATGGCCTGAAGGCGAGAAGAGAGAAACTGGGTTATTAGAAGACATGTATCAAAATGAAAAAAGGGGGTAAGGA:T:chr14_66758452-chr14_66758601	0/1:NA:146:0,0:+:353:DEL:8086:NA:NA:chr14_66758455-chr14_66758601	split	q23.3	GPHN				NM_001024218	2	66508146	67181803	151	0	0	no	22	intron2-intron2	CDS	17861	3'	66758451	66758602																													gnomAD-SV_v3_DEL_chr14_040215df;gnomAD-SV_v3_DEL_chr14_aa7bf92b	chr14:41140072-69277618;chr14:56471274-99420950	0.633				IMH	14:23629193-97505158	0.4022																								2	0	2.69	0.037021	-0.55458	-0.32602	0.35526	3.981971	hereditary hyperekplexia;sulfite oxidase deficiency due to molybdenum cofactor deficiency type C	AD;AR	Limited;Moderate;Strong;Supportive	11095995;12684523;20301437[PMID];22040219;9812897	10243	603930	Molybdenum cofactor deficiency C, 615501 (3) AR	AR	yes		0	1.00E+00	1.00E+00	0.4913		Cleft lip;Cleft palate;Holoprosencephaly;Hypertelorism;Micrognathia;Microphthalmia;abnormal motor neuron morphology;abnormal retina inner plexiform layer morphology				full=3	0/1
3_7562320_7562538_DEL_1	3	7562320	7562538	-217	DEL	ILS_AP001,ILS_AP001_1,ILS_AP001_2	MantaDEL:157633:0:1:0:0:0	GTGAAGTAATAGTTCACTTTCCCCCTTATATTTTTTTAAAAAGAAAAAAAAAATACTAGTTTTAATTGACTATTACCCCTCTAAAATACTCTCAAATATAGAGTCATATTTGGTTTTTCTGTAACTCCATTAATTGACCTCACAGAAGTTAAGTGAGTCGATCAAGATGATCTAGCCAGAATTGAGAGCGTTTGCAACCCAGTTCATGTTCTATAAGT	G	900	PASS	SUPP=3;SUPP_VEC=111;SVLEN=-217;SVTYPE=DEL;SVMETHOD=SURVIVOR1.0.7;CHR2=3;END=7562537;CIPOS=0,1;CIEND=0,1;STRANDS=+;Max_AF=0.000312;Max_Het=24;Max_HomAlt=0;Max_PopMax_AF=0.003191;CCDG_Count=1;Best_CCDG_ID=311214;Best_CCDG_OFP=0.995413;Best_CCDG_AF=0.0002393;Best_CCDG_Het=0;Best_CCDG_HomAlt=0;Best_CCDG_PopMax_AF=0;Best_CCDG_Mismatch_ID=505309;Best_CCDG_Mismatch_OFP=0.00318746;Best_CCDG_Mismatch_SVTYPE=DUP;Best_CCDG_Mismatch_AF=3.419e-05;Best_CCDG_Mismatch_Het=0;Best_CCDG_Mismatch_HomAlt=0;SV_Cov=1;CCDG_SV_Cov=0.995413;TOPMed_Count=0;Best_TOPMed_Mismatch_ID=DUP_3:7509306-7783201;Best_TOPMed_Mismatch_OFP=0.00079592;Best_TOPMed_Mismatch_SVTYPE=DUP;Best_TOPMed_Mismatch_AF=4e-06;Best_TOPMed_Mismatch_Het=1;Best_TOPMed_Mismatch_HomAlt=0;TOPMed_SV_Cov=0;ThousG_Count=1;Best_ThousG_ID=HGSV_97735;Best_ThousG_OFP=0.995413;Best_ThousG_AF=0.000312;Best_ThousG_Het=2;Best_ThousG_HomAlt=0;Best_ThousG_PopMax_AF=0.001664;Best_ThousG_Mismatch_ID=HGSV_97728;Best_ThousG_Mismatch_OFP=0.000861827;Best_ThousG_Mismatch_SVTYPE=DUP;Best_ThousG_Mismatch_AF=0.000312;Best_ThousG_Mismatch_Het=2;Best_ThousG_Mismatch_HomAlt=0;ThousG_SV_Cov=0.995413;gnomAD_Count=1;Best_gnomAD_ID=gnomAD-SV_v3_DEL_chr3_6696a17a;Best_gnomAD_OFP=0.889908;Best_gnomAD_AF=0.000191;Best_gnomAD_Het=24;Best_gnomAD_HomAlt=0;Best_gnomAD_PopMax_AF=0.003191;Best_gnomAD_Mismatch_ID=gnomAD-SV_v3_DUP_chr3_bfde11c2;Best_gnomAD_Mismatch_OFP=0.000433586;Best_gnomAD_Mismatch_SVTYPE=DUP;Best_gnomAD_Mismatch_AF=8e-06;Best_gnomAD_Mismatch_Het=1;Best_gnomAD_Mismatch_HomAlt=0;gnomAD_SV_Cov=1	GT:PSV:LN:DR:ST:QV:TY:ID:RAL:AAL:CO	0/1:NA:218:0,0:+:900:DEL:DEL00009225:NA:NA:chr3_7562320-chr3_7562538	0/1:NA:217:0,0:+:574:DEL:MantaDEL_157633_0_1_0_0_0:GTGAAGTAATAGTTCACTTTCCCCCTTATATTTTTTTAAAAAGAAAAAAAAAATACTAGTTTTAATTGACTATTACCCCTCTAAAATACTCTCAAATATAGAGTCATATTTGGTTTTTCTGTAACTCCATTAATTGACCTCACAGAAGTTAAGTGAGTCGATCAAGATGATCTAGCCAGAATTGAGAGCGTTTGCAACCCAGTTCATGTTCTATAAGT:G:chr3_7562320-chr3_7562537	0/1:NA:216:0,0:+:407:DEL:1877:NA:NA:chr3_7562321-chr3_7562537	split	p26.1	GRM7				XM_047448053	1	6861114	7680295	219	0	0	no	9	intron7-intron7	CDS	15883	3'	7562319	7562538																										dbVar	chr3:7413097-7915880;chr3:7507741-7629043;chr3:7509066-7752803	0.01	dbVar	chr3:2977315-7774313	0.01																																8.74				0.251415	3.075868	neurodevelopmental disorder with seizures, hypotonia, and brain imaging abnormalities	AR	Moderate;Strong	27435318;28097321;32286009	2917	604101	Neurodevelopmental disorder with seizures, hypotonia, and brain abnormalities, 618922 (3) AR	AR	yes		0	9.99E-01	9.99E-01	0.5649	Cleft lip;Cleft palate;Early infantile epileptic encephalopathy;Micrognathia;Short finger					full=3	0/1
11_113935087_113935461_DEL_1	11	1.14E+08	1.14E+08	-373	DEL	ILS_AP001,ILS_AP001_1,ILS_AP001_2	MantaDEL:34525:0:0:0:0:0	TATTATTTGTAGTTACATGTCACTTTCCTTCACCCTAAATGTTAATTCGTCAACAGCAGCTGAAGGTTTTCTATATACCATCTCCATTTTAATTATGATGAACAGAGACAGAAGATTGTCTAATGGAGGGGAGGCTTCCCTGGATCTCAGAGCCATGGTCCAGTTCCTCTGCTCTCCTCACTGCCATGCTTCCCCCCGGGTACGTTTTGAAGTGGAAGTGTGCTGGAGGCAGGTGCAATTTGATTTCATGCTCTTGGAACTTTTCCATGGGTCCTTTTGCCCCGGGGGTGGACGTGGGTTATCTAGCAGGGCTGGCCTCTGCCCTGGTCCTCAGGAGCAGAGTAGGGAGAGAATCCTGAGTAGAGTCACAGATC	T	611	PASS	SUPP=3;SUPP_VEC=111;SVLEN=-373;SVTYPE=DEL;SVMETHOD=SURVIVOR1.0.7;CHR2=11;END=113935460;CIPOS=0,2;CIEND=0,1;STRANDS=+;Max_AF=0.0028;Max_Het=614;Max_HomAlt=0;Max_PopMax_AF=0.00483092;CCDG_Count=0;Best_CCDG_Mismatch_ID=468820;Best_CCDG_Mismatch_OFP=0.563571;Best_CCDG_Mismatch_SVTYPE=DUP;Best_CCDG_Mismatch_AF=0.1058;Best_CCDG_Mismatch_Het=0;Best_CCDG_Mismatch_HomAlt=0;SV_Cov=1;CCDG_SV_Cov=0;TOPMed_Count=1;Best_TOPMed_ID=DEL_11:113935090-113935460;Best_TOPMed_OFP=0.994652;Best_TOPMed_AF=0.0028;Best_TOPMed_Het=614;Best_TOPMed_HomAlt=0;Best_TOPMed_PopMax_AF=0.00483092;Best_TOPMed_Mismatch_ID=INV_11:107936486-114400629;Best_TOPMed_Mismatch_OFP=5.78576e-05;Best_TOPMed_Mismatch_SVTYPE=INV;Best_TOPMed_Mismatch_AF=4e-06;Best_TOPMed_Mismatch_Het=1;Best_TOPMed_Mismatch_HomAlt=0;TOPMed_SV_Cov=0.994652;ThousG_Count=0;Best_ThousG_ID=HGSV_27620;Best_ThousG_OFP=4.5051e-05;Best_ThousG_AF=0.000156;Best_ThousG_Het=1;Best_ThousG_HomAlt=0;Best_ThousG_PopMax_AF=0.000789;Best_ThousG_Mismatch_ID=HGSV_28027;Best_ThousG_Mismatch_OFP=0.563571;Best_ThousG_Mismatch_SVTYPE=DUP;Best_ThousG_Mismatch_AF=0.102124;Best_ThousG_Mismatch_Het=336;Best_ThousG_Mismatch_HomAlt=159;ThousG_SV_Cov=1;gnomAD_Count=0;Best_gnomAD_ID=gnomAD-SV_v3_DEL_chr11_15837620;Best_gnomAD_OFP=1.9578e-05;Best_gnomAD_AF=8e-06;Best_gnomAD_Het=1;Best_gnomAD_HomAlt=0;Best_gnomAD_PopMax_AF=0.000248;Best_gnomAD_Mismatch_ID=gnomAD-SV_v3_DUP_chr11_8701e58a;Best_gnomAD_Mismatch_OFP=8.59435e-05;Best_gnomAD_Mismatch_SVTYPE=DUP;Best_gnomAD_Mismatch_AF=0.066172;Best_gnomAD_Mismatch_Het=7511;Best_gnomAD_Mismatch_HomAlt=169;gnomAD_SV_Cov=1	GT:PSV:LN:DR:ST:QV:TY:ID:RAL:AAL:CO	0/1:NA:374:0,0:+:540:DEL:DEL00036329:NA:NA:chr11_113935087-chr11_113935461	0/1:NA:373:0,0:+:611:DEL:MantaDEL_34525_0_0_0_0_0:TATTATTTGTAGTTACATGTCACTTTCCTTCACCCTAAATGTTAATTCGTCAACAGCAGCTGAAGGTTTTCTATATACCATCTCCATTTTAATTATGATGAACAGAGACAGAAGATTGTCTAATGGAGGGGAGGCTTCCCTGGATCTCAGAGCCATGGTCCAGTTCCTCTGCTCTCCTCACTGCCATGCTTCCCCCCGGGTACGTTTTGAAGTGGAAGTGTGCTGGAGGCAGGTGCAATTTGATTTCATGCTCTTGGAACTTTTCCATGGGTCCTTTTGCCCCGGGGGTGGACGTGGGTTATCTAGCAGGGCTGGCCTCTGCCCTGGTCCTCAGGAGCAGAGTAGGGAGAGAATCCTGAGTAGAGTCACAGATC:T:chr11_113935087-chr11_113935460	0/1:NA:371:0,0:+:401:DEL:6861:NA:NA:chr11_113935089-chr11_113935460	split	q23.2	HTR3B				XM_024448767	2	1.14E+08	1.14E+08	375	0	0	no	9	intron6-intron6	CDS	1993	5'	1.14E+08	1.14E+08																																																															57.7	-0.74871	0.590207	0.068695	0.432545	-0.26425					9177						5	2.48E-07	6.15E-07	0						full=3	0/1
1_228180857_228185000_DEL_1	1	2.28E+08	2.28E+08	-4142	DEL	ILS_AP001,ILS_AP001_1,ILS_AP001_2	MantaDEL:203946:0:1:0:0:0	C		1214	PASS	SUPP=3;SUPP_VEC=111;SVLEN=-4142;SVTYPE=DEL;SVMETHOD=SURVIVOR1.0.7;CHR2=1;END=228184999;CIPOS=0,3;CIEND=0,1;STRANDS=+;Max_AF=0.000781;Max_Het=129;Max_HomAlt=1;Max_PopMax_AF=0.00534468;CCDG_Count=0;Best_CCDG_Mismatch_ID=141197_1;Best_CCDG_Mismatch_OFP=0.000241371;Best_CCDG_Mismatch_SVTYPE=BND;Best_CCDG_Mismatch_AF=0.007283;Best_CCDG_Mismatch_Het=0;Best_CCDG_Mismatch_HomAlt=0;SV_Cov=1;CCDG_SV_Cov=0;TOPMed_Count=1;Best_TOPMed_ID=DEL_1:228180861-228185003;Best_TOPMed_OFP=0.998311;Best_TOPMed_AF=0.000467;Best_TOPMed_Het=129;Best_TOPMed_HomAlt=1;Best_TOPMed_PopMax_AF=0.00534468;Best_TOPMed_Mismatch_ID=DUP_1:228142774-228211680;Best_TOPMed_Mismatch_OFP=0.0601236;Best_TOPMed_Mismatch_SVTYPE=DUP;Best_TOPMed_Mismatch_AF=4e-06;Best_TOPMed_Mismatch_Het=1;Best_TOPMed_Mismatch_HomAlt=0;TOPMed_SV_Cov=1;ThousG_Count=1;Best_ThousG_ID=HGSV_11644;Best_ThousG_OFP=0.998073;Best_ThousG_AF=0.000781;Best_ThousG_Het=5;Best_ThousG_HomAlt=0;Best_ThousG_PopMax_AF=0.003328;ThousG_SV_Cov=1;gnomAD_Count=1;Best_gnomAD_ID=gnomAD-SV_v3_DEL_chr1_491b7c9a;Best_gnomAD_OFP=0.976662;Best_gnomAD_AF=0.000365;Best_gnomAD_Het=44;Best_gnomAD_HomAlt=1;Best_gnomAD_PopMax_AF=0.003631;Best_gnomAD_Mismatch_ID=gnomAD-SV_v3_DUP_chr1_4733695d;Best_gnomAD_Mismatch_OFP=0.0240871;Best_gnomAD_Mismatch_SVTYPE=DUP;Best_gnomAD_Mismatch_AF=8e-06;Best_gnomAD_Mismatch_Het=1;Best_gnomAD_Mismatch_HomAlt=0;gnomAD_SV_Cov=1	GT:PSV:LN:DR:ST:QV:TY:ID:RAL:AAL:CO	0/1:NA:4143:16,11:+:1214:DEL:DEL00004747:NA:NA:chr1_228180857-chr1_228185000	0/1:NA:4142:0,0:+:375:DEL:MantaDEL_203946_0_1_0_0_0:NA:NA:chr1_228180857-chr1_228184999	0/1:NA:4139:0,0:+:596:DEL:959:NA:NA:chr1_228180860-chr1_228184999	split	q42.13	IBA57				NM_001010867	4	2.28E+08	2.28E+08	1401	0	0	no	3	exon3-txEnd	3'UTR	5735	3'	2.28E+08	2.28E+08																													dbVar	chr1:228180819-228184966;chr1:228180831-228184999	0.01				IMH	1:16798094-234784887;1:107555343-232824950;1:208408863-234126540	0.3106																										80.91	0.234203	0.61881	0.672301	0.547812	0.716851	hereditary spastic paraplegia 74;multiple mitochondrial dysfunctions syndrome 3	AR	Limited;Strong;Supportive	23462291;23462291[PMID];25609768;25609768[PMID];2569768;25971455;27785568;28671726	200205	615316	?Spastic paraplegia 74, AR, 616451 (3) AR;Multiple mitochondrial dysfunctions syndrome 3, 615330 (3) AR	AR	yes	yes	5	8.39E-03	8.30E-02	0.4683	Cleft palate;High palate;Micrognathia;Multiple mitochondrial dysfunctions syndrome 3;Retrognathia					full=3	0/1
X_105056165_105057819_DUP_1	X	1.05E+08	1.05E+08	1651	DUP	ILS_AP001,ILS_AP001_1,ILS_AP001_2	MantaDUP:TANDEM:5078:0:1:0:0:0	A		1680	PASS	SUPP=3;SUPP_VEC=111;SVLEN=1651;SVTYPE=DUP;SVMETHOD=SURVIVOR1.0.7;CHR2=X;END=105057816;CIPOS=0,1;CIEND=0,3;STRANDS=-;Max_AF=0.000218878;Max_Het=17;Max_HomAlt=2;Max_PopMax_AF=0.00763914;CCDG_Count=0;Best_CCDG_Mismatch_ID=449704_1;Best_CCDG_Mismatch_OFP=0.000605327;Best_CCDG_Mismatch_SVTYPE=BND;Best_CCDG_Mismatch_AF=0.0003077;Best_CCDG_Mismatch_Het=0;Best_CCDG_Mismatch_HomAlt=0;SV_Cov=1;CCDG_SV_Cov=0;TOPMed_Count=0;Best_TOPMed_ID=DUP_23:105010354-105059267;Best_TOPMed_OFP=0.0337729;Best_TOPMed_AF=9e-06;Best_TOPMed_Het=1;Best_TOPMed_HomAlt=0;Best_TOPMed_PopMax_AF=1.64107e-05;Best_TOPMed_Mismatch_ID=DEL_23:105051214-105069228;Best_TOPMed_Mismatch_OFP=0.0916963;Best_TOPMed_Mismatch_SVTYPE=DEL;Best_TOPMed_Mismatch_AF=5e-06;Best_TOPMed_Mismatch_Het=1;Best_TOPMed_Mismatch_HomAlt=0;TOPMed_SV_Cov=1;ThousG_Count=0;Best_ThousG_Mismatch_ID=HGSV_170862;Best_ThousG_Mismatch_OFP=0.000605327;Best_ThousG_Mismatch_SVTYPE=INS;Best_ThousG_Mismatch_AF=0.0025;Best_ThousG_Mismatch_Het=0;Best_ThousG_Mismatch_HomAlt=0;ThousG_SV_Cov=0;gnomAD_Count=1;Best_gnomAD_ID=gnomAD-SV_v3_DUP_chrX_d04d6449;Best_gnomAD_OFP=0.998187;Best_gnomAD_AF=0.000218878;Best_gnomAD_Het=17;Best_gnomAD_HomAlt=2;Best_gnomAD_PopMax_AF=0.00763914;Best_gnomAD_Mismatch_ID=gnomAD-SV_v3_DEL_chrX_b5294bd1;Best_gnomAD_Mismatch_OFP=0.618644;Best_gnomAD_Mismatch_SVTYPE=DEL;Best_gnomAD_Mismatch_AF=0.000145946;Best_gnomAD_Mismatch_Het=13;Best_gnomAD_Mismatch_HomAlt=1;gnomAD_SV_Cov=1	GT:PSV:LN:DR:ST:QV:TY:ID:RAL:AAL:CO	0/1:NA:1650:7,20:-:1680:DUP:DUP00063615:NA:NA:chrX_105056166-chrX_105057816	0/1:NA:1651:0,0:-:999:DUP:MantaDUP_TANDEM_5078_0_1_0_0_0:NA:NA:chrX_105056165-chrX_105057816	1/1:NA:1654:0,0:-:534:DUP:11517:NA:NA:chrX_105056165-chrX_105057819	split	q22.3	IL1RAPL2				NM_017416	2	1.05E+08	1.06E+08	1655	0	0	no	11	intron2-intron2	CDS	137655	3'	1.05E+08	1.05E+08																										dbVar	chrX:105035005-105106372	0.01	gnomAD-SV_v3_DEL_chrX_25f0d36f;gnomAD-SV_v3_DEL_chrX_a8ba77a6	chrX:85568529-142769920;chrX:101187696-118298467	0.4905				IMH	X:101070962-130555575;X:101432821-114762040	0.59																										4.12				0.303567	1.985591					26280						1	9.84E-01	9.73E-01	0						full=3	1/1
X_105056492_105057815_DEL_1	X	1.05E+08	1.05E+08	-1323	DEL	ILS_AP001,ILS_AP001_1,ILS_AP001_2	MantaDEL:5078:0:1:1:0:0	T		742	PASS	SUPP=3;SUPP_VEC=111;SVLEN=-1323;SVTYPE=DEL;SVMETHOD=SURVIVOR1.0.7;CHR2=X;END=105057815;CIPOS=0,0;CIEND=-8,0;STRANDS=+;Max_AF=0;Max_Het=0;Max_HomAlt=0;Max_PopMax_AF=0;CCDG_Count=0;Best_CCDG_Mismatch_ID=449704_2;Best_CCDG_Mismatch_OFP=0.000755287;Best_CCDG_Mismatch_SVTYPE=BND;Best_CCDG_Mismatch_AF=0.0003077;Best_CCDG_Mismatch_Het=0;Best_CCDG_Mismatch_HomAlt=0;SV_Cov=1;CCDG_SV_Cov=0;TOPMed_Count=0;Best_TOPMed_ID=DEL_23:105051214-105069228;Best_TOPMed_OFP=0.0734902;Best_TOPMed_AF=5e-06;Best_TOPMed_Het=1;Best_TOPMed_HomAlt=0;Best_TOPMed_PopMax_AF=0.000139373;Best_TOPMed_Mismatch_ID=DUP_23:105010354-105059267;Best_TOPMed_Mismatch_OFP=0.0270674;Best_TOPMed_Mismatch_SVTYPE=DUP;Best_TOPMed_Mismatch_AF=9e-06;Best_TOPMed_Mismatch_Het=1;Best_TOPMed_Mismatch_HomAlt=0;TOPMed_SV_Cov=1;ThousG_Count=0;Best_ThousG_Mismatch_ID=HGSV_170862;Best_ThousG_Mismatch_OFP=0.000755287;Best_ThousG_Mismatch_SVTYPE=INS;Best_ThousG_Mismatch_AF=0.0025;Best_ThousG_Mismatch_Het=0;Best_ThousG_Mismatch_HomAlt=0;ThousG_SV_Cov=0;gnomAD_Count=0;Best_gnomAD_ID=gnomAD-SV_v3_DEL_chrX_b5294bd1;Best_gnomAD_OFP=0.771903;Best_gnomAD_AF=0.000145946;Best_gnomAD_Het=13;Best_gnomAD_HomAlt=1;Best_gnomAD_PopMax_AF=0.00511135;gnomAD_Mismatches=gnomAD-SV_v3_DUP_chrX_d04d6449;gnomAD_Mismatches_Count=1;gnomAD_Mismatch_SVTYPEs=DUP;Best_gnomAD_Mismatch_ID=gnomAD-SV_v3_DUP_chrX_d04d6449;Best_gnomAD_Mismatch_OFP=0.8;Best_gnomAD_Mismatch_SVTYPE=DUP;Best_gnomAD_Mismatch_AF=0.000218878;Best_gnomAD_Mismatch_Het=17;Best_gnomAD_Mismatch_HomAlt=2;gnomAD_SV_Cov=1	GT:PSV:LN:DR:ST:QV:TY:ID:RAL:AAL:CO	0/1:NA:1315:6,10:+:742:DEL:DEL00063616:NA:NA:chrX_105056492-chrX_105057807	0/1:NA:1323:0,0:+:734:DEL:MantaDEL_5078_0_1_1_0_0:NA:NA:chrX_105056492-chrX_105057815	0/1:NA:1323:0,0:+:488:DEL:11518:NA:NA:chrX_105056492-chrX_105057815	split	q22.3	IL1RAPL2				NM_017416	2	1.05E+08	1.06E+08	1324	0	0	no	11	intron2-intron2	CDS	137659	3'	1.05E+08	1.05E+08																										dbVar	chrX:105035005-105106372	0.01	gnomAD-SV_v3_DEL_chrX_25f0d36f;gnomAD-SV_v3_DEL_chrX_a8ba77a6	chrX:85568529-142769920;chrX:101187696-118298467	0.4905				IMH	X:101070962-130555575;X:101432821-114762040	0.59																										4.12				0.303567	1.985591					26280						1	9.84E-01	9.73E-01	0						full=3	0/1
12_118350_118652_DEL_1	12	118350	118652	-301	DEL	ILS_AP001,ILS_AP001_1,ILS_AP001_2	MantaDEL:14387:0:1:0:0:0	TTCTGCCCGTCTGAGATCACCATATTGATTCAAAGAAAGGAGGGAAAAGAAGTGTTTTAGAAAGGGCTAAAAATCTTGAGGGGACGTGACGCATGTTGTTAGCTTTTGTCTAGAAAATGCTTCATAGCTTTGTCCCAGAAGAGCTTTAAGAATGGCTGGCAGAGTCTGGGATAATCTAGTGCAATTGGCCAGGCCCTCTGGAGGAGTCTTCATACCCAAGGAGTCCATCAGCCTTCAAAATGCTCCTGGTAGAGTCTCTAGTAATTACTGTGCTAGCCATTGCCGAGATAGGAGAACAGCCC	T	540	PASS	SUPP=3;SUPP_VEC=111;SVLEN=-301;SVTYPE=DEL;SVMETHOD=SURVIVOR1.0.7;CHR2=12;END=118651;CIPOS=0,2;CIEND=0,1;STRANDS=+;Max_AF=0.006244;Max_Het=28;Max_HomAlt=6;Max_PopMax_AF=0.02579;CCDG_Count=0;Best_CCDG_Mismatch_ID=174164_1;Best_CCDG_Mismatch_OFP=0.00331126;Best_CCDG_Mismatch_SVTYPE=BND;Best_CCDG_Mismatch_AF=0.004035;Best_CCDG_Mismatch_Het=0;Best_CCDG_Mismatch_HomAlt=0;SV_Cov=1;CCDG_SV_Cov=0;TOPMed_Count=0;Best_TOPMed_ID=DEL_12:105734-121528;Best_TOPMed_OFP=0.0191188;Best_TOPMed_AF=7e-06;Best_TOPMed_Het=2;Best_TOPMed_HomAlt=0;Best_TOPMed_PopMax_AF=1.25214e-05;Best_TOPMed_Mismatch_ID=INV_12:109574-130409;Best_TOPMed_Mismatch_OFP=0.0144934;Best_TOPMed_Mismatch_SVTYPE=INV;Best_TOPMed_Mismatch_AF=7e-06;Best_TOPMed_Mismatch_Het=2;Best_TOPMed_Mismatch_HomAlt=0;TOPMed_SV_Cov=1;ThousG_Count=1;Best_ThousG_ID=HGSV_29233;Best_ThousG_OFP=0.993378;Best_ThousG_AF=0.006244;Best_ThousG_Het=28;Best_ThousG_HomAlt=6;Best_ThousG_PopMax_AF=0.02579;Best_ThousG_Mismatch_ID=HGSV_29232;Best_ThousG_Mismatch_OFP=0.00221494;Best_ThousG_Mismatch_SVTYPE=DUP;Best_ThousG_Mismatch_AF=0.001093;Best_ThousG_Mismatch_Het=7;Best_ThousG_Mismatch_HomAlt=0;ThousG_SV_Cov=1;gnomAD_Count=0;Best_gnomAD_ID=gnomAD-SV_v3_DEL_chr12_ca8579db;Best_gnomAD_OFP=0.464615;Best_gnomAD_AF=0.002994;Best_gnomAD_Het=376;Best_gnomAD_HomAlt=0;Best_gnomAD_PopMax_AF=0.0108;Best_gnomAD_Mismatch_ID=gnomAD-SV_v3_DUP_chr12_d0d84cd3;Best_gnomAD_Mismatch_OFP=0.00578821;Best_gnomAD_Mismatch_SVTYPE=DUP;Best_gnomAD_Mismatch_AF=1.6e-05;Best_gnomAD_Mismatch_Het=2;Best_gnomAD_Mismatch_HomAlt=0;gnomAD_SV_Cov=1	GT:PSV:LN:DR:ST:QV:TY:ID:RAL:AAL:CO	0/1:NA:302:0,0:+:540:DEL:DEL00036695:NA:NA:chr12_118350-chr12_118652	0/1:NA:301:0,0:+:328:DEL:MantaDEL_14387_0_1_0_0_0:TTCTGCCCGTCTGAGATCACCATATTGATTCAAAGAAAGGAGGGAAAAGAAGTGTTTTAGAAAGGGCTAAAAATCTTGAGGGGACGTGACGCATGTTGTTAGCTTTTGTCTAGAAAATGCTTCATAGCTTTGTCCCAGAAGAGCTTTAAGAATGGCTGGCAGAGTCTGGGATAATCTAGTGCAATTGGCCAGGCCCTCTGGAGGAGTCTTCATACCCAAGGAGTCCATCAGCCTTCAAAATGCTCCTGGTAGAGTCTCTAGTAATTACTGTGCTAGCCATTGCCGAGATAGGAGAACAGCCC:T:chr12_118350-chr12_118651	0/1:NA:299:0,0:+:326:DEL:6921:NA:NA:chr12_118352-chr12_118651	split	p13.33	IQSEC3				XM_047428865	1	66766	171330	303	0	0	no	13	intron2-intron2	CDS	6980	3'	118349	118652																										dbVar	chr12:45740-204934;chr12:72834-118834;chr12:114421-232803;chr12:115648-162429	0.01	dbVar	chr12:115648-162429	0.01																																56.71				2.479006	3.288811					440073						2	3.87E-03	9.25E-01	0.4858		Cleft lip;Cleft palate;Hypertelorism;Micrognathia;Microphthalmia;decreased total retina thickness				full=1	0/1
7_31547158_31552382_INV_1	7	31547158	31552382	5224	INV	ILS_AP001,ILS_AP001_1,ILS_AP001_2	MantaBND:107320:0:2:0:0:0:1	T	[chr7:31552382[T	1860	PASS	SUPP=3;SUPP_VEC=111;SVLEN=5224;SVTYPE=INV;SVMETHOD=SURVIVOR1.0.7;CHR2=7;END=31552382;CIPOS=0,0;CIEND=0,0;STRANDS=;Max_AF=0;Max_Het=0;Max_HomAlt=0;Max_PopMax_AF=0;CCDG_Count=0;Best_CCDG_ID=599604;Best_CCDG_OFP=0.627368;Best_CCDG_AF=0.1509;Best_CCDG_Het=0;Best_CCDG_HomAlt=0;Best_CCDG_PopMax_AF=0;Best_CCDG_Mismatch_ID=393327;Best_CCDG_Mismatch_OFP=0.323062;Best_CCDG_Mismatch_SVTYPE=DEL;Best_CCDG_Mismatch_AF=0.1649;Best_CCDG_Mismatch_Het=0;Best_CCDG_Mismatch_HomAlt=0;SV_Cov=1;CCDG_SV_Cov=1;TOPMed_Count=0;Best_TOPMed_ID=INV_7:31547416-31550692;Best_TOPMed_OFP=0.627368;Best_TOPMed_AF=0.164089;Best_TOPMed_Het=35249;Best_TOPMed_HomAlt=4452;Best_TOPMed_PopMax_AF=0.318713;Best_TOPMed_Mismatch_ID=DEL_7:31539302-31551300;Best_TOPMed_Mismatch_OFP=0.273755;Best_TOPMed_Mismatch_SVTYPE=DEL;Best_TOPMed_Mismatch_AF=0.000166;Best_TOPMed_Mismatch_Het=43;Best_TOPMed_Mismatch_HomAlt=0;TOPMed_SV_Cov=1;ThousG_Count=0;Best_ThousG_ID=HGSV_142363;Best_ThousG_OFP=0.00333769;Best_ThousG_AF=0.000156;Best_ThousG_Het=1;Best_ThousG_HomAlt=0;Best_ThousG_PopMax_AF=0.00056;ThousG_Mismatches=HGSV_142371;ThousG_Mismatches_Count=1;ThousG_Mismatch_SVTYPEs=CPX;Best_ThousG_Mismatch_ID=HGSV_142371;Best_ThousG_Mismatch_OFP=0.999617;Best_ThousG_Mismatch_SVTYPE=CPX;Best_ThousG_Mismatch_AF=0.175773;Best_ThousG_Mismatch_Het=856;Best_ThousG_Mismatch_HomAlt=135;ThousG_SV_Cov=1;gnomAD_Count=0;Best_gnomAD_ID=gnomAD-SV_v3_INV_chr7_30ab2e39;Best_gnomAD_OFP=0.00333769;Best_gnomAD_AF=1.6e-05;Best_gnomAD_Het=0;Best_gnomAD_HomAlt=1;Best_gnomAD_PopMax_AF=5.9e-05;Best_gnomAD_Mismatch_ID=gnomAD-SV_v3_DEL_chr7_eb25a3d2;Best_gnomAD_Mismatch_OFP=0.522166;Best_gnomAD_Mismatch_SVTYPE=DEL;Best_gnomAD_Mismatch_AF=8e-06;Best_gnomAD_Mismatch_Het=1;Best_gnomAD_Mismatch_HomAlt=0;gnomAD_SV_Cov=1	GT:PSV:LN:DR:ST:QV:TY:ID:RAL:AAL:CO	0/1:NA:5224:18,14:--:1860:INV:INV00022053:NA:NA:chr7_31547158-chr7_31552382	0/1:NA:5224:0,0::851:INV:MantaBND_107320_0_2_0_0_0_1:NA:NA:chr7_31547158-chr7_31552382	0/1:NA:5224:0,0:--:707:INV:4351_1:NA:NA:chr7_31547158-chr7_31552382	split	p14.3	ITPRID1				NM_001257967	3	31514089	31656504	5225	0	0	no	15	intron1-intron2	5'UTR	619	3'	31547157	31552382																													gnomAD-SV_v3_DEL_chr7_f5bb684d	chr7:26936102-36716780	0.0533				1000g;IMH	7:23851402-66061756;7:29684534-32729958;7:31547152-31552405	0.391																																				223075									0						full=NA	0/1
6_36476339_36476763_DEL_1	6	36476339	36476763	-423	DEL	ILS_AP001,ILS_AP001_1,ILS_AP001_2	MantaDEL:121876:0:1:0:0:0	TGATCACACTAATTTATTGAATTCTAGGCTAAATAGAATCCAGCCATTCAAATATATGTGTATTTGAACATATAAATTCACAGTGAACCATTTTTTTTTTTTTTTTTTTAAGACAGAGTCTCACTCTGTCACCAAGGCTGGAGGGCAGTGGTGCAATCTCGGCTCACTGCAACCTCCACCTCCCGGGTTCAAGCAATTCTCGTGCCTCAGCCTCCCAAGTAGCTGGGATCACAGGTGTGTGCCACCACACCCGGCAAATTTTTGTATTTTTAGTAGAGATGGGGTTTCACCACGTTGGTCAGGCTGGTCTTGAACTCCTGACCTCGTGATCCGCCCACCTTAGCCTCCCAAAGTGCTGGGATTACAGGCATGAGCCACTGTGCCTGGCCACAGCGAACCACTTTTTAATTAGAACCAAACCTGT	TTTTAA	600	PASS	SUPP=3;SUPP_VEC=111;SVLEN=-423;SVTYPE=DEL;SVMETHOD=SURVIVOR1.0.7;CHR2=6;END=36476762;CIPOS=0,7;CIEND=0,1;STRANDS=+;Max_AF=0.000175;Max_Het=20;Max_HomAlt=1;Max_PopMax_AF=0.001923;CCDG_Count=0;Best_CCDG_ID=374925;Best_CCDG_OFP=0.0373634;Best_CCDG_AF=3.419e-05;Best_CCDG_Het=0;Best_CCDG_HomAlt=0;Best_CCDG_PopMax_AF=0;Best_CCDG_Mismatch_ID=595637;Best_CCDG_Mismatch_OFP=0.119041;Best_CCDG_Mismatch_SVTYPE=INV;Best_CCDG_Mismatch_AF=3.419e-05;Best_CCDG_Mismatch_Het=0;Best_CCDG_Mismatch_HomAlt=0;SV_Cov=1;CCDG_SV_Cov=1;TOPMed_Count=0;Best_TOPMed_ID=DEL_6:36474415-36478946;Best_TOPMed_OFP=0.0935363;Best_TOPMed_AF=4e-06;Best_TOPMed_Het=1;Best_TOPMed_HomAlt=0;Best_TOPMed_PopMax_AF=6.26088e-06;Best_TOPMed_Mismatch_ID=DUP_6:36465002-36492500;Best_TOPMed_Mismatch_OFP=0.0154182;Best_TOPMed_Mismatch_SVTYPE=DUP;Best_TOPMed_Mismatch_AF=0.000155;Best_TOPMed_Mismatch_Het=41;Best_TOPMed_Mismatch_HomAlt=0;TOPMed_SV_Cov=1;ThousG_Count=0;ThousG_SV_Cov=0;gnomAD_Count=1;Best_gnomAD_ID=gnomAD-SV_v3_DEL_chr6_035dfea3;Best_gnomAD_OFP=1;Best_gnomAD_AF=0.000175;Best_gnomAD_Het=20;Best_gnomAD_HomAlt=1;Best_gnomAD_PopMax_AF=0.001923;Best_gnomAD_Mismatch_ID=gnomAD-SV_v3_CPX_chr6_cd4299ce;Best_gnomAD_Mismatch_OFP=0.400378;Best_gnomAD_Mismatch_SVTYPE=CPX;Best_gnomAD_Mismatch_AF=8e-06;Best_gnomAD_Mismatch_Het=1;Best_gnomAD_Mismatch_HomAlt=0;gnomAD_SV_Cov=1	GT:PSV:LN:DR:ST:QV:TY:ID:RAL:AAL:CO	0/1:NA:417:15,2:+:600:DEL:DEL00019390:NA:NA:chr6_36476346-chr6_36476763	0/1:NA:423:0,0:+:275:DEL:MantaDEL_121876_0_1_0_0_0:TGATCACACTAATTTATTGAATTCTAGGCTAAATAGAATCCAGCCATTCAAATATATGTGTATTTGAACATATAAATTCACAGTGAACCATTTTTTTTTTTTTTTTTTTAAGACAGAGTCTCACTCTGTCACCAAGGCTGGAGGGCAGTGGTGCAATCTCGGCTCACTGCAACCTCCACCTCCCGGGTTCAAGCAATTCTCGTGCCTCAGCCTCCCAAGTAGCTGGGATCACAGGTGTGTGCCACCACACCCGGCAAATTTTTGTATTTTTAGTAGAGATGGGGTTTCACCACGTTGGTCAGGCTGGTCTTGAACTCCTGACCTCGTGATCCGCCCACCTTAGCCTCCCAAAGTGCTGGGATTACAGGCATGAGCCACTGTGCCTGGCCACAGCGAACCACTTTTTAATTAGAACCAAACCTGT:TTTTAA:chr6_36476339-chr6_36476762	0/1:NA:423:0,0:+:299:DEL:3823:NA:NA:chr6_36476339-chr6_36476762	split	p21.31	KCTD20				XM_047418378	1	36442999	36484824	425	0	0	no	7	intron3-intron3	CDS	1276	5'	36476338	36476763																													dgv1761e212	6:36476140-36486595	0.01																																42.66	0.013891	1.195528	0.837954	0.449161	1.083038					222658						4	9.43E-04	3.99E-01	0						full=1	0/1
6_61568977_61569054_DEL_1	6	61568977	61569054	-76	DEL	ILS_AP001,ILS_AP001_1,ILS_AP001_2	MantaDEL:123588:0:0:0:0:0	AGTGGGCATATGGGGAAGTGTGAGGTCCAAAAGCCATGCTTGCTTTCTTGGAAGGGAAGCTCATGGCATGGGGCAGG	A	999	PASS	SUPP=3;SUPP_VEC=111;SVLEN=-76;SVTYPE=DEL;SVMETHOD=SURVIVOR1.0.7;CHR2=6;END=61569053;CIPOS=0,2;CIEND=0,1;STRANDS=+;Max_AF=0;Max_Het=0;Max_HomAlt=0;Max_PopMax_AF=0;CCDG_Count=0;Best_CCDG_Mismatch_ID=377597_2;Best_CCDG_Mismatch_OFP=0.012987;Best_CCDG_Mismatch_SVTYPE=BND;Best_CCDG_Mismatch_AF=0.5879;Best_CCDG_Mismatch_Het=0;Best_CCDG_Mismatch_HomAlt=0;SV_Cov=1;CCDG_SV_Cov=0;TOPMed_Count=0;TOPMed_SV_Cov=0;ThousG_Count=0;Best_ThousG_Mismatch_ID=HGSV_132575;Best_ThousG_Mismatch_OFP=2.5552e-06;Best_ThousG_Mismatch_SVTYPE=CPX;Best_ThousG_Mismatch_AF=0.000156;Best_ThousG_Mismatch_Het=1;Best_ThousG_Mismatch_HomAlt=0;ThousG_SV_Cov=0;gnomAD_Count=0;Best_gnomAD_ID=gnomAD-SV_v3_DEL_chr6_3ece45c4;Best_gnomAD_OFP=4.3123e-06;Best_gnomAD_AF=8e-06;Best_gnomAD_Het=1;Best_gnomAD_HomAlt=0;Best_gnomAD_PopMax_AF=3e-05;Best_gnomAD_Mismatch_ID=gnomAD-SV_v3_CPX_chr6_63da49e2;Best_gnomAD_Mismatch_OFP=1.55211e-06;Best_gnomAD_Mismatch_SVTYPE=CPX;Best_gnomAD_Mismatch_AF=2.4e-05;Best_gnomAD_Mismatch_Het=3;Best_gnomAD_Mismatch_HomAlt=0;gnomAD_SV_Cov=1	GT:PSV:LN:DR:ST:QV:TY:ID:RAL:AAL:CO	1/1:NA:77:0,0:+:780:DEL:DEL00019859:NA:NA:chr6_61568977-chr6_61569054	1/1:NA:76:0,0:+:999:DEL:MantaDEL_123588_0_0_0_0_0:AGTGGGCATATGGGGAAGTGTGAGGTCCAAAAGCCATGCTTGCTTTCTTGGAAGGGAAGCTCATGGCATGGGGCAGG:A:chr6_61568977-chr6_61569053	1/1:NA:74:0,0:+:649:DEL:3909:NA:NA:chr6_61568979-chr6_61569053	split	q11.1	KHDRBS2				NR_146870	2	61542669	62286225	78	0	0	no	16	intron14-intron14	UTR	22533	3'	61568976	61569054																										dbVar	chr6:61468685-62211095;chr6:61539594-61702095;chr6:61539985-61604664	0.01							IMH	6:38040936-64295998;6:47589331-73036081	0.2088																										11.27				-2.14059	-0.63655					202559						3	3.85E-02	1.43E-01	0.3426		Holoprosencephaly;tau protein deposits				full=3	1/1
3_160519499_160519819_DEL_1	3	1.61E+08	1.61E+08	-319	DEL	ILS_AP001,ILS_AP001_1,ILS_AP001_2	MantaDEL:167979:0:1:0:0:0	TAAAAAAAATAGGCGGCCGGGCGCGGTGGCTCACGCCTGTAATCCCAGCACTTTGGGAGGCTGAGGCGGGTGGATCATGAGGTCAGGAGATCGAGACCATCCTGGCTAACAAGGTGAAACCCCGTCTCTACTAAAAAATACAAAAAATTAGCCGGGCGCGGTGGTGGGCGCCTGTAGTCCCAGCTACTGGGGAGGCTGAGGCAGGAGAATGGCGTGAACCCGGGAAGCGGAGCTTGCAGTGAGCCGAGATTGTGCCACTGCAGTCCGCAGTCCGGCCTGGGCGACAGAGCGAGACTCCGTCTCAAAAAAAAAAAAAAAAA	T	720	PASS	SUPP=3;SUPP_VEC=111;SVLEN=-319;SVTYPE=DEL;SVMETHOD=SURVIVOR1.0.7;CHR2=3;END=160519818;CIPOS=0,13;CIEND=0,1;STRANDS=+;Max_AF=0.002822;Max_Het=353;Max_HomAlt=1;Max_PopMax_AF=0.005784;CCDG_Count=0;CCDG_Mismatches=327029;CCDG_Mismatches_Count=1;CCDG_Mismatch_SVTYPEs=MEI;Best_CCDG_Mismatch_ID=327029;Best_CCDG_Mismatch_OFP=0.959375;Best_CCDG_Mismatch_SVTYPE=MEI;Best_CCDG_Mismatch_AF=0.0003761;Best_CCDG_Mismatch_Het=0;Best_CCDG_Mismatch_HomAlt=0;SV_Cov=1;CCDG_SV_Cov=0;TOPMed_Count=0;Best_TOPMed_Mismatch_ID=DUP_3:160478802-160535100;Best_TOPMed_Mismatch_OFP=0.00568384;Best_TOPMed_Mismatch_SVTYPE=DUP;Best_TOPMed_Mismatch_AF=3.6e-05;Best_TOPMed_Mismatch_Het=10;Best_TOPMed_Mismatch_HomAlt=0;TOPMed_SV_Cov=0;ThousG_Count=1;Best_ThousG_ID=HGSV_105866;Best_ThousG_OFP=0.959375;Best_ThousG_AF=0.000781;Best_ThousG_Het=5;Best_ThousG_HomAlt=0;Best_ThousG_PopMax_AF=0.00416;ThousG_SV_Cov=0.959375;gnomAD_Count=1;Best_gnomAD_ID=gnomAD-SV_v3_DEL_chr3_e32466a0;Best_gnomAD_OFP=0.895185;Best_gnomAD_AF=0.002822;Best_gnomAD_Het=353;Best_gnomAD_HomAlt=1;Best_gnomAD_PopMax_AF=0.005784;Best_gnomAD_Mismatch_ID=gnomAD-SV_v3_DUP_chr3_0ec68f91;Best_gnomAD_Mismatch_OFP=0.015436;Best_gnomAD_Mismatch_SVTYPE=DUP;Best_gnomAD_Mismatch_AF=8.7e-05;Best_gnomAD_Mismatch_Het=9;Best_gnomAD_Mismatch_HomAlt=1;gnomAD_SV_Cov=1	GT:PSV:LN:DR:ST:QV:TY:ID:RAL:AAL:CO	0/1:NA:320:0,0:+:720:DEL:DEL00011547:NA:NA:chr3_160519499-chr3_160519819	0/1:NA:319:0,0:+:539:DEL:MantaDEL_167979_0_1_0_0_0:TAAAAAAAATAGGCGGCCGGGCGCGGTGGCTCACGCCTGTAATCCCAGCACTTTGGGAGGCTGAGGCGGGTGGATCATGAGGTCAGGAGATCGAGACCATCCTGGCTAACAAGGTGAAACCCCGTCTCTACTAAAAAATACAAAAAATTAGCCGGGCGCGGTGGTGGGCGCCTGTAGTCCCAGCTACTGGGGAGGCTGAGGCAGGAGAATGGCGTGAACCCGGGAAGCGGAGCTTGCAGTGAGCCGAGATTGTGCCACTGCAGTCCGCAGTCCGGCCTGGGCGACAGAGCGAGACTCCGTCTCAAAAAAAAAAAAAAAAA:T:chr3_160519499-chr3_160519818	0/1:NA:306:0,0:+:509:DEL:2379:NA:NA:chr3_160519512-chr3_160519818	split	q25.33	KPNA4				NM_002268	5	1.6E+08	1.61E+08	321	0	0	no	17	intron11-intron11	CDS	1959	5'	1.61E+08	1.61E+08																																			IMH	3:72502419-189537676;3:152031051-176727053;3:152031060-176727190	0.1058																										12.34	1.049593	0.011461	0.436278	0.176904	2.94291					3840						0	1.00E+00	9.98E-01	0						full=3	0/1
22_33237876_33238156_DEL_1	22	33237876	33238156	-279	DEL	ILS_AP001,ILS_AP001_1,ILS_AP001_2	MantaDEL:94356:0:1:0:0:0	AAATAAATGTGCATAACGCACGGGAGTACAGTATGGTAAAGGAAAGGGAGTGGAGGCTGACATCACATTCAGAGGCTGTATTTTAAGATGAAAGGTAACATATATGTAGTTTGTCTCAAAGTTATATCTTGAGACCAGGATGGGGACGAGGAAAGGGGACAGAAGAAAAAGGTTTCAAATGCAGTTTGAGGCTCAGCTGCTAAGCTGCTGGTTACAATAATAATAAATTGGAAACAACAACCAAAAAAGCTGTTTAGAGTTTTTAAAGGAGCACAAATAT	A	900	PASS	SUPP=3;SUPP_VEC=111;SVLEN=-279;SVTYPE=DEL;SVMETHOD=SURVIVOR1.0.7;CHR2=22;END=33238155;CIPOS=0,2;CIEND=0,1;STRANDS=+;Max_AF=0;Max_Het=0;Max_HomAlt=0;Max_PopMax_AF=0;CCDG_Count=0;Best_CCDG_ID=306905;Best_CCDG_OFP=0.00124581;Best_CCDG_AF=3.419e-05;Best_CCDG_Het=0;Best_CCDG_HomAlt=0;Best_CCDG_PopMax_AF=0;SV_Cov=1;CCDG_SV_Cov=1;TOPMed_Count=0;Best_TOPMed_Mismatch_ID=DUP_22:33131875-33262184;Best_TOPMed_Mismatch_OFP=0.00214871;Best_TOPMed_Mismatch_SVTYPE=DUP;Best_TOPMed_Mismatch_AF=7e-06;Best_TOPMed_Mismatch_Het=2;Best_TOPMed_Mismatch_HomAlt=0;TOPMed_SV_Cov=0;ThousG_Count=0;ThousG_SV_Cov=0;gnomAD_Count=0;Best_gnomAD_ID=gnomAD-SV_v3_DEL_chr22_b967dc51;Best_gnomAD_OFP=0.00200649;Best_gnomAD_AF=1.6e-05;Best_gnomAD_Het=2;Best_gnomAD_HomAlt=0;Best_gnomAD_PopMax_AF=0.000159;Best_gnomAD_Mismatch_ID=gnomAD-SV_v3_DUP_chr22_38e9d68d;Best_gnomAD_Mismatch_OFP=0.00214871;Best_gnomAD_Mismatch_SVTYPE=DUP;Best_gnomAD_Mismatch_AF=1.6e-05;Best_gnomAD_Mismatch_Het=2;Best_gnomAD_Mismatch_HomAlt=0;gnomAD_SV_Cov=1	GT:PSV:LN:DR:ST:QV:TY:ID:RAL:AAL:CO	0/1:NA:280:0,0:+:900:DEL:DEL00061816:NA:NA:chr22_33237876-chr22_33238156	0/1:NA:279:0,0:+:753:DEL:MantaDEL_94356_0_1_0_0_0:AAATAAATGTGCATAACGCACGGGAGTACAGTATGGTAAAGGAAAGGGAGTGGAGGCTGACATCACATTCAGAGGCTGTATTTTAAGATGAAAGGTAACATATATGTAGTTTGTCTCAAAGTTATATCTTGAGACCAGGATGGGGACGAGGAAAGGGGACAGAAGAAAAAGGTTTCAAATGCAGTTTGAGGCTCAGCTGCTAAGCTGCTGGTTACAATAATAATAAATTGGAAACAACAACCAAAAAAGCTGTTTAGAGTTTTTAAAGGAGCACAAATAT:A:chr22_33237876-chr22_33238155	0/1:NA:277:0,0:+:827:DEL:11292:NA:NA:chr22_33237878-chr22_33238155	split	q12.3	LARGE1				XR_007067993	1	33066662	33920476	281	0	0	no	16	intron14-intron14	UTR	45045	5'	33237875	33238156																																																													30	0							congenital muscular dystrophy with intellectual disability;muscular dystrophy-dystroglycanopathy (congenital with brain and eye anomalies), type A6;muscular dystrophy-dystroglycanopathy type B6;muscular dystrophy-dystroglycanopathy, type A	AR	Definitive;Strong;Supportive	12966029;17436019;19067344;19299310;21248746;21397493[PMID];21727005[PMID]_19299310[PMID]	9215	603590	Muscular dystrophy-dystroglycanopathy (congenital with brain and eye anomalies), type A, 6, 613154 (3) AR;Muscular dystrophy-dystroglycanopathy (congenital with impaired intellectual development), type B, 6, 608840 (3) AR	AR						0.7369	Cleft lip;Cleft palate;Microphthalmia;Walker-Warburg syndrome	Cleft lip;Cleft palate;Holoprosencephaly;Hypertelorism;Micrognathia;Microphthalmia;abnormal myelination;abnormal tongue morphology				full=3	0/1
11_1894044_1915726_DEL_1	11	1894044	1915726	-21681	DEL	ILS_AP001,ILS_AP001_1,ILS_AP001_2	MantaDEL:25948:0:1:0:0:0	G		830	PASS	SUPP=3;SUPP_VEC=111;SVLEN=-21681;SVTYPE=DEL;SVMETHOD=SURVIVOR1.0.7;CHR2=11;END=1915725;CIPOS=0,3;CIEND=0,1;STRANDS=+;Max_AF=0;Max_Het=0;Max_HomAlt=0;Max_PopMax_AF=0;CCDG_Count=0;Best_CCDG_ID=159323;Best_CCDG_OFP=0.312102;Best_CCDG_AF=3.419e-05;Best_CCDG_Het=0;Best_CCDG_HomAlt=0;Best_CCDG_PopMax_AF=0;Best_CCDG_Mismatch_ID=551838;Best_CCDG_Mismatch_OFP=0.000310112;Best_CCDG_Mismatch_SVTYPE=INV;Best_CCDG_Mismatch_AF=0.01792;Best_CCDG_Mismatch_Het=0;Best_CCDG_Mismatch_HomAlt=0;SV_Cov=1;CCDG_SV_Cov=0.341389;TOPMed_Count=0;Best_TOPMed_ID=DEL_11:1905428-1912864;Best_TOPMed_OFP=0.34305;Best_TOPMed_AF=1.8e-05;Best_TOPMed_Het=5;Best_TOPMed_HomAlt=0;Best_TOPMed_PopMax_AF=3.13138e-05;Best_TOPMed_Mismatch_ID=INV_11:1707647-1896713;Best_TOPMed_Mismatch_OFP=0.00173902;Best_TOPMed_Mismatch_SVTYPE=INV;Best_TOPMed_Mismatch_AF=2.9e-05;Best_TOPMed_Mismatch_Het=8;Best_TOPMed_Mismatch_HomAlt=0;TOPMed_SV_Cov=1;ThousG_Count=0;Best_ThousG_ID=HGSV_21180;Best_ThousG_OFP=0.018633;Best_ThousG_AF=0.005781;Best_ThousG_Het=37;Best_ThousG_HomAlt=0;Best_ThousG_PopMax_AF=0.01636;Best_ThousG_Mismatch_ID=HGSV_21179;Best_ThousG_Mismatch_OFP=0.331427;Best_ThousG_Mismatch_SVTYPE=DUP;Best_ThousG_Mismatch_AF=0.000156;Best_ThousG_Mismatch_Het=1;Best_ThousG_Mismatch_HomAlt=0;ThousG_SV_Cov=0.018633;gnomAD_Count=0;Best_gnomAD_ID=gnomAD-SV_v3_DEL_chr11_0a7bb213;Best_gnomAD_OFP=0.34305;Best_gnomAD_AF=8e-06;Best_gnomAD_Het=1;Best_gnomAD_HomAlt=0;Best_gnomAD_PopMax_AF=1.7e-05;Best_gnomAD_Mismatch_ID=gnomAD-SV_v3_DUP_chr11_5791e2d7;Best_gnomAD_Mismatch_OFP=0.330274;Best_gnomAD_Mismatch_SVTYPE=DUP;Best_gnomAD_Mismatch_AF=1.6e-05;Best_gnomAD_Mismatch_Het=2;Best_gnomAD_Mismatch_HomAlt=0;gnomAD_SV_Cov=0.620976	GT:PSV:LN:DR:ST:QV:TY:ID:RAL:AAL:CO	1/1:NA:21679:0,0:+:600:DEL:DEL00033993:NA:NA:chr11_1894047-chr11_1915726	1/1:NA:21681:0,0:+:830:DEL:MantaDEL_25948_0_1_0_0_0:NA:NA:chr11_1894044-chr11_1915725	1/1:NA:21681:0,0:+:827:DEL:6377:NA:NA:chr11_1894044-chr11_1915725	split	p15.5	LINC01150				NR_120534	1	1896758	1908359	11601	0	0	no	4	txStart-txEnd	UTR			1896758	1908359																													1000g;CMRI:0_pbsv.DEL.123_duplicate4;DDD:34791;HI40:ISCA-46652;HPRC:pbsv.DEL.895;dbVar;esv2666901;esv3625119	chr11:1894005-1915726;11:1894006-1915726;11:1894006-1915726;11:1894016-1915772;11:1894020-1915779;11:1894044-1915722;chr11:1894044-1915722;11:1894045-1915726	0.9522				IMH	11:1621661-71538522	0.0179																																				1.02E+08									0						full=1	1/1
3_109455011_109455111_DEL_1	3	1.09E+08	1.09E+08	-99	DEL	ILS_AP001,ILS_AP001_1,ILS_AP001_2	MantaDEL:164604:0:1:0:0:0	TTGTGAAAGACCTGACTCTGAGGCAGAGTTCTTCGGTGGATTCAACCACCTCCCACCAGGTCCCTCCCACAACACGTGGGAATTCAAGATGAGATTTGGG	T	523	PASS	SUPP=3;SUPP_VEC=111;SVLEN=-99;SVTYPE=DEL;SVMETHOD=SURVIVOR1.0.7;CHR2=3;END=109455110;CIPOS=0,2;CIEND=0,1;STRANDS=+;Max_AF=0;Max_Het=0;Max_HomAlt=0;Max_PopMax_AF=0;CCDG_Count=0;Best_CCDG_ID=322619;Best_CCDG_OFP=0.00047242;Best_CCDG_AF=3.419e-05;Best_CCDG_Het=0;Best_CCDG_HomAlt=0;Best_CCDG_PopMax_AF=0;Best_CCDG_Mismatch_ID=508909;Best_CCDG_Mismatch_OFP=0.00323572;Best_CCDG_Mismatch_SVTYPE=DUP;Best_CCDG_Mismatch_AF=0.000171;Best_CCDG_Mismatch_Het=0;Best_CCDG_Mismatch_HomAlt=0;SV_Cov=1;CCDG_SV_Cov=1;TOPMed_Count=0;Best_TOPMed_ID=DEL_3:109369440-109465112;Best_TOPMed_OFP=0.00104522;Best_TOPMed_AF=4e-06;Best_TOPMed_Het=1;Best_TOPMed_HomAlt=0;Best_TOPMed_PopMax_AF=6.28228e-06;Best_TOPMed_Mismatch_ID=DUP_3:109424903-109455806;Best_TOPMed_Mismatch_OFP=0.00323572;Best_TOPMed_Mismatch_SVTYPE=DUP;Best_TOPMed_Mismatch_AF=0.000229;Best_TOPMed_Mismatch_Het=59;Best_TOPMed_Mismatch_HomAlt=2;TOPMed_SV_Cov=1;ThousG_Count=0;Best_ThousG_Mismatch_ID=HGSV_103179;Best_ThousG_Mismatch_OFP=0.00323572;Best_ThousG_Mismatch_SVTYPE=DUP;Best_ThousG_Mismatch_AF=0.000468;Best_ThousG_Mismatch_Het=3;Best_ThousG_Mismatch_HomAlt=0;ThousG_SV_Cov=0;gnomAD_Count=0;Best_gnomAD_ID=gnomAD-SV_v3_DEL_chr3_646c3991;Best_gnomAD_OFP=0.00104523;Best_gnomAD_AF=8e-06;Best_gnomAD_Het=1;Best_gnomAD_HomAlt=0;Best_gnomAD_PopMax_AF=1.7e-05;Best_gnomAD_Mismatch_ID=gnomAD-SV_v3_DUP_chr3_444bdf92;Best_gnomAD_Mismatch_OFP=0.00323572;Best_gnomAD_Mismatch_SVTYPE=DUP;Best_gnomAD_Mismatch_AF=0.000159;Best_gnomAD_Mismatch_Het=20;Best_gnomAD_Mismatch_HomAlt=0;gnomAD_SV_Cov=1	GT:PSV:LN:DR:ST:QV:TY:ID:RAL:AAL:CO	0/1:NA:100:0,0:+:420:DEL:DEL00010828:NA:NA:chr3_109455011-chr3_109455111	0/1:NA:99:0,0:+:523:DEL:MantaDEL_164604_0_1_0_0_0:TTGTGAAAGACCTGACTCTGAGGCAGAGTTCTTCGGTGGATTCAACCACCTCCCACCAGGTCCCTCCCACAACACGTGGGAATTCAAGATGAGATTTGGG:T:chr3_109455011-chr3_109455110	0/1:NA:97:0,0:+:293:DEL:2228:NA:NA:chr3_109455013-chr3_109455110	split	q13.13	LINC01205				NR_109841	1	1.09E+08	1.09E+08	101	0	0	no	5	intron2-intron2	UTR	4771	3'	1.09E+08	1.09E+08																										dbVar	chr3:109424902-109455806	0.01	dbVar;gnomAD-SV_v3_DEL_chr3_9b8d4286	chr3:11110259-145805573;chr3:108633893-111492183;chr3:109441411-109471369;chr3:109453096-109716138	0.0446				IMH	3:30036454-134858252;3:72502419-189537676;3:75447628-125901392	0.239																																				401082									0						full=1	0/1
1_185620013_185620102_DEL_1	1	1.86E+08	1.86E+08	-88	DEL	ILS_AP001,ILS_AP001_1,ILS_AP001_2	MantaDEL:200913:0:1:0:0:0	AATGTGTGTATATATATATGGAATCTTCATATATGTGTATATATATATATACACATATATGAAGATTCCATATATATATACACACACAT	A	239	PASS	SUPP=3;SUPP_VEC=111;SVLEN=-88;SVTYPE=DEL;SVMETHOD=SURVIVOR1.0.7;CHR2=1;END=185620101;CIPOS=0,14;CIEND=0,1;STRANDS=+;Max_AF=4.8e-05;Max_Het=6;Max_HomAlt=0;Max_PopMax_AF=0.000247;CCDG_Count=0;Best_CCDG_ID=137217;Best_CCDG_OFP=0.00985931;Best_CCDG_AF=3.419e-05;Best_CCDG_Het=0;Best_CCDG_HomAlt=0;Best_CCDG_PopMax_AF=0;Best_CCDG_Mismatch_ID=137220_1;Best_CCDG_Mismatch_OFP=0.011236;Best_CCDG_Mismatch_SVTYPE=BND;Best_CCDG_Mismatch_AF=0.06291;Best_CCDG_Mismatch_Het=0;Best_CCDG_Mismatch_HomAlt=0;SV_Cov=1;CCDG_SV_Cov=1;TOPMed_Count=0;Best_TOPMed_ID=DEL_1:185617022-185620071;Best_TOPMed_OFP=0.0128195;Best_TOPMed_AF=4e-06;Best_TOPMed_Het=1;Best_TOPMed_HomAlt=0;Best_TOPMed_PopMax_AF=6.26786e-06;Best_TOPMed_Mismatch_ID=INV_1:183856810-187909288;Best_TOPMed_Mismatch_OFP=2.19619e-05;Best_TOPMed_Mismatch_SVTYPE=INV;Best_TOPMed_Mismatch_AF=4e-06;Best_TOPMed_Mismatch_Het=1;Best_TOPMed_Mismatch_HomAlt=0;TOPMed_SV_Cov=0.662921;ThousG_Count=0;Best_ThousG_Mismatch_ID=HGSV_9278;Best_ThousG_Mismatch_OFP=0.418323;Best_ThousG_Mismatch_SVTYPE=INS;Best_ThousG_Mismatch_AF=0.000625;Best_ThousG_Mismatch_Het=4;Best_ThousG_Mismatch_HomAlt=0;ThousG_SV_Cov=0;gnomAD_Count=2;Best_gnomAD_ID=gnomAD-SV_v3_DEL_chr1_91caac2e;Best_gnomAD_OFP=0.842697;Best_gnomAD_AF=3.3e-05;Best_gnomAD_Het=4;Best_gnomAD_HomAlt=0;Best_gnomAD_PopMax_AF=5.1e-05;Best_gnomAD_Mismatch_ID=gnomAD-SV_v3_INS_chr1_5eaa9906;Best_gnomAD_Mismatch_OFP=0.494382;Best_gnomAD_Mismatch_SVTYPE=INS;Best_gnomAD_Mismatch_AF=0.004189;Best_gnomAD_Mismatch_Het=524;Best_gnomAD_Mismatch_HomAlt=2;gnomAD_SV_Cov=1	GT:PSV:LN:DR:ST:QV:TY:ID:RAL:AAL:CO	0/1:NA:89:0,0:+:239:DEL:DEL00004115:NA:NA:chr1_185620013-chr1_185620102	0/1:NA:88:0,0:+:139:DEL:MantaDEL_200913_0_1_0_0_0:AATGTGTGTATATATATATGGAATCTTCATATATGTGTATATATATATATACACATATATGAAGATTCCATATATATATACACACACAT:A:chr1_185620013-chr1_185620101	0/1:NA:74:0,0:+:83:DEL:818:NA:NA:chr1_185620027-chr1_185620101	split	q25.3	LINC01350				NR_110793	1	1.86E+08	1.86E+08	90	0	0	no	4	intron2-intron2	UTR	6389	5'	1.86E+08	1.86E+08																													gnomAD-SV_v3_DEL_chr1_339fd21d	chr1:168349020-187167586	0.9262				IMH	1:10187873-186857210;1:16798094-234784887;1:68663392-188869781;1:107555343-232824950	0.4698																																				1.02E+08									0						full=1	0/1
12_127057469_127058489_DEL_1	12	1.27E+08	1.27E+08	-1019	DEL	ILS_AP001,ILS_AP001_1,ILS_AP001_2	MantaDEL:24988:0:1:0:0:0	C		1208	PASS	SUPP=3;SUPP_VEC=111;SVLEN=-1019;SVTYPE=DEL;SVMETHOD=SURVIVOR1.0.7;CHR2=12;END=127058488;CIPOS=0,0;CIEND=0,1;STRANDS=+;Max_AF=0.002342;Max_Het=26;Max_HomAlt=6;Max_PopMax_AF=0.011111;CCDG_Count=1;Best_CCDG_ID=189451;Best_CCDG_OFP=1;Best_CCDG_AF=0.0002052;Best_CCDG_Het=0;Best_CCDG_HomAlt=0;Best_CCDG_PopMax_AF=0;Best_CCDG_Mismatch_ID=473555;Best_CCDG_Mismatch_OFP=0.000673294;Best_CCDG_Mismatch_SVTYPE=DUP;Best_CCDG_Mismatch_AF=3.419e-05;Best_CCDG_Mismatch_Het=0;Best_CCDG_Mismatch_HomAlt=0;SV_Cov=1;CCDG_SV_Cov=1;TOPMed_Count=0;Best_TOPMed_ID=DEL_12:127053980-127059275;Best_TOPMed_OFP=0.192562;Best_TOPMed_AF=1.8e-05;Best_TOPMed_Het=5;Best_TOPMed_HomAlt=0;Best_TOPMed_PopMax_AF=0.000319829;Best_TOPMed_Mismatch_ID=DUP_12:127022996-127106412;Best_TOPMed_Mismatch_OFP=0.0122276;Best_TOPMed_Mismatch_SVTYPE=DUP;Best_TOPMed_Mismatch_AF=1.1e-05;Best_TOPMed_Mismatch_Het=3;Best_TOPMed_Mismatch_HomAlt=0;TOPMed_SV_Cov=1;ThousG_Count=1;Best_ThousG_ID=HGSV_36909;Best_ThousG_OFP=1;Best_ThousG_AF=0.002342;Best_ThousG_Het=11;Best_ThousG_HomAlt=2;Best_ThousG_PopMax_AF=0.011111;ThousG_SV_Cov=1;gnomAD_Count=1;Best_gnomAD_ID=gnomAD-SV_v3_DEL_chr12_1208cb5d;Best_gnomAD_OFP=1;Best_gnomAD_AF=0.000301;Best_gnomAD_Het=26;Best_gnomAD_HomAlt=6;Best_gnomAD_PopMax_AF=0.005673;Best_gnomAD_Mismatch_ID=gnomAD-SV_v3_DUP_chr12_ba1f336a;Best_gnomAD_Mismatch_OFP=0.0122276;Best_gnomAD_Mismatch_SVTYPE=DUP;Best_gnomAD_Mismatch_AF=8e-06;Best_gnomAD_Mismatch_Het=1;Best_gnomAD_Mismatch_HomAlt=0;gnomAD_SV_Cov=1	GT:PSV:LN:DR:ST:QV:TY:ID:RAL:AAL:CO	0/1:NA:1020:18,10:+:1208:DEL:DEL00039909:NA:NA:chr12_127057469-chr12_127058489	0/1:NA:1019:0,0:+:738:DEL:MantaDEL_24988_0_1_0_0_0:NA:NA:chr12_127057469-chr12_127058488	0/1:NA:1019:0,0:+:638:DEL:7506:NA:NA:chr12_127057469-chr12_127058488	split	q24.32	LINC02405				NR_104646	1	1.27E+08	1.27E+08	1021	0	0	no	7	intron1-intron1	UTR	1597	5'	1.27E+08	1.27E+08																										1000g;dbVar;esv3631095	chr12:126267752-127405141;chr12:126945957-127328431;12:126945958-127328431;12:126945958-127328431	0.2423	dbVar	chr12:126929324-127229788;chr12:126945957-127328431;chr12:127051823-127065815;chr12:127053979-127059273	0.01																																										1.02E+08									0						full=1	0/1
4_32069419_32072988_DUP_1	4	32069419	32072988	3567	DUP	ILS_AP001,ILS_AP001_1,ILS_AP001_2	MantaDUP:TANDEM:146148:0:4:0:3:0	A		780	PASS	SUPP=3;SUPP_VEC=111;SVLEN=3567;SVTYPE=DUP;SVMETHOD=SURVIVOR1.0.7;CHR2=4;END=32072986;CIPOS=0,1;CIEND=0,2;STRANDS=-;Max_AF=0;Max_Het=0;Max_HomAlt=0;Max_PopMax_AF=0;CCDG_Count=0;Best_CCDG_Mismatch_ID=335440_2;Best_CCDG_Mismatch_OFP=0.000280269;Best_CCDG_Mismatch_SVTYPE=BND;Best_CCDG_Mismatch_AF=0.0769;Best_CCDG_Mismatch_Het=0;Best_CCDG_Mismatch_HomAlt=0;SV_Cov=1;CCDG_SV_Cov=0;TOPMed_Count=0;Best_TOPMed_ID=DUP_4:32067402-32083700;Best_TOPMed_OFP=0.218896;Best_TOPMed_AF=1.8e-05;Best_TOPMed_Het=5;Best_TOPMed_HomAlt=0;Best_TOPMed_PopMax_AF=3.15235e-05;Best_TOPMed_Mismatch_ID=DEL_4:32071446-32071490;Best_TOPMed_Mismatch_OFP=0.0128924;Best_TOPMed_Mismatch_SVTYPE=DEL;Best_TOPMed_Mismatch_AF=0.733614;Best_TOPMed_Mismatch_Het=49055;Best_TOPMed_Mismatch_HomAlt=74776;TOPMed_SV_Cov=1;ThousG_Count=0;Best_ThousG_ID=HGSV_110436;Best_ThousG_OFP=0.0746085;Best_ThousG_AF=0.000156;Best_ThousG_Het=1;Best_ThousG_HomAlt=0;Best_ThousG_PopMax_AF=0.000832;ThousG_SV_Cov=1;gnomAD_Count=0;Best_gnomAD_ID=gnomAD-SV_v3_DUP_chr4_3dfbb6eb;Best_gnomAD_OFP=0.775484;Best_gnomAD_AF=8e-06;Best_gnomAD_Het=1;Best_gnomAD_HomAlt=0;Best_gnomAD_PopMax_AF=1.7e-05;Best_gnomAD_Mismatch_ID=gnomAD-SV_v3_DEL_chr4_169d334c;Best_gnomAD_Mismatch_OFP=0.342667;Best_gnomAD_Mismatch_SVTYPE=DEL;Best_gnomAD_Mismatch_AF=8e-06;Best_gnomAD_Mismatch_Het=1;Best_gnomAD_Mismatch_HomAlt=0;gnomAD_SV_Cov=1	GT:PSV:LN:DR:ST:QV:TY:ID:RAL:AAL:CO	0/1:NA:3566:23,5:-:780:DUP:DUP00012709:NA:NA:chr4_32069420-chr4_32072986	0/1:NA:3567:0,0:-:234:DUP:MantaDUP_TANDEM_146148_0_4_0_3_0:NA:NA:chr4_32069419-chr4_32072986	0/1:NA:3569:0,0:-:179:DUP:2628:NA:NA:chr4_32069419-chr4_32072988	split	p15.1	LINC02506				NR_125936	1	31997378	32155406	3570	0	0	no	3	intron1-intron1	UTR	71596	5'	32069418	32072988																													dbVar;gnomAD-SV_v3_DEL_chr4_6939d627	chr4:22150396-36380550;chr4:31682402-32265926;chr4:31692223-32398776	0.0703				IMH	4:1330317-92330038;4:4772332-76608755;4:21291890-139485605;4:21757282-83521389	0.0791																																				1.03E+08									0						full=3	0/1
5_96989313_96989413_DEL_1	5	96989313	96989413	-100	DEL	ILS_AP001,ILS_AP001_1,ILS_AP001_2	MantaDEL:137680:0:1:0:0:0	TTATATATAATATATAATTATATATAATATATAATATATTATATATTATATATAATTATATATTATATATTATATACAATATATTATATATTATATATAAT	TA	840	PASS	SUPP=3;SUPP_VEC=111;SVLEN=-100;SVTYPE=DEL;SVMETHOD=SURVIVOR1.0.7;CHR2=5;END=96989413;CIPOS=0,6;CIEND=-18,0;STRANDS=+;Max_AF=0;Max_Het=0;Max_HomAlt=0;Max_PopMax_AF=0;CCDG_Count=0;Best_CCDG_ID=360884;Best_CCDG_OFP=0.712871;Best_CCDG_AF=0.5915;Best_CCDG_Het=0;Best_CCDG_HomAlt=0;Best_CCDG_PopMax_AF=0;Best_CCDG_Mismatch_ID=518287;Best_CCDG_Mismatch_OFP=0.00013066;Best_CCDG_Mismatch_SVTYPE=DUP;Best_CCDG_Mismatch_AF=3.419e-05;Best_CCDG_Mismatch_Het=0;Best_CCDG_Mismatch_HomAlt=0;SV_Cov=1;CCDG_SV_Cov=1;TOPMed_Count=0;Best_TOPMed_ID=DEL_5:94767306-97708544;Best_TOPMed_OFP=3.43393e-05;Best_TOPMed_AF=4e-06;Best_TOPMed_Het=1;Best_TOPMed_HomAlt=0;Best_TOPMed_PopMax_AF=6.26991e-06;Best_TOPMed_Mismatch_ID=DUP_5:96412204-97185198;Best_TOPMed_Mismatch_OFP=0.00013066;Best_TOPMed_Mismatch_SVTYPE=DUP;Best_TOPMed_Mismatch_AF=4e-06;Best_TOPMed_Mismatch_Het=1;Best_TOPMed_Mismatch_HomAlt=0;TOPMed_SV_Cov=1;ThousG_Count=0;Best_ThousG_ID=HGSV_125011;Best_ThousG_OFP=0.712871;Best_ThousG_AF=0.543035;Best_ThousG_Het=1813;Best_ThousG_HomAlt=797;Best_ThousG_PopMax_AF=0.588296;Best_ThousG_Mismatch_ID=HGSV_123838;Best_ThousG_Mismatch_OFP=3.0536e-06;Best_ThousG_Mismatch_SVTYPE=INV;Best_ThousG_Mismatch_AF=0.000156;Best_ThousG_Mismatch_Het=1;Best_ThousG_Mismatch_HomAlt=0;ThousG_SV_Cov=0.712871;gnomAD_Count=0;Best_gnomAD_ID=gnomAD-SV_v3_DEL_chr5_10bc6569;Best_gnomAD_OFP=0.712871;Best_gnomAD_AF=0.464419;Best_gnomAD_Het=36288;Best_gnomAD_HomAlt=8294;Best_gnomAD_PopMax_AF=0.557355;gnomAD_Mismatches=gnomAD-SV_v3_DUP_chr5_e1bfdcf7,gnomAD-SV_v3_DUP_chr5_d71655ea;gnomAD_Mismatches_Count=2;gnomAD_Mismatch_SVTYPEs=DUP;Best_gnomAD_Mismatch_ID=gnomAD-SV_v3_DUP_chr5_d71655ea;Best_gnomAD_Mismatch_OFP=0.942132;Best_gnomAD_Mismatch_SVTYPE=DUP;Best_gnomAD_Mismatch_AF=0.00069;Best_gnomAD_Mismatch_Het=75;Best_gnomAD_Mismatch_HomAlt=0;gnomAD_SV_Cov=1	GT:PSV:LN:DR:ST:QV:TY:ID:RAL:AAL:CO	0/1:NA:99:0,0:+:840:DEL:DEL00017100:NA:NA:chr5_96989313-chr5_96989412	0/1:NA:100:0,0:+:569:DEL:MantaDEL_137680_0_1_0_0_0:TTATATATAATATATAATTATATATAATATATAATATATTATATATTATATATAATTATATATTATATATTATATACAATATATTATATATTATATATAAT:TA:chr5_96989313-chr5_96989413	0/1:NA:76:0,0:+:137:DEL:3366:NA:NA:chr5_96989319-chr5_96989395	split	q15	LNPEP				NM_005575	3	96936079	97037513	101	0	0	no	18	intron4-intron4	CDS	2642	5'	96989312	96989413																													IMH	5:96989258-96989487	0.5915				IMH	5:24758176-102357714;5:58813595-102383104;5:85192778-162907706;5:96855635-159536527	0.5229																										30.65	-0.25318	1.308589	0.738283	-1.2897	0.496385					4012						0	1.00E+00	9.98E-01	0						full=1	0/1
14_64893668_64896823_DEL_1	14	64893668	64896823	-3154	DEL	ILS_AP001,ILS_AP001_1,ILS_AP001_2	MantaDEL:2676:14:16:0:0:0	A		1980	PASS	SUPP=3;SUPP_VEC=111;SVLEN=-3154;SVTYPE=DEL;SVMETHOD=SURVIVOR1.0.7;CHR2=14;END=64896822;CIPOS=0,10;CIEND=0,1;STRANDS=+;Max_AF=0;Max_Het=0;Max_HomAlt=0;Max_PopMax_AF=0;CCDG_Count=0;Best_CCDG_Mismatch_ID=85670_2;Best_CCDG_Mismatch_OFP=0.000316957;Best_CCDG_Mismatch_SVTYPE=BND;Best_CCDG_Mismatch_AF=6.839e-05;Best_CCDG_Mismatch_Het=0;Best_CCDG_Mismatch_HomAlt=0;SV_Cov=1;CCDG_SV_Cov=0;TOPMed_Count=0;Best_TOPMed_ID=DEL_14:64884040-64901316;Best_TOPMed_OFP=0.182602;Best_TOPMed_AF=2e-05;Best_TOPMed_Het=5;Best_TOPMed_HomAlt=0;Best_TOPMed_PopMax_AF=0.000365586;Best_TOPMed_Mismatch_ID=DUP_14:64880628-64904139;Best_TOPMed_Mismatch_OFP=0.134181;Best_TOPMed_Mismatch_SVTYPE=DUP;Best_TOPMed_Mismatch_AF=4e-06;Best_TOPMed_Mismatch_Het=1;Best_TOPMed_Mismatch_HomAlt=0;TOPMed_SV_Cov=1;ThousG_Count=0;ThousG_SV_Cov=0;gnomAD_Count=0;Best_gnomAD_ID=gnomAD-SV_v3_DEL_chr14_e5ca311d;Best_gnomAD_OFP=0.182623;Best_gnomAD_AF=3.2e-05;Best_gnomAD_Het=4;Best_gnomAD_HomAlt=0;Best_gnomAD_PopMax_AF=6.8e-05;Best_gnomAD_Mismatch_ID=gnomAD-SV_v3_DUP_chr14_55ea796c;Best_gnomAD_Mismatch_OFP=0.62143;Best_gnomAD_Mismatch_SVTYPE=DUP;Best_gnomAD_Mismatch_AF=0.025569;Best_gnomAD_Mismatch_Het=2157;Best_gnomAD_Mismatch_HomAlt=97;gnomAD_SV_Cov=1	GT:PSV:LN:DR:ST:QV:TY:ID:RAL:AAL:CO	0/1:NA:3155:9,16:+:1980:DEL:DEL00042387:NA:NA:chr14_64893668-chr14_64896823	0/1:NA:3154:0,0:+:999:DEL:MantaDEL_2676_14_16_0_0_0:NA:NA:chr14_64893668-chr14_64896822	0/1:NA:3144:0,0:+:968:DEL:8075:NA:NA:chr14_64893678-chr14_64896822	split	q23.3	LOC105370534				XR_943942	3	64880880	64902128	3156	0	0	no	3	intron1-intron1	UTR	5248	5'	64893667	64896823																										gnomAD-SV_v3_DUP_chr14_55ea796c	chr14:64892637-64897713	0.0539	dbVar;gnomAD-SV_v3_DEL_chr14_040215df;gnomAD-SV_v3_DEL_chr14_aa7bf92b	chr14:41140072-69277618;chr14:56471274-99420950;chr14:64884041-64901316	0.633				IMH	14:23629193-97505158	0.4022																																													-1						full=1	0/1
19_9516280_9516365_DEL_1	19	9516280	9516365	-84	DEL	ILS_AP001,ILS_AP001_1,ILS_AP001_2	MantaDEL:80093:0:1:0:0:0	CAGTGGCCAGGCATGGTGGCTCATGCCTGTAATCCCAGCACTTTGGGAGGCTGAGGTGGTTGGATCACCTGAGGCTGGGAGTTTG	C	399	PASS	SUPP=3;SUPP_VEC=111;SVLEN=-84;SVTYPE=DEL;SVMETHOD=SURVIVOR1.0.7;CHR2=19;END=9516364;CIPOS=0,2;CIEND=0,1;STRANDS=+;Max_AF=0.005932;Max_Het=90;Max_HomAlt=13;Max_PopMax_AF=0.031614;CCDG_Count=0;Best_CCDG_Mismatch_ID=255366_1;Best_CCDG_Mismatch_OFP=0.0117647;Best_CCDG_Mismatch_SVTYPE=BND;Best_CCDG_Mismatch_AF=0.001641;Best_CCDG_Mismatch_Het=0;Best_CCDG_Mismatch_HomAlt=0;SV_Cov=1;CCDG_SV_Cov=0;TOPMed_Count=0;Best_TOPMed_ID=DEL_19:9515494-9519966;Best_TOPMed_OFP=0.0189987;Best_TOPMed_AF=7e-06;Best_TOPMed_Het=2;Best_TOPMed_HomAlt=0;Best_TOPMed_PopMax_AF=2.40125e-05;Best_TOPMed_Mismatch_ID=INV_19:9514942-9524335;Best_TOPMed_Mismatch_OFP=0.00904737;Best_TOPMed_Mismatch_SVTYPE=INV;Best_TOPMed_Mismatch_AF=1.8e-05;Best_TOPMed_Mismatch_Het=5;Best_TOPMed_Mismatch_HomAlt=0;TOPMed_SV_Cov=1;ThousG_Count=1;Best_ThousG_ID=HGSV_70656;Best_ThousG_OFP=0.976471;Best_ThousG_AF=0.005932;Best_ThousG_Het=34;Best_ThousG_HomAlt=2;Best_ThousG_PopMax_AF=0.031614;ThousG_SV_Cov=0.976471;gnomAD_Count=1;Best_gnomAD_ID=gnomAD-SV_v3_DEL_chr19_6613b8a4;Best_gnomAD_OFP=0.976471;Best_gnomAD_AF=0.000921;Best_gnomAD_Het=90;Best_gnomAD_HomAlt=13;Best_gnomAD_PopMax_AF=0.026232;Best_gnomAD_Mismatch_ID=gnomAD-SV_v3_CPX_chr19_66bc3b95;Best_gnomAD_Mismatch_OFP=1.04375e-05;Best_gnomAD_Mismatch_SVTYPE=CPX;Best_gnomAD_Mismatch_AF=0.000158;Best_gnomAD_Mismatch_Het=5;Best_gnomAD_Mismatch_HomAlt=0;gnomAD_SV_Cov=1	GT:PSV:LN:DR:ST:QV:TY:ID:RAL:AAL:CO	0/1:NA:85:0,0:+:399:DEL:DEL00054768:NA:NA:chr19_9516280-chr19_9516365	0/1:NA:84:0,0:+:398:DEL:MantaDEL_80093_0_1_0_0_0:CAGTGGCCAGGCATGGTGGCTCATGCCTGTAATCCCAGCACTTTGGGAGGCTGAGGTGGTTGGATCACCTGAGGCTGGGAGTTTG:C:chr19_9516280-chr19_9516364	0/1:NA:82:0,0:+:212:DEL:10163:NA:NA:chr19_9516282-chr19_9516364	split	p13.2	LOC105372268				XR_001753862	2	9507807	9520840	86	0	0	no	3	intron1-intron1	UTR	1625	3'	9516279	9516365																													dbVar	chr19:9504987-9533322	0.01				IMH	19:1692676-41507766	0.0103																																													-1						full=1	0/1
20_17303439_17303526_DEL_1	20	17303439	17303526	-84	DEL	ILS_AP001,ILS_AP001_1,ILS_AP001_2	MantaDEL:97657:0:1:0:0:0	AATATATATTATATTAAATATAATATATATTATATATAATATATATTATATTTAATATAATATATATTATATATAATATAATATA	AT	415	PASS	SUPP=3;SUPP_VEC=111;SVLEN=-84;SVTYPE=DEL;SVMETHOD=SURVIVOR1.0.7;CHR2=20;END=17303526;CIPOS=-3,9;CIEND=-10,0;STRANDS=+;Max_AF=0;Max_Het=0;Max_HomAlt=0;Max_PopMax_AF=0;CCDG_Count=0;Best_CCDG_ID=293077;Best_CCDG_OFP=0.0182639;Best_CCDG_AF=0.0001026;Best_CCDG_Het=0;Best_CCDG_HomAlt=0;Best_CCDG_PopMax_AF=0;Best_CCDG_Mismatch_ID=293079_1;Best_CCDG_Mismatch_OFP=0.0117647;Best_CCDG_Mismatch_SVTYPE=BND;Best_CCDG_Mismatch_AF=0.227;Best_CCDG_Mismatch_Het=0;Best_CCDG_Mismatch_HomAlt=0;SV_Cov=1;CCDG_SV_Cov=1;TOPMed_Count=0;Best_TOPMed_Mismatch_ID=DUP_20:17274064-17318502;Best_TOPMed_Mismatch_OFP=0.00191269;Best_TOPMed_Mismatch_SVTYPE=DUP;Best_TOPMed_Mismatch_AF=7e-06;Best_TOPMed_Mismatch_Het=2;Best_TOPMed_Mismatch_HomAlt=0;TOPMed_SV_Cov=0;ThousG_Count=0;Best_ThousG_Mismatch_ID=HGSV_89585;Best_ThousG_Mismatch_OFP=0.000140685;Best_ThousG_Mismatch_SVTYPE=DUP;Best_ThousG_Mismatch_AF=0.000156;Best_ThousG_Mismatch_Het=1;Best_ThousG_Mismatch_HomAlt=0;ThousG_SV_Cov=0;gnomAD_Count=0;Best_gnomAD_ID=gnomAD-SV_v3_DEL_chr20_94becb03;Best_gnomAD_OFP=0.729412;Best_gnomAD_AF=0.04733;Best_gnomAD_Het=4091;Best_gnomAD_HomAlt=1;Best_gnomAD_PopMax_AF=0.166667;gnomAD_Mismatches=gnomAD-SV_v3_DUP_chr20_91afbc86;gnomAD_Mismatches_Count=1;gnomAD_Mismatch_SVTYPEs=DUP;Best_gnomAD_Mismatch_ID=gnomAD-SV_v3_DUP_chr20_91afbc86;Best_gnomAD_Mismatch_OFP=0.835294;Best_gnomAD_Mismatch_SVTYPE=DUP;Best_gnomAD_Mismatch_AF=0.036155;Best_gnomAD_Mismatch_Het=4531;Best_gnomAD_Mismatch_HomAlt=9;gnomAD_SV_Cov=1	GT:PSV:LN:DR:ST:QV:TY:ID:RAL:AAL:CO	0/1:NA:83:0,0:+:415:DEL:DEL00058353:NA:NA:chr20_17303439-chr20_17303522	0/1:NA:84:0,0:+:385:DEL:MantaDEL_97657_0_1_0_0_0:AATATATATTATATTAAATATAATATATATTATATATAATATATATTATATTTAATATAATATATATTATATATAATATAATATA:AT:chr20_17303442-chr20_17303526	0/1:NA:65:0,0:+:68:DEL:10795:NA:NA:chr20_17303451-chr20_17303516	split	p12.1	LOC105372546				XR_007067540	1	17232476	17326898	88	0	0	no	5	intron2-intron2	UTR	21685	5'	17303438	17303526																										dbVar	chr20:17301743-17309639	0.01																																																						-1						full=3	0/1
22_44860912_44860969_DEL_1	22	44860912	44860969	-56	DEL	ILS_AP001,ILS_AP001_1,ILS_AP001_2	MantaDEL:95612:0:1:0:0:0	TTGGAGAATGTCACTTCTTGGTTTTACAACAATTTTTTTTCTCCCTGTTGCTTGACA	T	653	PASS	SUPP=3;SUPP_VEC=111;SVLEN=-56;SVTYPE=DEL;SVMETHOD=SURVIVOR1.0.7;CHR2=22;END=44860968;CIPOS=0,1;CIEND=0,1;STRANDS=+;Max_AF=0.000781;Max_Het=18;Max_HomAlt=0;Max_PopMax_AF=0.004274;CCDG_Count=0;Best_CCDG_ID=308768;Best_CCDG_OFP=0.00374237;Best_CCDG_AF=3.419e-05;Best_CCDG_Het=0;Best_CCDG_HomAlt=0;Best_CCDG_PopMax_AF=0;Best_CCDG_Mismatch_ID=504733;Best_CCDG_Mismatch_OFP=0.00542289;Best_CCDG_Mismatch_SVTYPE=DUP;Best_CCDG_Mismatch_AF=3.419e-05;Best_CCDG_Mismatch_Het=0;Best_CCDG_Mismatch_HomAlt=0;SV_Cov=1;CCDG_SV_Cov=1;TOPMed_Count=0;Best_TOPMed_ID=DEL_22:44858643-44861351;Best_TOPMed_OFP=0.0210332;Best_TOPMed_AF=4e-06;Best_TOPMed_Het=1;Best_TOPMed_HomAlt=0;Best_TOPMed_PopMax_AF=6.26535e-06;Best_TOPMed_Mismatch_ID=INV_22:44757699-44872616;Best_TOPMed_Mismatch_OFP=0.000496002;Best_TOPMed_Mismatch_SVTYPE=INV;Best_TOPMed_Mismatch_AF=4e-06;Best_TOPMed_Mismatch_Het=1;Best_TOPMed_Mismatch_HomAlt=0;TOPMed_SV_Cov=1;ThousG_Count=1;Best_ThousG_ID=HGSV_96575;Best_ThousG_OFP=0.982456;Best_ThousG_AF=0.000781;Best_ThousG_Het=5;Best_ThousG_HomAlt=0;Best_ThousG_PopMax_AF=0.004274;Best_ThousG_Mismatch_ID=HGSV_96574;Best_ThousG_Mismatch_OFP=0.00901471;Best_ThousG_Mismatch_SVTYPE=CPX;Best_ThousG_Mismatch_AF=0.000156;Best_ThousG_Mismatch_Het=1;Best_ThousG_Mismatch_HomAlt=0;ThousG_SV_Cov=0.982456;gnomAD_Count=1;Best_gnomAD_ID=gnomAD-SV_v3_DEL_chr22_e53f8373;Best_gnomAD_OFP=0.982456;Best_gnomAD_AF=0.000143;Best_gnomAD_Het=18;Best_gnomAD_HomAlt=0;Best_gnomAD_PopMax_AF=0.0042;Best_gnomAD_Mismatch_ID=gnomAD-SV_v3_DUP_chr22_abddc20b;Best_gnomAD_Mismatch_OFP=0.00712411;Best_gnomAD_Mismatch_SVTYPE=DUP;Best_gnomAD_Mismatch_AF=1.7e-05;Best_gnomAD_Mismatch_Het=2;Best_gnomAD_Mismatch_HomAlt=0;gnomAD_SV_Cov=1	GT:PSV:LN:DR:ST:QV:TY:ID:RAL:AAL:CO	0/1:NA:57:0,0:+:653:DEL:DEL00062432:NA:NA:chr22_44860912-chr22_44860969	1/1:NA:56:0,0:+:486:DEL:MantaDEL_95612_0_1_0_0_0:TTGGAGAATGTCACTTCTTGGTTTTACAACAATTTTTTTTCTCCCTGTTGCTTGACA:T:chr22_44860912-chr22_44860968	0/1:NA:55:0,0:+:272:DEL:11382:NA:NA:chr22_44860913-chr22_44860968	split	q13.31	LOC105373062				XR_938302	3	44859455	44864239	58	0	0	no	4	intron3-intron3	UTR	1428	3'	44860911	44860969																													dbVar	chr22:44820754-44869341;chr22:44837184-44861234	0.01				IMH	22:44479419-47655346	0.2479																																													-1						full=3	0/1
X_33781588_33785512_DEL_1	X	33781588	33785512	-3923	DEL	ILS_AP001,ILS_AP001_1,ILS_AP001_2	MantaDEL:2903:0:1:0:0:0	T		1315	PASS	SUPP=3;SUPP_VEC=111;SVLEN=-3923;SVTYPE=DEL;SVMETHOD=SURVIVOR1.0.7;CHR2=X;END=33785511;CIPOS=0,3;CIEND=0,1;STRANDS=+;Max_AF=0;Max_Het=0;Max_HomAlt=0;Max_PopMax_AF=0;CCDG_Count=0;Best_CCDG_Mismatch_ID=539821;Best_CCDG_Mismatch_OFP=0.0146633;Best_CCDG_Mismatch_SVTYPE=DUP;Best_CCDG_Mismatch_AF=3.419e-05;Best_CCDG_Mismatch_Het=0;Best_CCDG_Mismatch_HomAlt=0;SV_Cov=1;CCDG_SV_Cov=0;TOPMed_Count=0;Best_TOPMed_Mismatch_ID=DUP_23:33748407-33870946;Best_TOPMed_Mismatch_OFP=0.0320219;Best_TOPMed_Mismatch_SVTYPE=DUP;Best_TOPMed_Mismatch_AF=9e-06;Best_TOPMed_Mismatch_Het=2;Best_TOPMed_Mismatch_HomAlt=0;TOPMed_SV_Cov=0;ThousG_Count=0;Best_ThousG_ID=HGSV_168273;Best_ThousG_OFP=0.00554321;Best_ThousG_AF=0.002185;Best_ThousG_Het=14;Best_ThousG_HomAlt=0;Best_ThousG_PopMax_AF=0.005521;Best_ThousG_Mismatch_ID=HGSV_168277;Best_ThousG_Mismatch_OFP=0.0140504;Best_ThousG_Mismatch_SVTYPE=DUP;Best_ThousG_Mismatch_AF=0.000312;Best_ThousG_Mismatch_Het=0;Best_ThousG_Mismatch_HomAlt=1;ThousG_SV_Cov=1;gnomAD_Count=0;Best_gnomAD_ID=gnomAD-SV_v3_DEL_chrX_2bdd080e;Best_gnomAD_OFP=0.219512;Best_gnomAD_AF=9.38057e-05;Best_gnomAD_Het=9;Best_gnomAD_HomAlt=0;Best_gnomAD_PopMax_AF=0.000116722;Best_gnomAD_Mismatch_ID=gnomAD-SV_v3_DUP_chrX_fb572db6;Best_gnomAD_Mismatch_OFP=0.0211519;Best_gnomAD_Mismatch_SVTYPE=DUP;Best_gnomAD_Mismatch_AF=1.04225e-05;Best_gnomAD_Mismatch_Het=1;Best_gnomAD_Mismatch_HomAlt=0;gnomAD_SV_Cov=1	GT:PSV:LN:DR:ST:QV:TY:ID:RAL:AAL:CO	1/1:NA:3924:0,13:+:1315:DEL:DEL00063126:NA:NA:chrX_33781588-chrX_33785512	1/1:NA:3923:0,0:+:873:DEL:MantaDEL_2903_0_1_0_0_0:NA:NA:chrX_33781588-chrX_33785511	1/1:NA:3920:0,0:+:827:DEL:11464:NA:NA:chrX_33781591-chrX_33785511	split	p21.1	LOC105373153				XR_950542	4	33726365	34077114	3925	0	0	no	3	intron1-intron1	UTR	54952	5'	33781587	33785512																										dbVar	chrX:33602583-34344183	0.01	dbVar	chrX:33721460-33821194;chrX:33769575-33787450	0.01																																																			-1						full=1	1/1
4_19649216_19650455_DEL_1	4	19649216	19650455	-1238	DEL	ILS_AP001,ILS_AP001_1,ILS_AP001_2	MantaDEL:145217:0:1:0:0:0	A		1560	PASS	SUPP=3;SUPP_VEC=111;SVLEN=-1238;SVTYPE=DEL;SVMETHOD=SURVIVOR1.0.7;CHR2=4;END=19650454;CIPOS=0,1;CIEND=0,1;STRANDS=+;Max_AF=4.8e-05;Max_Het=6;Max_HomAlt=0;Max_PopMax_AF=0.001362;CCDG_Count=1;Best_CCDG_ID=334245;Best_CCDG_OFP=0.999193;Best_CCDG_AF=3.419e-05;Best_CCDG_Het=0;Best_CCDG_HomAlt=0;Best_CCDG_PopMax_AF=0;Best_CCDG_Mismatch_ID=586428;Best_CCDG_Mismatch_OFP=2.6142e-05;Best_CCDG_Mismatch_SVTYPE=INV;Best_CCDG_Mismatch_AF=3.419e-05;Best_CCDG_Mismatch_Het=0;Best_CCDG_Mismatch_HomAlt=0;SV_Cov=1;CCDG_SV_Cov=1;TOPMed_Count=0;Best_TOPMed_ID=DEL_4:19649081-19653066;Best_TOPMed_OFP=0.31076;Best_TOPMed_AF=4e-06;Best_TOPMed_Het=1;Best_TOPMed_HomAlt=0;Best_TOPMed_PopMax_AF=1.19849e-05;Best_TOPMed_Mismatch_ID=DUP_4:19295396-20168361;Best_TOPMed_Mismatch_OFP=0.0014193;Best_TOPMed_Mismatch_SVTYPE=DUP;Best_TOPMed_Mismatch_AF=4e-06;Best_TOPMed_Mismatch_Het=1;Best_TOPMed_Mismatch_HomAlt=0;TOPMed_SV_Cov=1;ThousG_Count=0;Best_ThousG_ID=HGSV_109700;Best_ThousG_OFP=0.0104568;Best_ThousG_AF=0.000312;Best_ThousG_Het=2;Best_ThousG_HomAlt=0;Best_ThousG_PopMax_AF=0.002041;Best_ThousG_Mismatch_ID=HGSV_109703;Best_ThousG_Mismatch_OFP=8.25469e-05;Best_ThousG_Mismatch_SVTYPE=DUP;Best_ThousG_Mismatch_AF=0.000156;Best_ThousG_Mismatch_Het=1;Best_ThousG_Mismatch_HomAlt=0;ThousG_SV_Cov=1;gnomAD_Count=1;Best_gnomAD_ID=gnomAD-SV_v3_DEL_chr4_c18c8389;Best_gnomAD_OFP=0.999193;Best_gnomAD_AF=4.8e-05;Best_gnomAD_Het=6;Best_gnomAD_HomAlt=0;Best_gnomAD_PopMax_AF=0.001362;Best_gnomAD_Mismatch_ID=gnomAD-SV_v3_DUP_chr4_5a32eae1;Best_gnomAD_Mismatch_OFP=0.206466;Best_gnomAD_Mismatch_SVTYPE=DUP;Best_gnomAD_Mismatch_AF=8e-06;Best_gnomAD_Mismatch_Het=1;Best_gnomAD_Mismatch_HomAlt=0;gnomAD_SV_Cov=1	GT:PSV:LN:DR:ST:QV:TY:ID:RAL:AAL:CO	0/1:NA:1239:20,17:+:1560:DEL:DEL00012561:NA:NA:chr4_19649216-chr4_19650455	0/1:NA:1238:0,0:+:881:DEL:MantaDEL_145217_0_1_0_0_0:NA:NA:chr4_19649216-chr4_19650454	0/1:NA:1237:0,0:+:722:DEL:2587:NA:NA:chr4_19649217-chr4_19650454	split	p15.31	LOC105374511				NR_188378	1	19455417	19937562	1240	0	0	no	4	intron1-intron1	UTR	193563	5'	19649215	19650455																													dbVar	chr4:19081377-19733377;chr4:19561981-19730035;chr4:19609252-19655172;chr4:19634532-19727318	0.01				IMH	4:1330317-92330038;4:4772332-76608755	0.0791																																													-1						full=1	0/1
6_43418273_43418352_DEL_1	6	43418273	43418352	-75	DEL	ILS_AP001,ILS_AP001_1,ILS_AP001_2	MantaDEL:7791:8:8:0:0:0	AAGGAAGGAAGGAAGGAAGGAAGGAAGGAAGGAAGGAAGGAAGGAAGGAAGGAAGGAAGGAAAGAAAGAAAGAAAG	ATA	553	PASS	SUPP=3;SUPP_VEC=111;SVLEN=-75;SVTYPE=DEL;SVMETHOD=SURVIVOR1.0.7;CHR2=6;END=43418352;CIPOS=-4,0;CIEND=-7,0;STRANDS=+;Max_AF=0;Max_Het=0;Max_HomAlt=0;Max_PopMax_AF=0;CCDG_Count=0;Best_CCDG_Mismatch_ID=375664_2;Best_CCDG_Mismatch_OFP=0.0131579;Best_CCDG_Mismatch_SVTYPE=BND;Best_CCDG_Mismatch_AF=0.6983;Best_CCDG_Mismatch_Het=0;Best_CCDG_Mismatch_HomAlt=0;SV_Cov=1;CCDG_SV_Cov=0;TOPMed_Count=0;Best_TOPMed_Mismatch_ID=DUP_6:43339402-43752100;Best_TOPMed_Mismatch_OFP=0.000184153;Best_TOPMed_Mismatch_SVTYPE=DUP;Best_TOPMed_Mismatch_AF=7e-06;Best_TOPMed_Mismatch_Het=2;Best_TOPMed_Mismatch_HomAlt=0;TOPMed_SV_Cov=0;ThousG_Count=0;Best_ThousG_ID=HGSV_132721;Best_ThousG_OFP=0.449045;Best_ThousG_AF=0.512644;Best_ThousG_Het=2786;Best_ThousG_HomAlt=249;Best_ThousG_PopMax_AF=0.571795;Best_ThousG_Mismatch_ID=HGSV_132722;Best_ThousG_Mismatch_OFP=0.0444079;Best_ThousG_Mismatch_SVTYPE=INS;Best_ThousG_Mismatch_AF=0.055626;Best_ThousG_Mismatch_Het=348;Best_ThousG_Mismatch_HomAlt=1;ThousG_SV_Cov=0.776316;gnomAD_Count=0;Best_gnomAD_ID=gnomAD-SV_v3_DEL_chr6_cc44a6bc;Best_gnomAD_OFP=0.459888;Best_gnomAD_AF=0.49725;Best_gnomAD_Het=48660;Best_gnomAD_HomAlt=3694;Best_gnomAD_PopMax_AF=0.569136;gnomAD_Mismatches=gnomAD-SV_v3_DUP_chr6_7545c21e;gnomAD_Mismatches_Count=1;gnomAD_Mismatch_SVTYPEs=DUP;Best_gnomAD_Mismatch_ID=gnomAD-SV_v3_DUP_chr6_7545c21e;Best_gnomAD_Mismatch_OFP=0.818876;Best_gnomAD_Mismatch_SVTYPE=DUP;Best_gnomAD_Mismatch_AF=1.6e-05;Best_gnomAD_Mismatch_Het=2;Best_gnomAD_Mismatch_HomAlt=0;gnomAD_SV_Cov=1	GT:PSV:LN:DR:ST:QV:TY:ID:RAL:AAL:CO	1/1:NA:69:0,0:+:461:DEL:DEL00019591:NA:NA:chr6_43418276-chr6_43418345	1/1:NA:75:0,0:+:553:DEL:MantaDEL_7791_8_8_0_0_0:AAGGAAGGAAGGAAGGAAGGAAGGAAGGAAGGAAGGAAGGAAGGAAGGAAGGAAGGAAGGAAAGAAAGAAAGAAAG:ATA:chr6_43418277-chr6_43418352	1/1:NA:79:0,0:+:472:DEL:3852:NA:NA:chr6_43418273-chr6_43418352	split	p21.1	LOC105375065				XR_001744123	2	43391172	43424730	80	0	0	no	5	intron4-intron4	UTR	831	5'	43418272	43418352																													dbVar	chr6:43418240-43418352	0.01				IMH	6:38040936-64295998	0.0991																																													-1						full=1	1/1
8_48780211_48783201_DEL_1	8	48780211	48783201	-2989	DEL	ILS_AP001,ILS_AP001_1,ILS_AP001_2	MantaDEL:218300:0:1:0:0:0	T		1680	PASS	SUPP=3;SUPP_VEC=111;SVLEN=-2989;SVTYPE=DEL;SVMETHOD=SURVIVOR1.0.7;CHR2=8;END=48783200;CIPOS=0,2;CIEND=0,1;STRANDS=+;Max_AF=0.00843;Max_Het=349;Max_HomAlt=26;Max_PopMax_AF=0.046754;CCDG_Count=1;Best_CCDG_ID=415954;Best_CCDG_OFP=0.999331;Best_CCDG_AF=0.002188;Best_CCDG_Het=0;Best_CCDG_HomAlt=0;Best_CCDG_PopMax_AF=0;Best_CCDG_Mismatch_ID=603900;Best_CCDG_Mismatch_OFP=4.07356e-05;Best_CCDG_Mismatch_SVTYPE=INV;Best_CCDG_Mismatch_AF=0.0006839;Best_CCDG_Mismatch_Het=0;Best_CCDG_Mismatch_HomAlt=0;SV_Cov=1;CCDG_SV_Cov=0.999331;TOPMed_Count=1;Best_TOPMed_ID=DEL_8:48780214-48783203;Best_TOPMed_OFP=0.998329;Best_TOPMed_AF=0.001281;Best_TOPMed_Het=349;Best_TOPMed_HomAlt=6;Best_TOPMed_PopMax_AF=0.0363676;Best_TOPMed_Mismatch_ID=DUP_8:48722331-48892732;Best_TOPMed_Mismatch_OFP=0.0175466;Best_TOPMed_Mismatch_SVTYPE=DUP;Best_TOPMed_Mismatch_AF=7e-06;Best_TOPMed_Mismatch_Het=2;Best_TOPMed_Mismatch_HomAlt=0;TOPMed_SV_Cov=0.999331;ThousG_Count=1;Best_ThousG_ID=HGSV_153702;Best_ThousG_OFP=0.999331;Best_ThousG_AF=0.00843;Best_ThousG_Het=50;Best_ThousG_HomAlt=2;Best_ThousG_PopMax_AF=0.044925;Best_ThousG_Mismatch_ID=HGSV_153347;Best_ThousG_Mismatch_OFP=5.27058e-05;Best_ThousG_Mismatch_SVTYPE=CTX;Best_ThousG_Mismatch_AF=0.000156;Best_ThousG_Mismatch_Het=1;Best_ThousG_Mismatch_HomAlt=0;ThousG_SV_Cov=0.999331;gnomAD_Count=1;Best_gnomAD_ID=gnomAD-SV_v3_DEL_chr8_30e467c1;Best_gnomAD_OFP=0.999331;Best_gnomAD_AF=0.001745;Best_gnomAD_Het=168;Best_gnomAD_HomAlt=26;Best_gnomAD_PopMax_AF=0.046754;Best_gnomAD_Mismatch_ID=gnomAD-SV_v3_DUP_chr8_aab752ee;Best_gnomAD_Mismatch_OFP=0.0175466;Best_gnomAD_Mismatch_SVTYPE=DUP;Best_gnomAD_Mismatch_AF=8e-06;Best_gnomAD_Mismatch_Het=1;Best_gnomAD_Mismatch_HomAlt=0;gnomAD_SV_Cov=1	GT:PSV:LN:DR:ST:QV:TY:ID:RAL:AAL:CO	0/1:NA:2990:23,12:+:1680:DEL:DEL00026148:NA:NA:chr8_48780211-chr8_48783201	0/1:NA:2989:0,0:+:985:DEL:MantaDEL_218300_0_1_0_0_0:NA:NA:chr8_48780211-chr8_48783200	0/1:NA:2987:0,0:+:725:DEL:5120:NA:NA:chr8_48780213-chr8_48783200	split	q11.21	LOC105375825				XR_001745894	2	48780607	48828577	2594	0	0	no	6	txStart-intron1	UTR	166	3'	48780607	48783201																										dbVar	chr8:48708380-49699549	0.01	1000g;dbVar	chr8:48779934-48783395;chr8:48780157-48783358;8:48780158-48783358	0.0419																																																			-1						full=1	0/1
9_7209820_7212300_DEL_1	9	7209820	7212300	-2479	DEL	ILS_AP001,ILS_AP001_1,ILS_AP001_2	MantaDEL:206240:0:1:0:0:0	G		780	PASS	SUPP=3;SUPP_VEC=111;SVLEN=-2479;SVTYPE=DEL;SVMETHOD=SURVIVOR1.0.7;CHR2=9;END=7212299;CIPOS=0,3;CIEND=0,1;STRANDS=+;Max_AF=0.000468;Max_Het=34;Max_HomAlt=2;Max_PopMax_AF=0.002951;CCDG_Count=1;Best_CCDG_ID=426586;Best_CCDG_OFP=0.99879;Best_CCDG_AF=0.0001368;Best_CCDG_Het=0;Best_CCDG_HomAlt=0;Best_CCDG_PopMax_AF=0;SV_Cov=1;CCDG_SV_Cov=1;TOPMed_Count=1;Best_TOPMed_ID=DEL_9:7209824-7212299;Best_TOPMed_OFP=0.99879;Best_TOPMed_AF=0.000121;Best_TOPMed_Het=34;Best_TOPMed_HomAlt=0;Best_TOPMed_PopMax_AF=0.00291545;Best_TOPMed_Mismatch_ID=DUP_9:6991180-7215651;Best_TOPMed_Mismatch_OFP=0.0110481;Best_TOPMed_Mismatch_SVTYPE=DUP;Best_TOPMed_Mismatch_AF=1.1e-05;Best_TOPMed_Mismatch_Het=3;Best_TOPMed_Mismatch_HomAlt=0;TOPMed_SV_Cov=1;ThousG_Count=1;Best_ThousG_ID=HGSV_159767;Best_ThousG_OFP=0.99879;Best_ThousG_AF=0.000468;Best_ThousG_Het=1;Best_ThousG_HomAlt=1;Best_ThousG_PopMax_AF=0.002496;ThousG_SV_Cov=0.99879;gnomAD_Count=1;Best_gnomAD_ID=gnomAD-SV_v3_DEL_chr9_1d907117;Best_gnomAD_OFP=0.995584;Best_gnomAD_AF=0.000127;Best_gnomAD_Het=12;Best_gnomAD_HomAlt=2;Best_gnomAD_PopMax_AF=0.002951;Best_gnomAD_Mismatch_ID=gnomAD-SV_v3_DUP_chr9_d8f0accb;Best_gnomAD_Mismatch_OFP=0.0110481;Best_gnomAD_Mismatch_SVTYPE=DUP;Best_gnomAD_Mismatch_AF=8e-06;Best_gnomAD_Mismatch_Het=1;Best_gnomAD_Mismatch_HomAlt=0;gnomAD_SV_Cov=1	GT:PSV:LN:DR:ST:QV:TY:ID:RAL:AAL:CO	0/1:NA:2480:17,6:+:780:DEL:DEL00027669:NA:NA:chr9_7209820-chr9_7212300	0/1:NA:2479:0,0:+:322:DEL:MantaDEL_206240_0_1_0_0_0:NA:NA:chr9_7209820-chr9_7212299	0/1:NA:2476:0,0:+:197:DEL:5450:NA:NA:chr9_7209823-chr9_7212299	split	p24.1	LOC105375969				XR_007061413	1	7202335	7210505	686	0	0	no	3	exon3-txEnd	UTR	903	3'	7209819	7210505																																																																																		-1						full=3	0/1
10_2581858_2636673_DUP_1	10	2581858	2636673	54815	DUP	ILS_AP001,ILS_AP001_1,ILS_AP001_2	MantaDUP:TANDEM:36455:0:1:0:0:0	T		660	PASS	SUPP=3;SUPP_VEC=111;SVLEN=54815;SVTYPE=DUP;SVMETHOD=SURVIVOR1.0.7;CHR2=10;END=2636673;CIPOS=0,1;CIEND=0,0;STRANDS=-;Max_AF=0;Max_Het=0;Max_HomAlt=0;Max_PopMax_AF=0;CCDG_Count=0;Best_CCDG_ID=461358;Best_CCDG_OFP=0.390488;Best_CCDG_AF=6.839e-05;Best_CCDG_Het=0;Best_CCDG_HomAlt=0;Best_CCDG_PopMax_AF=0;Best_CCDG_Mismatch_ID=144152;Best_CCDG_Mismatch_OFP=0.21979;Best_CCDG_Mismatch_SVTYPE=DEL;Best_CCDG_Mismatch_AF=6.839e-05;Best_CCDG_Mismatch_Het=0;Best_CCDG_Mismatch_HomAlt=0;SV_Cov=1;CCDG_SV_Cov=1;TOPMed_Count=0;Best_TOPMed_ID=DUP_10:2600430-2645536;Best_TOPMed_OFP=0.531294;Best_TOPMed_AF=1.1e-05;Best_TOPMed_Het=3;Best_TOPMed_HomAlt=0;Best_TOPMed_PopMax_AF=3.61185e-05;Best_TOPMed_Mismatch_ID=DEL_10:2542693-2659833;Best_TOPMed_Mismatch_OFP=0.467945;Best_TOPMed_Mismatch_SVTYPE=DEL;Best_TOPMed_Mismatch_AF=4e-06;Best_TOPMed_Mismatch_Het=1;Best_TOPMed_Mismatch_HomAlt=0;TOPMed_SV_Cov=1;ThousG_Count=0;Best_ThousG_ID=HGSV_13339;Best_ThousG_OFP=0.0167112;Best_ThousG_AF=0.000156;Best_ThousG_Het=1;Best_ThousG_HomAlt=0;Best_ThousG_PopMax_AF=0.000855;Best_ThousG_Mismatch_ID=HGSV_13340;Best_ThousG_Mismatch_OFP=0.221651;Best_ThousG_Mismatch_SVTYPE=CPX;Best_ThousG_Mismatch_AF=0.000156;Best_ThousG_Mismatch_Het=1;Best_ThousG_Mismatch_HomAlt=0;ThousG_SV_Cov=0.417615;gnomAD_Count=0;Best_gnomAD_ID=gnomAD-SV_v3_DUP_chr10_e1dac703;Best_gnomAD_OFP=0.531294;Best_gnomAD_AF=8e-06;Best_gnomAD_Het=1;Best_gnomAD_HomAlt=0;Best_gnomAD_PopMax_AF=3e-05;Best_gnomAD_Mismatch_ID=gnomAD-SV_v3_CPX_chr10_d7e99c97;Best_gnomAD_Mismatch_OFP=0.221195;Best_gnomAD_Mismatch_SVTYPE=CPX;Best_gnomAD_Mismatch_AF=4.8e-05;Best_gnomAD_Mismatch_Het=6;Best_gnomAD_Mismatch_HomAlt=0;gnomAD_SV_Cov=1	GT:PSV:LN:DR:ST:QV:TY:ID:RAL:AAL:CO	0/1:NA:54814:22,3:-:660:DUP:DUP00030026:NA:NA:chr10_2581859-chr10_2636673	0/1:NA:54815:0,0:-:530:DUP:MantaDUP_TANDEM_36455_0_1_0_0_0:NA:NA:chr10_2581858-chr10_2636673	0/1:NA:54815:0,0:-:122:DUP:5867:NA:NA:chr10_2581858-chr10_2636673	split	p15.3	LOC105376350				NR_188171	1	2501852	2618741	36884	0	0	no	6	intron4-txEnd	UTR	11592	5'	2581857	2618741																										dbVar	chr10:2357805-2763808;chr10:2460844-3257750	0.01	dbVar	chr10:2505807-3017808	0.01																																																			-1						full=3	0/1
10_2580097_2636532_DEL_1	10	2580097	2636532	-56434	DEL	ILS_AP001,ILS_AP001_1,ILS_AP001_2	MantaDEL:36411:0:1:0:0:0	A		1260	PASS	SUPP=3;SUPP_VEC=111;SVLEN=-56434;SVTYPE=DEL;SVMETHOD=SURVIVOR1.0.7;CHR2=10;END=2636531;CIPOS=0,1;CIEND=0,1;STRANDS=+;Max_AF=0;Max_Het=0;Max_HomAlt=0;Max_PopMax_AF=0;CCDG_Count=0;Best_CCDG_ID=144152;Best_CCDG_OFP=0.213485;Best_CCDG_AF=6.839e-05;Best_CCDG_Het=0;Best_CCDG_HomAlt=0;Best_CCDG_PopMax_AF=0;Best_CCDG_Mismatch_ID=461358;Best_CCDG_Mismatch_OFP=0.379286;Best_CCDG_Mismatch_SVTYPE=DUP;Best_CCDG_Mismatch_AF=6.839e-05;Best_CCDG_Mismatch_Het=0;Best_CCDG_Mismatch_HomAlt=0;SV_Cov=1;CCDG_SV_Cov=0.415522;TOPMed_Count=0;Best_TOPMed_ID=DEL_10:2542693-2659833;Best_TOPMed_OFP=0.481766;Best_TOPMed_AF=4e-06;Best_TOPMed_Het=1;Best_TOPMed_HomAlt=0;Best_TOPMed_PopMax_AF=0.00010879;Best_TOPMed_Mismatch_ID=DUP_10:2600430-2645536;Best_TOPMed_Mismatch_OFP=0.512017;Best_TOPMed_Mismatch_SVTYPE=DUP;Best_TOPMed_Mismatch_AF=1.1e-05;Best_TOPMed_Mismatch_Het=3;Best_TOPMed_Mismatch_HomAlt=0;TOPMed_SV_Cov=1;ThousG_Count=0;Best_ThousG_ID=HGSV_13341;Best_ThousG_OFP=0.213502;Best_ThousG_AF=0.000156;Best_ThousG_Het=1;Best_ThousG_HomAlt=0;Best_ThousG_PopMax_AF=0;Best_ThousG_Mismatch_ID=HGSV_13340;Best_ThousG_Mismatch_OFP=0.215292;Best_ThousG_Mismatch_SVTYPE=CPX;Best_ThousG_Mismatch_AF=0.000156;Best_ThousG_Mismatch_Het=1;Best_ThousG_Mismatch_HomAlt=0;ThousG_SV_Cov=0.508869;gnomAD_Count=0;Best_gnomAD_ID=gnomAD-SV_v3_DEL_chr10_e348455b;Best_gnomAD_OFP=0.198069;Best_gnomAD_AF=0.000325;Best_gnomAD_Het=41;Best_gnomAD_HomAlt=0;Best_gnomAD_PopMax_AF=0.001572;Best_gnomAD_Mismatch_ID=gnomAD-SV_v3_DUP_chr10_e1dac703;Best_gnomAD_Mismatch_OFP=0.512017;Best_gnomAD_Mismatch_SVTYPE=DUP;Best_gnomAD_Mismatch_AF=8e-06;Best_gnomAD_Mismatch_Het=1;Best_gnomAD_Mismatch_HomAlt=0;gnomAD_SV_Cov=1	GT:PSV:LN:DR:ST:QV:TY:ID:RAL:AAL:CO	0/1:NA:56435:30,13:+:1260:DEL:DEL00030025:NA:NA:chr10_2580097-chr10_2636532	0/1:NA:56434:0,0:+:779:DEL:MantaDEL_36411_0_1_0_0_0:NA:NA:chr10_2580097-chr10_2636531	0/1:NA:56433:0,0:+:479:DEL:5866:NA:NA:chr10_2580098-chr10_2636531	split	p15.3	LOC105376350				NR_188171	1	2501852	2618741	38645	0	0	no	6	intron4-txEnd	UTR	9831	5'	2580096	2618741																										dbVar	chr10:2357805-2763808;chr10:2460844-3257750	0.01	dbVar	chr10:2505807-3017808	0.01																																																			-1						full=1	0/1
1_19035733_19044940_DEL_1	1	19035733	19044940	-9206	DEL	ILS_AP001,ILS_AP001_1,ILS_AP001_2	MantaDEL:189990:0:1:0:0:0	T		720	PASS	SUPP=3;SUPP_VEC=111;SVLEN=-9206;SVTYPE=DEL;SVMETHOD=SURVIVOR1.0.7;CHR2=1;END=19044939;CIPOS=0,2;CIEND=0,1;STRANDS=+;Max_AF=0.004527;Max_Het=162;Max_HomAlt=2;Max_PopMax_AF=0.023295;CCDG_Count=0;Best_CCDG_ID=122441;Best_CCDG_OFP=0.031339;Best_CCDG_AF=0.0003078;Best_CCDG_Het=0;Best_CCDG_HomAlt=0;Best_CCDG_PopMax_AF=0;Best_CCDG_Mismatch_ID=543252;Best_CCDG_Mismatch_OFP=0.000215224;Best_CCDG_Mismatch_SVTYPE=INV;Best_CCDG_Mismatch_AF=0.01679;Best_CCDG_Mismatch_Het=0;Best_CCDG_Mismatch_HomAlt=0;SV_Cov=1;CCDG_SV_Cov=0.0326925;TOPMed_Count=2;Best_TOPMed_ID=DEL_1:19035736-19044942;Best_TOPMed_OFP=0.999457;Best_TOPMed_AF=0.000589;Best_TOPMed_Het=162;Best_TOPMed_HomAlt=2;Best_TOPMed_PopMax_AF=0.0173014;Best_TOPMed_Mismatch_ID=DUP_1:18981802-19060600;Best_TOPMed_Mismatch_OFP=0.11684;Best_TOPMed_Mismatch_SVTYPE=DUP;Best_TOPMed_Mismatch_AF=1e-05;Best_TOPMed_Mismatch_Het=2;Best_TOPMed_Mismatch_HomAlt=0;TOPMed_SV_Cov=1;ThousG_Count=1;Best_ThousG_ID=HGSV_1365;Best_ThousG_OFP=0.999783;Best_ThousG_AF=0.004527;Best_ThousG_Het=29;Best_ThousG_HomAlt=0;Best_ThousG_PopMax_AF=0.023295;ThousG_SV_Cov=0.999783;gnomAD_Count=2;Best_gnomAD_ID=gnomAD-SV_v3_DEL_chr1_f6c129cb;Best_gnomAD_OFP=0.998264;Best_gnomAD_AF=5.6e-05;Best_gnomAD_Het=7;Best_gnomAD_HomAlt=0;Best_gnomAD_PopMax_AF=0.001589;Best_gnomAD_Mismatch_ID=gnomAD-SV_v3_INS_chr1_d65cf9e9;Best_gnomAD_Mismatch_OFP=0.0016292;Best_gnomAD_Mismatch_SVTYPE=INS;Best_gnomAD_Mismatch_AF=0.000841;Best_gnomAD_Mismatch_Het=106;Best_gnomAD_Mismatch_HomAlt=0;gnomAD_SV_Cov=1	GT:PSV:LN:DR:ST:QV:TY:ID:RAL:AAL:CO	0/1:NA:9207:12,7:+:720:DEL:DEL00000764:NA:NA:chr1_19035733-chr1_19044940	0/1:NA:9206:0,0:+:318:DEL:MantaDEL_189990_0_1_0_0_0:NA:NA:chr1_19035733-chr1_19044939	0/1:NA:9204:0,0:+:275:DEL:150:NA:NA:chr1_19035735-chr1_19044939	split	p36.13	LOC105376815				XR_947015	2	18970477	19051428	9208	0	0	no	3	intron1-intron1	UTR	5937	5'	19035732	19044940																													1000g;dbVar;gnomAD-SV_v3_DEL_chr1_cf5f2df8	chr1:13203398-167621949;chr1:19035658-19045037;1:19035659-19045037	0.1676				IMH	1:7601938-50381771;1:10187873-186857210;1:16798094-234784887	0.4698																																													-1						full=1	0/1
14_48882017_49020572_DUP_1	14	48882017	49020572	138551	DUP	ILS_AP001,ILS_AP001_1,ILS_AP001_2	MantaDUP:TANDEM:73635:0:1:0:0:0	C		2007	PASS	SUPP=3;SUPP_VEC=111;SVLEN=138551;SVTYPE=DUP;SVMETHOD=SURVIVOR1.0.7;CHR2=14;END=49020572;CIPOS=-4,0;CIEND=0,0;STRANDS=-;Max_AF=0;Max_Het=0;Max_HomAlt=0;Max_PopMax_AF=0;CCDG_Count=0;Best_CCDG_ID=477632;Best_CCDG_OFP=0.0752779;Best_CCDG_AF=3.419e-05;Best_CCDG_Het=0;Best_CCDG_HomAlt=0;Best_CCDG_PopMax_AF=0;Best_CCDG_Mismatch_ID=204462;Best_CCDG_Mismatch_OFP=0.493609;Best_CCDG_Mismatch_SVTYPE=DEL;Best_CCDG_Mismatch_AF=3.419e-05;Best_CCDG_Mismatch_Het=0;Best_CCDG_Mismatch_HomAlt=0;SV_Cov=1;CCDG_SV_Cov=0.282233;TOPMed_Count=0;Best_TOPMed_ID=DUP_14:48916068-48955171;Best_TOPMed_OFP=0.282241;Best_TOPMed_AF=4e-06;Best_TOPMed_Het=1;Best_TOPMed_HomAlt=0;Best_TOPMed_PopMax_AF=7.29714e-05;Best_TOPMed_Mismatch_ID=DEL_14:48939002-49018200;Best_TOPMed_Mismatch_OFP=0.571627;Best_TOPMed_Mismatch_SVTYPE=DEL;Best_TOPMed_Mismatch_AF=0.000811;Best_TOPMed_Mismatch_Het=206;Best_TOPMed_Mismatch_HomAlt=0;TOPMed_SV_Cov=1;ThousG_Count=0;Best_ThousG_Mismatch_ID=HGSV_45304;Best_ThousG_Mismatch_OFP=0.168825;Best_ThousG_Mismatch_SVTYPE=DEL;Best_ThousG_Mismatch_AF=0.000312;Best_ThousG_Mismatch_Het=2;Best_ThousG_Mismatch_HomAlt=0;ThousG_SV_Cov=0;gnomAD_Count=0;Best_gnomAD_ID=gnomAD-SV_v3_DUP_chr14_cae0226a;Best_gnomAD_OFP=0.282241;Best_gnomAD_AF=2.4e-05;Best_gnomAD_Het=3;Best_gnomAD_HomAlt=0;Best_gnomAD_PopMax_AF=0.000493;Best_gnomAD_Mismatch_ID=gnomAD-SV_v3_DEL_chr14_0e69a72e;Best_gnomAD_Mismatch_OFP=0.493609;Best_gnomAD_Mismatch_SVTYPE=DEL;Best_gnomAD_Mismatch_AF=4e-05;Best_gnomAD_Mismatch_Het=5;Best_gnomAD_Mismatch_HomAlt=0;gnomAD_SV_Cov=1	GT:PSV:LN:DR:ST:QV:TY:ID:RAL:AAL:CO	0/1:NA:138555:18,18:-:2007:DUP:DUP00042067:NA:NA:chr14_48882017-chr14_49020572	0/1:NA:138551:0,0:-:999:DUP:MantaDUP_TANDEM_73635_0_1_0_0_0:NA:NA:chr14_48882021-chr14_49020572	1/1:NA:138551:0,0:-:658:DUP:8005:NA:NA:chr14_48882021-chr14_49020572	split	q21.3	LOC105378178				XR_007064152	1	48393998	49288023	138556	0	0	no	10	intron4-intron4	UTR	49219	5'	48882016	49020572																										dbVar	chr14:48646756-49611813	0.01	dbVar;gnomAD-SV_v3_DEL_chr14_040215df	chr14:41140072-69277618;chr14:48768510-49049201	0.0745				IMH	14:23629193-97505158;14:46648665-62759566;14:46648974-62759579	0.4022																																													-1						full=3	1/1
1_185620013_185620102_DEL_1	1	1.86E+08	1.86E+08	-88	DEL	ILS_AP001,ILS_AP001_1,ILS_AP001_2	MantaDEL:200913:0:1:0:0:0	AATGTGTGTATATATATATGGAATCTTCATATATGTGTATATATATATATACACATATATGAAGATTCCATATATATATACACACACAT	A	239	PASS	SUPP=3;SUPP_VEC=111;SVLEN=-88;SVTYPE=DEL;SVMETHOD=SURVIVOR1.0.7;CHR2=1;END=185620101;CIPOS=0,14;CIEND=0,1;STRANDS=+;Max_AF=4.8e-05;Max_Het=6;Max_HomAlt=0;Max_PopMax_AF=0.000247;CCDG_Count=0;Best_CCDG_ID=137217;Best_CCDG_OFP=0.00985931;Best_CCDG_AF=3.419e-05;Best_CCDG_Het=0;Best_CCDG_HomAlt=0;Best_CCDG_PopMax_AF=0;Best_CCDG_Mismatch_ID=137220_1;Best_CCDG_Mismatch_OFP=0.011236;Best_CCDG_Mismatch_SVTYPE=BND;Best_CCDG_Mismatch_AF=0.06291;Best_CCDG_Mismatch_Het=0;Best_CCDG_Mismatch_HomAlt=0;SV_Cov=1;CCDG_SV_Cov=1;TOPMed_Count=0;Best_TOPMed_ID=DEL_1:185617022-185620071;Best_TOPMed_OFP=0.0128195;Best_TOPMed_AF=4e-06;Best_TOPMed_Het=1;Best_TOPMed_HomAlt=0;Best_TOPMed_PopMax_AF=6.26786e-06;Best_TOPMed_Mismatch_ID=INV_1:183856810-187909288;Best_TOPMed_Mismatch_OFP=2.19619e-05;Best_TOPMed_Mismatch_SVTYPE=INV;Best_TOPMed_Mismatch_AF=4e-06;Best_TOPMed_Mismatch_Het=1;Best_TOPMed_Mismatch_HomAlt=0;TOPMed_SV_Cov=0.662921;ThousG_Count=0;Best_ThousG_Mismatch_ID=HGSV_9278;Best_ThousG_Mismatch_OFP=0.418323;Best_ThousG_Mismatch_SVTYPE=INS;Best_ThousG_Mismatch_AF=0.000625;Best_ThousG_Mismatch_Het=4;Best_ThousG_Mismatch_HomAlt=0;ThousG_SV_Cov=0;gnomAD_Count=2;Best_gnomAD_ID=gnomAD-SV_v3_DEL_chr1_91caac2e;Best_gnomAD_OFP=0.842697;Best_gnomAD_AF=3.3e-05;Best_gnomAD_Het=4;Best_gnomAD_HomAlt=0;Best_gnomAD_PopMax_AF=5.1e-05;Best_gnomAD_Mismatch_ID=gnomAD-SV_v3_INS_chr1_5eaa9906;Best_gnomAD_Mismatch_OFP=0.494382;Best_gnomAD_Mismatch_SVTYPE=INS;Best_gnomAD_Mismatch_AF=0.004189;Best_gnomAD_Mismatch_Het=524;Best_gnomAD_Mismatch_HomAlt=2;gnomAD_SV_Cov=1	GT:PSV:LN:DR:ST:QV:TY:ID:RAL:AAL:CO	0/1:NA:89:0,0:+:239:DEL:DEL00004115:NA:NA:chr1_185620013-chr1_185620102	0/1:NA:88:0,0:+:139:DEL:MantaDEL_200913_0_1_0_0_0:AATGTGTGTATATATATATGGAATCTTCATATATGTGTATATATATATATACACATATATGAAGATTCCATATATATATACACACACAT:A:chr1_185620013-chr1_185620101	0/1:NA:74:0,0:+:83:DEL:818:NA:NA:chr1_185620027-chr1_185620101	split	q25.3	LOC107985239				XR_001738340	2	1.85E+08	1.86E+08	90	0	0	no	3	intron1-intron1	UTR	59346	3'	1.86E+08	1.86E+08																													gnomAD-SV_v3_DEL_chr1_339fd21d	chr1:168349020-187167586	0.9262				IMH	1:10187873-186857210;1:16798094-234784887;1:68663392-188869781;1:107555343-232824950	0.4698																																													-1						full=1	0/1
4_58562829_58569632_DUP_1	4	58562829	58569632	6800	DUP	ILS_AP001,ILS_AP001_1,ILS_AP001_2	MantaDUP:TANDEM:148111:0:1:1:1:0	T		1440	PASS	SUPP=3;SUPP_VEC=111;SVLEN=6800;SVTYPE=DUP;SVMETHOD=SURVIVOR1.0.7;CHR2=4;END=58569632;CIPOS=-3,0;CIEND=-1,0;STRANDS=-;Max_AF=0;Max_Het=0;Max_HomAlt=0;Max_PopMax_AF=0;CCDG_Count=0;Best_CCDG_Mismatch_ID=586980;Best_CCDG_Mismatch_OFP=0.000206405;Best_CCDG_Mismatch_SVTYPE=INV;Best_CCDG_Mismatch_AF=0.004513;Best_CCDG_Mismatch_Het=0;Best_CCDG_Mismatch_HomAlt=0;SV_Cov=1;CCDG_SV_Cov=0;TOPMed_Count=0;Best_TOPMed_ID=DUP_4:58560946-58570336;Best_TOPMed_OFP=0.724127;Best_TOPMed_AF=7e-06;Best_TOPMed_Het=0;Best_TOPMed_HomAlt=1;Best_TOPMed_PopMax_AF=0.000218007;Best_TOPMed_Mismatch_ID=INV_4:57645482-58575877;Best_TOPMed_Mismatch_OFP=0.00730978;Best_TOPMed_Mismatch_SVTYPE=INV;Best_TOPMed_Mismatch_AF=4e-06;Best_TOPMed_Mismatch_Het=1;Best_TOPMed_Mismatch_HomAlt=0;TOPMed_SV_Cov=1;ThousG_Count=0;Best_ThousG_ID=HGSV_111856;Best_ThousG_OFP=0.0024321;Best_ThousG_AF=0.000156;Best_ThousG_Het=1;Best_ThousG_HomAlt=0;Best_ThousG_PopMax_AF=0.000855;Best_ThousG_Mismatch_ID=HGSV_111855;Best_ThousG_Mismatch_OFP=0.000147037;Best_ThousG_Mismatch_SVTYPE=INS;Best_ThousG_Mismatch_AF=0.15029;Best_ThousG_Mismatch_Het=0;Best_ThousG_Mismatch_HomAlt=0;ThousG_SV_Cov=0.169534;gnomAD_Count=0;Best_gnomAD_ID=gnomAD-SV_v3_DUP_chr4_41308de1;Best_gnomAD_OFP=0.0382297;Best_gnomAD_AF=0.030721;Best_gnomAD_Het=1874;Best_gnomAD_HomAlt=276;Best_gnomAD_PopMax_AF=0.162393;gnomAD_Mismatches=gnomAD-SV_v3_DEL_chr4_a828013e;gnomAD_Mismatches_Count=1;gnomAD_Mismatch_SVTYPEs=DEL;Best_gnomAD_Mismatch_ID=gnomAD-SV_v3_DEL_chr4_a828013e;Best_gnomAD_Mismatch_OFP=0.843692;Best_gnomAD_Mismatch_SVTYPE=DEL;Best_gnomAD_Mismatch_AF=1.6e-05;Best_gnomAD_Mismatch_Het=2;Best_gnomAD_Mismatch_HomAlt=0;gnomAD_SV_Cov=1	GT:PSV:LN:DR:ST:QV:TY:ID:RAL:AAL:CO	0/1:NA:6802:25,13:-:1440:DUP:DUP00014346:NA:NA:chr4_58562829-chr4_58569631	0/1:NA:6800:0,0:-:780:DUP:MantaDUP_TANDEM_148111_0_1_1_1_0:NA:NA:chr4_58562832-chr4_58569632	1/1:NA:6800:0,0:-:305:DUP:2704:NA:NA:chr4_58562832-chr4_58569632	split	q13.1	LOC107986228				XR_001741512	1	58567286	58590243	2346	0	0	no	4	txStart-intron1	UTR	1952	5'	58567286	58569632																										dbVar	chr4:57322428-61871079	0.01							IMH	4:1330317-92330038;4:4772332-76608755;4:21291890-139485605;4:21757282-83521389;4:32217847-78249129;4:41369891-94869199	0.1882																																													-1						full=3	1/1
4_58562830_58569374_DEL_1	4	58562830	58569374	-6543	DEL	ILS_AP001,ILS_AP001_1,ILS_AP001_2	MantaDEL:148111:0:1:0:0:0	G		1680	PASS	SUPP=3;SUPP_VEC=111;SVLEN=-6543;SVTYPE=DEL;SVMETHOD=SURVIVOR1.0.7;CHR2=4;END=58569373;CIPOS=0,4;CIEND=0,1;STRANDS=+;Max_AF=1.6e-05;Max_Het=2;Max_HomAlt=0;Max_PopMax_AF=0.000247;CCDG_Count=0;Best_CCDG_Mismatch_ID=586980;Best_CCDG_Mismatch_OFP=0.000198605;Best_CCDG_Mismatch_SVTYPE=INV;Best_CCDG_Mismatch_AF=0.004513;Best_CCDG_Mismatch_Het=0;Best_CCDG_Mismatch_HomAlt=0;SV_Cov=1;CCDG_SV_Cov=0;TOPMed_Count=0;Best_TOPMed_ID=DEL_4:58042412-59020458;Best_TOPMed_OFP=0.00669088;Best_TOPMed_AF=4e-06;Best_TOPMed_Het=1;Best_TOPMed_HomAlt=0;Best_TOPMed_PopMax_AF=6.26025e-06;Best_TOPMed_Mismatch_ID=DUP_4:58560946-58570336;Best_TOPMed_Mismatch_OFP=0.696763;Best_TOPMed_Mismatch_SVTYPE=DUP;Best_TOPMed_Mismatch_AF=7e-06;Best_TOPMed_Mismatch_Het=0;Best_TOPMed_Mismatch_HomAlt=1;TOPMed_SV_Cov=1;ThousG_Count=0;Best_ThousG_Mismatch_ID=HGSV_111856;Best_ThousG_Mismatch_OFP=0.00151959;Best_ThousG_Mismatch_SVTYPE=DUP;Best_ThousG_Mismatch_AF=0.000156;Best_ThousG_Mismatch_Het=1;Best_ThousG_Mismatch_HomAlt=0;ThousG_SV_Cov=0;gnomAD_Count=1;Best_gnomAD_ID=gnomAD-SV_v3_DEL_chr4_a828013e;Best_gnomAD_OFP=0.81181;Best_gnomAD_AF=1.6e-05;Best_gnomAD_Het=2;Best_gnomAD_HomAlt=0;Best_gnomAD_PopMax_AF=0.000247;Best_gnomAD_Mismatch_ID=gnomAD-SV_v3_DUP_chr4_e4405b6f;Best_gnomAD_Mismatch_OFP=0.0265892;Best_gnomAD_Mismatch_SVTYPE=DUP;Best_gnomAD_Mismatch_AF=0.083682;Best_gnomAD_Mismatch_Het=9593;Best_gnomAD_Mismatch_HomAlt=0;gnomAD_SV_Cov=1	GT:PSV:LN:DR:ST:QV:TY:ID:RAL:AAL:CO	0/1:NA:6544:26,17:+:1680:DEL:DEL00014347:NA:NA:chr4_58562830-chr4_58569374	0/1:NA:6543:0,0:+:754:DEL:MantaDEL_148111_0_1_0_0_0:NA:NA:chr4_58562830-chr4_58569373	0/1:NA:6539:0,0:+:737:DEL:2703:NA:NA:chr4_58562834-chr4_58569373	split	q13.1	LOC107986228				XR_001741512	1	58567286	58590243	2088	0	0	no	4	txStart-intron1	UTR	1694	5'	58567286	58569374																										dbVar	chr4:57322428-61871079	0.01							IMH	4:1330317-92330038;4:4772332-76608755;4:21291890-139485605;4:21757282-83521389;4:32217847-78249129;4:41369891-94869199	0.1882																																													-1						full=3	0/1
6_169981355_169981464_DEL_1	6	1.7E+08	1.7E+08	-108	DEL	ILS_AP001,ILS_AP001_1,ILS_AP001_2	MantaDEL:131153:0:1:0:0:0	GTCACTGAACCTGGAGGGGGCTCAGGGTCCTCACTGCTCACTGAACCTGGAGGGGGCTCAGGATCCTCACTGCTCACTGAGGCTGGAGGGGGCTCAGGGTCCTCAAAGC	G	240	PASS	SUPP=3;SUPP_VEC=111;SVLEN=-108;SVTYPE=DEL;SVMETHOD=SURVIVOR1.0.7;CHR2=6;END=169981463;CIPOS=0,40;CIEND=0,1;STRANDS=+;Max_AF=0;Max_Het=0;Max_HomAlt=0;Max_PopMax_AF=0;CCDG_Count=0;Best_CCDG_ID=388275;Best_CCDG_OFP=0.633027;Best_CCDG_AF=0.2137;Best_CCDG_Het=0;Best_CCDG_HomAlt=0;Best_CCDG_PopMax_AF=0;SV_Cov=1;CCDG_SV_Cov=1;TOPMed_Count=0;Best_TOPMed_ID=DEL_6:169981396-169981504;Best_TOPMed_OFP=0.397081;Best_TOPMed_AF=0.176012;Best_TOPMed_Het=39469;Best_TOPMed_HomAlt=188;Best_TOPMed_PopMax_AF=0.409962;Best_TOPMed_Mismatch_ID=INV_6:169946302-169997872;Best_TOPMed_Mismatch_OFP=0.00211355;Best_TOPMed_Mismatch_SVTYPE=INV;Best_TOPMed_Mismatch_AF=4e-06;Best_TOPMed_Mismatch_Het=1;Best_TOPMed_Mismatch_HomAlt=0;TOPMed_SV_Cov=1;ThousG_Count=0;Best_ThousG_ID=HGSV_139953;Best_ThousG_OFP=0.633027;Best_ThousG_AF=0.306588;Best_ThousG_Het=1332;Best_ThousG_HomAlt=316;Best_ThousG_PopMax_AF=0.54188;Best_ThousG_Mismatch_ID=HGSV_139951;Best_ThousG_Mismatch_OFP=0.00939979;Best_ThousG_Mismatch_SVTYPE=DUP;Best_ThousG_Mismatch_AF=0.000156;Best_ThousG_Mismatch_Het=1;Best_ThousG_Mismatch_HomAlt=0;ThousG_SV_Cov=0.633027;gnomAD_Count=0;Best_gnomAD_ID=gnomAD-SV_v3_DEL_chr6_773e44d8;Best_gnomAD_OFP=0.201479;Best_gnomAD_AF=0.168424;Best_gnomAD_Het=20073;Best_gnomAD_HomAlt=535;Best_gnomAD_PopMax_AF=0.449405;Best_gnomAD_Mismatch_ID=gnomAD-SV_v3_INS_chr6_0cc115b6;Best_gnomAD_Mismatch_OFP=0.550459;Best_gnomAD_Mismatch_SVTYPE=INS;Best_gnomAD_Mismatch_AF=0.062465;Best_gnomAD_Mismatch_Het=5871;Best_gnomAD_Mismatch_HomAlt=2;gnomAD_SV_Cov=1	GT:PSV:LN:DR:ST:QV:TY:ID:RAL:AAL:CO	0/1:NA:109:0,0:+:240:DEL:DEL00021316:NA:NA:chr6_169981355-chr6_169981464	0/1:NA:108:0,0:+:227:DEL:MantaDEL_131153_0_1_0_0_0:GTCACTGAACCTGGAGGGGGCTCAGGGTCCTCACTGCTCACTGAACCTGGAGGGGGCTCAGGATCCTCACTGCTCACTGAGGCTGGAGGGGGCTCAGGGTCCTCAAAGC:G:chr6_169981355-chr6_169981463	0/1:NA:68:0,0:+:86:DEL:4219:NA:NA:chr6_169981395-chr6_169981463	split	q27	LOC107986675				XR_001744484	3	1.7E+08	1.7E+08	110	0	0	no	2	exon2-exon2	UTR	1609	3'	1.7E+08	1.7E+08																																																																																		-1						full=3	0/1
9_110267689_110275007_DUP_1	9	1.1E+08	1.1E+08	7316	DUP	ILS_AP001,ILS_AP001_1,ILS_AP001_2	MantaDUP:TANDEM:211865:1:2:0:0:0	T		480	PASS	SUPP=3;SUPP_VEC=111;SVLEN=7316;SVTYPE=DUP;SVMETHOD=SURVIVOR1.0.7;CHR2=9;END=110275005;CIPOS=0,1;CIEND=0,2;STRANDS=-;Max_AF=8e-06;Max_Het=1;Max_HomAlt=0;Max_PopMax_AF=0.000229;CCDG_Count=0;Best_CCDG_Mismatch_ID=434989;Best_CCDG_Mismatch_OFP=0.554245;Best_CCDG_Mismatch_SVTYPE=DEL;Best_CCDG_Mismatch_AF=0.1392;Best_CCDG_Mismatch_Het=0;Best_CCDG_Mismatch_HomAlt=0;SV_Cov=1;CCDG_SV_Cov=0;TOPMed_Count=0;Best_TOPMed_Mismatch_ID=DEL_9:110266596-110270066;Best_TOPMed_Mismatch_OFP=0.222593;Best_TOPMed_Mismatch_SVTYPE=DEL;Best_TOPMed_Mismatch_AF=4e-06;Best_TOPMed_Mismatch_Het=1;Best_TOPMed_Mismatch_HomAlt=0;TOPMed_SV_Cov=0;ThousG_Count=0;Best_ThousG_ID=HGSV_164584;Best_ThousG_OFP=0.0261064;Best_ThousG_AF=0.265436;Best_ThousG_Het=808;Best_ThousG_HomAlt=430;Best_ThousG_PopMax_AF=0.613074;Best_ThousG_Mismatch_ID=HGSV_164582;Best_ThousG_Mismatch_OFP=2.5337e-08;Best_ThousG_Mismatch_SVTYPE=CPX;Best_ThousG_Mismatch_AF=0.228224;Best_ThousG_Mismatch_Het=1136;Best_ThousG_Mismatch_HomAlt=163;ThousG_SV_Cov=1;gnomAD_Count=1;Best_gnomAD_ID=gnomAD-SV_v3_DUP_chr9_e71fd71d;Best_gnomAD_OFP=0.820145;Best_gnomAD_AF=8e-06;Best_gnomAD_Het=1;Best_gnomAD_HomAlt=0;Best_gnomAD_PopMax_AF=0.000229;Best_gnomAD_Mismatch_ID=gnomAD-SV_v3_DEL_chr9_72a68986;Best_gnomAD_Mismatch_OFP=0.56303;Best_gnomAD_Mismatch_SVTYPE=DEL;Best_gnomAD_Mismatch_AF=8.2e-05;Best_gnomAD_Mismatch_Het=10;Best_gnomAD_Mismatch_HomAlt=0;gnomAD_SV_Cov=1	GT:PSV:LN:DR:ST:QV:TY:ID:RAL:AAL:CO	0/1:NA:7315:21,2:-:480:DUP:DUP00029046:NA:NA:chr9_110267690-chr9_110275005	0/1:NA:7316:0,0:-:344:DUP:MantaDUP_TANDEM_211865_1_2_0_0_0:NA:NA:chr9_110267689-chr9_110275005	0/1:NA:7318:0,0:-:110:DUP:5706:NA:NA:chr9_110267689-chr9_110275007	split	q31.3	LOC107987114				XR_001746890	1	1.1E+08	1.1E+08	7319	0	0	no	3	intron1-intron2	UTR	2249	5'	1.1E+08	1.1E+08																										dbVar	chr9:110129325-110304387	0.01							IMH	9:65437055-134286391;9:80897170-125694547;9:89671548-130954998	0.4714																																													-1						full=3	0/1
9_110765848_110766057_DEL_1	9	1.11E+08	1.11E+08	-208	DEL	ILS_AP001,ILS_AP001_1,ILS_AP001_2	MantaDEL:211911:0:1:0:0:0	ACAGGTGTGAGCCACCACACTCAGCCAAAGCAATCCATTTTTATGCTTAGGTTCAATGAAGTATGAACAGCTGTGTAGAAATATGATTAGACACAAAGGGTATGATCTAATGCTAACAGGCTGAGTGGGGAAACTAGCCCATCCTGCTTGTTCAGATTTTTCTCAGCCCCTCTGTGCAGCACTCACTCCTCCCAGGCAAGAGGTAGGAT	A	325	PASS	SUPP=3;SUPP_VEC=111;SVLEN=-208;SVTYPE=DEL;SVMETHOD=SURVIVOR1.0.7;CHR2=9;END=110766056;CIPOS=0,1;CIEND=0,1;STRANDS=+;Max_AF=0.0003419;Max_Het=30;Max_HomAlt=1;Max_PopMax_AF=0.003636;CCDG_Count=1;Best_CCDG_ID=435033;Best_CCDG_OFP=0.995215;Best_CCDG_AF=0.0003419;Best_CCDG_Het=0;Best_CCDG_HomAlt=0;Best_CCDG_PopMax_AF=0;Best_CCDG_Mismatch_ID=608311;Best_CCDG_Mismatch_OFP=7.767e-06;Best_CCDG_Mismatch_SVTYPE=INV;Best_CCDG_Mismatch_AF=3.419e-05;Best_CCDG_Mismatch_Het=0;Best_CCDG_Mismatch_HomAlt=0;SV_Cov=1;CCDG_SV_Cov=0.995215;TOPMed_Count=1;Best_TOPMed_ID=DEL_9:110765850-110766056;Best_TOPMed_OFP=0.995215;Best_TOPMed_AF=0.000123;Best_TOPMed_Het=30;Best_TOPMed_HomAlt=1;Best_TOPMed_PopMax_AF=0.00344418;TOPMed_SV_Cov=1;ThousG_Count=1;Best_ThousG_ID=HGSV_164623;Best_ThousG_OFP=0.995215;Best_ThousG_AF=0.000312;Best_ThousG_Het=2;Best_ThousG_HomAlt=0;Best_ThousG_PopMax_AF=0.001664;ThousG_SV_Cov=0.995215;gnomAD_Count=1;Best_gnomAD_ID=gnomAD-SV_v3_DEL_chr9_c7517a2c;Best_gnomAD_OFP=0.995215;Best_gnomAD_AF=0.000127;Best_gnomAD_Het=14;Best_gnomAD_HomAlt=1;Best_gnomAD_PopMax_AF=0.003636;Best_gnomAD_Mismatch_ID=gnomAD-SV_v3_DUP_chr9_005c44cc;Best_gnomAD_Mismatch_OFP=0.000567588;Best_gnomAD_Mismatch_SVTYPE=DUP;Best_gnomAD_Mismatch_AF=1.6e-05;Best_gnomAD_Mismatch_Het=2;Best_gnomAD_Mismatch_HomAlt=0;gnomAD_SV_Cov=1	GT:PSV:LN:DR:ST:QV:TY:ID:RAL:AAL:CO	0/1:NA:209:0,0:+:145:DEL:DEL00029056:NA:NA:chr9_110765848-chr9_110766057	0/1:NA:208:0,0:+:325:DEL:MantaDEL_211911_0_1_0_0_0:ACAGGTGTGAGCCACCACACTCAGCCAAAGCAATCCATTTTTATGCTTAGGTTCAATGAAGTATGAACAGCTGTGTAGAAATATGATTAGACACAAAGGGTATGATCTAATGCTAACAGGCTGAGTGGGGAAACTAGCCCATCCTGCTTGTTCAGATTTTTCTCAGCCCCTCTGTGCAGCACTCACTCCTCCCAGGCAAGAGGTAGGAT:A:chr9_110765848-chr9_110766056	0/1:NA:207:0,0:+:185:DEL:5707:NA:NA:chr9_110765849-chr9_110766056	split	q31.3	LOC107987115				XR_001746892	2	1.11E+08	1.11E+08	210	0	0	no	3	intron2-intron2	UTR	1010	3'	1.11E+08	1.11E+08																																			IMH	9:65437055-134286391;9:80897170-125694547;9:89671548-130954998	0.4714																																													-1						full=3	0/1
4_9677435_9677500_DEL_1	4	9677435	9677500	-64	DEL	ILS_AP001,ILS_AP001_1,ILS_AP001_2	MantaDEL:144537:0:1:0:0:0	CATCGATACTGTCATGAACTGGTTCACCTAGGAAGACTTTGACTTTGTGACTCTGTGCTACAGAG	C	624	PASS	SUPP=3;SUPP_VEC=111;SVLEN=-64;SVTYPE=DEL;SVMETHOD=SURVIVOR1.0.7;CHR2=4;END=9677499;CIPOS=0,1;CIEND=0,1;STRANDS=+;Max_AF=0.000781;Max_Het=67;Max_HomAlt=1;Max_PopMax_AF=0.005102;CCDG_Count=0;Best_CCDG_ID=333250;Best_CCDG_OFP=0.0093633;Best_CCDG_AF=0.0001026;Best_CCDG_Het=0;Best_CCDG_HomAlt=0;Best_CCDG_PopMax_AF=0;Best_CCDG_Mismatch_ID=333251_1;Best_CCDG_Mismatch_OFP=0.0153846;Best_CCDG_Mismatch_SVTYPE=BND;Best_CCDG_Mismatch_AF=0.0007864;Best_CCDG_Mismatch_Het=0;Best_CCDG_Mismatch_HomAlt=0;SV_Cov=1;CCDG_SV_Cov=1;TOPMed_Count=0;Best_TOPMed_ID=DEL_4:9676028-9683014;Best_TOPMed_OFP=0.00930166;Best_TOPMed_AF=7.8e-05;Best_TOPMed_Het=22;Best_TOPMed_HomAlt=0;Best_TOPMed_PopMax_AF=0.000239527;Best_TOPMed_Mismatch_ID=INV_4:9552055-9715383;Best_TOPMed_Mismatch_OFP=0.000397967;Best_TOPMed_Mismatch_SVTYPE=INV;Best_TOPMed_Mismatch_AF=4e-06;Best_TOPMed_Mismatch_Het=1;Best_TOPMed_Mismatch_HomAlt=0;TOPMed_SV_Cov=1;ThousG_Count=1;Best_ThousG_ID=HGSV_109152;Best_ThousG_OFP=0.984615;Best_ThousG_AF=0.000781;Best_ThousG_Het=5;Best_ThousG_HomAlt=0;Best_ThousG_PopMax_AF=0.003419;Best_ThousG_Mismatch_ID=HGSV_109100;Best_ThousG_Mismatch_OFP=0.000133584;Best_ThousG_Mismatch_SVTYPE=DUP;Best_ThousG_Mismatch_AF=0.000312;Best_ThousG_Mismatch_Het=2;Best_ThousG_Mismatch_HomAlt=0;ThousG_SV_Cov=1;gnomAD_Count=1;Best_gnomAD_ID=gnomAD-SV_v3_DEL_chr4_864b8b7d;Best_gnomAD_OFP=0.90022;Best_gnomAD_AF=0.000548;Best_gnomAD_Het=67;Best_gnomAD_HomAlt=1;Best_gnomAD_PopMax_AF=0.005102;Best_gnomAD_Mismatch_ID=gnomAD-SV_v3_DUP_chr4_522a85bf;Best_gnomAD_Mismatch_OFP=0.000836045;Best_gnomAD_Mismatch_SVTYPE=DUP;Best_gnomAD_Mismatch_AF=8e-06;Best_gnomAD_Mismatch_Het=1;Best_gnomAD_Mismatch_HomAlt=0;gnomAD_SV_Cov=1	GT:PSV:LN:DR:ST:QV:TY:ID:RAL:AAL:CO	0/1:NA:65:0,0:+:386:DEL:DEL00012463:NA:NA:chr4_9677435-chr4_9677500	0/1:NA:64:0,0:+:624:DEL:MantaDEL_144537_0_1_0_0_0:CATCGATACTGTCATGAACTGGTTCACCTAGGAAGACTTTGACTTTGTGACTCTGTGCTACAGAG:C:chr4_9677435-chr4_9677499	0/1:NA:63:0,0:+:353:DEL:2565:NA:NA:chr4_9677436-chr4_9677499	split	p16.1	LOC124900660				XR_007058018	1	9659024	9687076	66	0	0	no	5	exon3-exon3	UTR	124	3'	9677434	9677500																										dbVar	chr4:9634602-9716386	0.01	dbVar	chr4:9675966-9682958;chr4:9676050-9682991	0.01				IMH	4:1330317-92330038;4:2821273-17704603;4:4772332-76608755	0.0791																																													-1						full=1	0/1
4_58562829_58569632_DUP_1	4	58562829	58569632	6800	DUP	ILS_AP001,ILS_AP001_1,ILS_AP001_2	MantaDUP:TANDEM:148111:0:1:1:1:0	T		1440	PASS	SUPP=3;SUPP_VEC=111;SVLEN=6800;SVTYPE=DUP;SVMETHOD=SURVIVOR1.0.7;CHR2=4;END=58569632;CIPOS=-3,0;CIEND=-1,0;STRANDS=-;Max_AF=0;Max_Het=0;Max_HomAlt=0;Max_PopMax_AF=0;CCDG_Count=0;Best_CCDG_Mismatch_ID=586980;Best_CCDG_Mismatch_OFP=0.000206405;Best_CCDG_Mismatch_SVTYPE=INV;Best_CCDG_Mismatch_AF=0.004513;Best_CCDG_Mismatch_Het=0;Best_CCDG_Mismatch_HomAlt=0;SV_Cov=1;CCDG_SV_Cov=0;TOPMed_Count=0;Best_TOPMed_ID=DUP_4:58560946-58570336;Best_TOPMed_OFP=0.724127;Best_TOPMed_AF=7e-06;Best_TOPMed_Het=0;Best_TOPMed_HomAlt=1;Best_TOPMed_PopMax_AF=0.000218007;Best_TOPMed_Mismatch_ID=INV_4:57645482-58575877;Best_TOPMed_Mismatch_OFP=0.00730978;Best_TOPMed_Mismatch_SVTYPE=INV;Best_TOPMed_Mismatch_AF=4e-06;Best_TOPMed_Mismatch_Het=1;Best_TOPMed_Mismatch_HomAlt=0;TOPMed_SV_Cov=1;ThousG_Count=0;Best_ThousG_ID=HGSV_111856;Best_ThousG_OFP=0.0024321;Best_ThousG_AF=0.000156;Best_ThousG_Het=1;Best_ThousG_HomAlt=0;Best_ThousG_PopMax_AF=0.000855;Best_ThousG_Mismatch_ID=HGSV_111855;Best_ThousG_Mismatch_OFP=0.000147037;Best_ThousG_Mismatch_SVTYPE=INS;Best_ThousG_Mismatch_AF=0.15029;Best_ThousG_Mismatch_Het=0;Best_ThousG_Mismatch_HomAlt=0;ThousG_SV_Cov=0.169534;gnomAD_Count=0;Best_gnomAD_ID=gnomAD-SV_v3_DUP_chr4_41308de1;Best_gnomAD_OFP=0.0382297;Best_gnomAD_AF=0.030721;Best_gnomAD_Het=1874;Best_gnomAD_HomAlt=276;Best_gnomAD_PopMax_AF=0.162393;gnomAD_Mismatches=gnomAD-SV_v3_DEL_chr4_a828013e;gnomAD_Mismatches_Count=1;gnomAD_Mismatch_SVTYPEs=DEL;Best_gnomAD_Mismatch_ID=gnomAD-SV_v3_DEL_chr4_a828013e;Best_gnomAD_Mismatch_OFP=0.843692;Best_gnomAD_Mismatch_SVTYPE=DEL;Best_gnomAD_Mismatch_AF=1.6e-05;Best_gnomAD_Mismatch_Het=2;Best_gnomAD_Mismatch_HomAlt=0;gnomAD_SV_Cov=1	GT:PSV:LN:DR:ST:QV:TY:ID:RAL:AAL:CO	0/1:NA:6802:25,13:-:1440:DUP:DUP00014346:NA:NA:chr4_58562829-chr4_58569631	0/1:NA:6800:0,0:-:780:DUP:MantaDUP_TANDEM_148111_0_1_1_1_0:NA:NA:chr4_58562832-chr4_58569632	1/1:NA:6800:0,0:-:305:DUP:2704:NA:NA:chr4_58562832-chr4_58569632	split	q13.1	LOC124900833				XR_007058424	1	58562164	58565212	2384	0	0	no	3	txStart-intron2	UTR	509	3'	58562828	58565212																										dbVar	chr4:57322428-61871079	0.01							IMH	4:1330317-92330038;4:4772332-76608755;4:21291890-139485605;4:21757282-83521389;4:32217847-78249129;4:41369891-94869199	0.1882																																													-1						full=3	1/1
4_58562830_58569374_DEL_1	4	58562830	58569374	-6543	DEL	ILS_AP001,ILS_AP001_1,ILS_AP001_2	MantaDEL:148111:0:1:0:0:0	G		1680	PASS	SUPP=3;SUPP_VEC=111;SVLEN=-6543;SVTYPE=DEL;SVMETHOD=SURVIVOR1.0.7;CHR2=4;END=58569373;CIPOS=0,4;CIEND=0,1;STRANDS=+;Max_AF=1.6e-05;Max_Het=2;Max_HomAlt=0;Max_PopMax_AF=0.000247;CCDG_Count=0;Best_CCDG_Mismatch_ID=586980;Best_CCDG_Mismatch_OFP=0.000198605;Best_CCDG_Mismatch_SVTYPE=INV;Best_CCDG_Mismatch_AF=0.004513;Best_CCDG_Mismatch_Het=0;Best_CCDG_Mismatch_HomAlt=0;SV_Cov=1;CCDG_SV_Cov=0;TOPMed_Count=0;Best_TOPMed_ID=DEL_4:58042412-59020458;Best_TOPMed_OFP=0.00669088;Best_TOPMed_AF=4e-06;Best_TOPMed_Het=1;Best_TOPMed_HomAlt=0;Best_TOPMed_PopMax_AF=6.26025e-06;Best_TOPMed_Mismatch_ID=DUP_4:58560946-58570336;Best_TOPMed_Mismatch_OFP=0.696763;Best_TOPMed_Mismatch_SVTYPE=DUP;Best_TOPMed_Mismatch_AF=7e-06;Best_TOPMed_Mismatch_Het=0;Best_TOPMed_Mismatch_HomAlt=1;TOPMed_SV_Cov=1;ThousG_Count=0;Best_ThousG_Mismatch_ID=HGSV_111856;Best_ThousG_Mismatch_OFP=0.00151959;Best_ThousG_Mismatch_SVTYPE=DUP;Best_ThousG_Mismatch_AF=0.000156;Best_ThousG_Mismatch_Het=1;Best_ThousG_Mismatch_HomAlt=0;ThousG_SV_Cov=0;gnomAD_Count=1;Best_gnomAD_ID=gnomAD-SV_v3_DEL_chr4_a828013e;Best_gnomAD_OFP=0.81181;Best_gnomAD_AF=1.6e-05;Best_gnomAD_Het=2;Best_gnomAD_HomAlt=0;Best_gnomAD_PopMax_AF=0.000247;Best_gnomAD_Mismatch_ID=gnomAD-SV_v3_DUP_chr4_e4405b6f;Best_gnomAD_Mismatch_OFP=0.0265892;Best_gnomAD_Mismatch_SVTYPE=DUP;Best_gnomAD_Mismatch_AF=0.083682;Best_gnomAD_Mismatch_Het=9593;Best_gnomAD_Mismatch_HomAlt=0;gnomAD_SV_Cov=1	GT:PSV:LN:DR:ST:QV:TY:ID:RAL:AAL:CO	0/1:NA:6544:26,17:+:1680:DEL:DEL00014347:NA:NA:chr4_58562830-chr4_58569374	0/1:NA:6543:0,0:+:754:DEL:MantaDEL_148111_0_1_0_0_0:NA:NA:chr4_58562830-chr4_58569373	0/1:NA:6539:0,0:+:737:DEL:2703:NA:NA:chr4_58562834-chr4_58569373	split	q13.1	LOC124900833				XR_007058424	1	58562164	58565212	2383	0	0	no	3	txStart-intron2	UTR	510	3'	58562829	58565212																										dbVar	chr4:57322428-61871079	0.01							IMH	4:1330317-92330038;4:4772332-76608755;4:21291890-139485605;4:21757282-83521389;4:32217847-78249129;4:41369891-94869199	0.1882																																													-1						full=3	0/1
9_110267689_110275007_DUP_1	9	1.1E+08	1.1E+08	7316	DUP	ILS_AP001,ILS_AP001_1,ILS_AP001_2	MantaDUP:TANDEM:211865:1:2:0:0:0	T		480	PASS	SUPP=3;SUPP_VEC=111;SVLEN=7316;SVTYPE=DUP;SVMETHOD=SURVIVOR1.0.7;CHR2=9;END=110275005;CIPOS=0,1;CIEND=0,2;STRANDS=-;Max_AF=8e-06;Max_Het=1;Max_HomAlt=0;Max_PopMax_AF=0.000229;CCDG_Count=0;Best_CCDG_Mismatch_ID=434989;Best_CCDG_Mismatch_OFP=0.554245;Best_CCDG_Mismatch_SVTYPE=DEL;Best_CCDG_Mismatch_AF=0.1392;Best_CCDG_Mismatch_Het=0;Best_CCDG_Mismatch_HomAlt=0;SV_Cov=1;CCDG_SV_Cov=0;TOPMed_Count=0;Best_TOPMed_Mismatch_ID=DEL_9:110266596-110270066;Best_TOPMed_Mismatch_OFP=0.222593;Best_TOPMed_Mismatch_SVTYPE=DEL;Best_TOPMed_Mismatch_AF=4e-06;Best_TOPMed_Mismatch_Het=1;Best_TOPMed_Mismatch_HomAlt=0;TOPMed_SV_Cov=0;ThousG_Count=0;Best_ThousG_ID=HGSV_164584;Best_ThousG_OFP=0.0261064;Best_ThousG_AF=0.265436;Best_ThousG_Het=808;Best_ThousG_HomAlt=430;Best_ThousG_PopMax_AF=0.613074;Best_ThousG_Mismatch_ID=HGSV_164582;Best_ThousG_Mismatch_OFP=2.5337e-08;Best_ThousG_Mismatch_SVTYPE=CPX;Best_ThousG_Mismatch_AF=0.228224;Best_ThousG_Mismatch_Het=1136;Best_ThousG_Mismatch_HomAlt=163;ThousG_SV_Cov=1;gnomAD_Count=1;Best_gnomAD_ID=gnomAD-SV_v3_DUP_chr9_e71fd71d;Best_gnomAD_OFP=0.820145;Best_gnomAD_AF=8e-06;Best_gnomAD_Het=1;Best_gnomAD_HomAlt=0;Best_gnomAD_PopMax_AF=0.000229;Best_gnomAD_Mismatch_ID=gnomAD-SV_v3_DEL_chr9_72a68986;Best_gnomAD_Mismatch_OFP=0.56303;Best_gnomAD_Mismatch_SVTYPE=DEL;Best_gnomAD_Mismatch_AF=8.2e-05;Best_gnomAD_Mismatch_Het=10;Best_gnomAD_Mismatch_HomAlt=0;gnomAD_SV_Cov=1	GT:PSV:LN:DR:ST:QV:TY:ID:RAL:AAL:CO	0/1:NA:7315:21,2:-:480:DUP:DUP00029046:NA:NA:chr9_110267690-chr9_110275005	0/1:NA:7316:0,0:-:344:DUP:MantaDUP_TANDEM_211865_1_2_0_0_0:NA:NA:chr9_110267689-chr9_110275005	0/1:NA:7318:0,0:-:110:DUP:5706:NA:NA:chr9_110267689-chr9_110275007	split	q31.3	LOC124902246				XR_007061729	1	1.1E+08	1.1E+08	1101	0	0	no	2	txStart-intron1	UTR	296	5'	1.1E+08	1.1E+08																										dbVar	chr9:110129325-110304387	0.01							IMH	9:65437055-134286391;9:80897170-125694547;9:89671548-130954998	0.4714																																													-1						full=3	0/1
1_238530121_238538212_DUP_1	1	2.39E+08	2.39E+08	8091	DUP	ILS_AP001,ILS_AP001_1,ILS_AP001_2	MantaDUP:TANDEM:204825:0:1:2:0:0	T		2280	PASS	SUPP=3;SUPP_VEC=111;SVLEN=8091;SVTYPE=DUP;SVMETHOD=SURVIVOR1.0.7;CHR2=1;END=238538212;CIPOS=0,1;CIEND=0,0;STRANDS=-;Max_AF=0;Max_Het=0;Max_HomAlt=0;Max_PopMax_AF=0;CCDG_Count=0;Best_CCDG_ID=460872;Best_CCDG_OFP=0.00656401;Best_CCDG_AF=3.419e-05;Best_CCDG_Het=0;Best_CCDG_HomAlt=0;Best_CCDG_PopMax_AF=0;Best_CCDG_Mismatch_ID=142309;Best_CCDG_Mismatch_OFP=0.0403346;Best_CCDG_Mismatch_SVTYPE=DEL;Best_CCDG_Mismatch_AF=3.42e-05;Best_CCDG_Mismatch_Het=0;Best_CCDG_Mismatch_HomAlt=0;SV_Cov=1;CCDG_SV_Cov=1;TOPMed_Count=0;Best_TOPMed_ID=DUP_1:238488419-238615184;Best_TOPMed_OFP=0.0638336;Best_TOPMed_AF=0.000178;Best_TOPMed_Het=48;Best_TOPMed_HomAlt=1;Best_TOPMed_PopMax_AF=0.000296026;Best_TOPMed_Mismatch_ID=DEL_1:238534402-238541900;Best_TOPMed_Mismatch_OFP=0.239436;Best_TOPMed_Mismatch_SVTYPE=DEL;Best_TOPMed_Mismatch_AF=4e-06;Best_TOPMed_Mismatch_Het=1;Best_TOPMed_Mismatch_HomAlt=0;TOPMed_SV_Cov=1;ThousG_Count=0;Best_ThousG_ID=HGSV_12298;Best_ThousG_OFP=0.0638336;Best_ThousG_AF=0.000156;Best_ThousG_Het=1;Best_ThousG_HomAlt=0;Best_ThousG_PopMax_AF=0.00102;ThousG_Mismatches=HGSV_12303;ThousG_Mismatches_Count=1;ThousG_Mismatch_SVTYPEs=DEL;Best_ThousG_Mismatch_ID=HGSV_12303;Best_ThousG_Mismatch_OFP=0.967364;Best_ThousG_Mismatch_SVTYPE=DEL;Best_ThousG_Mismatch_AF=0.000156;Best_ThousG_Mismatch_Het=1;Best_ThousG_Mismatch_HomAlt=0;ThousG_SV_Cov=1;gnomAD_Count=0;Best_gnomAD_ID=gnomAD-SV_v3_DUP_chr1_cd345c39;Best_gnomAD_OFP=0.0638336;Best_gnomAD_AF=0.000254;Best_gnomAD_Het=30;Best_gnomAD_HomAlt=1;Best_gnomAD_PopMax_AF=0.000636;gnomAD_Mismatches=gnomAD-SV_v3_DEL_chr1_ea98a6f7;gnomAD_Mismatches_Count=1;gnomAD_Mismatch_SVTYPEs=DEL;Best_gnomAD_Mismatch_ID=gnomAD-SV_v3_DEL_chr1_ea98a6f7;Best_gnomAD_Mismatch_OFP=0.921782;Best_gnomAD_Mismatch_SVTYPE=DEL;Best_gnomAD_Mismatch_AF=0.000135;Best_gnomAD_Mismatch_Het=17;Best_gnomAD_Mismatch_HomAlt=0;gnomAD_SV_Cov=1	GT:PSV:LN:DR:ST:QV:TY:ID:RAL:AAL:CO	0/1:NA:8090:36,21:-:2280:DUP:DUP00004939:NA:NA:chr1_238530122-chr1_238538212	0/1:NA:8091:0,0:-:999:DUP:MantaDUP_TANDEM_204825_0_1_2_0_0:NA:NA:chr1_238530121-chr1_238538212	1/1:NA:8091:0,0:-:503:DUP:1012:NA:NA:chr1_238530121-chr1_238538212	split	q43	LOC124904565				XR_007066966	1	2.39E+08	2.39E+08	4764	0	0	no	4	txStart-intron1	UTR	4700	5'	2.39E+08	2.39E+08																										dbVar	chr1:237600877-238691725;chr1:238021699-238768700;chr1:238488418-238615188;chr1:238498219-238566226	0.01																																																						-1						full=3	1/1
1_238530104_238537891_DEL_1	1	2.39E+08	2.39E+08	-7786	DEL	ILS_AP001,ILS_AP001_1,ILS_AP001_2	MantaDEL:204825:0:1:0:0:0	G		1080	PASS	SUPP=3;SUPP_VEC=111;SVLEN=-7786;SVTYPE=DEL;SVMETHOD=SURVIVOR1.0.7;CHR2=1;END=238537890;CIPOS=0,4;CIEND=0,1;STRANDS=+;Max_AF=0.000156;Max_Het=17;Max_HomAlt=0;Max_PopMax_AF=0.00056;CCDG_Count=0;Best_CCDG_ID=142309;Best_CCDG_OFP=0.0388143;Best_CCDG_AF=3.42e-05;Best_CCDG_Het=0;Best_CCDG_HomAlt=0;Best_CCDG_PopMax_AF=0;Best_CCDG_Mismatch_ID=460872;Best_CCDG_Mismatch_OFP=0.0063166;Best_CCDG_Mismatch_SVTYPE=DUP;Best_CCDG_Mismatch_AF=3.419e-05;Best_CCDG_Mismatch_Het=0;Best_CCDG_Mismatch_HomAlt=0;SV_Cov=1;CCDG_SV_Cov=1;TOPMed_Count=0;Best_TOPMed_ID=DEL_1:238534402-238541900;Best_TOPMed_OFP=0.208554;Best_TOPMed_AF=4e-06;Best_TOPMed_Het=1;Best_TOPMed_HomAlt=0;Best_TOPMed_PopMax_AF=6.2626e-06;Best_TOPMed_Mismatch_ID=DUP_1:238488419-238615184;Best_TOPMed_Mismatch_OFP=0.0614277;Best_TOPMed_Mismatch_SVTYPE=DUP;Best_TOPMed_Mismatch_AF=0.000178;Best_TOPMed_Mismatch_Het=48;Best_TOPMed_Mismatch_HomAlt=1;TOPMed_SV_Cov=1;ThousG_Count=1;Best_ThousG_ID=HGSV_12303;Best_ThousG_OFP=0.930903;Best_ThousG_AF=0.000156;Best_ThousG_Het=1;Best_ThousG_HomAlt=0;Best_ThousG_PopMax_AF=0.00056;Best_ThousG_Mismatch_ID=HGSV_12298;Best_ThousG_Mismatch_OFP=0.0614277;Best_ThousG_Mismatch_SVTYPE=DUP;Best_ThousG_Mismatch_AF=0.000156;Best_ThousG_Mismatch_Het=1;Best_ThousG_Mismatch_HomAlt=0;ThousG_SV_Cov=1;gnomAD_Count=1;Best_gnomAD_ID=gnomAD-SV_v3_DEL_chr1_ea98a6f7;Best_gnomAD_OFP=0.962245;Best_gnomAD_AF=0.000135;Best_gnomAD_Het=17;Best_gnomAD_HomAlt=0;Best_gnomAD_PopMax_AF=0.000385;Best_gnomAD_Mismatch_ID=gnomAD-SV_v3_DUP_chr1_cd345c39;Best_gnomAD_Mismatch_OFP=0.0614277;Best_gnomAD_Mismatch_SVTYPE=DUP;Best_gnomAD_Mismatch_AF=0.000254;Best_gnomAD_Mismatch_Het=30;Best_gnomAD_Mismatch_HomAlt=1;gnomAD_SV_Cov=1	GT:PSV:LN:DR:ST:QV:TY:ID:RAL:AAL:CO	0/1:NA:7787:33,8:+:1080:DEL:DEL00004938:NA:NA:chr1_238530104-chr1_238537891	0/1:NA:7786:0,0:+:445:DEL:MantaDEL_204825_0_1_0_0_0:NA:NA:chr1_238530104-chr1_238537890	0/1:NA:7782:0,0:+:299:DEL:1011:NA:NA:chr1_238530108-chr1_238537890	split	q43	LOC124904565				XR_007066966	1	2.39E+08	2.39E+08	4443	0	0	no	4	txStart-intron1	UTR	4379	5'	2.39E+08	2.39E+08																										dbVar	chr1:237600877-238691725;chr1:238021699-238768700;chr1:238488418-238615188;chr1:238498219-238566226	0.01																																																						-1						full=3	0/1
2_44409095_44409184_DEL_1	2	44409095	44409184	-88	DEL	ILS_AP001,ILS_AP001_1,ILS_AP001_2	MantaDEL:174423:0:0:0:0:0	ATATATATATATATATATATATATATATATATATATATATATATATATATATATATATATATATATATATATATATATATATATATATG	A	1140	PASS	SUPP=3;SUPP_VEC=111;SVLEN=-88;SVTYPE=DEL;SVMETHOD=SURVIVOR1.0.7;CHR2=2;END=44409183;CIPOS=0,5;CIEND=-15,1;STRANDS=+;Max_AF=0;Max_Het=0;Max_HomAlt=0;Max_PopMax_AF=0;CCDG_Count=0;Best_CCDG_ID=271444;Best_CCDG_OFP=0.00477493;Best_CCDG_AF=3.419e-05;Best_CCDG_Het=0;Best_CCDG_HomAlt=0;Best_CCDG_PopMax_AF=0;Best_CCDG_Mismatch_ID=271446_1;Best_CCDG_Mismatch_OFP=0.011236;Best_CCDG_Mismatch_SVTYPE=BND;Best_CCDG_Mismatch_AF=0.636;Best_CCDG_Mismatch_Het=0;Best_CCDG_Mismatch_HomAlt=0;SV_Cov=1;CCDG_SV_Cov=1;TOPMed_Count=0;Best_TOPMed_ID=DEL_2:44391632-44410277;Best_TOPMed_OFP=0.00477289;Best_TOPMed_AF=3.2e-05;Best_TOPMed_Het=9;Best_TOPMed_HomAlt=0;Best_TOPMed_PopMax_AF=0.000107787;Best_TOPMed_Mismatch_ID=INV_2:44403211-44419415;Best_TOPMed_Mismatch_OFP=0.00549179;Best_TOPMed_Mismatch_SVTYPE=INV;Best_TOPMed_Mismatch_AF=4e-06;Best_TOPMed_Mismatch_Het=1;Best_TOPMed_Mismatch_HomAlt=0;TOPMed_SV_Cov=1;ThousG_Count=0;Best_ThousG_ID=HGSV_77537;Best_ThousG_OFP=0.775281;Best_ThousG_AF=0.437305;Best_ThousG_Het=1391;Best_ThousG_HomAlt=703;Best_ThousG_PopMax_AF=0.627962;Best_ThousG_Mismatch_ID=HGSV_77475;Best_ThousG_Mismatch_OFP=6.63578e-07;Best_ThousG_Mismatch_SVTYPE=INS;Best_ThousG_Mismatch_AF=0.000468;Best_ThousG_Mismatch_Het=3;Best_ThousG_Mismatch_HomAlt=0;ThousG_SV_Cov=0.775281;gnomAD_Count=0;Best_gnomAD_ID=gnomAD-SV_v3_DEL_chr2_ac5169c0;Best_gnomAD_OFP=0.775281;Best_gnomAD_AF=0.424911;Best_gnomAD_Het=38541;Best_gnomAD_HomAlt=6924;Best_gnomAD_PopMax_AF=0.550847;gnomAD_Mismatches=gnomAD-SV_v3_DUP_chr2_a7918d70,gnomAD-SV_v3_DUP_chr2_d33e7116,gnomAD-SV_v3_DUP_chr2_a1ef2047;gnomAD_Mismatches_Count=3;gnomAD_Mismatch_SVTYPEs=DUP;Best_gnomAD_Mismatch_ID=gnomAD-SV_v3_DUP_chr2_a1ef2047;Best_gnomAD_Mismatch_OFP=0.828365;Best_gnomAD_Mismatch_SVTYPE=DUP;Best_gnomAD_Mismatch_AF=0.000226;Best_gnomAD_Mismatch_Het=20;Best_gnomAD_Mismatch_HomAlt=4;gnomAD_SV_Cov=1	GT:PSV:LN:DR:ST:QV:TY:ID:RAL:AAL:CO	1/1:NA:89:0,0:+:1140:DEL:DEL00006084:NA:NA:chr2_44409095-chr2_44409184	1/1:NA:88:0,0:+:732:DEL:MantaDEL_174423_0_0_0_0_0:ATATATATATATATATATATATATATATATATATATATATATATATATATATATATATATATATATATATATATATATATATATATATG:A:chr2_44409095-chr2_44409183	1/1:NA:68:0,0:+:699:DEL:1226:NA:NA:chr2_44409100-chr2_44409168	split	p21	LOC124907759				XR_007086304	1	44401132	44421913	90	0	0	no	2	intron1-intron1	UTR	2828	3'	44409094	44409184																										dbVar;gnomAD-SV_v3_DUP_chr2_d33e7116	chr2:44405812-44507018;chr2:44409080-44409188	0.0624	dbVar	chr2:44278237-44413378;chr2:44391631-44410269	0.01				IMH	2:21055811-76514955;2:32873521-53185851	0.1623																																													-1						full=1	1/1
9_110998042_111009830_DEL_1	9	1.11E+08	1.11E+08	-11787	DEL	ILS_AP001,ILS_AP001_1,ILS_AP001_2	MantaDEL:211928:0:1:0:0:0	C		1103	PASS	SUPP=3;SUPP_VEC=111;SVLEN=-11787;SVTYPE=DEL;SVMETHOD=SURVIVOR1.0.7;CHR2=9;END=111009829;CIPOS=0,6;CIEND=0,1;STRANDS=+;Max_AF=0.000312;Max_Het=19;Max_HomAlt=1;Max_PopMax_AF=0.0021777;CCDG_Count=1;Best_CCDG_ID=435050;Best_CCDG_OFP=0.999491;Best_CCDG_AF=3.419e-05;Best_CCDG_Het=0;Best_CCDG_HomAlt=0;Best_CCDG_PopMax_AF=0;Best_CCDG_Mismatch_ID=608311;Best_CCDG_Mismatch_OFP=0.000438074;Best_CCDG_Mismatch_SVTYPE=INV;Best_CCDG_Mismatch_AF=3.419e-05;Best_CCDG_Mismatch_Het=0;Best_CCDG_Mismatch_HomAlt=0;SV_Cov=1;CCDG_SV_Cov=0.999491;TOPMed_Count=1;Best_TOPMed_ID=DEL_9:110998049-111009829;Best_TOPMed_OFP=0.999491;Best_TOPMed_AF=7.4e-05;Best_TOPMed_Het=19;Best_TOPMed_HomAlt=1;Best_TOPMed_PopMax_AF=0.0021777;Best_TOPMed_Mismatch_ID=DUP_9:111002802-111008800;Best_TOPMed_Mismatch_OFP=0.508992;Best_TOPMed_Mismatch_SVTYPE=DUP;Best_TOPMed_Mismatch_AF=4e-06;Best_TOPMed_Mismatch_Het=1;Best_TOPMed_Mismatch_HomAlt=0;TOPMed_SV_Cov=0.999491;ThousG_Count=1;Best_ThousG_ID=HGSV_164637;Best_ThousG_OFP=0.999491;Best_ThousG_AF=0.000312;Best_ThousG_Het=2;Best_ThousG_HomAlt=0;Best_ThousG_PopMax_AF=0.001664;ThousG_SV_Cov=0.999491;gnomAD_Count=1;Best_gnomAD_ID=gnomAD-SV_v3_DEL_chr9_7139caaf;Best_gnomAD_OFP=0.999491;Best_gnomAD_AF=3.2e-05;Best_gnomAD_Het=4;Best_gnomAD_HomAlt=0;Best_gnomAD_PopMax_AF=0.000681;Best_gnomAD_Mismatch_ID=gnomAD-SV_v3_INS_chr9_6fd63941;Best_gnomAD_Mismatch_OFP=0.00398711;Best_gnomAD_Mismatch_SVTYPE=INS;Best_gnomAD_Mismatch_AF=8e-06;Best_gnomAD_Mismatch_Het=1;Best_gnomAD_Mismatch_HomAlt=0;gnomAD_SV_Cov=1	GT:PSV:LN:DR:ST:QV:TY:ID:RAL:AAL:CO	0/1:NA:11788:19,7:+:1103:DEL:DEL00029057:NA:NA:chr9_110998042-chr9_111009830	0/1:NA:11787:0,0:+:498:DEL:MantaDEL_211928_0_1_0_0_0:NA:NA:chr9_110998042-chr9_111009829	0/1:NA:11781:0,0:+:368:DEL:5708:NA:NA:chr9_110998048-chr9_111009829	split	q31.3	LPAR1				XM_047422903	1	1.11E+08	1.11E+08	11789	0	0	no	8	intron3-intron4	5'UTR	629	3'	1.11E+08	1.11E+08																																			IMH	9:65437055-134286391;9:80897170-125694547;9:89671548-130954998	0.4714																										11.39	0.873443	1.061705	1.264533	-0.79036	2.571295					1902						3	1.86E-01	1.42E-03	0.9511		Cleft lip;Cleft palate;Hypertelorism;Micrognathia;Microphthalmia;long incisors;ocular hypertelorism;short snout	Meckel's cartilage deformed, abnormal;Micrognathia			full=3	0/1
10_19237576_19237674_DEL_1	10	19237576	19237674	-97	DEL	ILS_AP001,ILS_AP001_1,ILS_AP001_2	MantaDEL:38088:0:0:0:2:0	AATTATAATTATATAATTATATAATTATAATTATAATTATATAATTATATAATTATAATTATATATAAATTATTATAATTATATATAAATTATATAAT	A	480	PASS	SUPP=3;SUPP_VEC=111;SVLEN=-97;SVTYPE=DEL;SVMETHOD=SURVIVOR1.0.7;CHR2=10;END=19237673;CIPOS=0,0;CIEND=-10,1;STRANDS=+;Max_AF=0;Max_Het=0;Max_HomAlt=0;Max_PopMax_AF=0;CCDG_Count=0;Best_CCDG_ID=146122;Best_CCDG_OFP=0.022192;Best_CCDG_AF=3.419e-05;Best_CCDG_Het=0;Best_CCDG_HomAlt=0;Best_CCDG_PopMax_AF=0;Best_CCDG_Mismatch_ID=146123_1;Best_CCDG_Mismatch_OFP=0.0102041;Best_CCDG_Mismatch_SVTYPE=BND;Best_CCDG_Mismatch_AF=0.06837;Best_CCDG_Mismatch_Het=0;Best_CCDG_Mismatch_HomAlt=0;SV_Cov=1;CCDG_SV_Cov=1;TOPMed_Count=0;Best_TOPMed_ID=DEL_10:19227120-19238593;Best_TOPMed_OFP=0.00854031;Best_TOPMed_AF=4e-06;Best_TOPMed_Het=1;Best_TOPMed_HomAlt=0;Best_TOPMed_PopMax_AF=6.26339e-06;Best_TOPMed_Mismatch_ID=INV_10:19224623-19248206;Best_TOPMed_Mismatch_OFP=0.00415518;Best_TOPMed_Mismatch_SVTYPE=INV;Best_TOPMed_Mismatch_AF=0.0001;Best_TOPMed_Mismatch_Het=28;Best_TOPMed_Mismatch_HomAlt=0;TOPMed_SV_Cov=1;ThousG_Count=0;Best_ThousG_ID=HGSV_14412;Best_ThousG_OFP=0.00363988;Best_ThousG_AF=0.000156;Best_ThousG_Het=1;Best_ThousG_HomAlt=0;Best_ThousG_PopMax_AF=0;Best_ThousG_Mismatch_ID=HGSV_14289;Best_ThousG_Mismatch_OFP=1.22078e-05;Best_ThousG_Mismatch_SVTYPE=INV;Best_ThousG_Mismatch_AF=0.000156;Best_ThousG_Mismatch_Het=1;Best_ThousG_Mismatch_HomAlt=0;ThousG_SV_Cov=1;gnomAD_Count=0;Best_gnomAD_ID=gnomAD-SV_v3_DEL_chr10_8222a499;Best_gnomAD_OFP=0.52973;Best_gnomAD_AF=8e-06;Best_gnomAD_Het=1;Best_gnomAD_HomAlt=0;Best_gnomAD_PopMax_AF=0.000227;Best_gnomAD_Mismatch_ID=gnomAD-SV_v3_DUP_chr10_3dd3b75f;Best_gnomAD_Mismatch_OFP=0.000489772;Best_gnomAD_Mismatch_SVTYPE=DUP;Best_gnomAD_Mismatch_AF=1.6e-05;Best_gnomAD_Mismatch_Het=2;Best_gnomAD_Mismatch_HomAlt=0;gnomAD_SV_Cov=1	GT:PSV:LN:DR:ST:QV:TY:ID:RAL:AAL:CO	0/1:NA:98:0,0:+:480:DEL:DEL00030447:NA:NA:chr10_19237576-chr10_19237674	0/1:NA:97:0,0:+:251:DEL:MantaDEL_38088_0_0_0_2_0:AATTATAATTATATAATTATATAATTATAATTATAATTATATAATTATATAATTATAATTATATATAAATTATTATAATTATATATAAATTATATAAT:A:chr10_19237576-chr10_19237673	0/1:NA:87:0,0:+:200:DEL:5951:NA:NA:chr10_19237576-chr10_19237663	split	p12.31	MALRD1				XM_047425168	1	19046926	19287047	99	0	0	no	23	intron19-intron19	CDS	20009	3'	19237575	19237674																													dbVar	chr10:19093771-19584071;chr10:19223342-19249473;chr10:19224621-19255405	0.01																																72.62										340895						4	9.38E-21		0.7305		Cleft lip;Cleft palate;Hypertelorism;Micrognathia;Microphthalmia;abnormal eye morphology;anophthalmia				full=3	0/1
10_19473089_19473179_DEL_1	10	19473089	19473179	-89	DEL	ILS_AP001,ILS_AP001_1,ILS_AP001_2	MantaDEL:38069:0:1:0:0:0	GAAATGTGAAAGCTTGTTCATAACCTCACTTTATCCTCAACTTAAATTTTGAGGATAAATTGAGGTTATGAACAAGCTTTCACATTTCTT	G	240	PASS	SUPP=3;SUPP_VEC=111;SVLEN=-89;SVTYPE=DEL;SVMETHOD=SURVIVOR1.0.7;CHR2=10;END=19473178;CIPOS=0,10;CIEND=0,1;STRANDS=+;Max_AF=0;Max_Het=0;Max_HomAlt=0;Max_PopMax_AF=0;CCDG_Count=0;Best_CCDG_ID=146150;Best_CCDG_OFP=0.00583733;Best_CCDG_AF=3.419e-05;Best_CCDG_Het=0;Best_CCDG_HomAlt=0;Best_CCDG_PopMax_AF=0;Best_CCDG_Mismatch_ID=146153_2;Best_CCDG_Mismatch_OFP=0.0111111;Best_CCDG_Mismatch_SVTYPE=BND;Best_CCDG_Mismatch_AF=0.2654;Best_CCDG_Mismatch_Het=0;Best_CCDG_Mismatch_HomAlt=0;SV_Cov=1;CCDG_SV_Cov=1;TOPMed_Count=0;Best_TOPMed_ID=DEL_10:19470114-19478623;Best_TOPMed_OFP=0.0105746;Best_TOPMed_AF=4e-06;Best_TOPMed_Het=1;Best_TOPMed_HomAlt=0;Best_TOPMed_PopMax_AF=6.26205e-06;Best_TOPMed_Mismatch_ID=DUP_10:19321083-19476602;Best_TOPMed_Mismatch_OFP=0.0005787;Best_TOPMed_Mismatch_SVTYPE=DUP;Best_TOPMed_Mismatch_AF=4e-06;Best_TOPMed_Mismatch_Het=1;Best_TOPMed_Mismatch_HomAlt=0;TOPMed_SV_Cov=1;ThousG_Count=0;Best_ThousG_Mismatch_ID=HGSV_14289;Best_ThousG_Mismatch_OFP=1.12113e-05;Best_ThousG_Mismatch_SVTYPE=INV;Best_ThousG_Mismatch_AF=0.000156;Best_ThousG_Mismatch_Het=1;Best_ThousG_Mismatch_HomAlt=0;ThousG_SV_Cov=0;gnomAD_Count=0;Best_gnomAD_ID=gnomAD-SV_v3_DEL_chr10_d0388585;Best_gnomAD_OFP=0.243902;Best_gnomAD_AF=0.00067;Best_gnomAD_Het=84;Best_gnomAD_HomAlt=0;Best_gnomAD_PopMax_AF=0.001188;Best_gnomAD_Mismatch_ID=gnomAD-SV_v3_DUP_chr10_006be9e5;Best_gnomAD_Mismatch_OFP=0.000304053;Best_gnomAD_Mismatch_SVTYPE=DUP;Best_gnomAD_Mismatch_AF=8e-06;Best_gnomAD_Mismatch_Het=1;Best_gnomAD_Mismatch_HomAlt=0;gnomAD_SV_Cov=1	GT:PSV:LN:DR:ST:QV:TY:ID:RAL:AAL:CO	0/1:NA:90:0,0:+:240:DEL:DEL00030450:NA:NA:chr10_19473089-chr10_19473179	0/1:NA:89:0,0:+:164:DEL:MantaDEL_38069_0_1_0_0_0:GAAATGTGAAAGCTTGTTCATAACCTCACTTTATCCTCAACTTAAATTTTGAGGATAAATTGAGGTTATGAACAAGCTTTCACATTTCTT:G:chr10_19473089-chr10_19473178	0/1:NA:79:0,0:+:47:DEL:5952:NA:NA:chr10_19473099-chr10_19473178	split	p12.31	MALRD1				XM_047425167	1	19046926	19567592	91	0	0	no	34	intron30-intron30	CDS	18337	3'	19473088	19473179																													dbVar	chr10:19093771-19584071;chr10:19355571-19822382	0.01																																72.62										340895						4	9.38E-21		0.7305		Cleft lip;Cleft palate;Hypertelorism;Micrognathia;Microphthalmia;abnormal eye morphology;anophthalmia				full=3	0/1
19_4005348_4159440_DEL_1	19	4005348	4159440	-154090	DEL	ILS_AP001,ILS_AP001_1,ILS_AP001_2	MantaDEL:79083:0:1:0:0:0	C		1740	PASS	SUPP=3;SUPP_VEC=111;SVLEN=-154090;SVTYPE=DEL;SVMETHOD=SURVIVOR1.0.7;CHR2=19;END=4159440;CIPOS=-2,0;CIEND=-5,0;STRANDS=+;Max_AF=0;Max_Het=0;Max_HomAlt=0;Max_PopMax_AF=0;CCDG_Count=0;Best_CCDG_ID=253950;Best_CCDG_OFP=0.113861;Best_CCDG_AF=6.839e-05;Best_CCDG_Het=0;Best_CCDG_HomAlt=0;Best_CCDG_PopMax_AF=0;Best_CCDG_Mismatch_ID=490213;Best_CCDG_Mismatch_OFP=0.150976;Best_CCDG_Mismatch_SVTYPE=DUP;Best_CCDG_Mismatch_AF=3.419e-05;Best_CCDG_Mismatch_Het=0;Best_CCDG_Mismatch_HomAlt=0;SV_Cov=0.735014;CCDG_SV_Cov=0.290815;TOPMed_Count=0;Best_TOPMed_ID=DEL_19:4130566-4146337;Best_TOPMed_OFP=0.102362;Best_TOPMed_AF=4e-06;Best_TOPMed_Het=1;Best_TOPMed_HomAlt=0;Best_TOPMed_PopMax_AF=0.000108719;Best_TOPMed_Mismatch_ID=INV_19:3851264-4364229;Best_TOPMed_Mismatch_OFP=0.300392;Best_TOPMed_Mismatch_SVTYPE=INV;Best_TOPMed_Mismatch_AF=4e-06;Best_TOPMed_Mismatch_Het=1;Best_TOPMed_Mismatch_HomAlt=0;TOPMed_SV_Cov=0.400108;ThousG_Count=0;Best_ThousG_ID=HGSV_70074;Best_ThousG_OFP=0.0204879;Best_ThousG_AF=0.000312;Best_ThousG_Het=2;Best_ThousG_HomAlt=0;Best_ThousG_PopMax_AF=0.00112;Best_ThousG_Mismatch_ID=HGSV_70075;Best_ThousG_Mismatch_OFP=0.148371;Best_ThousG_Mismatch_SVTYPE=DUP;Best_ThousG_Mismatch_AF=0.000312;Best_ThousG_Mismatch_Het=2;Best_ThousG_Mismatch_HomAlt=0;ThousG_SV_Cov=0.0925492;gnomAD_Count=0;Best_gnomAD_ID=gnomAD-SV_v3_DEL_chr19_5ba69aa0;Best_gnomAD_OFP=0.172119;Best_gnomAD_AF=6.3e-05;Best_gnomAD_Het=8;Best_gnomAD_HomAlt=0;Best_gnomAD_PopMax_AF=0.000493;Best_gnomAD_Mismatch_ID=gnomAD-SV_v3_DUP_chr19_9114b2c5;Best_gnomAD_Mismatch_OFP=0.367091;Best_gnomAD_Mismatch_SVTYPE=DUP;Best_gnomAD_Mismatch_AF=8e-06;Best_gnomAD_Mismatch_Het=1;Best_gnomAD_Mismatch_HomAlt=0;gnomAD_SV_Cov=0.733638	GT:PSV:LN:DR:ST:QV:TY:ID:RAL:AAL:CO	0/1:NA:154087:26,11:+:1740:DEL:DEL00054230:NA:NA:chr19_4005348-chr19_4159435	0/1:NA:154090:0,0:+:999:DEL:MantaDEL_79083_0_1_0_0_0:NA:NA:chr19_4005350-chr19_4159440	0/1:NA:154090:0,0:+:650:DEL:10046:NA:NA:chr19_4005350-chr19_4159440	split	p13.3	MAP2K2				NM_030662	4	4090320	4124122	33802	1203	100	no	11	txStart-txEnd	5'UTR-3'UTR			4090320	4124122						Cardiofaciocutaneous syndrome 4, 615280 (3) AD		morbid:MAP2K2	19:4090321-4124122															11	Cardio-facio-cutaneous_syndrome;Cardiofaciocutaneous_syndrome_1;Cardiofaciocutaneous_syndrome_4;Castleman-Kojima_disease;Gastric_adenocarcinoma;Malignant_melanoma_of_skin;Melanoma;Noonan_syndrome;Noonan_syndrome_1;Pancreatic_adenocarcinoma;RASopathy;Smith-Magenis_Syndrome-like;Squamous_cell_carcinoma_of_the_head_and_neck										IMH	19:1692676-41507766	0.0103																										38.94	0.37913	0.438659	0.549595	-1.31922	1.476321	Noonan syndrome;cardiofaciocutaneous syndrome;cardiofaciocutaneous syndrome 1;cardiofaciocutaneous syndrome 4;neurofibromatosis-Noonan syndrome	AD	Definitive;Limited;Strong;Supportive	16439621;18042262;20301365[PMID]_16825433[PMID];20358587;21396583;23379592;24311457[PMID]	5605	601263	Cardiofaciocutaneous syndrome 4, 615280 (3) AD	AD	yes		1	9.67E-01	8.63E-01	0.6168	Cardiofaciocutaneous syndrome;Cleft lip;Cleft palate;High palate;Hypertelorism;Hypoplasia of the zygomatic bone;Micrognathia;Submucous cleft hard palate					full=5	0/1
19_4005230_4159441_DUP_1	19	4005230	4159441	154171	DUP	ILS_AP001,ILS_AP001_1,ILS_AP001_2	MantaDUP:TANDEM:79083:0:1:1:0:0	T		999	PASS	SUPP=3;SUPP_VEC=111;SVLEN=154171;SVTYPE=DUP;SVMETHOD=SURVIVOR1.0.7;CHR2=19;END=4159423;CIPOS=-22,1;CIEND=0,18;STRANDS=-;Max_AF=0;Max_Het=0;Max_HomAlt=0;Max_PopMax_AF=0;CCDG_Count=0;Best_CCDG_ID=490213;Best_CCDG_OFP=0.150896;Best_CCDG_AF=3.419e-05;Best_CCDG_Het=0;Best_CCDG_HomAlt=0;Best_CCDG_PopMax_AF=0;Best_CCDG_Mismatch_ID=253950;Best_CCDG_Mismatch_OFP=0.113801;Best_CCDG_Mismatch_SVTYPE=DEL;Best_CCDG_Mismatch_AF=6.839e-05;Best_CCDG_Mismatch_Het=0;Best_CCDG_Mismatch_HomAlt=0;SV_Cov=0.97591;CCDG_SV_Cov=0.202644;TOPMed_Count=0;Best_TOPMed_ID=DUP_19:4084740-4226078;Best_TOPMed_OFP=0.255974;Best_TOPMed_AF=4e-06;Best_TOPMed_Het=1;Best_TOPMed_HomAlt=0;Best_TOPMed_PopMax_AF=6.35898e-06;Best_TOPMed_Mismatch_ID=INV_19:3851264-4364229;Best_TOPMed_Mismatch_OFP=0.30055;Best_TOPMed_Mismatch_SVTYPE=INV;Best_TOPMed_Mismatch_AF=4e-06;Best_TOPMed_Mismatch_Het=1;Best_TOPMed_Mismatch_HomAlt=0;TOPMed_SV_Cov=0.741834;ThousG_Count=0;Best_ThousG_ID=HGSV_70075;Best_ThousG_OFP=0.14813;Best_ThousG_AF=0.000312;Best_ThousG_Het=2;Best_ThousG_HomAlt=0;Best_ThousG_PopMax_AF=0.002041;Best_ThousG_Mismatch_ID=HGSV_70074;Best_ThousG_Mismatch_OFP=0.0204771;Best_ThousG_Mismatch_SVTYPE=DEL;Best_ThousG_Mismatch_AF=0.000312;Best_ThousG_Mismatch_Het=2;Best_ThousG_Mismatch_HomAlt=0;ThousG_SV_Cov=0.285162;gnomAD_Count=0;Best_gnomAD_ID=gnomAD-SV_v3_DUP_chr19_9114b2c5;Best_gnomAD_OFP=0.36671;Best_gnomAD_AF=8e-06;Best_gnomAD_Het=1;Best_gnomAD_HomAlt=0;Best_gnomAD_PopMax_AF=7.9e-05;Best_gnomAD_Mismatch_ID=gnomAD-SV_v3_DEL_chr19_5ba69aa0;Best_gnomAD_Mismatch_OFP=0.172029;Best_gnomAD_Mismatch_SVTYPE=DEL;Best_gnomAD_Mismatch_AF=6.3e-05;Best_gnomAD_Mismatch_Het=8;Best_gnomAD_Mismatch_HomAlt=0;gnomAD_SV_Cov=0.960771	GT:PSV:LN:DR:ST:QV:TY:ID:RAL:AAL:CO	0/1:NA:154170:21,8:-:600:DUP:DUP00054229:NA:NA:chr19_4005253-chr19_4159423	0/1:NA:154171:0,0:-:999:DUP:MantaDUP_TANDEM_79083_0_1_1_0_0:NA:NA:chr19_4005252-chr19_4159423	0/1:NA:154211:0,0:-:106:DUP:10045:NA:NA:chr19_4005230-chr19_4159441	split	p13.3	MAP2K2				NM_030662	4	4090320	4124122	33802	1203	100	no	11	txStart-txEnd	5'UTR-3'UTR			4090320	4124122						Cardiofaciocutaneous syndrome 4, 615280 (3) AD		morbid:MAP2K2	19:4090321-4124122															11	Cardio-facio-cutaneous_syndrome;Cardiofaciocutaneous_syndrome_1;Cardiofaciocutaneous_syndrome_4;Castleman-Kojima_disease;Gastric_adenocarcinoma;Malignant_melanoma_of_skin;Melanoma;Noonan_syndrome;Noonan_syndrome_1;Pancreatic_adenocarcinoma;RASopathy;Smith-Magenis_Syndrome-like;Squamous_cell_carcinoma_of_the_head_and_neck										IMH	19:1692676-41507766	0.0103																										38.94	0.37913	0.438659	0.549595	-1.31922	1.476321	Noonan syndrome;cardiofaciocutaneous syndrome;cardiofaciocutaneous syndrome 1;cardiofaciocutaneous syndrome 4;neurofibromatosis-Noonan syndrome	AD	Definitive;Limited;Strong;Supportive	16439621;18042262;20301365[PMID]_16825433[PMID];20358587;21396583;23379592;24311457[PMID]	5605	601263	Cardiofaciocutaneous syndrome 4, 615280 (3) AD	AD	yes		1	9.67E-01	8.63E-01	0.6168	Cardiofaciocutaneous syndrome;Cleft lip;Cleft palate;High palate;Hypertelorism;Hypoplasia of the zygomatic bone;Micrognathia;Submucous cleft hard palate					full=3	0/1
15_99687009_99687107_DEL_1	15	99687009	99687107	-97	DEL	ILS_AP001,ILS_AP001_1,ILS_AP001_2	MantaDEL:71235:0:1:0:0:0	CTGCCTCCTGGGCTCACCTCAGCCTCCTTAGTTTCTGGGACTACAGGTGCACACCACCATGTCCTATTAATTTTTCTTTTTTTTTTTTTTTTTTTTTT	C	602	PASS	SUPP=3;SUPP_VEC=111;SVLEN=-97;SVTYPE=DEL;SVMETHOD=SURVIVOR1.0.7;CHR2=15;END=99687106;CIPOS=0,1;CIEND=0,1;STRANDS=+;Max_AF=0.000156;Max_Het=8;Max_HomAlt=0;Max_PopMax_AF=0.000832;CCDG_Count=0;Best_CCDG_Mismatch_ID=481461;Best_CCDG_Mismatch_OFP=0.000322503;Best_CCDG_Mismatch_SVTYPE=DUP;Best_CCDG_Mismatch_AF=3.419e-05;Best_CCDG_Mismatch_Het=0;Best_CCDG_Mismatch_HomAlt=0;SV_Cov=1;CCDG_SV_Cov=0;TOPMed_Count=0;Best_TOPMed_ID=DEL_15:93794810-100070222;Best_TOPMed_OFP=1.56165e-05;Best_TOPMed_AF=5.4e-05;Best_TOPMed_Het=13;Best_TOPMed_HomAlt=0;Best_TOPMed_PopMax_AF=0.000244559;Best_TOPMed_Mismatch_ID=INV_15:99320521-99873567;Best_TOPMed_Mismatch_OFP=0.0001772;Best_TOPMed_Mismatch_SVTYPE=INV;Best_TOPMed_Mismatch_AF=7e-06;Best_TOPMed_Mismatch_Het=2;Best_TOPMed_Mismatch_HomAlt=0;TOPMed_SV_Cov=1;ThousG_Count=1;Best_ThousG_ID=HGSV_53719;Best_ThousG_OFP=0.989796;Best_ThousG_AF=0.000156;Best_ThousG_Het=1;Best_ThousG_HomAlt=0;Best_ThousG_PopMax_AF=0.000832;ThousG_SV_Cov=0.989796;gnomAD_Count=1;Best_gnomAD_ID=gnomAD-SV_v3_DEL_chr15_589928e5;Best_gnomAD_OFP=0.979696;Best_gnomAD_AF=6.4e-05;Best_gnomAD_Het=8;Best_gnomAD_HomAlt=0;Best_gnomAD_PopMax_AF=0.000454;Best_gnomAD_Mismatch_ID=gnomAD-SV_v3_DUP_chr15_c71511db;Best_gnomAD_Mismatch_OFP=0.000322503;Best_gnomAD_Mismatch_SVTYPE=DUP;Best_gnomAD_Mismatch_AF=1.6e-05;Best_gnomAD_Mismatch_Het=2;Best_gnomAD_Mismatch_HomAlt=0;gnomAD_SV_Cov=1	GT:PSV:LN:DR:ST:QV:TY:ID:RAL:AAL:CO	0/1:NA:98:0,0:+:480:DEL:DEL00045486:NA:NA:chr15_99687009-chr15_99687107	0/1:NA:97:0,0:+:602:DEL:MantaDEL_71235_0_1_0_0_0:CTGCCTCCTGGGCTCACCTCAGCCTCCTTAGTTTCTGGGACTACAGGTGCACACCACCATGTCCTATTAATTTTTCTTTTTTTTTTTTTTTTTTTTTT:C:chr15_99687009-chr15_99687106	0/1:NA:96:0,0:+:284:DEL:8640:NA:NA:chr15_99687010-chr15_99687106	split	q26.3	MEF2A				NM_001352617	4	99565416	99716488	99	0	0	no	11	intron7-intron7	CDS	3133	3'	99687008	99687107																										dbVar	chr15:99437793-99780795;chr15:99645280-99949158	0.01							IMH	15:52285432-100775019	0.2918																										6.04	0.108002	-0.74081	-0.49525	-0.919	0.53855					4205	600660	(Coronary artery disease, AD, 1), 608320 (3) AD	AD		yes	1	9.87E-01	7.37E-01	0						full=3	0/1
11_64341843_64341924_DEL_1	11	64341843	64341924	-80	DEL	ILS_AP001,ILS_AP001_1,ILS_AP001_2	MantaDEL:30764:0:1:0:0:0	GGTTGTGGTCTGAGGTCTTGGGCCATCAGTGATGTCACAACCAGATGGCCCAAGACCCCAGACCACAACCCCATGTCTGGT	G	480	PASS	SUPP=3;SUPP_VEC=111;SVLEN=-80;SVTYPE=DEL;SVMETHOD=SURVIVOR1.0.7;CHR2=11;END=64341923;CIPOS=0,8;CIEND=0,1;STRANDS=+;Max_AF=0.000519;Max_Het=65;Max_HomAlt=0;Max_PopMax_AF=0.000951;CCDG_Count=0;Best_CCDG_ID=166951;Best_CCDG_OFP=0.00442405;Best_CCDG_AF=3.419e-05;Best_CCDG_Het=0;Best_CCDG_HomAlt=0;Best_CCDG_PopMax_AF=0;Best_CCDG_Mismatch_ID=166953_2;Best_CCDG_Mismatch_OFP=0.0123457;Best_CCDG_Mismatch_SVTYPE=BND;Best_CCDG_Mismatch_AF=0.06302;Best_CCDG_Mismatch_Het=0;Best_CCDG_Mismatch_HomAlt=0;SV_Cov=1;CCDG_SV_Cov=1;TOPMed_Count=0;Best_TOPMed_ID=DEL_11:59466655-66168743;Best_TOPMed_OFP=1.20858e-05;Best_TOPMed_AF=4e-06;Best_TOPMed_Het=1;Best_TOPMed_HomAlt=0;Best_TOPMed_PopMax_AF=6.26354e-06;Best_TOPMed_Mismatch_ID=DUP_11:60405002-68536600;Best_TOPMed_Mismatch_OFP=9.96114e-06;Best_TOPMed_Mismatch_SVTYPE=DUP;Best_TOPMed_Mismatch_AF=4e-06;Best_TOPMed_Mismatch_Het=1;Best_TOPMed_Mismatch_HomAlt=0;TOPMed_SV_Cov=1;ThousG_Count=0;Best_ThousG_Mismatch_ID=HGSV_24190;Best_ThousG_Mismatch_OFP=2.06875e-06;Best_ThousG_Mismatch_SVTYPE=INV;Best_ThousG_Mismatch_AF=0.488601;Best_ThousG_Mismatch_Het=3129;Best_ThousG_Mismatch_HomAlt=0;ThousG_SV_Cov=0;gnomAD_Count=1;Best_gnomAD_ID=gnomAD-SV_v3_DEL_chr11_5ab5afbb;Best_gnomAD_OFP=0.901235;Best_gnomAD_AF=0.000519;Best_gnomAD_Het=65;Best_gnomAD_HomAlt=0;Best_gnomAD_PopMax_AF=0.000951;Best_gnomAD_Mismatch_ID=gnomAD-SV_v3_DUP_chr11_4e711591;Best_gnomAD_Mismatch_OFP=0.00309149;Best_gnomAD_Mismatch_SVTYPE=DUP;Best_gnomAD_Mismatch_AF=1.7e-05;Best_gnomAD_Mismatch_Het=0;Best_gnomAD_Mismatch_HomAlt=1;gnomAD_SV_Cov=1	GT:PSV:LN:DR:ST:QV:TY:ID:RAL:AAL:CO	0/1:NA:81:0,0:+:480:DEL:DEL00035331:NA:NA:chr11_64341843-chr11_64341924	0/1:NA:80:0,0:+:280:DEL:MantaDEL_30764_0_1_0_0_0:GGTTGTGGTCTGAGGTCTTGGGCCATCAGTGATGTCACAACCAGATGGCCCAAGACCCCAGACCACAACCCCATGTCTGGT:G:chr11_64341843-chr11_64341923	0/1:NA:72:0,0:+:197:DEL:6678:NA:NA:chr11_64341851-chr11_64341923	split	q13.1	MIR7155				NR_106977	1	64341848	64341904	56	0	0	no	1	txStart-txEnd	UTR			64341848	64341904																																			IMH	11:1621661-71538522;11:49193342-89651496;11:49211577-89635120;11:49243027-89597882;11:49358811-89347590;11:49403038-89297530;11:49586833-89037283;11:49629782-88995227;11:49736709-88892563	0.4958																																				1.02E+08									0						full=3	0/1
12_123221910_123222409_DEL_1	12	1.23E+08	1.23E+08	-498	DEL	ILS_AP001,ILS_AP001_1,ILS_AP001_2	MantaDEL:24599:0:1:2:0:0	TTATAGATTTGGGTAAGAAAGTATATTAAACATCATATTTTTATGACTGTTACAAGAATATCATGTTTCAAAATATGATGAGATATTATTCTAAAGATAATGTGATACTTTATCTTTAGTTTATAAACAAAATTGAGGCTGGTGGCCAGGCACAGTGGCTCACGCCTGTAATCCCAGCACTTTGGGAGGCCGAGGTGGGCAGATCACAAGGTCAGGAGCTCAAGACCAGCTTGGCCAACACGGTGAAACTCTGACTCTACTAAAAATTAGCCAGACACGGTGGTGCGCATCTGTAATTCCAGCTACTCAGGAGTCTGAGGCAGGAGAATTTCTTGAACCCAGGAGGCAGAGGTTGCAGTCAGCTAAAATCATGTCACTGCACGCCAGCCTGGGTGACAGAGTGAGACCTTGTCTCAAAAAAAAAAAAAAAAATTGATATAACGTATTGTTTTTTCTTACAAATATAAAAAAGGATGCTGGGTGTGGTGGCTCACGCCTG	T	1072	PASS	SUPP=3;SUPP_VEC=111;SVLEN=-498;SVTYPE=DEL;SVMETHOD=SURVIVOR1.0.7;CHR2=12;END=123222408;CIPOS=0,2;CIEND=0,1;STRANDS=+;Max_AF=0;Max_Het=0;Max_HomAlt=0;Max_PopMax_AF=0;CCDG_Count=0;Best_CCDG_ID=188912;Best_CCDG_OFP=0.0898775;Best_CCDG_AF=3.419e-05;Best_CCDG_Het=0;Best_CCDG_HomAlt=0;Best_CCDG_PopMax_AF=0;Best_CCDG_Mismatch_ID=473454;Best_CCDG_Mismatch_OFP=0.00533964;Best_CCDG_Mismatch_SVTYPE=DUP;Best_CCDG_Mismatch_AF=3.419e-05;Best_CCDG_Mismatch_Het=0;Best_CCDG_Mismatch_HomAlt=0;SV_Cov=1;CCDG_SV_Cov=1;TOPMed_Count=0;Best_TOPMed_Mismatch_ID=DUP_12:123175345-123268795;Best_TOPMed_Mismatch_OFP=0.00533964;Best_TOPMed_Mismatch_SVTYPE=DUP;Best_TOPMed_Mismatch_AF=1.8e-05;Best_TOPMed_Mismatch_Het=5;Best_TOPMed_Mismatch_HomAlt=0;TOPMed_SV_Cov=0;ThousG_Count=0;Best_ThousG_Mismatch_ID=HGSV_36639;Best_ThousG_Mismatch_OFP=0.0314054;Best_ThousG_Mismatch_SVTYPE=DUP;Best_ThousG_Mismatch_AF=0.001249;Best_ThousG_Mismatch_Het=8;Best_ThousG_Mismatch_HomAlt=0;ThousG_SV_Cov=0;gnomAD_Count=0;Best_gnomAD_ID=gnomAD-SV_v3_DEL_chr12_41af712a;Best_gnomAD_OFP=0.268201;Best_gnomAD_AF=4e-05;Best_gnomAD_Het=5;Best_gnomAD_HomAlt=0;Best_gnomAD_PopMax_AF=0.000643;Best_gnomAD_Mismatch_ID=gnomAD-SV_v3_DUP_chr12_c72d7304;Best_gnomAD_Mismatch_OFP=0.0314054;Best_gnomAD_Mismatch_SVTYPE=DUP;Best_gnomAD_Mismatch_AF=3.2e-05;Best_gnomAD_Mismatch_Het=4;Best_gnomAD_Mismatch_HomAlt=0;gnomAD_SV_Cov=1	GT:PSV:LN:DR:ST:QV:TY:ID:RAL:AAL:CO	0/1:NA:499:17,5:+:1072:DEL:DEL00039773:NA:NA:chr12_123221910-chr12_123222409	0/1:NA:498:0,0:+:970:DEL:MantaDEL_24599_0_1_2_0_0:TTATAGATTTGGGTAAGAAAGTATATTAAACATCATATTTTTATGACTGTTACAAGAATATCATGTTTCAAAATATGATGAGATATTATTCTAAAGATAATGTGATACTTTATCTTTAGTTTATAAACAAAATTGAGGCTGGTGGCCAGGCACAGTGGCTCACGCCTGTAATCCCAGCACTTTGGGAGGCCGAGGTGGGCAGATCACAAGGTCAGGAGCTCAAGACCAGCTTGGCCAACACGGTGAAACTCTGACTCTACTAAAAATTAGCCAGACACGGTGGTGCGCATCTGTAATTCCAGCTACTCAGGAGTCTGAGGCAGGAGAATTTCTTGAACCCAGGAGGCAGAGGTTGCAGTCAGCTAAAATCATGTCACTGCACGCCAGCCTGGGTGACAGAGTGAGACCTTGTCTCAAAAAAAAAAAAAAAAATTGATATAACGTATTGTTTTTTCTTACAAATATAAAAAAGGATGCTGGGTGTGGTGGCTCACGCCTG:T:chr12_123221910-chr12_123222408	0/1:NA:496:0,0:+:719:DEL:7478:NA:NA:chr12_123221912-chr12_123222408	split	q24.31	MPHOSPH9				XM_047428063	1	1.23E+08	1.23E+08	500	0	0	no	24	intron3-intron3	CDS	14	3'	1.23E+08	1.23E+08																										dbVar	chr12:123076425-123304379;chr12:123175344-123268795	0.01																																			47.37	-0.39718	-0.88773	-0.67925	1.085043	-0.1163					10198						3	1.66E-10	7.60E-04	0						full=3	0/1
13_29375409_29375645_DEL_1	13	29375409	29375645	-218	DEL	ILS_AP001,ILS_AP001_1,ILS_AP001_2	MantaDEL:8169:0:0:0:0:1	TTACTTACTTTTAATGGCAAAAACCGCAATTACTTACTTTTAATGGCAACAACCGCAATTACTTACTTTTAATGGCAAAAACCGCAATCACCTTTGCACCAGCCTACTATATATATATATATATATATACGTGTATATATATATATATACGTATATATATATATACATATATATATATACGTATATATATATATATATACGTATATATATATATATATA	TCACCTTTGCACCAACCTAC	999	PASS	SUPP=3;SUPP_VEC=111;SVLEN=-218;SVTYPE=DEL;SVMETHOD=SURVIVOR1.0.7;CHR2=13;END=29375627;CIPOS=0,126;CIEND=0,18;STRANDS=+;Max_AF=0;Max_Het=0;Max_HomAlt=0;Max_PopMax_AF=0;CCDG_Count=0;Best_CCDG_Mismatch_ID=31451_1;Best_CCDG_Mismatch_OFP=0.00456621;Best_CCDG_Mismatch_SVTYPE=BND;Best_CCDG_Mismatch_AF=0.1431;Best_CCDG_Mismatch_Het=0;Best_CCDG_Mismatch_HomAlt=0;SV_Cov=1;CCDG_SV_Cov=0;TOPMed_Count=0;Best_TOPMed_Mismatch_ID=DUP_13:29193402-29635100;Best_TOPMed_Mismatch_OFP=0.000495812;Best_TOPMed_Mismatch_SVTYPE=DUP;Best_TOPMed_Mismatch_AF=5.2e-05;Best_TOPMed_Mismatch_Het=14;Best_TOPMed_Mismatch_HomAlt=0;TOPMed_SV_Cov=0;ThousG_Count=0;ThousG_SV_Cov=0;gnomAD_Count=0;Best_gnomAD_ID=gnomAD-SV_v3_DEL_chr13_807ad7b8;Best_gnomAD_OFP=0.58451;Best_gnomAD_AF=0.00012;Best_gnomAD_Het=15;Best_gnomAD_HomAlt=0;Best_gnomAD_PopMax_AF=0.000952;Best_gnomAD_Mismatch_ID=gnomAD-SV_v3_BND_chr13_95491615;Best_gnomAD_Mismatch_OFP=0.00456621;Best_gnomAD_Mismatch_SVTYPE=BND;Best_gnomAD_Mismatch_AF=0.007648;Best_gnomAD_Mismatch_Het=871;Best_gnomAD_Mismatch_HomAlt=0;gnomAD_SV_Cov=1	GT:PSV:LN:DR:ST:QV:TY:ID:RAL:AAL:CO	1/1:NA:111:0,0:+:449:DEL:DEL00040360:NA:NA:chr13_29375534-chr13_29375645	1/1:NA:218:0,0:+:999:DEL:MantaDEL_8169_0_0_0_0_1:TTACTTACTTTTAATGGCAAAAACCGCAATTACTTACTTTTAATGGCAACAACCGCAATTACTTACTTTTAATGGCAAAAACCGCAATCACCTTTGCACCAGCCTACTATATATATATATATATATATACGTGTATATATATATATATACGTATATATATATATACATATATATATATACGTATATATATATATATATACGTATATATATATATATATA:TCACCTTTGCACCAACCTAC:chr13_29375409-chr13_29375627	1/1:NA:109:0,0:+:531:DEL:7674:NA:NA:chr13_29375535-chr13_29375644	split	q12.3	MTUS2				NM_001384605	1	28819962	29505947	237	0	0	no	16	intron8-intron8	CDS	15935	5'	29375408	29375645																																																															47.5	2.149185	0.994348	1.333913	-1.65367	-1.4549					23281						1	7.95E-01	3.31E-01	0						full=3	1/1
9_110765848_110766057_DEL_1	9	1.11E+08	1.11E+08	-208	DEL	ILS_AP001,ILS_AP001_1,ILS_AP001_2	MantaDEL:211911:0:1:0:0:0	ACAGGTGTGAGCCACCACACTCAGCCAAAGCAATCCATTTTTATGCTTAGGTTCAATGAAGTATGAACAGCTGTGTAGAAATATGATTAGACACAAAGGGTATGATCTAATGCTAACAGGCTGAGTGGGGAAACTAGCCCATCCTGCTTGTTCAGATTTTTCTCAGCCCCTCTGTGCAGCACTCACTCCTCCCAGGCAAGAGGTAGGAT	A	325	PASS	SUPP=3;SUPP_VEC=111;SVLEN=-208;SVTYPE=DEL;SVMETHOD=SURVIVOR1.0.7;CHR2=9;END=110766056;CIPOS=0,1;CIEND=0,1;STRANDS=+;Max_AF=0.0003419;Max_Het=30;Max_HomAlt=1;Max_PopMax_AF=0.003636;CCDG_Count=1;Best_CCDG_ID=435033;Best_CCDG_OFP=0.995215;Best_CCDG_AF=0.0003419;Best_CCDG_Het=0;Best_CCDG_HomAlt=0;Best_CCDG_PopMax_AF=0;Best_CCDG_Mismatch_ID=608311;Best_CCDG_Mismatch_OFP=7.767e-06;Best_CCDG_Mismatch_SVTYPE=INV;Best_CCDG_Mismatch_AF=3.419e-05;Best_CCDG_Mismatch_Het=0;Best_CCDG_Mismatch_HomAlt=0;SV_Cov=1;CCDG_SV_Cov=0.995215;TOPMed_Count=1;Best_TOPMed_ID=DEL_9:110765850-110766056;Best_TOPMed_OFP=0.995215;Best_TOPMed_AF=0.000123;Best_TOPMed_Het=30;Best_TOPMed_HomAlt=1;Best_TOPMed_PopMax_AF=0.00344418;TOPMed_SV_Cov=1;ThousG_Count=1;Best_ThousG_ID=HGSV_164623;Best_ThousG_OFP=0.995215;Best_ThousG_AF=0.000312;Best_ThousG_Het=2;Best_ThousG_HomAlt=0;Best_ThousG_PopMax_AF=0.001664;ThousG_SV_Cov=0.995215;gnomAD_Count=1;Best_gnomAD_ID=gnomAD-SV_v3_DEL_chr9_c7517a2c;Best_gnomAD_OFP=0.995215;Best_gnomAD_AF=0.000127;Best_gnomAD_Het=14;Best_gnomAD_HomAlt=1;Best_gnomAD_PopMax_AF=0.003636;Best_gnomAD_Mismatch_ID=gnomAD-SV_v3_DUP_chr9_005c44cc;Best_gnomAD_Mismatch_OFP=0.000567588;Best_gnomAD_Mismatch_SVTYPE=DUP;Best_gnomAD_Mismatch_AF=1.6e-05;Best_gnomAD_Mismatch_Het=2;Best_gnomAD_Mismatch_HomAlt=0;gnomAD_SV_Cov=1	GT:PSV:LN:DR:ST:QV:TY:ID:RAL:AAL:CO	0/1:NA:209:0,0:+:145:DEL:DEL00029056:NA:NA:chr9_110765848-chr9_110766057	0/1:NA:208:0,0:+:325:DEL:MantaDEL_211911_0_1_0_0_0:ACAGGTGTGAGCCACCACACTCAGCCAAAGCAATCCATTTTTATGCTTAGGTTCAATGAAGTATGAACAGCTGTGTAGAAATATGATTAGACACAAAGGGTATGATCTAATGCTAACAGGCTGAGTGGGGAAACTAGCCCATCCTGCTTGTTCAGATTTTTCTCAGCCCCTCTGTGCAGCACTCACTCCTCCCAGGCAAGAGGTAGGAT:A:chr9_110765848-chr9_110766056	0/1:NA:207:0,0:+:185:DEL:5707:NA:NA:chr9_110765849-chr9_110766056	split	q31.3	MUSK				NM_001166280	2	1.11E+08	1.11E+08	210	0	0	no	14	intron9-intron9	CDS	3639	5'	1.11E+08	1.11E+08																																			IMH	9:65437055-134286391;9:80897170-125694547;9:89671548-130954998	0.4714																										6.69	-2.62354	-0.96266	-2.19452	0.604569	0.165293	congenital myasthenic syndrome 9;fetal akinesia deformation sequence 1;postsynaptic congenital myasthenic syndrome	AR	Strong;Supportive	15496425;19949040;20301347[PMID];20371544;24122059;25537362;25612909;25612909[PMID]_25537362[PMID];25695962;25900532;32253145;8653786	4593	601296	Fetal akinesia deformation sequence 1, 208150 (3) AR;Myasthenic syndrome, congenital, 9, associated with acetylcholine receptor deficiency, 616325 (3) AR	AR	yes		3	4.25E-08	8.17E-05	0.7065	Cleft lip;Cleft palate;Fetal akinesia deformation sequence 1;Hypertelorism;Micrognathia	Holoprosencephaly;abnormal innervation	Holoprosencephaly;spinal cord motor neuron differentiation process quality, abnormal			full=3	0/1
11_19982066_19983779_DEL_1	11	19982066	19983779	-1712	DEL	ILS_AP001,ILS_AP001_1,ILS_AP001_2	MantaDEL:27470:0:1:0:0:0	A		1260	PASS	SUPP=3;SUPP_VEC=111;SVLEN=-1712;SVTYPE=DEL;SVMETHOD=SURVIVOR1.0.7;CHR2=11;END=19983778;CIPOS=0,3;CIEND=0,1;STRANDS=+;Max_AF=0.002966;Max_Het=51;Max_HomAlt=2;Max_PopMax_AF=0.015807;CCDG_Count=0;Best_CCDG_ID=161160;Best_CCDG_OFP=0.0656246;Best_CCDG_AF=3.419e-05;Best_CCDG_Het=0;Best_CCDG_HomAlt=0;Best_CCDG_PopMax_AF=0;Best_CCDG_Mismatch_ID=161162_1;Best_CCDG_Mismatch_OFP=0.000583771;Best_CCDG_Mismatch_SVTYPE=BND;Best_CCDG_Mismatch_AF=0.0003761;Best_CCDG_Mismatch_Het=0;Best_CCDG_Mismatch_HomAlt=0;SV_Cov=1;CCDG_SV_Cov=1;TOPMed_Count=0;Best_TOPMed_ID=DEL_11:19971227-19997332;Best_TOPMed_OFP=0.0656146;Best_TOPMed_AF=4e-06;Best_TOPMed_Het=1;Best_TOPMed_HomAlt=0;Best_TOPMed_PopMax_AF=6.26049e-06;Best_TOPMed_Mismatch_ID=INV_11:16966271-21356221;Best_TOPMed_Mismatch_OFP=0.000390209;Best_TOPMed_Mismatch_SVTYPE=INV;Best_TOPMed_Mismatch_AF=1.1e-05;Best_TOPMed_Mismatch_Het=3;Best_TOPMed_Mismatch_HomAlt=0;TOPMed_SV_Cov=1;ThousG_Count=1;Best_ThousG_ID=HGSV_22181;Best_ThousG_OFP=1;Best_ThousG_AF=0.002966;Best_ThousG_Het=17;Best_ThousG_HomAlt=1;Best_ThousG_PopMax_AF=0.015807;Best_ThousG_Mismatch_ID=HGSV_21984;Best_ThousG_Mismatch_OFP=0.000401453;Best_ThousG_Mismatch_SVTYPE=DUP;Best_ThousG_Mismatch_AF=0.000156;Best_ThousG_Mismatch_Het=1;Best_ThousG_Mismatch_HomAlt=0;ThousG_SV_Cov=1;gnomAD_Count=1;Best_gnomAD_ID=gnomAD-SV_v3_DEL_chr11_753e4dc0;Best_gnomAD_OFP=0.940176;Best_gnomAD_AF=0.000436;Best_gnomAD_Het=51;Best_gnomAD_HomAlt=2;Best_gnomAD_PopMax_AF=0.012483;Best_gnomAD_Mismatch_ID=gnomAD-SV_v3_DUP_chr11_949f9834;Best_gnomAD_Mismatch_OFP=0.00482213;Best_gnomAD_Mismatch_SVTYPE=DUP;Best_gnomAD_Mismatch_AF=8e-06;Best_gnomAD_Mismatch_Het=1;Best_gnomAD_Mismatch_HomAlt=0;gnomAD_SV_Cov=1	GT:PSV:LN:DR:ST:QV:TY:ID:RAL:AAL:CO	0/1:NA:1710:24,12:+:1260:DEL:DEL00034364:NA:NA:chr11_19982069-chr11_19983779	0/1:NA:1712:0,0:+:598:DEL:MantaDEL_27470_0_1_0_0_0:NA:NA:chr11_19982066-chr11_19983778	0/1:NA:1712:0,0:+:464:DEL:6447:NA:NA:chr11_19982066-chr11_19983778	split	p15.1	NAV2				NM_001111018	2	19345235	20121601	1714	0	0	no	39	intron11-intron11	CDS	345	3'	19982065	19983779																													1000g;dbVar;gnomAD-SV_v3_DEL_chr11_f71dd9b5	chr11:8142635-35706287;chr11:19981743-19984278;chr11:19982013-19983809;11:19982014-19983809	0.048				IMH	11:1621661-71538522	0.0179																										4.74	1.246539	0.869153	1.12478	-0.79706	1.145418					89797						0	1.00E+00	9.98E-01	0.4815		Cleft lip;Cleft palate;Holoprosencephaly;Hypertelorism;Micrognathia;Microphthalmia;abnormal olfaction;abnormal optic nerve morphology				full=3	0/1
2_205740722_205740786_DEL_1	2	2.06E+08	2.06E+08	-63	DEL	ILS_AP001,ILS_AP001_1,ILS_AP001_2	MantaDEL:185092:0:1:0:0:0	TTCAACTCTCTGCAAACAGCATGACTGCACCACATGACCTTCCCAGAAAGACTGGCTCAAGGAC	T	600	PASS	SUPP=3;SUPP_VEC=111;SVLEN=-63;SVTYPE=DEL;SVMETHOD=SURVIVOR1.0.7;CHR2=2;END=205740785;CIPOS=0,4;CIEND=0,1;STRANDS=+;Max_AF=0.000781;Max_Het=22;Max_HomAlt=0;Max_PopMax_AF=0.004781;CCDG_Count=0;Best_CCDG_Mismatch_ID=286657_1;Best_CCDG_Mismatch_OFP=0.015625;Best_CCDG_Mismatch_SVTYPE=BND;Best_CCDG_Mismatch_AF=0.000171;Best_CCDG_Mismatch_Het=0;Best_CCDG_Mismatch_HomAlt=0;SV_Cov=1;CCDG_SV_Cov=0;TOPMed_Count=0;TOPMed_SV_Cov=0;ThousG_Count=1;Best_ThousG_ID=HGSV_86223;Best_ThousG_OFP=0.9375;Best_ThousG_AF=0.000781;Best_ThousG_Het=5;Best_ThousG_HomAlt=0;Best_ThousG_PopMax_AF=0.00416;Best_ThousG_Mismatch_ID=HGSV_83331;Best_ThousG_Mismatch_OFP=8.01915e-07;Best_ThousG_Mismatch_SVTYPE=CPX;Best_ThousG_Mismatch_AF=0.000156;Best_ThousG_Mismatch_Het=1;Best_ThousG_Mismatch_HomAlt=0;ThousG_SV_Cov=0.9375;gnomAD_Count=1;Best_gnomAD_ID=gnomAD-SV_v3_DEL_chr2_05698c28;Best_gnomAD_OFP=0.9375;Best_gnomAD_AF=0.000175;Best_gnomAD_Het=22;Best_gnomAD_HomAlt=0;Best_gnomAD_PopMax_AF=0.004781;Best_gnomAD_Mismatch_ID=gnomAD-SV_v3_DUP_chr2_6713af26;Best_gnomAD_Mismatch_OFP=0.00914155;Best_gnomAD_Mismatch_SVTYPE=DUP;Best_gnomAD_Mismatch_AF=1.6e-05;Best_gnomAD_Mismatch_Het=2;Best_gnomAD_Mismatch_HomAlt=0;gnomAD_SV_Cov=1	GT:PSV:LN:DR:ST:QV:TY:ID:RAL:AAL:CO	0/1:NA:64:0,0:+:600:DEL:DEL00008477:NA:NA:chr2_205740722-chr2_205740786	0/1:NA:63:0,0:+:495:DEL:MantaDEL_185092_0_1_0_0_0:TTCAACTCTCTGCAAACAGCATGACTGCACCACATGACCTTCCCAGAAAGACTGGCTCAAGGAC:T:chr2_205740722-chr2_205740785	0/1:NA:59:0,0:+:233:DEL:1732:NA:NA:chr2_205740726-chr2_205740785	split	q33.3	NRP2				NM_201264	2	2.06E+08	2.06E+08	65	0	0	no	9	intron8-intron8	CDS	58	5'	2.06E+08	2.06E+08																										dbVar	chr2:204935496-205936029	0.01	gnomAD-SV_v3_DEL_chr2_d8522047	chr2:148424217-233186659	0.0146				IMH	2:65359567-213949290;2:121478683-234514775;2:136890294-221566955;2:198679776-209089296	0.3179																										3.46	-0.16967	1.072076	0.636235	0.439149	1.349211					4753						2	8.07E-04	1.71E-03	0.584		Cleft lip;Cleft palate;Holoprosencephaly;Hypertelorism;Micrognathia;Microphthalmia;abnormal vomeronasal sensory neuron morphology;abnormal vomeronasal sensory neuron physiology;hydrocephaly				full=3	0/1
11_55597948_55678392_INV_1	11	55597948	55678392	80444	INV	ILS_AP001,ILS_AP001_1,ILS_AP001_2	MantaBND:29968:0:1:0:0:0:0	A	A]chr11:55678392]	704	PASS	SUPP=3;SUPP_VEC=111;SVLEN=80444;SVTYPE=INV;SVMETHOD=SURVIVOR1.0.7;CHR2=11;END=55678392;CIPOS=0,4;CIEND=0,0;STRANDS=;Max_AF=0;Max_Het=0;Max_HomAlt=0;Max_PopMax_AF=0;CCDG_Count=0;Best_CCDG_ID=554066;Best_CCDG_OFP=0.139484;Best_CCDG_AF=0.005471;Best_CCDG_Het=0;Best_CCDG_HomAlt=0;Best_CCDG_PopMax_AF=0;Best_CCDG_Mismatch_ID=165830;Best_CCDG_Mismatch_OFP=0.356791;Best_CCDG_Mismatch_SVTYPE=DEL;Best_CCDG_Mismatch_AF=3.419e-05;Best_CCDG_Mismatch_Het=0;Best_CCDG_Mismatch_HomAlt=0;SV_Cov=1;CCDG_SV_Cov=1;TOPMed_Count=0;TOPMed_SV_Cov=0;ThousG_Count=0;Best_ThousG_ID=HGSV_24190;Best_ThousG_OFP=0.00205457;Best_ThousG_AF=0.488601;Best_ThousG_Het=3129;Best_ThousG_HomAlt=0;Best_ThousG_PopMax_AF=0;ThousG_SV_Cov=1;gnomAD_Count=0;Best_gnomAD_ID=gnomAD-SV_v3_INV_chr11_19a01d73;Best_gnomAD_OFP=0.00148313;Best_gnomAD_AF=8e-06;Best_gnomAD_Het=1;Best_gnomAD_HomAlt=0;Best_gnomAD_PopMax_AF=1.7e-05;Best_gnomAD_Mismatch_ID=gnomAD-SV_v3_CPX_chr11_66eeb635;Best_gnomAD_Mismatch_OFP=0.00209769;Best_gnomAD_Mismatch_SVTYPE=CPX;Best_gnomAD_Mismatch_AF=0.310979;Best_gnomAD_Mismatch_Het=36260;Best_gnomAD_Mismatch_HomAlt=1476;gnomAD_SV_Cov=1	GT:PSV:LN:DR:ST:QV:TY:ID:RAL:AAL:CO	0/1:NA:80444:19,8::704:INV:INV00035040:NA:NA:chr11_55597948-chr11_55678392	0/1:NA:80444:0,0::563:INV:MantaBND_29968_0_1_0_0_0_0:NA:NA:chr11_55597948-chr11_55678392	0/1:NA:80440:0,0::173:INV:6621_1:NA:NA:chr11_55597952-chr11_55678392	split	q11	OR4C11				NM_001004700	3	55602359	55607645	5286	933	95	no	4	txStart-txEnd	5'UTR-3'UTR			55602359	55607645																										DDD:35907;dbVar;dgv144n27;dgv369e212;nsv517440	11:55572904-55836936;11:55592738-55693312;11:55593807-55706749;11:55595852-55686087;chr11:55597674-55664124;chr11:55600896-55659493	0.4343	1000g;DDD:35907;DDD:35910;HI40:ISCA-46458;dbVar;dgv145n27;dgv1915n54;dgv211e199;dgv368e212;esv2421634;esv2663268;esv2760216;esv2760311;esv3626445;esv3892006;nsv517440	11:55572904-55836936;chr11:55590350-55664455;11:55591985-55842207;11:55592738-55703518;11:55593807-55706749;11:55593886-55736880;11:55595852-55685520;11:55596800-55676539;chr11:55596800-55676539;11:55596917-55697316;11:55597770-55664316;11:55597959-55664091;11:55600414-55685447;11:55600414-55685447;11:55600414-55685533;chr11:55600896-55659493;11:55600897-55659493;11:55600897-55659493	0.4107				CMRI:2_pbsv.INV.1044;HPRC:pbsv.INV.4207;IMH	11:1621661-71538522;11:49193342-89651496;11:49211577-89635120;11:49243027-89597882;11:49358811-89347590;11:49403038-89297530;11:49586833-89037283;11:49629782-88995227;11:49736709-88892563;chr11:55597265-55667018;11:55597333-55667017	0.4958																										87.48				-1.12753	-3.29256					219429						9	5.60E-06	1.41E-06	0						full=NA	0/1
11_55597948_55678392_INV_1	11	55597948	55678392	80444	INV	ILS_AP001,ILS_AP001_1,ILS_AP001_2	MantaBND:29968:0:1:0:0:0:0	A	A]chr11:55678392]	704	PASS	SUPP=3;SUPP_VEC=111;SVLEN=80444;SVTYPE=INV;SVMETHOD=SURVIVOR1.0.7;CHR2=11;END=55678392;CIPOS=0,4;CIEND=0,0;STRANDS=;Max_AF=0;Max_Het=0;Max_HomAlt=0;Max_PopMax_AF=0;CCDG_Count=0;Best_CCDG_ID=554066;Best_CCDG_OFP=0.139484;Best_CCDG_AF=0.005471;Best_CCDG_Het=0;Best_CCDG_HomAlt=0;Best_CCDG_PopMax_AF=0;Best_CCDG_Mismatch_ID=165830;Best_CCDG_Mismatch_OFP=0.356791;Best_CCDG_Mismatch_SVTYPE=DEL;Best_CCDG_Mismatch_AF=3.419e-05;Best_CCDG_Mismatch_Het=0;Best_CCDG_Mismatch_HomAlt=0;SV_Cov=1;CCDG_SV_Cov=1;TOPMed_Count=0;TOPMed_SV_Cov=0;ThousG_Count=0;Best_ThousG_ID=HGSV_24190;Best_ThousG_OFP=0.00205457;Best_ThousG_AF=0.488601;Best_ThousG_Het=3129;Best_ThousG_HomAlt=0;Best_ThousG_PopMax_AF=0;ThousG_SV_Cov=1;gnomAD_Count=0;Best_gnomAD_ID=gnomAD-SV_v3_INV_chr11_19a01d73;Best_gnomAD_OFP=0.00148313;Best_gnomAD_AF=8e-06;Best_gnomAD_Het=1;Best_gnomAD_HomAlt=0;Best_gnomAD_PopMax_AF=1.7e-05;Best_gnomAD_Mismatch_ID=gnomAD-SV_v3_CPX_chr11_66eeb635;Best_gnomAD_Mismatch_OFP=0.00209769;Best_gnomAD_Mismatch_SVTYPE=CPX;Best_gnomAD_Mismatch_AF=0.310979;Best_gnomAD_Mismatch_Het=36260;Best_gnomAD_Mismatch_HomAlt=1476;gnomAD_SV_Cov=1	GT:PSV:LN:DR:ST:QV:TY:ID:RAL:AAL:CO	0/1:NA:80444:19,8::704:INV:INV00035040:NA:NA:chr11_55597948-chr11_55678392	0/1:NA:80444:0,0::563:INV:MantaBND_29968_0_1_0_0_0_0:NA:NA:chr11_55597948-chr11_55678392	0/1:NA:80440:0,0::173:INV:6621_1:NA:NA:chr11_55597952-chr11_55678392	split	q11	OR4C6				NM_001004704	2	55662200	55666096	3896	930	100	no	2	txStart-txEnd	5'UTR-3'UTR			55662200	55666096																										DDD:35907;dgv144n27;dgv369e212;dgv373e212;dgv382e212;nsv517440	11:55572904-55836936;11:55592738-55693312;11:55593807-55706749;11:55595852-55686087;11:55618742-55674829;11:55635326-55693312	0.4343	DDD:35907;DDD:35912;HI40:ISCA-46458;dbVar;dgv145n27;dgv1915n54;dgv211e199;dgv368e212;dgv377e212;dgv378e212;dgv381e212;dgv383e212;esv2421634;esv2760216;esv2760311;esv3892006;nsv514622;nsv517440	11:55572904-55836936;11:55591985-55842207;11:55592738-55703518;11:55593807-55706749;11:55593886-55736880;11:55595852-55685520;11:55596800-55676539;chr11:55596800-55676539;11:55596917-55697316;11:55600414-55685447;11:55600414-55685447;11:55600414-55685533;11:55606557-55682976;11:55626140-55685520;11:55635326-55667703;11:55635326-55674829;11:55635641-55683696;11:55646170-55674829;chr11:55654858-55836170	0.4529				CMRI:2_pbsv.INV.1044;HPRC:pbsv.INV.4207;IMH	11:1621661-71538522;11:49193342-89651496;11:49211577-89635120;11:49243027-89597882;11:49358811-89347590;11:49403038-89297530;11:49586833-89037283;11:49629782-88995227;11:49736709-88892563;chr11:55597265-55667018;11:55597333-55667017	0.4958																										90.42	-2.62354	-2.53125	-2.47163	-1.99765	-3.80719					219432						9	5.13E-03	1.08E-05	0						full=NA	0/1
11_55597948_55678392_INV_1	11	55597948	55678392	80444	INV	ILS_AP001,ILS_AP001_1,ILS_AP001_2	MantaBND:29968:0:1:0:0:0:0	A	A]chr11:55678392]	704	PASS	SUPP=3;SUPP_VEC=111;SVLEN=80444;SVTYPE=INV;SVMETHOD=SURVIVOR1.0.7;CHR2=11;END=55678392;CIPOS=0,4;CIEND=0,0;STRANDS=;Max_AF=0;Max_Het=0;Max_HomAlt=0;Max_PopMax_AF=0;CCDG_Count=0;Best_CCDG_ID=554066;Best_CCDG_OFP=0.139484;Best_CCDG_AF=0.005471;Best_CCDG_Het=0;Best_CCDG_HomAlt=0;Best_CCDG_PopMax_AF=0;Best_CCDG_Mismatch_ID=165830;Best_CCDG_Mismatch_OFP=0.356791;Best_CCDG_Mismatch_SVTYPE=DEL;Best_CCDG_Mismatch_AF=3.419e-05;Best_CCDG_Mismatch_Het=0;Best_CCDG_Mismatch_HomAlt=0;SV_Cov=1;CCDG_SV_Cov=1;TOPMed_Count=0;TOPMed_SV_Cov=0;ThousG_Count=0;Best_ThousG_ID=HGSV_24190;Best_ThousG_OFP=0.00205457;Best_ThousG_AF=0.488601;Best_ThousG_Het=3129;Best_ThousG_HomAlt=0;Best_ThousG_PopMax_AF=0;ThousG_SV_Cov=1;gnomAD_Count=0;Best_gnomAD_ID=gnomAD-SV_v3_INV_chr11_19a01d73;Best_gnomAD_OFP=0.00148313;Best_gnomAD_AF=8e-06;Best_gnomAD_Het=1;Best_gnomAD_HomAlt=0;Best_gnomAD_PopMax_AF=1.7e-05;Best_gnomAD_Mismatch_ID=gnomAD-SV_v3_CPX_chr11_66eeb635;Best_gnomAD_Mismatch_OFP=0.00209769;Best_gnomAD_Mismatch_SVTYPE=CPX;Best_gnomAD_Mismatch_AF=0.310979;Best_gnomAD_Mismatch_Het=36260;Best_gnomAD_Mismatch_HomAlt=1476;gnomAD_SV_Cov=1	GT:PSV:LN:DR:ST:QV:TY:ID:RAL:AAL:CO	0/1:NA:80444:19,8::704:INV:INV00035040:NA:NA:chr11_55597948-chr11_55678392	0/1:NA:80444:0,0::563:INV:MantaBND_29968_0_1_0_0_0_0:NA:NA:chr11_55597948-chr11_55678392	0/1:NA:80440:0,0::173:INV:6621_1:NA:NA:chr11_55597952-chr11_55678392	split	q11	OR4P4				NM_001405919	1	55635112	55640309	5197	939	48	no	2	txStart-txEnd	5'UTR-3'UTR			55635112	55640309																										DDD:35907;dbVar;dgv144n27;dgv369e212;dgv373e212;nsv517440	11:55572904-55836936;11:55592738-55693312;11:55593807-55706749;11:55595852-55686087;chr11:55597674-55664124;chr11:55600896-55659493;11:55618742-55674829	0.4343	1000g;DDD:35907;DDD:35910;DDD:35912;HI40:ISCA-46458;dbVar;dgv145n27;dgv1915n54;dgv211e199;dgv368e212;dgv377e212;esv2421634;esv2663268;esv2760216;esv2760311;esv3626445;esv3892006;nsv517440	11:55572904-55836936;chr11:55590350-55664455;11:55591985-55842207;11:55592738-55703518;11:55593807-55706749;11:55593886-55736880;11:55595852-55685520;11:55596800-55676539;chr11:55596800-55676539;11:55596917-55697316;11:55597770-55664316;11:55597959-55664091;11:55600414-55685447;11:55600414-55685447;11:55600414-55685533;chr11:55600896-55659493;11:55600897-55659493;11:55600897-55659493;11:55606557-55682976;11:55626140-55685520	0.4529				CMRI:2_pbsv.INV.1044;HPRC:pbsv.INV.4207;IMH	11:1621661-71538522;11:49193342-89651496;11:49211577-89635120;11:49243027-89597882;11:49358811-89347590;11:49403038-89297530;11:49586833-89037283;11:49629782-88995227;11:49736709-88892563;chr11:55597265-55667018;11:55597333-55667017	0.4958																										87.96				-0.20623	-1.30402					81300						8	1.76E-02	5.74E-02	0						full=NA	0/1
11_55597948_55678392_INV_1	11	55597948	55678392	80444	INV	ILS_AP001,ILS_AP001_1,ILS_AP001_2	MantaBND:29968:0:1:0:0:0:0	A	A]chr11:55678392]	704	PASS	SUPP=3;SUPP_VEC=111;SVLEN=80444;SVTYPE=INV;SVMETHOD=SURVIVOR1.0.7;CHR2=11;END=55678392;CIPOS=0,4;CIEND=0,0;STRANDS=;Max_AF=0;Max_Het=0;Max_HomAlt=0;Max_PopMax_AF=0;CCDG_Count=0;Best_CCDG_ID=554066;Best_CCDG_OFP=0.139484;Best_CCDG_AF=0.005471;Best_CCDG_Het=0;Best_CCDG_HomAlt=0;Best_CCDG_PopMax_AF=0;Best_CCDG_Mismatch_ID=165830;Best_CCDG_Mismatch_OFP=0.356791;Best_CCDG_Mismatch_SVTYPE=DEL;Best_CCDG_Mismatch_AF=3.419e-05;Best_CCDG_Mismatch_Het=0;Best_CCDG_Mismatch_HomAlt=0;SV_Cov=1;CCDG_SV_Cov=1;TOPMed_Count=0;TOPMed_SV_Cov=0;ThousG_Count=0;Best_ThousG_ID=HGSV_24190;Best_ThousG_OFP=0.00205457;Best_ThousG_AF=0.488601;Best_ThousG_Het=3129;Best_ThousG_HomAlt=0;Best_ThousG_PopMax_AF=0;ThousG_SV_Cov=1;gnomAD_Count=0;Best_gnomAD_ID=gnomAD-SV_v3_INV_chr11_19a01d73;Best_gnomAD_OFP=0.00148313;Best_gnomAD_AF=8e-06;Best_gnomAD_Het=1;Best_gnomAD_HomAlt=0;Best_gnomAD_PopMax_AF=1.7e-05;Best_gnomAD_Mismatch_ID=gnomAD-SV_v3_CPX_chr11_66eeb635;Best_gnomAD_Mismatch_OFP=0.00209769;Best_gnomAD_Mismatch_SVTYPE=CPX;Best_gnomAD_Mismatch_AF=0.310979;Best_gnomAD_Mismatch_Het=36260;Best_gnomAD_Mismatch_HomAlt=1476;gnomAD_SV_Cov=1	GT:PSV:LN:DR:ST:QV:TY:ID:RAL:AAL:CO	0/1:NA:80444:19,8::704:INV:INV00035040:NA:NA:chr11_55597948-chr11_55678392	0/1:NA:80444:0,0::563:INV:MantaBND_29968_0_1_0_0_0_0:NA:NA:chr11_55597948-chr11_55678392	0/1:NA:80440:0,0::173:INV:6621_1:NA:NA:chr11_55597952-chr11_55678392	split	q11	OR4S2				NM_001004059	3	55648326	55652854	4528	936	47	no	2	txStart-txEnd	5'UTR-3'UTR			55648326	55652854																										DDD:35907;dbVar;dgv144n27;dgv369e212;dgv373e212;dgv382e212;nsv517440	11:55572904-55836936;11:55592738-55693312;11:55593807-55706749;11:55595852-55686087;chr11:55597674-55664124;chr11:55600896-55659493;11:55618742-55674829;11:55635326-55693312	0.4343	1000g;DDD:35907;DDD:35910;DDD:35912;HI40:ISCA-46458;dbVar;dgv145n27;dgv1915n54;dgv211e199;dgv368e212;dgv377e212;dgv378e212;dgv381e212;dgv383e212;esv2421634;esv2663268;esv2760216;esv2760311;esv3626445;esv3892006;nsv514622;nsv517440	11:55572904-55836936;chr11:55590350-55664455;11:55591985-55842207;11:55592738-55703518;11:55593807-55706749;11:55593886-55736880;11:55595852-55685520;11:55596800-55676539;chr11:55596800-55676539;11:55596917-55697316;11:55597770-55664316;11:55597959-55664091;11:55600414-55685447;11:55600414-55685447;11:55600414-55685533;chr11:55600896-55659493;11:55600897-55659493;11:55600897-55659493;11:55606557-55682976;11:55626140-55685520;11:55635326-55667703;11:55635326-55674829;11:55635641-55683696;11:55646170-55674829	0.4529				CMRI:2_pbsv.INV.1044;HPRC:pbsv.INV.4207;IMH	11:1621661-71538522;11:49193342-89651496;11:49211577-89635120;11:49243027-89597882;11:49358811-89347590;11:49403038-89297530;11:49586833-89037283;11:49629782-88995227;11:49736709-88892563;chr11:55597265-55667018;11:55597333-55667017	0.4958																										79.58				1.165	-0.93491					219431						9	4.74E-10	1.04E-03	0						full=NA	0/1
20_17303439_17303526_DEL_1	20	17303439	17303526	-84	DEL	ILS_AP001,ILS_AP001_1,ILS_AP001_2	MantaDEL:97657:0:1:0:0:0	AATATATATTATATTAAATATAATATATATTATATATAATATATATTATATTTAATATAATATATATTATATATAATATAATATA	AT	415	PASS	SUPP=3;SUPP_VEC=111;SVLEN=-84;SVTYPE=DEL;SVMETHOD=SURVIVOR1.0.7;CHR2=20;END=17303526;CIPOS=-3,9;CIEND=-10,0;STRANDS=+;Max_AF=0;Max_Het=0;Max_HomAlt=0;Max_PopMax_AF=0;CCDG_Count=0;Best_CCDG_ID=293077;Best_CCDG_OFP=0.0182639;Best_CCDG_AF=0.0001026;Best_CCDG_Het=0;Best_CCDG_HomAlt=0;Best_CCDG_PopMax_AF=0;Best_CCDG_Mismatch_ID=293079_1;Best_CCDG_Mismatch_OFP=0.0117647;Best_CCDG_Mismatch_SVTYPE=BND;Best_CCDG_Mismatch_AF=0.227;Best_CCDG_Mismatch_Het=0;Best_CCDG_Mismatch_HomAlt=0;SV_Cov=1;CCDG_SV_Cov=1;TOPMed_Count=0;Best_TOPMed_Mismatch_ID=DUP_20:17274064-17318502;Best_TOPMed_Mismatch_OFP=0.00191269;Best_TOPMed_Mismatch_SVTYPE=DUP;Best_TOPMed_Mismatch_AF=7e-06;Best_TOPMed_Mismatch_Het=2;Best_TOPMed_Mismatch_HomAlt=0;TOPMed_SV_Cov=0;ThousG_Count=0;Best_ThousG_Mismatch_ID=HGSV_89585;Best_ThousG_Mismatch_OFP=0.000140685;Best_ThousG_Mismatch_SVTYPE=DUP;Best_ThousG_Mismatch_AF=0.000156;Best_ThousG_Mismatch_Het=1;Best_ThousG_Mismatch_HomAlt=0;ThousG_SV_Cov=0;gnomAD_Count=0;Best_gnomAD_ID=gnomAD-SV_v3_DEL_chr20_94becb03;Best_gnomAD_OFP=0.729412;Best_gnomAD_AF=0.04733;Best_gnomAD_Het=4091;Best_gnomAD_HomAlt=1;Best_gnomAD_PopMax_AF=0.166667;gnomAD_Mismatches=gnomAD-SV_v3_DUP_chr20_91afbc86;gnomAD_Mismatches_Count=1;gnomAD_Mismatch_SVTYPEs=DUP;Best_gnomAD_Mismatch_ID=gnomAD-SV_v3_DUP_chr20_91afbc86;Best_gnomAD_Mismatch_OFP=0.835294;Best_gnomAD_Mismatch_SVTYPE=DUP;Best_gnomAD_Mismatch_AF=0.036155;Best_gnomAD_Mismatch_Het=4531;Best_gnomAD_Mismatch_HomAlt=9;gnomAD_SV_Cov=1	GT:PSV:LN:DR:ST:QV:TY:ID:RAL:AAL:CO	0/1:NA:83:0,0:+:415:DEL:DEL00058353:NA:NA:chr20_17303439-chr20_17303522	0/1:NA:84:0,0:+:385:DEL:MantaDEL_97657_0_1_0_0_0:AATATATATTATATTAAATATAATATATATTATATATAATATATATTATATTTAATATAATATATATTATATATAATATAATATA:AT:chr20_17303442-chr20_17303526	0/1:NA:65:0,0:+:68:DEL:10795:NA:NA:chr20_17303451-chr20_17303516	split	p12.1	PCSK2				NM_001201528	2	17226106	17484578	88	0	0	no	13	intron3-intron3	CDS	43094	5'	17303438	17303526																										dbVar	chr20:17301743-17309639	0.01																																			10.35	0.305053	-0.37288	-0.14209	1.676153	3.923633					5126						0	9.99E-01	9.94E-01	0						full=3	0/1
17_8259210_8261028_INV_1	17	8259210	8261028	1818	INV	ILS_AP001,ILS_AP001_1,ILS_AP001_2	MantaBND:48483:0:1:0:0:0:1	T	T]chr17:8261028]	960	PASS	SUPP=3;SUPP_VEC=111;SVLEN=1818;SVTYPE=INV;SVMETHOD=SURVIVOR1.0.7;CHR2=17;END=8261028;CIPOS=0,6;CIEND=0,0;STRANDS=;Max_AF=0;Max_Het=0;Max_HomAlt=0;Max_PopMax_AF=0;CCDG_Count=0;CCDG_Mismatches=234321;CCDG_Mismatches_Count=1;CCDG_Mismatch_SVTYPEs=DEL;Best_CCDG_Mismatch_ID=234321;Best_CCDG_Mismatch_OFP=0.990654;Best_CCDG_Mismatch_SVTYPE=DEL;Best_CCDG_Mismatch_AF=0.0001368;Best_CCDG_Mismatch_Het=0;Best_CCDG_Mismatch_HomAlt=0;SV_Cov=1;CCDG_SV_Cov=0;TOPMed_Count=0;Best_TOPMed_ID=INV_17:8260934-8261004;Best_TOPMed_OFP=0.0395822;Best_TOPMed_AF=0.00015;Best_TOPMed_Het=42;Best_TOPMed_HomAlt=0;Best_TOPMed_PopMax_AF=0.0042735;TOPMed_Mismatches=DEL_17:8259218-8261018;TOPMed_Mismatches_Count=1;TOPMed_Mismatch_SVTYPEs=DEL;Best_TOPMed_Mismatch_ID=DEL_17:8259218-8261018;Best_TOPMed_Mismatch_OFP=0.990654;Best_TOPMed_Mismatch_SVTYPE=DEL;Best_TOPMed_Mismatch_AF=0.000124;Best_TOPMed_Mismatch_Het=32;Best_TOPMed_Mismatch_HomAlt=0;TOPMed_SV_Cov=1;ThousG_Count=0;Best_ThousG_ID=HGSV_59918;Best_ThousG_OFP=3.30527e-05;Best_ThousG_AF=0.000468;Best_ThousG_Het=3;Best_ThousG_HomAlt=0;Best_ThousG_PopMax_AF=0;ThousG_Mismatches=HGSV_60141;ThousG_Mismatches_Count=1;ThousG_Mismatch_SVTYPEs=DEL;Best_ThousG_Mismatch_ID=HGSV_60141;Best_ThousG_Mismatch_OFP=0.991204;Best_ThousG_Mismatch_SVTYPE=DEL;Best_ThousG_Mismatch_AF=0.000937;Best_ThousG_Mismatch_Het=6;Best_ThousG_Mismatch_HomAlt=0;ThousG_SV_Cov=1;gnomAD_Count=0;Best_gnomAD_ID=gnomAD-SV_v3_INV_chr17_02b6b8d7;Best_gnomAD_OFP=0.000256028;Best_gnomAD_AF=1.6e-05;Best_gnomAD_Het=2;Best_gnomAD_HomAlt=0;Best_gnomAD_PopMax_AF=3e-05;gnomAD_Mismatches=gnomAD-SV_v3_DEL_chr17_fc0648d6,gnomAD-SV_v3_DEL_chr17_f9c01235;gnomAD_Mismatches_Count=2;gnomAD_Mismatch_SVTYPEs=DEL;Best_gnomAD_Mismatch_ID=gnomAD-SV_v3_DEL_chr17_fc0648d6;Best_gnomAD_Mismatch_OFP=0.965366;Best_gnomAD_Mismatch_SVTYPE=DEL;Best_gnomAD_Mismatch_AF=0.000119;Best_gnomAD_Mismatch_Het=15;Best_gnomAD_Mismatch_HomAlt=0;gnomAD_SV_Cov=1	GT:PSV:LN:DR:ST:QV:TY:ID:RAL:AAL:CO	0/1:NA:1818:21,6::960:INV:INV00049625:NA:NA:chr17_8259210-chr17_8261028	0/1:NA:1818:0,0::367:INV:MantaBND_48483_0_1_0_0_0_1:NA:NA:chr17_8259210-chr17_8261028	0/1:NA:1812:0,0::242:INV:9205_1:NA:NA:chr17_8259216-chr17_8261028	split	p13.1	PFAS				XM_047436297	1	8247607	8266746	1819	0	0	no	23	intron11-intron11	CDS	1010	5'	8259209	8261028																										dbVar	chr17:8202867-8297966	0.01																																			47.86	-0.15132	0.255333	0.133119	-0.80046	0.774299					5198						4	9.85E-18	2.79E-08	0.9544		Cleft lip;Cleft palate;Hypertelorism;Micrognathia;Microphthalmia;alveolar process atrophy;microphthalmia;ocular hypertelorism;upturned snout				full=NA	0/1
19_4005348_4159440_DEL_1	19	4005348	4159440	-154090	DEL	ILS_AP001,ILS_AP001_1,ILS_AP001_2	MantaDEL:79083:0:1:0:0:0	C		1740	PASS	SUPP=3;SUPP_VEC=111;SVLEN=-154090;SVTYPE=DEL;SVMETHOD=SURVIVOR1.0.7;CHR2=19;END=4159440;CIPOS=-2,0;CIEND=-5,0;STRANDS=+;Max_AF=0;Max_Het=0;Max_HomAlt=0;Max_PopMax_AF=0;CCDG_Count=0;Best_CCDG_ID=253950;Best_CCDG_OFP=0.113861;Best_CCDG_AF=6.839e-05;Best_CCDG_Het=0;Best_CCDG_HomAlt=0;Best_CCDG_PopMax_AF=0;Best_CCDG_Mismatch_ID=490213;Best_CCDG_Mismatch_OFP=0.150976;Best_CCDG_Mismatch_SVTYPE=DUP;Best_CCDG_Mismatch_AF=3.419e-05;Best_CCDG_Mismatch_Het=0;Best_CCDG_Mismatch_HomAlt=0;SV_Cov=0.735014;CCDG_SV_Cov=0.290815;TOPMed_Count=0;Best_TOPMed_ID=DEL_19:4130566-4146337;Best_TOPMed_OFP=0.102362;Best_TOPMed_AF=4e-06;Best_TOPMed_Het=1;Best_TOPMed_HomAlt=0;Best_TOPMed_PopMax_AF=0.000108719;Best_TOPMed_Mismatch_ID=INV_19:3851264-4364229;Best_TOPMed_Mismatch_OFP=0.300392;Best_TOPMed_Mismatch_SVTYPE=INV;Best_TOPMed_Mismatch_AF=4e-06;Best_TOPMed_Mismatch_Het=1;Best_TOPMed_Mismatch_HomAlt=0;TOPMed_SV_Cov=0.400108;ThousG_Count=0;Best_ThousG_ID=HGSV_70074;Best_ThousG_OFP=0.0204879;Best_ThousG_AF=0.000312;Best_ThousG_Het=2;Best_ThousG_HomAlt=0;Best_ThousG_PopMax_AF=0.00112;Best_ThousG_Mismatch_ID=HGSV_70075;Best_ThousG_Mismatch_OFP=0.148371;Best_ThousG_Mismatch_SVTYPE=DUP;Best_ThousG_Mismatch_AF=0.000312;Best_ThousG_Mismatch_Het=2;Best_ThousG_Mismatch_HomAlt=0;ThousG_SV_Cov=0.0925492;gnomAD_Count=0;Best_gnomAD_ID=gnomAD-SV_v3_DEL_chr19_5ba69aa0;Best_gnomAD_OFP=0.172119;Best_gnomAD_AF=6.3e-05;Best_gnomAD_Het=8;Best_gnomAD_HomAlt=0;Best_gnomAD_PopMax_AF=0.000493;Best_gnomAD_Mismatch_ID=gnomAD-SV_v3_DUP_chr19_9114b2c5;Best_gnomAD_Mismatch_OFP=0.367091;Best_gnomAD_Mismatch_SVTYPE=DUP;Best_gnomAD_Mismatch_AF=8e-06;Best_gnomAD_Mismatch_Het=1;Best_gnomAD_Mismatch_HomAlt=0;gnomAD_SV_Cov=0.733638	GT:PSV:LN:DR:ST:QV:TY:ID:RAL:AAL:CO	0/1:NA:154087:26,11:+:1740:DEL:DEL00054230:NA:NA:chr19_4005348-chr19_4159435	0/1:NA:154090:0,0:+:999:DEL:MantaDEL_79083_0_1_0_0_0:NA:NA:chr19_4005350-chr19_4159440	0/1:NA:154090:0,0:+:650:DEL:10046:NA:NA:chr19_4005350-chr19_4159440	split	p13.3	PIAS4				XM_011528060	3	4009947	4039386	29439	1590	100	no	11	txStart-txEnd	5'UTR-3'UTR			4009947	4039386																																			IMH	19:1692676-41507766	0.0103																										36.28	0.52138	0.896295	0.995383	-0.62518	3.255531					51588						0	1.00E+00	9.94E-01	0						full=5	0/1
19_4005230_4159441_DUP_1	19	4005230	4159441	154171	DUP	ILS_AP001,ILS_AP001_1,ILS_AP001_2	MantaDUP:TANDEM:79083:0:1:1:0:0	T		999	PASS	SUPP=3;SUPP_VEC=111;SVLEN=154171;SVTYPE=DUP;SVMETHOD=SURVIVOR1.0.7;CHR2=19;END=4159423;CIPOS=-22,1;CIEND=0,18;STRANDS=-;Max_AF=0;Max_Het=0;Max_HomAlt=0;Max_PopMax_AF=0;CCDG_Count=0;Best_CCDG_ID=490213;Best_CCDG_OFP=0.150896;Best_CCDG_AF=3.419e-05;Best_CCDG_Het=0;Best_CCDG_HomAlt=0;Best_CCDG_PopMax_AF=0;Best_CCDG_Mismatch_ID=253950;Best_CCDG_Mismatch_OFP=0.113801;Best_CCDG_Mismatch_SVTYPE=DEL;Best_CCDG_Mismatch_AF=6.839e-05;Best_CCDG_Mismatch_Het=0;Best_CCDG_Mismatch_HomAlt=0;SV_Cov=0.97591;CCDG_SV_Cov=0.202644;TOPMed_Count=0;Best_TOPMed_ID=DUP_19:4084740-4226078;Best_TOPMed_OFP=0.255974;Best_TOPMed_AF=4e-06;Best_TOPMed_Het=1;Best_TOPMed_HomAlt=0;Best_TOPMed_PopMax_AF=6.35898e-06;Best_TOPMed_Mismatch_ID=INV_19:3851264-4364229;Best_TOPMed_Mismatch_OFP=0.30055;Best_TOPMed_Mismatch_SVTYPE=INV;Best_TOPMed_Mismatch_AF=4e-06;Best_TOPMed_Mismatch_Het=1;Best_TOPMed_Mismatch_HomAlt=0;TOPMed_SV_Cov=0.741834;ThousG_Count=0;Best_ThousG_ID=HGSV_70075;Best_ThousG_OFP=0.14813;Best_ThousG_AF=0.000312;Best_ThousG_Het=2;Best_ThousG_HomAlt=0;Best_ThousG_PopMax_AF=0.002041;Best_ThousG_Mismatch_ID=HGSV_70074;Best_ThousG_Mismatch_OFP=0.0204771;Best_ThousG_Mismatch_SVTYPE=DEL;Best_ThousG_Mismatch_AF=0.000312;Best_ThousG_Mismatch_Het=2;Best_ThousG_Mismatch_HomAlt=0;ThousG_SV_Cov=0.285162;gnomAD_Count=0;Best_gnomAD_ID=gnomAD-SV_v3_DUP_chr19_9114b2c5;Best_gnomAD_OFP=0.36671;Best_gnomAD_AF=8e-06;Best_gnomAD_Het=1;Best_gnomAD_HomAlt=0;Best_gnomAD_PopMax_AF=7.9e-05;Best_gnomAD_Mismatch_ID=gnomAD-SV_v3_DEL_chr19_5ba69aa0;Best_gnomAD_Mismatch_OFP=0.172029;Best_gnomAD_Mismatch_SVTYPE=DEL;Best_gnomAD_Mismatch_AF=6.3e-05;Best_gnomAD_Mismatch_Het=8;Best_gnomAD_Mismatch_HomAlt=0;gnomAD_SV_Cov=0.960771	GT:PSV:LN:DR:ST:QV:TY:ID:RAL:AAL:CO	0/1:NA:154170:21,8:-:600:DUP:DUP00054229:NA:NA:chr19_4005253-chr19_4159423	0/1:NA:154171:0,0:-:999:DUP:MantaDUP_TANDEM_79083_0_1_1_0_0:NA:NA:chr19_4005252-chr19_4159423	0/1:NA:154211:0,0:-:106:DUP:10045:NA:NA:chr19_4005230-chr19_4159441	split	p13.3	PIAS4				XM_011528060	3	4009947	4039386	29439	1590	100	no	11	txStart-txEnd	5'UTR-3'UTR			4009947	4039386																																			IMH	19:1692676-41507766	0.0103																										36.28	0.52138	0.896295	0.995383	-0.62518	3.255531					51588						0	1.00E+00	9.94E-01	0						full=3	0/1
2_46140344_46140465_DEL_1	2	46140344	46140465	-120	DEL	ILS_AP001,ILS_AP001_1,ILS_AP001_2	MantaDEL:174550:0:1:0:0:0	AATAAAAGATACAGGAAAAATCCAAGTGCACATGAGGATTTAGTACTAGTATTTGATAAAGGTGGTATTTCAATCAGTGAGGAAAAGATGACCTATTCAACGTAAGGGACTGGGACAACTT	A	720	PASS	SUPP=3;SUPP_VEC=111;SVLEN=-120;SVTYPE=DEL;SVMETHOD=SURVIVOR1.0.7;CHR2=2;END=46140464;CIPOS=0,1;CIEND=0,1;STRANDS=+;Max_AF=0;Max_Het=0;Max_HomAlt=0;Max_PopMax_AF=0;CCDG_Count=0;Best_CCDG_Mismatch_ID=573589;Best_CCDG_Mismatch_OFP=5.95697e-06;Best_CCDG_Mismatch_SVTYPE=INV;Best_CCDG_Mismatch_AF=0.024;Best_CCDG_Mismatch_Het=0;Best_CCDG_Mismatch_HomAlt=0;SV_Cov=1;CCDG_SV_Cov=0;TOPMed_Count=0;Best_TOPMed_ID=DEL_2:46099129-46208220;Best_TOPMed_OFP=0.00110915;Best_TOPMed_AF=4e-06;Best_TOPMed_Het=1;Best_TOPMed_HomAlt=0;Best_TOPMed_PopMax_AF=1.19801e-05;Best_TOPMed_Mismatch_ID=INV_2:39986389-47339194;Best_TOPMed_Mismatch_OFP=1.64563e-05;Best_TOPMed_Mismatch_SVTYPE=INV;Best_TOPMed_Mismatch_AF=2.2e-05;Best_TOPMed_Mismatch_Het=4;Best_TOPMed_Mismatch_HomAlt=1;TOPMed_SV_Cov=1;ThousG_Count=0;Best_ThousG_Mismatch_ID=HGSV_77475;Best_ThousG_Mismatch_OFP=9.02168e-07;Best_ThousG_Mismatch_SVTYPE=INS;Best_ThousG_Mismatch_AF=0.000468;Best_ThousG_Mismatch_Het=3;Best_ThousG_Mismatch_HomAlt=0;ThousG_SV_Cov=0;gnomAD_Count=0;Best_gnomAD_ID=gnomAD-SV_v3_DEL_chr2_7a56e34d;Best_gnomAD_OFP=5.94011e-06;Best_gnomAD_AF=0.00015;Best_gnomAD_Het=18;Best_gnomAD_HomAlt=0;Best_gnomAD_PopMax_AF=0.000719;Best_gnomAD_Mismatch_ID=gnomAD-SV_v3_INV_chr2_a005ba16;Best_gnomAD_Mismatch_OFP=7.13011e-06;Best_gnomAD_Mismatch_SVTYPE=INV;Best_gnomAD_Mismatch_AF=8e-06;Best_gnomAD_Mismatch_Het=1;Best_gnomAD_Mismatch_HomAlt=0;gnomAD_SV_Cov=1	GT:PSV:LN:DR:ST:QV:TY:ID:RAL:AAL:CO	0/1:NA:121:0,0:+:720:DEL:DEL00006104:NA:NA:chr2_46140344-chr2_46140465	0/1:NA:120:0,0:+:692:DEL:MantaDEL_174550_0_1_0_0_0:AATAAAAGATACAGGAAAAATCCAAGTGCACATGAGGATTTAGTACTAGTATTTGATAAAGGTGGTATTTCAATCAGTGAGGAAAAGATGACCTATTCAACGTAAGGGACTGGGACAACTT:A:chr2_46140344-chr2_46140464	0/1:NA:119:0,0:+:311:DEL:1234:NA:NA:chr2_46140345-chr2_46140464	split	p21	PRKCE				XM_005264428	2	45651278	46187990	122	0	0	no	16	intron12-intron12	CDS	4627	3'	46140343	46140465																																			IMH	2:21055811-76514955;2:32873521-53185851	0.1623																										5.17	0.65515	-0.35058	-0.00176	0.685911	4.857257					5581						0	1.00E+00	1.00E+00	0						full=3	0/1
6_161872925_161878005_DEL_1	6	1.62E+08	1.62E+08	-5079	DEL	ILS_AP001,ILS_AP001_1,ILS_AP001_2	MantaDEL:130517:0:1:0:0:0	A		1380	PASS	SUPP=3;SUPP_VEC=111;SVLEN=-5079;SVTYPE=DEL;SVMETHOD=SURVIVOR1.0.7;CHR2=6;END=161878004;CIPOS=0,3;CIEND=0,1;STRANDS=+;Max_AF=0.004215;Max_Het=183;Max_HomAlt=2;Max_PopMax_AF=0.022463;CCDG_Count=0;Best_CCDG_ID=386885;Best_CCDG_OFP=0.0131656;Best_CCDG_AF=3.419e-05;Best_CCDG_Het=0;Best_CCDG_HomAlt=0;Best_CCDG_PopMax_AF=0;Best_CCDG_Mismatch_ID=525230;Best_CCDG_Mismatch_OFP=0.0393058;Best_CCDG_Mismatch_SVTYPE=DUP;Best_CCDG_Mismatch_AF=6.839e-05;Best_CCDG_Mismatch_Het=0;Best_CCDG_Mismatch_HomAlt=0;SV_Cov=1;CCDG_SV_Cov=1;TOPMed_Count=1;Best_TOPMed_ID=DEL_6:161872929-161878008;Best_TOPMed_OFP=0.998623;Best_TOPMed_AF=0.00067;Best_TOPMed_Het=183;Best_TOPMed_HomAlt=2;Best_TOPMed_PopMax_AF=0.019193;Best_TOPMed_Mismatch_ID=INV_6:161869430-161880304;Best_TOPMed_Mismatch_OFP=0.467083;Best_TOPMed_Mismatch_SVTYPE=INV;Best_TOPMed_Mismatch_AF=4e-06;Best_TOPMed_Mismatch_Het=1;Best_TOPMed_Mismatch_HomAlt=0;TOPMed_SV_Cov=1;ThousG_Count=1;Best_ThousG_ID=HGSV_139376;Best_ThousG_OFP=0.999409;Best_ThousG_AF=0.004215;Best_ThousG_Het=27;Best_ThousG_HomAlt=0;Best_ThousG_PopMax_AF=0.022463;Best_ThousG_Mismatch_ID=HGSV_139355;Best_ThousG_Mismatch_OFP=0.00980807;Best_ThousG_Mismatch_SVTYPE=DUP;Best_ThousG_Mismatch_AF=0.000156;Best_ThousG_Mismatch_Het=1;Best_ThousG_Mismatch_HomAlt=0;ThousG_SV_Cov=1;gnomAD_Count=1;Best_gnomAD_ID=gnomAD-SV_v3_DEL_chr6_dabe8de7;Best_gnomAD_OFP=0.999409;Best_gnomAD_AF=0.001412;Best_gnomAD_Het=178;Best_gnomAD_HomAlt=0;Best_gnomAD_PopMax_AF=0.01802;Best_gnomAD_Mismatch_ID=gnomAD-SV_v3_CPX_chr6_d2bb65d8;Best_gnomAD_Mismatch_OFP=0.464096;Best_gnomAD_Mismatch_SVTYPE=CPX;Best_gnomAD_Mismatch_AF=8e-06;Best_gnomAD_Mismatch_Het=1;Best_gnomAD_Mismatch_HomAlt=0;gnomAD_SV_Cov=1	GT:PSV:LN:DR:ST:QV:TY:ID:RAL:AAL:CO	0/1:NA:5080:18,13:+:1380:DEL:DEL00021210:NA:NA:chr6_161872925-chr6_161878005	0/1:NA:5079:0,0:+:543:DEL:MantaDEL_130517_0_1_0_0_0:NA:NA:chr6_161872925-chr6_161878004	0/1:NA:5076:0,0:+:518:DEL:4201:NA:NA:chr6_161872928-chr6_161878004	split	q26	PRKN				XM_017010908	2	1.61E+08	1.62E+08	5081	0	0	no	12	intron6-intron6	CDS	87016	3'	1.62E+08	1.62E+08																										dbVar	chr6:161780967-162146968;chr6:161853183-162068074;chr6:161857468-161902968	0.01	dbVar	chr6:161747555-162202352;chr6:161872385-161878213	0.01																														30	0							Parkinson disease;autosomal recessive juvenile Parkinson disease 2;young-onset Parkinson disease	AR	Definitive;Strong;Supportive	22166458[PMID]	5071	602544	Adenocarcinoma of lung, somatic, 211980 (3);Ovarian cancer, somatic, 167000 (3);Parkinson disease, juvenile, type 2, 600116 (3) AR	AR	yes					0.3875		Holoprosencephaly;tau protein deposits				full=1	0/1
10_13652960_13653018_DEL_1	10	13652960	13653018	-57	DEL	ILS_AP001,ILS_AP001_1,ILS_AP001_2	MantaDEL:37558:0:0:0:0:0	ACAGAGGGCAGAGCTGGAATTCCAGCTCCCTCCAGCTGTCAGACTTTGGGCCAGGCAC	A	720	PASS	SUPP=3;SUPP_VEC=111;SVLEN=-57;SVTYPE=DEL;SVMETHOD=SURVIVOR1.0.7;CHR2=10;END=13653017;CIPOS=0,0;CIEND=0,1;STRANDS=+;Max_AF=0.00921;Max_Het=374;Max_HomAlt=11;Max_PopMax_AF=0.049085;CCDG_Count=0;Best_CCDG_Mismatch_ID=145531_1;Best_CCDG_Mismatch_OFP=0.0172414;Best_CCDG_Mismatch_SVTYPE=BND;Best_CCDG_Mismatch_AF=0.001368;Best_CCDG_Mismatch_Het=0;Best_CCDG_Mismatch_HomAlt=0;SV_Cov=1;CCDG_SV_Cov=0;TOPMed_Count=1;Best_TOPMed_ID=DEL_10:13652961-13653017;Best_TOPMed_OFP=1;Best_TOPMed_AF=0.001071;Best_TOPMed_Het=257;Best_TOPMed_HomAlt=6;Best_TOPMed_PopMax_AF=0.0312274;Best_TOPMed_Mismatch_ID=DUP_10:13580086-13654763;Best_TOPMed_Mismatch_OFP=0.000776657;Best_TOPMed_Mismatch_SVTYPE=DUP;Best_TOPMed_Mismatch_AF=1.4e-05;Best_TOPMed_Mismatch_Het=4;Best_TOPMed_Mismatch_HomAlt=0;TOPMed_SV_Cov=1;ThousG_Count=1;Best_ThousG_ID=HGSV_14079;Best_ThousG_OFP=1;Best_ThousG_AF=0.00921;Best_ThousG_Het=39;Best_ThousG_HomAlt=10;Best_ThousG_PopMax_AF=0.049085;ThousG_SV_Cov=1;gnomAD_Count=1;Best_gnomAD_ID=gnomAD-SV_v3_DEL_chr10_2288408b;Best_gnomAD_OFP=0.865672;Best_gnomAD_AF=0.003142;Best_gnomAD_Het=374;Best_gnomAD_HomAlt=11;Best_gnomAD_PopMax_AF=0.034968;Best_gnomAD_Mismatch_ID=gnomAD-SV_v3_DUP_chr10_f8fe9e87;Best_gnomAD_Mismatch_OFP=0.000776657;Best_gnomAD_Mismatch_SVTYPE=DUP;Best_gnomAD_Mismatch_AF=8e-06;Best_gnomAD_Mismatch_Het=1;Best_gnomAD_Mismatch_HomAlt=0;gnomAD_SV_Cov=1	GT:PSV:LN:DR:ST:QV:TY:ID:RAL:AAL:CO	0/1:NA:58:0,0:+:720:DEL:DEL00030300:NA:NA:chr10_13652960-chr10_13653018	0/1:NA:57:0,0:+:660:DEL:MantaDEL_37558_0_0_0_0_0:ACAGAGGGCAGAGCTGGAATTCCAGCTCCCTCCAGCTGTCAGACTTTGGGCCAGGCAC:A:chr10_13652960-chr10_13653017	0/1:NA:57:0,0:+:293:DEL:5923:NA:NA:chr10_13652960-chr10_13653017	split	p13	PRPF18				NR_172904	1	13586964	13655929	59	0	0	no	12	intron10-intron10	UTR	1334	3'	13652959	13653018																										dbVar	chr10:13543727-13697859;chr10:13580085-13654763	0.01																																			17.72	0.406129	-0.88084	-0.49222	-0.02436	2.440374					8559						4	2.68E-04	9.94E-02	0						full=3	0/1
11_1894044_1915726_DEL_1	11	1894044	1915726	-21681	DEL	ILS_AP001,ILS_AP001_1,ILS_AP001_2	MantaDEL:25948:0:1:0:0:0	G		830	PASS	SUPP=3;SUPP_VEC=111;SVLEN=-21681;SVTYPE=DEL;SVMETHOD=SURVIVOR1.0.7;CHR2=11;END=1915725;CIPOS=0,3;CIEND=0,1;STRANDS=+;Max_AF=0;Max_Het=0;Max_HomAlt=0;Max_PopMax_AF=0;CCDG_Count=0;Best_CCDG_ID=159323;Best_CCDG_OFP=0.312102;Best_CCDG_AF=3.419e-05;Best_CCDG_Het=0;Best_CCDG_HomAlt=0;Best_CCDG_PopMax_AF=0;Best_CCDG_Mismatch_ID=551838;Best_CCDG_Mismatch_OFP=0.000310112;Best_CCDG_Mismatch_SVTYPE=INV;Best_CCDG_Mismatch_AF=0.01792;Best_CCDG_Mismatch_Het=0;Best_CCDG_Mismatch_HomAlt=0;SV_Cov=1;CCDG_SV_Cov=0.341389;TOPMed_Count=0;Best_TOPMed_ID=DEL_11:1905428-1912864;Best_TOPMed_OFP=0.34305;Best_TOPMed_AF=1.8e-05;Best_TOPMed_Het=5;Best_TOPMed_HomAlt=0;Best_TOPMed_PopMax_AF=3.13138e-05;Best_TOPMed_Mismatch_ID=INV_11:1707647-1896713;Best_TOPMed_Mismatch_OFP=0.00173902;Best_TOPMed_Mismatch_SVTYPE=INV;Best_TOPMed_Mismatch_AF=2.9e-05;Best_TOPMed_Mismatch_Het=8;Best_TOPMed_Mismatch_HomAlt=0;TOPMed_SV_Cov=1;ThousG_Count=0;Best_ThousG_ID=HGSV_21180;Best_ThousG_OFP=0.018633;Best_ThousG_AF=0.005781;Best_ThousG_Het=37;Best_ThousG_HomAlt=0;Best_ThousG_PopMax_AF=0.01636;Best_ThousG_Mismatch_ID=HGSV_21179;Best_ThousG_Mismatch_OFP=0.331427;Best_ThousG_Mismatch_SVTYPE=DUP;Best_ThousG_Mismatch_AF=0.000156;Best_ThousG_Mismatch_Het=1;Best_ThousG_Mismatch_HomAlt=0;ThousG_SV_Cov=0.018633;gnomAD_Count=0;Best_gnomAD_ID=gnomAD-SV_v3_DEL_chr11_0a7bb213;Best_gnomAD_OFP=0.34305;Best_gnomAD_AF=8e-06;Best_gnomAD_Het=1;Best_gnomAD_HomAlt=0;Best_gnomAD_PopMax_AF=1.7e-05;Best_gnomAD_Mismatch_ID=gnomAD-SV_v3_DUP_chr11_5791e2d7;Best_gnomAD_Mismatch_OFP=0.330274;Best_gnomAD_Mismatch_SVTYPE=DUP;Best_gnomAD_Mismatch_AF=1.6e-05;Best_gnomAD_Mismatch_Het=2;Best_gnomAD_Mismatch_HomAlt=0;gnomAD_SV_Cov=0.620976	GT:PSV:LN:DR:ST:QV:TY:ID:RAL:AAL:CO	1/1:NA:21679:0,0:+:600:DEL:DEL00033993:NA:NA:chr11_1894047-chr11_1915726	1/1:NA:21681:0,0:+:830:DEL:MantaDEL_25948_0_1_0_0_0:NA:NA:chr11_1894044-chr11_1915725	1/1:NA:21681:0,0:+:827:DEL:6377:NA:NA:chr11_1894044-chr11_1915725	split	p15.5	PRR33				XM_047426236	1	1887986	1917708	21683	0	0	no	2	intron1-intron1	5'UTR	1912	5'	1894043	1915726																													1000g;HI40:ISCA-46652;dbVar;esv2666901;esv3625119	chr11:1894005-1915726;11:1894006-1915726;11:1894006-1915726;11:1894016-1915772;11:1894020-1915779	0.19				IMH	11:1621661-71538522	0.0179																										93.45										1.03E+08									0						full=1	1/1
22_44860912_44860969_DEL_1	22	44860912	44860969	-56	DEL	ILS_AP001,ILS_AP001_1,ILS_AP001_2	MantaDEL:95612:0:1:0:0:0	TTGGAGAATGTCACTTCTTGGTTTTACAACAATTTTTTTTCTCCCTGTTGCTTGACA	T	653	PASS	SUPP=3;SUPP_VEC=111;SVLEN=-56;SVTYPE=DEL;SVMETHOD=SURVIVOR1.0.7;CHR2=22;END=44860968;CIPOS=0,1;CIEND=0,1;STRANDS=+;Max_AF=0.000781;Max_Het=18;Max_HomAlt=0;Max_PopMax_AF=0.004274;CCDG_Count=0;Best_CCDG_ID=308768;Best_CCDG_OFP=0.00374237;Best_CCDG_AF=3.419e-05;Best_CCDG_Het=0;Best_CCDG_HomAlt=0;Best_CCDG_PopMax_AF=0;Best_CCDG_Mismatch_ID=504733;Best_CCDG_Mismatch_OFP=0.00542289;Best_CCDG_Mismatch_SVTYPE=DUP;Best_CCDG_Mismatch_AF=3.419e-05;Best_CCDG_Mismatch_Het=0;Best_CCDG_Mismatch_HomAlt=0;SV_Cov=1;CCDG_SV_Cov=1;TOPMed_Count=0;Best_TOPMed_ID=DEL_22:44858643-44861351;Best_TOPMed_OFP=0.0210332;Best_TOPMed_AF=4e-06;Best_TOPMed_Het=1;Best_TOPMed_HomAlt=0;Best_TOPMed_PopMax_AF=6.26535e-06;Best_TOPMed_Mismatch_ID=INV_22:44757699-44872616;Best_TOPMed_Mismatch_OFP=0.000496002;Best_TOPMed_Mismatch_SVTYPE=INV;Best_TOPMed_Mismatch_AF=4e-06;Best_TOPMed_Mismatch_Het=1;Best_TOPMed_Mismatch_HomAlt=0;TOPMed_SV_Cov=1;ThousG_Count=1;Best_ThousG_ID=HGSV_96575;Best_ThousG_OFP=0.982456;Best_ThousG_AF=0.000781;Best_ThousG_Het=5;Best_ThousG_HomAlt=0;Best_ThousG_PopMax_AF=0.004274;Best_ThousG_Mismatch_ID=HGSV_96574;Best_ThousG_Mismatch_OFP=0.00901471;Best_ThousG_Mismatch_SVTYPE=CPX;Best_ThousG_Mismatch_AF=0.000156;Best_ThousG_Mismatch_Het=1;Best_ThousG_Mismatch_HomAlt=0;ThousG_SV_Cov=0.982456;gnomAD_Count=1;Best_gnomAD_ID=gnomAD-SV_v3_DEL_chr22_e53f8373;Best_gnomAD_OFP=0.982456;Best_gnomAD_AF=0.000143;Best_gnomAD_Het=18;Best_gnomAD_HomAlt=0;Best_gnomAD_PopMax_AF=0.0042;Best_gnomAD_Mismatch_ID=gnomAD-SV_v3_DUP_chr22_abddc20b;Best_gnomAD_Mismatch_OFP=0.00712411;Best_gnomAD_Mismatch_SVTYPE=DUP;Best_gnomAD_Mismatch_AF=1.7e-05;Best_gnomAD_Mismatch_Het=2;Best_gnomAD_Mismatch_HomAlt=0;gnomAD_SV_Cov=1	GT:PSV:LN:DR:ST:QV:TY:ID:RAL:AAL:CO	0/1:NA:57:0,0:+:653:DEL:DEL00062432:NA:NA:chr22_44860912-chr22_44860969	1/1:NA:56:0,0:+:486:DEL:MantaDEL_95612_0_1_0_0_0:TTGGAGAATGTCACTTCTTGGTTTTACAACAATTTTTTTTCTCCCTGTTGCTTGACA:T:chr22_44860912-chr22_44860968	0/1:NA:55:0,0:+:272:DEL:11382:NA:NA:chr22_44860913-chr22_44860968	split	q13.31	PRR5-ARHGAP8				NM_181334	6	44702203	44862784	58	0	0	no	15	intron14-intron14	CDS	1077	5'	44860911	44860969																													dbVar	chr22:44820754-44869341;chr22:44837184-44861234	0.01				IMH	22:44479419-47655346	0.2479																										77.56	-2.62354	0.465669	-1.01906							553158						8	2.14E-28		0.5199		Cleft lip;Cleft palate;Hypertelorism;Micrognathia;Microphthalmia;cataract				full=3	0/1
8_128092107_128099360_DEL_1	8	1.28E+08	1.28E+08	-7252	DEL	ILS_AP001,ILS_AP001_1,ILS_AP001_2	MantaDEL:166772:1:2:0:0:0	C		1320	PASS	SUPP=3;SUPP_VEC=111;SVLEN=-7252;SVTYPE=DEL;SVMETHOD=SURVIVOR1.0.7;CHR2=8;END=128099359;CIPOS=0,0;CIEND=0,1;STRANDS=+;Max_AF=0.000156;Max_Het=27;Max_HomAlt=0;Max_PopMax_AF=0.00293606;CCDG_Count=0;Best_CCDG_Mismatch_ID=605159;Best_CCDG_Mismatch_OFP=0.000127629;Best_CCDG_Mismatch_SVTYPE=INV;Best_CCDG_Mismatch_AF=3.419e-05;Best_CCDG_Mismatch_Het=0;Best_CCDG_Mismatch_HomAlt=0;SV_Cov=1;CCDG_SV_Cov=0;TOPMed_Count=1;Best_TOPMed_ID=DEL_8:128092108-128099360;Best_TOPMed_OFP=0.999862;Best_TOPMed_AF=9.6e-05;Best_TOPMed_Het=27;Best_TOPMed_HomAlt=0;Best_TOPMed_PopMax_AF=0.00293606;Best_TOPMed_Mismatch_ID=INV_8:128096507-128103016;Best_TOPMed_Mismatch_OFP=0.172482;Best_TOPMed_Mismatch_SVTYPE=INV;Best_TOPMed_Mismatch_AF=4e-06;Best_TOPMed_Mismatch_Het=1;Best_TOPMed_Mismatch_HomAlt=0;TOPMed_SV_Cov=1;ThousG_Count=1;Best_ThousG_ID=HGSV_158111;Best_ThousG_OFP=1;Best_ThousG_AF=0.000156;Best_ThousG_Het=1;Best_ThousG_HomAlt=0;Best_ThousG_PopMax_AF=0.000832;ThousG_SV_Cov=1;gnomAD_Count=1;Best_gnomAD_ID=gnomAD-SV_v3_DEL_chr8_2492fa30;Best_gnomAD_OFP=1;Best_gnomAD_AF=5.6e-05;Best_gnomAD_Het=7;Best_gnomAD_HomAlt=0;Best_gnomAD_PopMax_AF=0.001589;Best_gnomAD_Mismatch_ID=gnomAD-SV_v3_DUP_chr8_e50742fa;Best_gnomAD_Mismatch_OFP=0.000526648;Best_gnomAD_Mismatch_SVTYPE=DUP;Best_gnomAD_Mismatch_AF=8e-06;Best_gnomAD_Mismatch_Het=1;Best_gnomAD_Mismatch_HomAlt=0;gnomAD_SV_Cov=1	GT:PSV:LN:DR:ST:QV:TY:ID:RAL:AAL:CO	0/1:NA:7253:16,12:+:1320:DEL:DEL00027180:NA:NA:chr8_128092107-chr8_128099360	0/1:NA:7252:0,0:+:674:DEL:MantaDEL_166772_1_2_0_0_0:NA:NA:chr8_128092107-chr8_128099359	0/1:NA:7252:0,0:+:548:DEL:5356:NA:NA:chr8_128092107-chr8_128099359	split	q24.21	PVT1				NR_003367	4	1.28E+08	1.28E+08	7254	0	0	no	9	intron6-intron7	UTR	9	3'	1.28E+08	1.28E+08																													dbVar	chr8:128091460-128099839	0.01																																										5820									0						full=1	0/1
20_20707383_20707707_DEL_1	20	20707383	20707707	-323	DEL	ILS_AP001,ILS_AP001_1,ILS_AP001_2	MantaDEL:97912:2:2:1:0:0	TCAGGAAAAGGGAAGGAAGAAAAAAAAGGAAAGAAGGAAGGAGAGAAAAGAACAAAATTTCACAATCCAGGAAATGGTAAGCTAAATTATGGGATATGGTCTCAACAAATGTTTGTACTTAAAAATGAGAGGAACAAAACATCAAAAATGCTAATGATAAAATAAAGACAACAGTTACAACTGTAAGTCAATTTGTTCCTACCTAGAGCAACATGAGCAACATATGCACAAAAGGAACTATACGACAATGCTATGTTTTGCTCTGTTTGTATATACAGCATTTGTGAGAGACGGTTCTCTACTCTCTGGTCCAAACCCAGGTCA	T	420	PASS	SUPP=3;SUPP_VEC=111;SVLEN=-323;SVTYPE=DEL;SVMETHOD=SURVIVOR1.0.7;CHR2=20;END=20707706;CIPOS=0,1;CIEND=0,1;STRANDS=+;Max_AF=0.001093;Max_Het=93;Max_HomAlt=0;Max_PopMax_AF=0.005067;CCDG_Count=0;Best_CCDG_Mismatch_ID=293405_1;Best_CCDG_Mismatch_OFP=0.00308642;Best_CCDG_Mismatch_SVTYPE=BND;Best_CCDG_Mismatch_AF=0.000718;Best_CCDG_Mismatch_Het=0;Best_CCDG_Mismatch_HomAlt=0;SV_Cov=1;CCDG_SV_Cov=0;TOPMed_Count=0;Best_TOPMed_ID=DEL_20:20573614-20734848;Best_TOPMed_OFP=0.00200948;Best_TOPMed_AF=4e-06;Best_TOPMed_Het=1;Best_TOPMed_HomAlt=0;Best_TOPMed_PopMax_AF=0.000108743;Best_TOPMed_Mismatch_ID=DUP_20:20642402-20730900;Best_TOPMed_Mismatch_OFP=0.00366102;Best_TOPMed_Mismatch_SVTYPE=DUP;Best_TOPMed_Mismatch_AF=4e-06;Best_TOPMed_Mismatch_Het=1;Best_TOPMed_Mismatch_HomAlt=0;TOPMed_SV_Cov=1;ThousG_Count=1;Best_ThousG_ID=HGSV_89805;Best_ThousG_OFP=0.996914;Best_ThousG_AF=0.001093;Best_ThousG_Het=7;Best_ThousG_HomAlt=0;Best_ThousG_PopMax_AF=0;ThousG_SV_Cov=0.996914;gnomAD_Count=1;Best_gnomAD_ID=gnomAD-SV_v3_DEL_chr20_ed4ccf3e;Best_gnomAD_OFP=0.996914;Best_gnomAD_AF=0.000742;Best_gnomAD_Het=93;Best_gnomAD_HomAlt=0;Best_gnomAD_PopMax_AF=0.005067;Best_gnomAD_Mismatch_ID=gnomAD-SV_v3_INV_chr20_01a6ba41;Best_gnomAD_Mismatch_OFP=9.80992e-06;Best_gnomAD_Mismatch_SVTYPE=INV;Best_gnomAD_Mismatch_AF=8e-06;Best_gnomAD_Mismatch_Het=1;Best_gnomAD_Mismatch_HomAlt=0;gnomAD_SV_Cov=1	GT:PSV:LN:DR:ST:QV:TY:ID:RAL:AAL:CO	0/1:NA:324:0,0:+:420:DEL:DEL00058410:NA:NA:chr20_20707383-chr20_20707707	0/1:NA:323:0,0:+:335:DEL:MantaDEL_97912_2_2_1_0_0:TCAGGAAAAGGGAAGGAAGAAAAAAAAGGAAAGAAGGAAGGAGAGAAAAGAACAAAATTTCACAATCCAGGAAATGGTAAGCTAAATTATGGGATATGGTCTCAACAAATGTTTGTACTTAAAAATGAGAGGAACAAAACATCAAAAATGCTAATGATAAAATAAAGACAACAGTTACAACTGTAAGTCAATTTGTTCCTACCTAGAGCAACATGAGCAACATATGCACAAAAGGAACTATACGACAATGCTATGTTTTGCTCTGTTTGTATATACAGCATTTGTGAGAGACGGTTCTCTACTCTCTGGTCCAAACCCAGGTCA:T:chr20_20707383-chr20_20707706	0/1:NA:322:0,0:+:143:DEL:10805:NA:NA:chr20_20707384-chr20_20707706	split	p11.23	RALGAPA2				NM_020343	4	20389529	20712644	325	0	0	no	40	intron1-intron1	CDS	4667	5'	20707382	20707707																													dbVar	chr20:20707354-20710228	0.01																																22.17	0.73525	1.134025	1.134389	-2.26508	-1.04803					57186						3	3.03E-17	8.49E-06	0						full=1	0/1
13_113999035_113999116_DEL_1	13	1.14E+08	1.14E+08	-80	DEL	ILS_AP001,ILS_AP001_1,ILS_AP001_2	MantaDEL:14350:0:1:0:0:0	ACAAGTGTGACTGGGGAACGAGGCGACATGTGGGTCCGGGTCAAGCGTGACCGGGGAACGAGGCCACACGTGGGCCCGGGT	A	550	PASS	SUPP=3;SUPP_VEC=111;SVLEN=-80;SVTYPE=DEL;SVMETHOD=SURVIVOR1.0.7;CHR2=13;END=113999115;CIPOS=0,23;CIEND=0,1;STRANDS=+;Max_AF=0;Max_Het=0;Max_HomAlt=0;Max_PopMax_AF=0;CCDG_Count=0;Best_CCDG_Mismatch_ID=201266_1;Best_CCDG_Mismatch_OFP=0.0123457;Best_CCDG_Mismatch_SVTYPE=BND;Best_CCDG_Mismatch_AF=0.5229;Best_CCDG_Mismatch_Het=0;Best_CCDG_Mismatch_HomAlt=0;SV_Cov=1;CCDG_SV_Cov=0;TOPMed_Count=0;Best_TOPMed_ID=DEL_13:113999059-113999115;Best_TOPMed_OFP=0.716049;Best_TOPMed_AF=0.541849;Best_TOPMed_Het=62552;Best_TOPMed_HomAlt=37716;Best_TOPMed_PopMax_AF=0.663503;Best_TOPMed_Mismatch_ID=DUP_13:113973753-114016426;Best_TOPMed_Mismatch_OFP=0.00189807;Best_TOPMed_Mismatch_SVTYPE=DUP;Best_TOPMed_Mismatch_AF=7e-06;Best_TOPMed_Mismatch_Het=2;Best_TOPMed_Mismatch_HomAlt=0;TOPMed_SV_Cov=0.716049;ThousG_Count=0;Best_ThousG_ID=HGSV_43308;Best_ThousG_OFP=0.716049;Best_ThousG_AF=0.590694;Best_ThousG_Het=1315;Best_ThousG_HomAlt=1215;Best_ThousG_PopMax_AF=0.675538;Best_ThousG_Mismatch_ID=HGSV_43286;Best_ThousG_Mismatch_OFP=0.000320288;Best_ThousG_Mismatch_SVTYPE=DUP;Best_ThousG_Mismatch_AF=0.000781;Best_ThousG_Mismatch_Het=5;Best_ThousG_Mismatch_HomAlt=0;ThousG_SV_Cov=1;gnomAD_Count=0;Best_gnomAD_ID=gnomAD-SV_v3_DEL_chr13_0d8739b9;Best_gnomAD_OFP=0.716049;Best_gnomAD_AF=0.439806;Best_gnomAD_Het=38962;Best_gnomAD_HomAlt=8247;Best_gnomAD_PopMax_AF=0.59375;Best_gnomAD_Mismatch_ID=gnomAD-SV_v3_DUP_chr13_c178adad;Best_gnomAD_Mismatch_OFP=0.00189798;Best_gnomAD_Mismatch_SVTYPE=DUP;Best_gnomAD_Mismatch_AF=8e-06;Best_gnomAD_Mismatch_Het=1;Best_gnomAD_Mismatch_HomAlt=0;gnomAD_SV_Cov=1	GT:PSV:LN:DR:ST:QV:TY:ID:RAL:AAL:CO	0/1:NA:81:0,0:+:300:DEL:DEL00041495:NA:NA:chr13_113999035-chr13_113999116	0/1:NA:80:0,0:+:550:DEL:MantaDEL_14350_0_1_0_0_0:ACAAGTGTGACTGGGGAACGAGGCGACATGTGGGTCCGGGTCAAGCGTGACCGGGGAACGAGGCCACACGTGGGCCCGGGT:A:chr13_113999035-chr13_113999115	0/1:NA:57:0,0:+:251:DEL:7892:NA:NA:chr13_113999058-chr13_113999115	split	q34	RASA3				XM_047430154	1	1.14E+08	1.14E+08	82	0	0	no	23	intron19-intron19	CDS	468	5'	1.14E+08	1.14E+08																													dgv587e212	13:113998170-114004956	0.01																																55.88	-2.62354	-2.38034	-2.41351	-0.46153	1.706913					22821						3	1.91E-10	4.34E-04	0						full=3	0/1
12_15187735_15190338_DEL_1	12	15187735	15190338	-2602	DEL	ILS_AP001,ILS_AP001_1,ILS_AP001_2	MantaDEL:15943:0:1:0:0:0	T		1400	PASS	SUPP=3;SUPP_VEC=111;SVLEN=-2602;SVTYPE=DEL;SVMETHOD=SURVIVOR1.0.7;CHR2=12;END=15190337;CIPOS=0,1;CIEND=0,1;STRANDS=+;Max_AF=0.004527;Max_Het=102;Max_HomAlt=4;Max_PopMax_AF=0.024739;CCDG_Count=0;Best_CCDG_Mismatch_ID=176473_2;Best_CCDG_Mismatch_OFP=0.000384172;Best_CCDG_Mismatch_SVTYPE=BND;Best_CCDG_Mismatch_AF=0.001368;Best_CCDG_Mismatch_Het=0;Best_CCDG_Mismatch_HomAlt=0;SV_Cov=1;CCDG_SV_Cov=0;TOPMed_Count=1;Best_TOPMed_ID=DEL_12:15187736-15190337;Best_TOPMed_OFP=1;Best_TOPMed_AF=7e-06;Best_TOPMed_Het=2;Best_TOPMed_HomAlt=0;Best_TOPMed_PopMax_AF=0.000224316;Best_TOPMed_Mismatch_ID=DUP_12:15170215-15277457;Best_TOPMed_Mismatch_OFP=0.0242718;Best_TOPMed_Mismatch_SVTYPE=DUP;Best_TOPMed_Mismatch_AF=4e-06;Best_TOPMed_Mismatch_Het=1;Best_TOPMed_Mismatch_HomAlt=0;TOPMed_SV_Cov=1;ThousG_Count=1;Best_ThousG_ID=HGSV_30358;Best_ThousG_OFP=0.999616;Best_ThousG_AF=0.004527;Best_ThousG_Het=29;Best_ThousG_HomAlt=0;Best_ThousG_PopMax_AF=0.024126;Best_ThousG_Mismatch_ID=HGSV_30359;Best_ThousG_Mismatch_OFP=0.569291;Best_ThousG_Mismatch_SVTYPE=DUP;Best_ThousG_Mismatch_AF=0.003443;Best_ThousG_Mismatch_Het=20;Best_ThousG_Mismatch_HomAlt=1;ThousG_SV_Cov=1;gnomAD_Count=1;Best_gnomAD_ID=gnomAD-SV_v3_DEL_chr12_0a8412c2;Best_gnomAD_OFP=1;Best_gnomAD_AF=0.000872;Best_gnomAD_Het=102;Best_gnomAD_HomAlt=4;Best_gnomAD_PopMax_AF=0.024739;gnomAD_Mismatches=gnomAD-SV_v3_DUP_chr12_3354a420;gnomAD_Mismatches_Count=1;gnomAD_Mismatch_SVTYPEs=DUP;Best_gnomAD_Mismatch_ID=gnomAD-SV_v3_DUP_chr12_3354a420;Best_gnomAD_Mismatch_OFP=0.894194;Best_gnomAD_Mismatch_SVTYPE=DUP;Best_gnomAD_Mismatch_AF=0.000151;Best_gnomAD_Mismatch_Het=19;Best_gnomAD_Mismatch_HomAlt=0;gnomAD_SV_Cov=1	GT:PSV:LN:DR:ST:QV:TY:ID:RAL:AAL:CO	0/1:NA:2603:26,14:+:1400:DEL:DEL00037138:NA:NA:chr12_15187735-chr12_15190338	0/1:NA:2602:0,0:+:891:DEL:MantaDEL_15943_0_1_0_0_0:NA:NA:chr12_15187735-chr12_15190337	0/1:NA:2601:0,0:+:578:DEL:7014:NA:NA:chr12_15187736-chr12_15190337	split	p12.3	RERG				XM_047429797	1	15107782	15217600	2604	0	0	no	5	intron1-intron1	5'UTR	27090	5'	15187734	15190338																																			IMH	12:9566784-31121901;12:9566998-31121904;12:9567304-31122039;12:9568909-31118782	0.2224																										34.36	0.626375	0.917174	0.957334	0.950319	0.512215					85004						7	1.20E-05	1.17E-03	0						full=3	0/1
12_15188606_15190644_DUP_1	12	15188606	15190644	2036	DUP	ILS_AP001,ILS_AP001_1,ILS_AP001_2	MantaDUP:TANDEM:15943:1:2:0:0:0	T		1260	PASS	SUPP=3;SUPP_VEC=111;SVLEN=2036;SVTYPE=DUP;SVMETHOD=SURVIVOR1.0.7;CHR2=12;END=15190642;CIPOS=0,1;CIEND=0,2;STRANDS=-;Max_AF=0.003443;Max_Het=20;Max_HomAlt=1;Max_PopMax_AF=0.01855;CCDG_Count=0;Best_CCDG_Mismatch_ID=470186_1;Best_CCDG_Mismatch_OFP=0.000490918;Best_CCDG_Mismatch_SVTYPE=BND;Best_CCDG_Mismatch_AF=0.001368;Best_CCDG_Mismatch_Het=0;Best_CCDG_Mismatch_HomAlt=0;SV_Cov=1;CCDG_SV_Cov=0;TOPMed_Count=0;Best_TOPMed_ID=DUP_12:15170215-15277457;Best_TOPMed_OFP=0.0189941;Best_TOPMed_AF=4e-06;Best_TOPMed_Het=1;Best_TOPMed_HomAlt=0;Best_TOPMed_PopMax_AF=0.000107021;Best_TOPMed_Mismatch_ID=DEL_12:15187736-15190337;Best_TOPMed_Mismatch_OFP=0.565758;Best_TOPMed_Mismatch_SVTYPE=DEL;Best_TOPMed_Mismatch_AF=7e-06;Best_TOPMed_Mismatch_Het=2;Best_TOPMed_Mismatch_HomAlt=0;TOPMed_SV_Cov=1;ThousG_Count=1;Best_ThousG_ID=HGSV_30359;Best_ThousG_OFP=0.966777;Best_ThousG_AF=0.003443;Best_ThousG_Het=20;Best_ThousG_HomAlt=1;Best_ThousG_PopMax_AF=0.01855;Best_ThousG_Mismatch_ID=HGSV_30358;Best_ThousG_Mismatch_OFP=0.565975;Best_ThousG_Mismatch_SVTYPE=DEL;Best_ThousG_Mismatch_AF=0.004527;Best_ThousG_Mismatch_Het=29;Best_ThousG_Mismatch_HomAlt=0;ThousG_SV_Cov=1;gnomAD_Count=1;Best_gnomAD_ID=gnomAD-SV_v3_DUP_chr12_12620f4f;Best_gnomAD_OFP=0.849288;Best_gnomAD_AF=8.7e-05;Best_gnomAD_Het=11;Best_gnomAD_HomAlt=0;Best_gnomAD_PopMax_AF=0.002608;Best_gnomAD_Mismatch_ID=gnomAD-SV_v3_DEL_chr12_0a8412c2;Best_gnomAD_Mismatch_OFP=0.565758;Best_gnomAD_Mismatch_SVTYPE=DEL;Best_gnomAD_Mismatch_AF=0.000872;Best_gnomAD_Mismatch_Het=102;Best_gnomAD_Mismatch_HomAlt=4;gnomAD_SV_Cov=1	GT:PSV:LN:DR:ST:QV:TY:ID:RAL:AAL:CO	0/1:NA:2035:30,13:-:1260:DUP:DUP00037139:NA:NA:chr12_15188607-chr12_15190642	0/1:NA:2036:0,0:-:711:DUP:MantaDUP_TANDEM_15943_1_2_0_0_0:NA:NA:chr12_15188606-chr12_15190642	0/1:NA:2038:0,0:-:280:DUP:7015:NA:NA:chr12_15188606-chr12_15190644	split	p12.3	RERG				XM_047429797	1	15107782	15217600	2039	0	0	no	5	intron1-intron1	5'UTR	26784	5'	15188605	15190644																																			IMH	12:9566784-31121901;12:9566998-31121904;12:9567304-31122039;12:9568909-31118782	0.2224																										34.36	0.626375	0.917174	0.957334	0.950319	0.512215					85004						7	1.20E-05	1.17E-03	0						full=3	0/1
17_80301098_80301374_INV_1	17	80301098	80301374	276	INV	ILS_AP001,ILS_AP001_1,ILS_AP001_2	MantaBND:55724:0:0:0:0:0:0	T	T]chr17:80301374]	240	PASS	SUPP=3;SUPP_VEC=111;SVLEN=276;SVTYPE=INV;SVMETHOD=SURVIVOR1.0.7;CHR2=17;END=80301374;CIPOS=0,259;CIEND=-259,0;STRANDS=;Max_AF=0;Max_Het=0;Max_HomAlt=0;Max_PopMax_AF=0;CCDG_Count=0;SV_Cov=1;CCDG_SV_Cov=0;TOPMed_Count=0;Best_TOPMed_ID=INV_17:79606853-81315890;Best_TOPMed_OFP=0.000162079;Best_TOPMed_AF=4e-06;Best_TOPMed_Het=1;Best_TOPMed_HomAlt=0;Best_TOPMed_PopMax_AF=6.25986e-06;Best_TOPMed_Mismatch_ID=DUP_17:80293180-80323606;Best_TOPMed_Mismatch_OFP=0.00910346;Best_TOPMed_Mismatch_SVTYPE=DUP;Best_TOPMed_Mismatch_AF=4e-06;Best_TOPMed_Mismatch_Het=1;Best_TOPMed_Mismatch_HomAlt=0;TOPMed_SV_Cov=1;ThousG_Count=0;ThousG_SV_Cov=0;gnomAD_Count=0;Best_gnomAD_Mismatch_ID=gnomAD-SV_v3_DUP_chr17_7f9a57b0;Best_gnomAD_Mismatch_OFP=0.00266346;Best_gnomAD_Mismatch_SVTYPE=DUP;Best_gnomAD_Mismatch_AF=1.6e-05;Best_gnomAD_Mismatch_Het=2;Best_gnomAD_Mismatch_HomAlt=0;gnomAD_SV_Cov=0	GT:PSV:LN:DR:ST:QV:TY:ID:RAL:AAL:CO	0/1:NA:276:0,0::240:INV:INV00052505:NA:NA:chr17_80301098-chr17_80301374	0/1:NA:276:0,0::23,23:INV,INV:MantaBND_55724_0_0_0_0_0_0:NA:NA:chr17_80301098-chr17_80301374,chr17_80301357-chr17_80301115	0/1:NA:251:0,0::32:INV:9722:NA:NA:chr17_80301098-chr17_80301349	split	q25.3	RNF213				NM_020954	4	80260851	80321463	277	0	0	no	17	intron11-intron11	CDS	2579	5'	80301097	80301374																										dbVar	chr17:80246582-80352532	0.01	dbVar	chr17:80274417-80303391	0.01																																93.8	-2.52003	-2.53125	-2.34602	-0.37966	2.289337	Moyamoya disease 2	AD;AR	Strong	21048783;22377813;22931863;23850618;23994138;25278557	57674	613768	(Moyamoya disease 2, susceptibility to), 607151 (3) AR,AD	AR,AD		yes	3	4.91E-55	1.68E-29	0.2179			Holoprosencephaly;MiP motor neuron decreased process quality axon extension involved in axon guidance, abnormal			full=NA	0/1
4_183652348_183652410_DEL_1	4	1.84E+08	1.84E+08	-61	DEL	ILS_AP001,ILS_AP001_1,ILS_AP001_2	MantaDEL:156308:0:0:0:0:0	CCCTCTCCCCTGGCTTTTTTTTTTTTTTTTTGAGACAGGGTCTCACTCCATTGCCCAGGCTG	C	703	PASS	SUPP=3;SUPP_VEC=111;SVLEN=-61;SVTYPE=DEL;SVMETHOD=SURVIVOR1.0.7;CHR2=4;END=183652409;CIPOS=0,0;CIEND=0,1;STRANDS=+;Max_AF=0;Max_Het=0;Max_HomAlt=0;Max_PopMax_AF=0;CCDG_Count=0;Best_CCDG_Mismatch_ID=514836;Best_CCDG_Mismatch_OFP=0.000217671;Best_CCDG_Mismatch_SVTYPE=DUP;Best_CCDG_Mismatch_AF=6.839e-05;Best_CCDG_Mismatch_Het=0;Best_CCDG_Mismatch_HomAlt=0;SV_Cov=1;CCDG_SV_Cov=0;TOPMed_Count=0;Best_TOPMed_ID=DEL_4:183652247-183656966;Best_TOPMed_OFP=0.0131328;Best_TOPMed_AF=4e-06;Best_TOPMed_Het=1;Best_TOPMed_HomAlt=0;Best_TOPMed_PopMax_AF=6.26676e-06;Best_TOPMed_Mismatch_ID=DUP_4:183509740-183794572;Best_TOPMed_Mismatch_OFP=0.000217671;Best_TOPMed_Mismatch_SVTYPE=DUP;Best_TOPMed_Mismatch_AF=7e-06;Best_TOPMed_Mismatch_Het=2;Best_TOPMed_Mismatch_HomAlt=0;TOPMed_SV_Cov=1;ThousG_Count=0;Best_ThousG_Mismatch_ID=HGSV_119000;Best_ThousG_Mismatch_OFP=0.000257214;Best_ThousG_Mismatch_SVTYPE=INV;Best_ThousG_Mismatch_AF=0.000156;Best_ThousG_Mismatch_Het=1;Best_ThousG_Mismatch_HomAlt=0;ThousG_SV_Cov=0;gnomAD_Count=0;Best_gnomAD_ID=gnomAD-SV_v3_DEL_chr4_acf5a577;Best_gnomAD_OFP=0.0709382;Best_gnomAD_AF=0.000103;Best_gnomAD_Het=11;Best_gnomAD_HomAlt=0;Best_gnomAD_PopMax_AF=0.000381;Best_gnomAD_Mismatch_ID=gnomAD-SV_v3_INV_chr4_61f32a06;Best_gnomAD_Mismatch_OFP=0.000257027;Best_gnomAD_Mismatch_SVTYPE=INV;Best_gnomAD_Mismatch_AF=8e-06;Best_gnomAD_Mismatch_Het=1;Best_gnomAD_Mismatch_HomAlt=0;gnomAD_SV_Cov=1	GT:PSV:LN:DR:ST:QV:TY:ID:RAL:AAL:CO	0/1:NA:62:0,0:+:703:DEL:DEL00015760:NA:NA:chr4_183652348-chr4_183652410	0/1:NA:61:0,0:+:370:DEL:MantaDEL_156308_0_0_0_0_0:CCCTCTCCCCTGGCTTTTTTTTTTTTTTTTTGAGACAGGGTCTCACTCCATTGCCCAGGCTG:C:chr4_183652348-chr4_183652409	0/1:NA:61:0,0:+:320:DEL:3022:NA:NA:chr4_183652348-chr4_183652409	split	q35.1	RWDD4				NM_001307922	2	1.84E+08	1.84E+08	63	0	0	no	8	intron2-intron2	5'UTR	1024	3'	1.84E+08	1.84E+08																													gnomAD-SV_v3_DEL_chr4_aa7116c9	chr4:73564711-189048534	0.2162				IMH	4:179407330-186243582	0.2421																										39.45	-0.36445	-0.66679	-0.5522	0.428558	0.565034					201965						4	1.74E-02	4.83E-01	0						full=1	0/1
9_133718380_133718862_DEL_1	9	1.34E+08	1.34E+08	-481	DEL	ILS_AP001,ILS_AP001_1,ILS_AP001_2	MantaDEL:213941:0:1:0:0:0	GTTTATTGTATGTCTCTCCACCAAAATATAGGCACCCAGAGTCAGGGACTTTTATCTTAGTTTCCCTCTTTCTCCAGAAATTAAAGCAGTTTTTAGCCCAAAGTAGCCACTCAGTCGTTCCATGTGTGGACTAAATGCATATACACATAGAACAGGGCTGCAGGGCTGCCCTAACCCCAGAAGCTGCCCGGGAAACAGCCCAGGCCAGGGGAAATGCCGCTGCAGCAGCTGGTTGGGAGGGCAGTGTCATTTGCTGAAGGACGTAGGACTTGTGTGCTCTCATGCCCTTCTGAGGTCATCACTCCACACTGCGCAGACCTTCTCTGTGGATGTTTTGAGGCAAAGCCCTCCAGCTGCCCTGGGTCTGTATTTGACTGCCTGCCAGGACCGTCAGGGTAAGAGCAAGATGGCTTTGAGCTTGGTGGGGTCAGGGGACCGGCCACTTCTCAGGTGTGCCTCTGAGGAGCTTCAGGAGGATGGAC	G	785	PASS	SUPP=3;SUPP_VEC=111;SVLEN=-481;SVTYPE=DEL;SVMETHOD=SURVIVOR1.0.7;CHR2=9;END=133718861;CIPOS=0,2;CIEND=0,1;STRANDS=+;Max_AF=0.005308;Max_Het=372;Max_HomAlt=5;Max_PopMax_AF=0.026496;CCDG_Count=0;Best_CCDG_Mismatch_ID=437615_2;Best_CCDG_Mismatch_OFP=0.00207469;Best_CCDG_Mismatch_SVTYPE=BND;Best_CCDG_Mismatch_AF=0.0007522;Best_CCDG_Mismatch_Het=0;Best_CCDG_Mismatch_HomAlt=0;SV_Cov=1;CCDG_SV_Cov=0;TOPMed_Count=1;Best_TOPMed_ID=DEL_9:133718383-133718861;Best_TOPMed_OFP=0.995851;Best_TOPMed_AF=0.001356;Best_TOPMed_Het=372;Best_TOPMed_HomAlt=1;Best_TOPMed_PopMax_AF=0.0195774;Best_TOPMed_Mismatch_ID=DUP_9:133630772-133737768;Best_TOPMed_Mismatch_OFP=0.00450476;Best_TOPMed_Mismatch_SVTYPE=DUP;Best_TOPMed_Mismatch_AF=4e-06;Best_TOPMed_Mismatch_Het=1;Best_TOPMed_Mismatch_HomAlt=0;TOPMed_SV_Cov=1;ThousG_Count=1;Best_ThousG_ID=HGSV_165988;Best_ThousG_OFP=0.995851;Best_ThousG_AF=0.005308;Best_ThousG_Het=24;Best_ThousG_HomAlt=5;Best_ThousG_PopMax_AF=0.026496;ThousG_SV_Cov=0.995851;gnomAD_Count=0;Best_gnomAD_ID=gnomAD-SV_v3_DEL_chr9_09eec3c6;Best_gnomAD_OFP=0.790164;Best_gnomAD_AF=0.002301;Best_gnomAD_Het=282;Best_gnomAD_HomAlt=4;Best_gnomAD_PopMax_AF=0.025407;Best_gnomAD_Mismatch_ID=gnomAD-SV_v3_DUP_chr9_771259bb;Best_gnomAD_Mismatch_OFP=0.00388153;Best_gnomAD_Mismatch_SVTYPE=DUP;Best_gnomAD_Mismatch_AF=8e-06;Best_gnomAD_Mismatch_Het=1;Best_gnomAD_Mismatch_HomAlt=0;gnomAD_SV_Cov=1	GT:PSV:LN:DR:ST:QV:TY:ID:RAL:AAL:CO	0/1:NA:482:0,0:+:366:DEL:DEL00029807:NA:NA:chr9_133718380-chr9_133718862	0/1:NA:481:0,0:+:785:DEL:MantaDEL_213941_0_1_0_0_0:GTTTATTGTATGTCTCTCCACCAAAATATAGGCACCCAGAGTCAGGGACTTTTATCTTAGTTTCCCTCTTTCTCCAGAAATTAAAGCAGTTTTTAGCCCAAAGTAGCCACTCAGTCGTTCCATGTGTGGACTAAATGCATATACACATAGAACAGGGCTGCAGGGCTGCCCTAACCCCAGAAGCTGCCCGGGAAACAGCCCAGGCCAGGGGAAATGCCGCTGCAGCAGCTGGTTGGGAGGGCAGTGTCATTTGCTGAAGGACGTAGGACTTGTGTGCTCTCATGCCCTTCTGAGGTCATCACTCCACACTGCGCAGACCTTCTCTGTGGATGTTTTGAGGCAAAGCCCTCCAGCTGCCCTGGGTCTGTATTTGACTGCCTGCCAGGACCGTCAGGGTAAGAGCAAGATGGCTTTGAGCTTGGTGGGGTCAGGGGACCGGCCACTTCTCAGGTGTGCCTCTGAGGAGCTTCAGGAGGATGGAC:G:chr9_133718380-chr9_133718861	0/1:NA:479:0,0:+:506:DEL:5829:NA:NA:chr9_133718382-chr9_133718861	split	q34.2	SARDH				XM_047422896	1	1.34E+08	1.34E+08	483	0	0	no	21	intron7-intron7	CDS	75	5'	1.34E+08	1.34E+08																													dbVar	chr9:133707784-133753594	0.01				IMH	9:65437055-134286391	0.2414																										61.17	-2.33998	-0.1572	-1.03945	0.591302	0.419139	sarcosinemia	AR	Limited;Supportive	22825317;22825317[PMID];27001897;30612563	1757	604455	[Sarcosinemia], 268900 (3) AR	AR			4	5.27E-16	8.20E-10	0.3044		Holoprosencephaly;abnormal hippocampus morphology				full=1	0/1
7_84422766_84423628_DEL_1	7	84422766	84423628	-861	DEL	ILS_AP001,ILS_AP001_1,ILS_AP001_2	MantaDEL:111727:0:1:0:0:0	TTATGTACCCAGTAGTCATTCAGGAGCAGGTTGTTCAGTTTCCATGAATTGGTATCAGTAAAAAGTAGGAAGTTACAAATTTAGAATGTCAATTGTAATCCCTATAGGGACCAAAGAAATGTATCTCTAAAGTACCAGCATGCAAACTCAGTTTAATTCCCCAGTCTATCCTTTTAACCATGTTATGCTCCTTCTTACTGTAAAAGGAGATTTGAATCATCCACACATTCATTGAGCAAATATGTATTTTCACCATCCAGTGATTAATATTGTAATAGGTTCTGGCATTATAATGCTGAATAAAATATTAAACATAGCTTCCAATAGATAATAGTTTTACCATTGTACAATATTTATTGTAACAATAGAAAAATGTACACAAATCAAAACATTTTCTATCAAATAAGTACAATGTAAAAGAAGACATGTTTGTAGTTCTCATACCATTAGAATACTATGAAAAGATTTGCAATATTAGACTCTAATATGAATTTGTTACTAGGTTAATGTTTGTAAGTTTCAAGTGGTCTCTCCACCTCTTTTACCTCAATTCCTGTCCTTCTTCCACTGCAGTCCCACTGATATCCTTGCCATTTCTTAAAAATACTGGGCATATTTCTGCCTTGAGACCTTTGATTTGCTATTCTTTCAGCAAAAACTCAGTCACTGCACATAACCTGGAGGGTCCTCCCTCACGTATTTCACTTAACCAAAGAGGTCTTCTGTGGTCATCCTGAACAAATGAATTGCATTCCTTCCCAGACACTTCCTATTGTAATTTATTTCCTATCTCTGTAATTATTTTTTTGTCGAAATAGATAGTTATATCATCTATATCTATATTTAAACCTATATCTATCTA	T	827	PASS	SUPP=3;SUPP_VEC=111;SVLEN=-861;SVTYPE=DEL;SVMETHOD=SURVIVOR1.0.7;CHR2=7;END=84423627;CIPOS=0,5;CIEND=0,1;STRANDS=+;Max_AF=0;Max_Het=0;Max_HomAlt=0;Max_PopMax_AF=0;CCDG_Count=0;Best_CCDG_ID=399968;Best_CCDG_OFP=0.00665745;Best_CCDG_AF=3.419e-05;Best_CCDG_Het=0;Best_CCDG_HomAlt=0;Best_CCDG_PopMax_AF=0;Best_CCDG_Mismatch_ID=528990;Best_CCDG_Mismatch_OFP=0.00901315;Best_CCDG_Mismatch_SVTYPE=DUP;Best_CCDG_Mismatch_AF=3.419e-05;Best_CCDG_Mismatch_Het=0;Best_CCDG_Mismatch_HomAlt=0;SV_Cov=1;CCDG_SV_Cov=1;TOPMed_Count=0;Best_TOPMed_ID=DEL_7:84422066-84478256;Best_TOPMed_OFP=0.0153403;Best_TOPMed_AF=3.6e-05;Best_TOPMed_Het=10;Best_TOPMed_HomAlt=0;Best_TOPMed_PopMax_AF=0.000120005;Best_TOPMed_Mismatch_ID=DUP_7:84416081-84427815;Best_TOPMed_Mismatch_OFP=0.0734492;Best_TOPMed_Mismatch_SVTYPE=DUP;Best_TOPMed_Mismatch_AF=4e-06;Best_TOPMed_Mismatch_Het=1;Best_TOPMed_Mismatch_HomAlt=0;TOPMed_SV_Cov=1;ThousG_Count=0;Best_ThousG_ID=HGSV_145954;Best_ThousG_OFP=0.000426217;Best_ThousG_AF=0.000156;Best_ThousG_Het=1;Best_ThousG_HomAlt=0;Best_ThousG_PopMax_AF=0.000832;Best_ThousG_Mismatch_ID=HGSV_140355;Best_ThousG_Mismatch_OFP=5.5682e-06;Best_ThousG_Mismatch_SVTYPE=CPX;Best_ThousG_Mismatch_AF=0.000156;Best_ThousG_Mismatch_Het=1;Best_ThousG_Mismatch_HomAlt=0;ThousG_SV_Cov=1;gnomAD_Count=0;Best_gnomAD_ID=gnomAD-SV_v3_DEL_chr7_32a2abc1;Best_gnomAD_OFP=0.138541;Best_gnomAD_AF=7.2e-05;Best_gnomAD_Het=9;Best_gnomAD_HomAlt=0;Best_gnomAD_PopMax_AF=0.000404;Best_gnomAD_Mismatch_ID=gnomAD-SV_v3_DUP_chr7_6c78517e;Best_gnomAD_Mismatch_OFP=0.138541;Best_gnomAD_Mismatch_SVTYPE=DUP;Best_gnomAD_Mismatch_AF=3.3e-05;Best_gnomAD_Mismatch_Het=4;Best_gnomAD_Mismatch_HomAlt=0;gnomAD_SV_Cov=1	GT:PSV:LN:DR:ST:QV:TY:ID:RAL:AAL:CO	0/1:NA:862:12,10:+:827:DEL:DEL00023602:NA:NA:chr7_84422766-chr7_84423628	0/1:NA:861:0,0:+:769:DEL:MantaDEL_111727_0_1_0_0_0:TTATGTACCCAGTAGTCATTCAGGAGCAGGTTGTTCAGTTTCCATGAATTGGTATCAGTAAAAAGTAGGAAGTTACAAATTTAGAATGTCAATTGTAATCCCTATAGGGACCAAAGAAATGTATCTCTAAAGTACCAGCATGCAAACTCAGTTTAATTCCCCAGTCTATCCTTTTAACCATGTTATGCTCCTTCTTACTGTAAAAGGAGATTTGAATCATCCACACATTCATTGAGCAAATATGTATTTTCACCATCCAGTGATTAATATTGTAATAGGTTCTGGCATTATAATGCTGAATAAAATATTAAACATAGCTTCCAATAGATAATAGTTTTACCATTGTACAATATTTATTGTAACAATAGAAAAATGTACACAAATCAAAACATTTTCTATCAAATAAGTACAATGTAAAAGAAGACATGTTTGTAGTTCTCATACCATTAGAATACTATGAAAAGATTTGCAATATTAGACTCTAATATGAATTTGTTACTAGGTTAATGTTTGTAAGTTTCAAGTGGTCTCTCCACCTCTTTTACCTCAATTCCTGTCCTTCTTCCACTGCAGTCCCACTGATATCCTTGCCATTTCTTAAAAATACTGGGCATATTTCTGCCTTGAGACCTTTGATTTGCTATTCTTTCAGCAAAAACTCAGTCACTGCACATAACCTGGAGGGTCCTCCCTCACGTATTTCACTTAACCAAAGAGGTCTTCTGTGGTCATCCTGAACAAATGAATTGCATTCCTTCCCAGACACTTCCTATTGTAATTTATTTCCTATCTCTGTAATTATTTTTTTGTCGAAATAGATAGTTATATCATCTATATCTATATTTAAACCTATATCTATCTA:T:chr7_84422766-chr7_84423627	0/1:NA:856:0,0:+:566:DEL:4655:NA:NA:chr7_84422771-chr7_84423627	split	q21.11	SEMA3A				XM_005250110	4	83955776	84492725	863	0	0	no	20	intron1-intron1	5'UTR	50865	3'	84422765	84423628																													dbVar;gnomAD-SV_v3_DEL_chr7_4d7b9722	chr7:83903053-107770163;chr7:84301051-84453488;chr7:84313235-84708494;chr7:84367300-84480840;chr7:84422065-84478253	0.0172				IMH	7:39507051-100351151;7:45847441-101265654;7:45847453-101265940;7:64004376-105619581;7:67304014-102378889;7:67304239-102379805;7:68170339-98454729;7:77095162-102776972;7:77167460-102703083;7:77537156-100694434;7:80929283-101607772	0.4994																								1	0	1.46	0.686272	0.083918	0.357968	0.229205	1.44116	Brugada syndrome;Kallmann syndrome;hypogonadotropic hypogonadism 16 with or without anosmia	AD	Strong;Supportive	22416012;22416012[PMID];22927827;24124006;25077900;28075028;28295047;29419413;29432577;30098700;30821013[PMID]	10371	603961	(Hypogonadotropic hypogonadism 16 with or without anosmia), 614897 (3) AD	AD		yes	1	9.78E-01	9.88E-01	0.599	Cleft lip;Cleft palate;Kallmann syndrome;Micrognathia;Tooth agenesis	Holoprosencephaly;decreased brain size	Holoprosencephaly;axon guidance disrupted, abnormal			full=3	0/1
2_1185338_1185428_DEL_1	2	1185338	1185428	-89	DEL	ILS_AP001,ILS_AP001_1,ILS_AP001_2	MantaDEL:170917:0:0:0:0:0	GTCTGGAGGATGGTAGCCTTCTTCTCACAGTTCCAGTAGTCAGCATCCCAGTAGAGACTCTGTGTGGGAGCTCCAACCGCACATTTCCCC	G	540	PASS	SUPP=3;SUPP_VEC=111;SVLEN=-89;SVTYPE=DEL;SVMETHOD=SURVIVOR1.0.7;CHR2=2;END=1185427;CIPOS=0,4;CIEND=0,1;STRANDS=+;Max_AF=0;Max_Het=0;Max_HomAlt=0;Max_PopMax_AF=0;CCDG_Count=0;Best_CCDG_Mismatch_ID=493883;Best_CCDG_Mismatch_OFP=0.000296683;Best_CCDG_Mismatch_SVTYPE=DUP;Best_CCDG_Mismatch_AF=3.419e-05;Best_CCDG_Mismatch_Het=0;Best_CCDG_Mismatch_HomAlt=0;SV_Cov=1;CCDG_SV_Cov=0;TOPMed_Count=0;Best_TOPMed_ID=DEL_2:1024951-1193643;Best_TOPMed_OFP=0.00053351;Best_TOPMed_AF=4e-06;Best_TOPMed_Het=1;Best_TOPMed_HomAlt=0;Best_TOPMed_PopMax_AF=1.19835e-05;Best_TOPMed_Mismatch_ID=DUP_2:1172982-1306338;Best_TOPMed_Mismatch_OFP=0.000674875;Best_TOPMed_Mismatch_SVTYPE=DUP;Best_TOPMed_Mismatch_AF=4e-06;Best_TOPMed_Mismatch_Het=1;Best_TOPMed_Mismatch_HomAlt=0;TOPMed_SV_Cov=1;ThousG_Count=0;Best_ThousG_Mismatch_ID=HGSV_74862;Best_ThousG_Mismatch_OFP=7.91844e-06;Best_ThousG_Mismatch_SVTYPE=INV;Best_ThousG_Mismatch_AF=0.000312;Best_ThousG_Mismatch_Het=2;Best_ThousG_Mismatch_HomAlt=0;ThousG_SV_Cov=0;gnomAD_Count=0;Best_gnomAD_ID=gnomAD-SV_v3_DEL_chr2_6ba1a7d9;Best_gnomAD_OFP=0.000673754;Best_gnomAD_AF=6.3e-05;Best_gnomAD_Het=8;Best_gnomAD_HomAlt=0;Best_gnomAD_PopMax_AF=0.000102;Best_gnomAD_Mismatch_ID=gnomAD-SV_v3_DUP_chr2_978e7d7f;Best_gnomAD_Mismatch_OFP=0.00200071;Best_gnomAD_Mismatch_SVTYPE=DUP;Best_gnomAD_Mismatch_AF=2.4e-05;Best_gnomAD_Mismatch_Het=3;Best_gnomAD_Mismatch_HomAlt=0;gnomAD_SV_Cov=1	GT:PSV:LN:DR:ST:QV:TY:ID:RAL:AAL:CO	0/1:NA:90:0,0:+:540:DEL:DEL00005171:NA:NA:chr2_1185338-chr2_1185428	0/1:NA:89:0,0:+:349:DEL:MantaDEL_170917_0_0_0_0_0:GTCTGGAGGATGGTAGCCTTCTTCTCACAGTTCCAGTAGTCAGCATCCCAGTAGAGACTCTGTGTGGGAGCTCCAACCGCACATTTCCCC:G:chr2_1185338-chr2_1185427	0/1:NA:85:0,0:+:212:DEL:1065:NA:NA:chr2_1185342-chr2_1185427	split	p25.3	SNTG2				XM_047444795	1	950848	1256610	91	0	0	no	13	intron8-intron8	CDS	12154	5'	1185337	1185428																													dbVar	chr2:1089976-1223555	0.01																																72.04				-0.67374	-0.84034					54221						6	1.31E-14	9.18E-07	0						full=1	0/1
3_181354497_181354568_DEL_1	3	1.81E+08	1.81E+08	-70	DEL	ILS_AP001,ILS_AP001_1,ILS_AP001_2	MantaDEL:169293:0:1:0:0:0	ATGAATGCTAGGTAGCATTCAGTACCTAATGAATGCTAGGTAGCATTCAGTACCTAGCATTCGTTAGGTAC	A	300	PASS	SUPP=3;SUPP_VEC=111;SVLEN=-70;SVTYPE=DEL;SVMETHOD=SURVIVOR1.0.7;CHR2=3;END=181354567;CIPOS=0,9;CIEND=0,1;STRANDS=+;Max_AF=8e-06;Max_Het=1;Max_HomAlt=0;Max_PopMax_AF=1.7e-05;CCDG_Count=0;Best_CCDG_Mismatch_ID=328924_1;Best_CCDG_Mismatch_OFP=0.0140845;Best_CCDG_Mismatch_SVTYPE=BND;Best_CCDG_Mismatch_AF=0.05714;Best_CCDG_Mismatch_Het=0;Best_CCDG_Mismatch_HomAlt=0;SV_Cov=1;CCDG_SV_Cov=0;TOPMed_Count=0;Best_TOPMed_Mismatch_ID=DUP_3:181345202-181356000;Best_TOPMed_Mismatch_OFP=0.00657407;Best_TOPMed_Mismatch_SVTYPE=DUP;Best_TOPMed_Mismatch_AF=4e-06;Best_TOPMed_Mismatch_Het=1;Best_TOPMed_Mismatch_HomAlt=0;TOPMed_SV_Cov=0;ThousG_Count=0;Best_ThousG_Mismatch_ID=HGSV_106001;Best_ThousG_Mismatch_OFP=2.01825e-06;Best_ThousG_Mismatch_SVTYPE=DUP;Best_ThousG_Mismatch_AF=0.000156;Best_ThousG_Mismatch_Het=1;Best_ThousG_Mismatch_HomAlt=0;ThousG_SV_Cov=0;gnomAD_Count=1;Best_gnomAD_ID=gnomAD-SV_v3_DEL_chr3_0dce689d;Best_gnomAD_OFP=0.859379;Best_gnomAD_AF=8e-06;Best_gnomAD_Het=1;Best_gnomAD_HomAlt=0;Best_gnomAD_PopMax_AF=1.7e-05;Best_gnomAD_Mismatch_ID=gnomAD-SV_v3_DUP_chr3_0a7a5017;Best_gnomAD_Mismatch_OFP=0.605055;Best_gnomAD_Mismatch_SVTYPE=DUP;Best_gnomAD_Mismatch_AF=0.00175;Best_gnomAD_Mismatch_Het=180;Best_gnomAD_Mismatch_HomAlt=9;gnomAD_SV_Cov=1	GT:PSV:LN:DR:ST:QV:TY:ID:RAL:AAL:CO	0/1:NA:71:0,0:+:300:DEL:DEL00011750:NA:NA:chr3_181354497-chr3_181354568	0/1:NA:70:0,0:+:81:DEL:MantaDEL_169293_0_1_0_0_0:ATGAATGCTAGGTAGCATTCAGTACCTAATGAATGCTAGGTAGCATTCAGTACCTAGCATTCGTTAGGTAC:A:chr3_181354497-chr3_181354567	0/1:NA:61:0,0:+:104:DEL:2423:NA:NA:chr3_181354506-chr3_181354567	split	q26.33	SOX2-OT				NR_075091	1	1.81E+08	1.82E+08	72	0	0	no	8	intron3-intron3	UTR	178913	5'	1.81E+08	1.81E+08																																			IMH	3:72502419-189537676	0.0342																																				347689									0						full=3	0/1
3_8678474_8679351_DEL_1	3	8678474	8679351	-876	DEL	ILS_AP001,ILS_AP001_1,ILS_AP001_2	MantaDEL:157697:0:1:0:0:0	GGACCCCCATTGCAAGGGGGTGAGGCACCCCCCGCGAGGCGGGGACTGAGAGCCAGCCCCTCTTCCCTCCATGGCTCTTAGGACCCCAATCACAGAGGGGGGAACACCCCCCGCGAGGCAGGGACTGAGAGCCAGCCCTTCTTCCCCCCCGGCTTTTGGGACCCCCATCGCAGTGGGGGGAGGCACCCCCCGCGAGGCGGGGATTGAGAGCCAGCCACTCTTCCCCTCCTGGGTCTTAGGACCCCCAACGTGGGGGGGGGAGGCACCACACGCGAGGCGGGGAAAGAGAGCCAGCCCCTCTTCCCTCCCTGCCACTTAGGACCCCATCGCAGGGAGAGGAGGCGGCACTCCCCACGAGGCGGGGACTGAGAACCAATCCCTCTTCCCCCAGCCTGGCTCTTAGGATCCCCATTGCAAGGGAGGGAGGCACCCCCCGCCAGGCGGGGACTGAGAGCCAGCCACTCTTCCCCTCCTGGCTCTTGGGACGCCCATCGCAGGGCGGAGAGTCACCCCCCGCGAGGTGGGGACTGAGAGCCAGCCCCTCTTCCCCCCCTGGCTCTTAGGACACCAAACGCAGGGGAGGAAGCACCCCCCGCGAGGCGGGGACTGAGAGCAAGCACCTCTTTCCCCCCTGGCTCTTAGGACCCAAATTGCGGGGGGGAGGCACCCCCGCGAGGCGGGGACTGAGAGGCAGCCGCTGTTCCCCCACACTGGCTCTTGGGACCCCCATCGCAGGGGGGATGGCACCCTCCGTGAGCGGGGACTGAGAGCCAGCCAATTTTCCCCCTGGCTTTTAGGACCCCCATCGGAGGGGGGGAGCCACCCCCCATGAGGCGGGGACTAAGAGCCAGCCCCTCTTCCCGCCCCTTGCTCTTCC	G	932	PASS	SUPP=3;SUPP_VEC=111;SVLEN=-876;SVTYPE=DEL;SVMETHOD=SURVIVOR1.0.7;CHR2=3;END=8679350;CIPOS=0,9;CIEND=0,1;STRANDS=+;Max_AF=0;Max_Het=0;Max_HomAlt=0;Max_PopMax_AF=0;CCDG_Count=0;Best_CCDG_ID=311418;Best_CCDG_OFP=0.13731;Best_CCDG_AF=3.419e-05;Best_CCDG_Het=0;Best_CCDG_HomAlt=0;Best_CCDG_PopMax_AF=0;Best_CCDG_Mismatch_ID=505362;Best_CCDG_Mismatch_OFP=0.342044;Best_CCDG_Mismatch_SVTYPE=DUP;Best_CCDG_Mismatch_AF=0.0001368;Best_CCDG_Mismatch_Het=0;Best_CCDG_Mismatch_HomAlt=0;SV_Cov=1;CCDG_SV_Cov=1;TOPMed_Count=0;Best_TOPMed_ID=DEL_3:8677157-8681292;Best_TOPMed_OFP=0.211989;Best_TOPMed_AF=0.001843;Best_TOPMed_Het=420;Best_TOPMed_HomAlt=0;Best_TOPMed_PopMax_AF=0.00302195;Best_TOPMed_Mismatch_ID=DUP_3:8644780-8777094;Best_TOPMed_Mismatch_OFP=0.00662807;Best_TOPMed_Mismatch_SVTYPE=DUP;Best_TOPMed_Mismatch_AF=4e-06;Best_TOPMed_Mismatch_Het=1;Best_TOPMed_Mismatch_HomAlt=0;TOPMed_SV_Cov=1;ThousG_Count=0;Best_ThousG_ID=HGSV_97811;Best_ThousG_OFP=0.468643;Best_ThousG_AF=0.5392;Best_ThousG_Het=2687;Best_ThousG_HomAlt=369;Best_ThousG_PopMax_AF=0.567726;Best_ThousG_Mismatch_ID=HGSV_97810;Best_ThousG_Mismatch_OFP=0.236032;Best_ThousG_Mismatch_SVTYPE=DUP;Best_ThousG_Mismatch_AF=0.002031;Best_ThousG_Mismatch_Het=13;Best_ThousG_Mismatch_HomAlt=0;ThousG_SV_Cov=1;gnomAD_Count=0;Best_gnomAD_ID=gnomAD-SV_v3_DEL_chr3_e61292ca;Best_gnomAD_OFP=0.567845;Best_gnomAD_AF=0.573604;Best_gnomAD_Het=43550;Best_gnomAD_HomAlt=9608;Best_gnomAD_PopMax_AF=0.612903;Best_gnomAD_Mismatch_ID=gnomAD-SV_v3_DUP_chr3_75bb515c;Best_gnomAD_Mismatch_OFP=0.5439;Best_gnomAD_Mismatch_SVTYPE=DUP;Best_gnomAD_Mismatch_AF=0.039067;Best_gnomAD_Mismatch_Het=4872;Best_gnomAD_Mismatch_HomAlt=27;gnomAD_SV_Cov=1	GT:PSV:LN:DR:ST:QV:TY:ID:RAL:AAL:CO	1/1:NA:877:0,0:+:209:DEL:DEL00009230:NA:NA:chr3_8678474-chr3_8679351	1/1:NA:876:0,0:+:932:DEL:MantaDEL_157697_0_1_0_0_0:GGACCCCCATTGCAAGGGGGTGAGGCACCCCCCGCGAGGCGGGGACTGAGAGCCAGCCCCTCTTCCCTCCATGGCTCTTAGGACCCCAATCACAGAGGGGGGAACACCCCCCGCGAGGCAGGGACTGAGAGCCAGCCCTTCTTCCCCCCCGGCTTTTGGGACCCCCATCGCAGTGGGGGGAGGCACCCCCCGCGAGGCGGGGATTGAGAGCCAGCCACTCTTCCCCTCCTGGGTCTTAGGACCCCCAACGTGGGGGGGGGAGGCACCACACGCGAGGCGGGGAAAGAGAGCCAGCCCCTCTTCCCTCCCTGCCACTTAGGACCCCATCGCAGGGAGAGGAGGCGGCACTCCCCACGAGGCGGGGACTGAGAACCAATCCCTCTTCCCCCAGCCTGGCTCTTAGGATCCCCATTGCAAGGGAGGGAGGCACCCCCCGCCAGGCGGGGACTGAGAGCCAGCCACTCTTCCCCTCCTGGCTCTTGGGACGCCCATCGCAGGGCGGAGAGTCACCCCCCGCGAGGTGGGGACTGAGAGCCAGCCCCTCTTCCCCCCCTGGCTCTTAGGACACCAAACGCAGGGGAGGAAGCACCCCCCGCGAGGCGGGGACTGAGAGCAAGCACCTCTTTCCCCCCTGGCTCTTAGGACCCAAATTGCGGGGGGGAGGCACCCCCGCGAGGCGGGGACTGAGAGGCAGCCGCTGTTCCCCCACACTGGCTCTTGGGACCCCCATCGCAGGGGGGATGGCACCCTCCGTGAGCGGGGACTGAGAGCCAGCCAATTTTCCCCCTGGCTTTTAGGACCCCCATCGGAGGGGGGGAGCCACCCCCCATGAGGCGGGGACTAAGAGCCAGCCCCTCTTCCCGCCCCTTGCTCTTCC:G:chr3_8678474-chr3_8679350	0/1:NA:867:0,0:+:731:DEL:1878:NA:NA:chr3_8678483-chr3_8679350	split	p25.3	SSUH2				XM_017006511	2	8619385	8679868	878	0	0	no	20	intron1-intron1	5'UTR	353	5'	8678473	8679351																										DDD:9536;dbVar	chr3:8611079-8688004;chr3:8629543-8715948;3:8670939-8681891;chr3:8677066-8679629;chr3:8678456-8679420	0.0142	DDD:9538;dbVar;esv2676237	chr3:8615314-8716514;chr3:8677123-8681253;chr3:8677685-8687052;chr3:8677729-8687092;3:8678260-8682556;3:8678416-8682364	0.0476				IMH	3:7779573-24170164	0.1237																										72.66				-1.07437	-1.15625	dentin dysplasia type I	AD	Supportive	27680507[PMID]	51066						6	3.99E-10	2.49E-08	0.6861		Cleft lip;Cleft palate;Hypertelorism;Micrognathia;Microphthalmia;abnormal tooth morphology	Micrognathia;ceratobranchial 5 tooth absent, abnormal			full=3	0/1
16_2467675_2468572_DUP_1	16	2467675	2468572	895	DUP	ILS_AP001,ILS_AP001_1,ILS_AP001_2	MantaDUP:TANDEM:56354:0:1:2:0:0	A		1380	PASS	SUPP=3;SUPP_VEC=111;SVLEN=895;SVTYPE=DUP;SVMETHOD=SURVIVOR1.0.7;CHR2=16;END=2468570;CIPOS=0,1;CIEND=0,2;STRANDS=-;Max_AF=0.0003419;Max_Het=5;Max_HomAlt=7;Max_PopMax_AF=0.003858;CCDG_Count=1;Best_CCDG_ID=481787;Best_CCDG_OFP=1;Best_CCDG_AF=0.0003419;Best_CCDG_Het=0;Best_CCDG_HomAlt=0;Best_CCDG_PopMax_AF=0;Best_CCDG_Mismatch_ID=221169;Best_CCDG_Mismatch_OFP=0.198934;Best_CCDG_Mismatch_SVTYPE=DEL;Best_CCDG_Mismatch_AF=0.0001368;Best_CCDG_Mismatch_Het=0;Best_CCDG_Mismatch_HomAlt=0;SV_Cov=1;CCDG_SV_Cov=1;TOPMed_Count=0;Best_TOPMed_Mismatch_ID=DEL_16:2465690-2470192;Best_TOPMed_Mismatch_OFP=0.198934;Best_TOPMed_Mismatch_SVTYPE=DEL;Best_TOPMed_Mismatch_AF=4.6e-05;Best_TOPMed_Mismatch_Het=13;Best_TOPMed_Mismatch_HomAlt=0;TOPMed_SV_Cov=0;ThousG_Count=0;Best_ThousG_Mismatch_ID=HGSV_54243;Best_ThousG_Mismatch_OFP=0.000139509;Best_ThousG_Mismatch_SVTYPE=CPX;Best_ThousG_Mismatch_AF=0.000781;Best_ThousG_Mismatch_Het=5;Best_ThousG_Mismatch_HomAlt=0;ThousG_SV_Cov=0;gnomAD_Count=1;Best_gnomAD_ID=gnomAD-SV_v3_DUP_chr16_bfcd0fc2;Best_gnomAD_OFP=1;Best_gnomAD_AF=0.000151;Best_gnomAD_Het=5;Best_gnomAD_HomAlt=7;Best_gnomAD_PopMax_AF=0.003858;Best_gnomAD_Mismatch_ID=gnomAD-SV_v3_DEL_chr16_a2b17a52;Best_gnomAD_Mismatch_OFP=0.565953;Best_gnomAD_Mismatch_SVTYPE=DEL;Best_gnomAD_Mismatch_AF=0.000112;Best_gnomAD_Mismatch_Het=12;Best_gnomAD_Mismatch_HomAlt=1;gnomAD_SV_Cov=1	GT:PSV:LN:DR:ST:QV:TY:ID:RAL:AAL:CO	0/1:NA:896:29,11:-:1380:DUP:DUP00045666:NA:NA:chr16_2467676-chr16_2468572	0/1:NA:895:0,0:-:681:DUP:MantaDUP_TANDEM_56354_0_1_2_0_0:NA:NA:chr16_2467675-chr16_2468570	0/1:NA:895:0,0:-:237:DUP:8673:NA:NA:chr16_2467675-chr16_2468570	split	p13.3	TEDC2-AS1				NR_135198	1	2464949	2468213	539	0	0	no	1	txStart-exon1	UTR	539	3'	2467674	2468213																													dbVar	chr16:2465689-2470192	0.01																																										729652									0						full=3	0/1
16_2467665_2468169_DEL_1	16	2467665	2468169	-503	DEL	ILS_AP001,ILS_AP001_1,ILS_AP001_2	MantaDEL:56354:0:1:0:1:0	ACCCAGAGGTAGCAGCTGACTCCTCGGGCTCCCGGCTGGCGTCACCGCCTCTGGAGCCAATGGGCTCTGACGTGGCGAGCCTGGAACGCCACTGGGACTGCGCGGGCGTCCACACCACCCACCGAGCGCGGCCACCGCGTTGGCGGCATGAGCGCGCTCGAGCCTCCCCCAGCGGCGCCCGCCTCGCCCCCCGGGCCGGGTCTGGCGCACCCGGAACCCCCCGGGGCCCTGCGCGCCCTGCTCTTGGCCACCGGCGGCGCTGGGGGCTCGTGGCCTCGCTGCATTCTGCCGTTCGGTGCCGTGGCCGTGCTGGCTGGTGCGGCTGCCACGGCCATCACCTTCTCGCTGCGGGGACCCCGCCTGGACCTGGCCCAGGGCTCGTCGCTGGCCGCGCTCAGTGCGGGCCTGGGGCTTCTGACCGCCGCCTTCCTGTGCTGGCGCGCTCGGCGGAGGCGAAGGGCCGGGCGCAGGGAGCCGGGGGCGCCCTGAGCCGCGGGCGAGGCC	A	458	PASS	SUPP=3;SUPP_VEC=111;SVLEN=-503;SVTYPE=DEL;SVMETHOD=SURVIVOR1.0.7;CHR2=16;END=2468168;CIPOS=0,3;CIEND=0,1;STRANDS=+;Max_AF=0.000112;Max_Het=12;Max_HomAlt=1;Max_PopMax_AF=0.00273;CCDG_Count=0;Best_CCDG_ID=221169;Best_CCDG_OFP=0.111901;Best_CCDG_AF=0.0001368;Best_CCDG_Het=0;Best_CCDG_HomAlt=0;Best_CCDG_PopMax_AF=0;Best_CCDG_Mismatch_ID=481787;Best_CCDG_Mismatch_OFP=0.5404;Best_CCDG_Mismatch_SVTYPE=DUP;Best_CCDG_Mismatch_AF=0.0003419;Best_CCDG_Mismatch_Het=0;Best_CCDG_Mismatch_HomAlt=0;SV_Cov=1;CCDG_SV_Cov=1;TOPMed_Count=0;Best_TOPMed_ID=DEL_16:2465690-2470192;Best_TOPMed_OFP=0.111901;Best_TOPMed_AF=4.6e-05;Best_TOPMed_Het=13;Best_TOPMed_HomAlt=0;Best_TOPMed_PopMax_AF=0.000119907;TOPMed_SV_Cov=1;ThousG_Count=0;Best_ThousG_Mismatch_ID=HGSV_54243;Best_ThousG_Mismatch_OFP=0.015873;Best_ThousG_Mismatch_SVTYPE=CPX;Best_ThousG_Mismatch_AF=0.000781;Best_ThousG_Mismatch_Het=5;Best_ThousG_Mismatch_HomAlt=0;ThousG_SV_Cov=0;gnomAD_Count=1;Best_gnomAD_ID=gnomAD-SV_v3_DEL_chr16_a2b17a52;Best_gnomAD_OFP=0.955888;Best_gnomAD_AF=0.000112;Best_gnomAD_Het=12;Best_gnomAD_HomAlt=1;Best_gnomAD_PopMax_AF=0.00273;Best_gnomAD_Mismatch_ID=gnomAD-SV_v3_DUP_chr16_bfcd0fc2;Best_gnomAD_Mismatch_OFP=0.5404;Best_gnomAD_Mismatch_SVTYPE=DUP;Best_gnomAD_Mismatch_AF=0.000151;Best_gnomAD_Mismatch_Het=5;Best_gnomAD_Mismatch_HomAlt=7;gnomAD_SV_Cov=1	GT:PSV:LN:DR:ST:QV:TY:ID:RAL:AAL:CO	0/1:NA:504:23,4:+:420:DEL:DEL00045665:NA:NA:chr16_2467665-chr16_2468169	0/1:NA:503:0,0:+:238:DEL:MantaDEL_56354_0_1_0_1_0:ACCCAGAGGTAGCAGCTGACTCCTCGGGCTCCCGGCTGGCGTCACCGCCTCTGGAGCCAATGGGCTCTGACGTGGCGAGCCTGGAACGCCACTGGGACTGCGCGGGCGTCCACACCACCCACCGAGCGCGGCCACCGCGTTGGCGGCATGAGCGCGCTCGAGCCTCCCCCAGCGGCGCCCGCCTCGCCCCCCGGGCCGGGTCTGGCGCACCCGGAACCCCCCGGGGCCCTGCGCGCCCTGCTCTTGGCCACCGGCGGCGCTGGGGGCTCGTGGCCTCGCTGCATTCTGCCGTTCGGTGCCGTGGCCGTGCTGGCTGGTGCGGCTGCCACGGCCATCACCTTCTCGCTGCGGGGACCCCGCCTGGACCTGGCCCAGGGCTCGTCGCTGGCCGCGCTCAGTGCGGGCCTGGGGCTTCTGACCGCCGCCTTCCTGTGCTGGCGCGCTCGGCGGAGGCGAAGGGCCGGGCGCAGGGAGCCGGGGGCGCCCTGAGCCGCGGGCGAGGCC:A:chr16_2467665-chr16_2468168	0/1:NA:500:0,0:+:458:DEL:8672:NA:NA:chr16_2467668-chr16_2468168	split	p13.3	TEDC2-AS1				NR_135198	1	2464949	2468213	505	0	0	no	1	exon1-exon1	UTR	44	3'	2467664	2468169																													dbVar	chr16:2465689-2470192	0.01																																										729652									0						full=1	0/1
5_110536114_110536179_DEL_1	5	1.11E+08	1.11E+08	-64	DEL	ILS_AP001,ILS_AP001_1,ILS_AP001_2	MantaDEL:138498:0:1:0:0:0	TCTTTTTCTGTCTTTTCCTTTCCAACGTGGGACCCTTGGTAGGCAGTGCCTAAACACAGAGGCAA	T	600	PASS	SUPP=3;SUPP_VEC=111;SVLEN=-64;SVTYPE=DEL;SVMETHOD=SURVIVOR1.0.7;CHR2=5;END=110536178;CIPOS=0,2;CIEND=0,1;STRANDS=+;Max_AF=0.002343;Max_Het=47;Max_HomAlt=1;Max_PopMax_AF=0.012521;CCDG_Count=0;Best_CCDG_ID=362171;Best_CCDG_OFP=0.000803938;Best_CCDG_AF=3.419e-05;Best_CCDG_Het=0;Best_CCDG_HomAlt=0;Best_CCDG_PopMax_AF=0;Best_CCDG_Mismatch_ID=362176_1;Best_CCDG_Mismatch_OFP=0.0153846;Best_CCDG_Mismatch_SVTYPE=BND;Best_CCDG_Mismatch_AF=0.0003419;Best_CCDG_Mismatch_Het=0;Best_CCDG_Mismatch_HomAlt=0;SV_Cov=1;CCDG_SV_Cov=1;TOPMed_Count=0;Best_TOPMed_ID=DEL_5:110533678-110539832;Best_TOPMed_OFP=0.0105588;Best_TOPMed_AF=1.1e-05;Best_TOPMed_Het=3;Best_TOPMed_HomAlt=0;Best_TOPMed_PopMax_AF=3.5929e-05;Best_TOPMed_Mismatch_ID=DUP_5:110530357-110547459;Best_TOPMed_Mismatch_OFP=0.00380028;Best_TOPMed_Mismatch_SVTYPE=DUP;Best_TOPMed_Mismatch_AF=4e-06;Best_TOPMed_Mismatch_Het=1;Best_TOPMed_Mismatch_HomAlt=0;TOPMed_SV_Cov=1;ThousG_Count=1;Best_ThousG_ID=HGSV_125856;Best_ThousG_OFP=0.969231;Best_ThousG_AF=0.002343;Best_ThousG_Het=15;Best_ThousG_HomAlt=0;Best_ThousG_PopMax_AF=0.012521;Best_ThousG_Mismatch_ID=HGSV_125854;Best_ThousG_Mismatch_OFP=0.00379961;Best_ThousG_Mismatch_SVTYPE=DUP;Best_ThousG_Mismatch_AF=0.000312;Best_ThousG_Mismatch_Het=2;Best_ThousG_Mismatch_HomAlt=0;ThousG_SV_Cov=1;gnomAD_Count=1;Best_gnomAD_ID=gnomAD-SV_v3_DEL_chr5_0be7075e;Best_gnomAD_OFP=0.955882;Best_gnomAD_AF=0.000389;Best_gnomAD_Het=47;Best_gnomAD_HomAlt=1;Best_gnomAD_PopMax_AF=0.01;Best_gnomAD_Mismatch_ID=gnomAD-SV_v3_DUP_chr5_9c1766b7;Best_gnomAD_Mismatch_OFP=0.00379961;Best_gnomAD_Mismatch_SVTYPE=DUP;Best_gnomAD_Mismatch_AF=8e-06;Best_gnomAD_Mismatch_Het=1;Best_gnomAD_Mismatch_HomAlt=0;gnomAD_SV_Cov=1	GT:PSV:LN:DR:ST:QV:TY:ID:RAL:AAL:CO	0/1:NA:65:0,0:+:600:DEL:DEL00017207:NA:NA:chr5_110536114-chr5_110536179	0/1:NA:64:0,0:+:400:DEL:MantaDEL_138498_0_1_0_0_0:TCTTTTTCTGTCTTTTCCTTTCCAACGTGGGACCCTTGGTAGGCAGTGCCTAAACACAGAGGCAA:T:chr5_110536114-chr5_110536178	0/1:NA:62:0,0:+:251:DEL:3385:NA:NA:chr5_110536116-chr5_110536178	split	q22.1	TMEM232				XR_001742182	2	1.1E+08	1.11E+08	66	0	0	no	16	intron14-intron14	UTR	32267	5'	1.11E+08	1.11E+08																													dbVar	chr5:110269511-110611886;chr5:110415987-110736461	0.01				IMH	5:85192778-162907706;5:96855635-159536527;5:99219064-135784342;5:99223293-135778815	0.5229																										77.61										642987						6	7.93E-12		0						full=1	0/1
14_90700685_90700781_DEL_1	14	90700685	90700781	-95	DEL	ILS_AP001,ILS_AP001_1,ILS_AP001_2	MantaDEL:77015:0:1:0:0:0	TATGTATCTGCTTTTCCAGAGTTGTAAGTGTTGACAAAGACAATGTCATTCTGGATATTGTCAACACTTACAACTCCGGAAAAGCAGATACATACC	T	240	PASS	SUPP=3;SUPP_VEC=111;SVLEN=-95;SVTYPE=DEL;SVMETHOD=SURVIVOR1.0.7;CHR2=14;END=90700780;CIPOS=0,8;CIEND=0,1;STRANDS=+;Max_AF=0;Max_Het=0;Max_HomAlt=0;Max_PopMax_AF=0;CCDG_Count=0;Best_CCDG_Mismatch_ID=208341_1;Best_CCDG_Mismatch_OFP=0.0104167;Best_CCDG_Mismatch_SVTYPE=BND;Best_CCDG_Mismatch_AF=0.08852;Best_CCDG_Mismatch_Het=0;Best_CCDG_Mismatch_HomAlt=0;SV_Cov=1;CCDG_SV_Cov=0;TOPMed_Count=0;Best_TOPMed_ID=DEL_14:90676902-90708200;Best_TOPMed_OFP=0.00306709;Best_TOPMed_AF=4e-06;Best_TOPMed_Het=1;Best_TOPMed_HomAlt=0;Best_TOPMed_PopMax_AF=1.19772e-05;Best_TOPMed_Mismatch_ID=INV_14:90104469-93174563;Best_TOPMed_Mismatch_OFP=3.12694e-05;Best_TOPMed_Mismatch_SVTYPE=INV;Best_TOPMed_Mismatch_AF=7e-06;Best_TOPMed_Mismatch_Het=2;Best_TOPMed_Mismatch_HomAlt=0;TOPMed_SV_Cov=1;ThousG_Count=0;Best_ThousG_Mismatch_ID=HGSV_47084;Best_ThousG_Mismatch_OFP=3.6584e-06;Best_ThousG_Mismatch_SVTYPE=DUP;Best_ThousG_Mismatch_AF=0.000312;Best_ThousG_Mismatch_Het=2;Best_ThousG_Mismatch_HomAlt=0;ThousG_SV_Cov=0;gnomAD_Count=0;Best_gnomAD_ID=gnomAD-SV_v3_DEL_chr14_a1dd9329;Best_gnomAD_OFP=4.75114e-06;Best_gnomAD_AF=0.000141;Best_gnomAD_Het=16;Best_gnomAD_HomAlt=0;Best_gnomAD_PopMax_AF=0.000682;Best_gnomAD_Mismatch_ID=gnomAD-SV_v3_CPX_chr14_821da3bd;Best_gnomAD_Mismatch_OFP=1.57307e-06;Best_gnomAD_Mismatch_SVTYPE=CPX;Best_gnomAD_Mismatch_AF=8e-06;Best_gnomAD_Mismatch_Het=1;Best_gnomAD_Mismatch_HomAlt=0;gnomAD_SV_Cov=1	GT:PSV:LN:DR:ST:QV:TY:ID:RAL:AAL:CO	0/1:NA:96:0,0:+:240:DEL:DEL00042995:NA:NA:chr14_90700685-chr14_90700781	0/1:NA:95:0,0:+:133:DEL:MantaDEL_77015_0_1_0_0_0:TATGTATCTGCTTTTCCAGAGTTGTAAGTGTTGACAAAGACAATGTCATTCTGGATATTGTCAACACTTACAACTCCGGAAAAGCAGATACATACC:T:chr14_90700685-chr14_90700780	0/1:NA:87:0,0:+:128:DEL:8205:NA:NA:chr14_90700693-chr14_90700780	split	q32.11	TTC7B				NM_001010854	2	90524563	90816430	97	0	0	no	20	intron5-intron5	CDS	5106	3'	90700684	90700781																													gnomAD-SV_v3_DEL_chr14_aa7bf92b	chr14:56471274-99420950	0.633				IMH	14:23629193-97505158	0.4022																										30.86	1.071135	-0.91594	-0.36248	-0.28547	2.065498					145567						0	1.00E+00	1.00E+00	0						full=1	0/1
16_78728053_78738602_DEL_1	16	78728053	78738602	-10548	DEL	ILS_AP001,ILS_AP001_1,ILS_AP001_2	MantaDEL:62766:0:1:0:0:0	T		1800	PASS	SUPP=3;SUPP_VEC=111;SVLEN=-10548;SVTYPE=DEL;SVMETHOD=SURVIVOR1.0.7;CHR2=16;END=78738601;CIPOS=0,3;CIEND=0,1;STRANDS=+;Max_AF=0.001873;Max_Het=123;Max_HomAlt=0;Max_PopMax_AF=0.008547;CCDG_Count=0;Best_CCDG_ID=230135;Best_CCDG_OFP=0.314722;Best_CCDG_AF=3.419e-05;Best_CCDG_Het=0;Best_CCDG_HomAlt=0;Best_CCDG_PopMax_AF=0;Best_CCDG_Mismatch_ID=484122;Best_CCDG_Mismatch_OFP=0.0155123;Best_CCDG_Mismatch_SVTYPE=DUP;Best_CCDG_Mismatch_AF=3.419e-05;Best_CCDG_Mismatch_Het=0;Best_CCDG_Mismatch_HomAlt=0;SV_Cov=1;CCDG_SV_Cov=1;TOPMed_Count=1;Best_TOPMed_ID=DEL_16:78728057-78738605;Best_TOPMed_OFP=0.999337;Best_TOPMed_AF=0.000436;Best_TOPMed_Het=123;Best_TOPMed_HomAlt=0;Best_TOPMed_PopMax_AF=0.00611532;Best_TOPMed_Mismatch_ID=DUP_16:78644060-78859840;Best_TOPMed_Mismatch_OFP=0.0488873;Best_TOPMed_Mismatch_SVTYPE=DUP;Best_TOPMed_Mismatch_AF=4e-06;Best_TOPMed_Mismatch_Het=1;Best_TOPMed_Mismatch_HomAlt=0;TOPMed_SV_Cov=1;ThousG_Count=1;Best_ThousG_ID=HGSV_58285;Best_ThousG_OFP=0.999716;Best_ThousG_AF=0.001873;Best_ThousG_Het=12;Best_ThousG_HomAlt=0;Best_ThousG_PopMax_AF=0.008547;Best_ThousG_Mismatch_ID=HGSV_58282;Best_ThousG_Mismatch_OFP=0.00994917;Best_ThousG_Mismatch_SVTYPE=DUP;Best_ThousG_Mismatch_AF=0.000312;Best_ThousG_Mismatch_Het=2;Best_ThousG_Mismatch_HomAlt=0;ThousG_SV_Cov=1;gnomAD_Count=1;Best_gnomAD_ID=gnomAD-SV_v3_DEL_chr16_c42f3f93;Best_gnomAD_OFP=0.999716;Best_gnomAD_AF=0.000389;Best_gnomAD_Het=49;Best_gnomAD_HomAlt=0;Best_gnomAD_PopMax_AF=0.007893;Best_gnomAD_Mismatch_ID=gnomAD-SV_v3_DUP_chr16_b692be56;Best_gnomAD_Mismatch_OFP=0.29302;Best_gnomAD_Mismatch_SVTYPE=DUP;Best_gnomAD_Mismatch_AF=8e-06;Best_gnomAD_Mismatch_Het=1;Best_gnomAD_Mismatch_HomAlt=0;gnomAD_SV_Cov=1	GT:PSV:LN:DR:ST:QV:TY:ID:RAL:AAL:CO	0/1:NA:10549:13,16:+:1800:DEL:DEL00048734:NA:NA:chr16_78728053-chr16_78738602	0/1:NA:10548:0,0:+:636:DEL:MantaDEL_62766_0_1_0_0_0:NA:NA:chr16_78728053-chr16_78738601	0/1:NA:10545:0,0:+:665:DEL:9063:NA:NA:chr16_78728056-chr16_78738601	split	q23.1	WWOX				NM_001291997	2	78099653	79212667	10550	0	0	no	8	intron7-intron7	CDS	295300	5'	78728052	78738602																										dbVar	chr16:77756392-78780785	0.01	dbVar	chr16:78522932-78900915;chr16:78680867-78881706;chr16:78727479-78738769;chr16:78728023-78738636	0.01				IMH	16:29226964-88193867	0.4997																								30	0	0.38				-3.32067	-3.50356	46,XY partial gonadal dysgenesis;autosomal recessive spinocerebellar ataxia 12;developmental and epileptic encephalopathy;developmental and epileptic encephalopathy, 28;undetermined early-onset epileptic encephalopathy	AD;AR	Definitive;Moderate;Strong;Supportive	22071891[PMID];24369382;24369382[PMID];24456803;25411445;25411445[PMID]	51741	605131	Developmental and epileptic encephalopathy 28, 616211 (3) AR;Esophageal squamous cell carcinoma, somatic, 133239 (3);Spinocerebellar ataxia, AR 12, 614322 (3) AR	AR	yes		8	1.24E-15	1.94E-08	0.4271	Cleft lip;Cleft palate;Developmental and epileptic encephalopathy 28;Long philtrum	Holoprosencephaly;increased brain weight	Microphthalmia;eye decreased size, abnormal			full=1	0/1
16_69820426_69824902_DEL_1	16	69820426	69824902	-4474	DEL	ILS_AP001,ILS_AP001_1,ILS_AP001_2	MantaDEL:61841:0:1:0:0:0	G		1773	PASS	SUPP=3;SUPP_VEC=111;SVLEN=-4474;SVTYPE=DEL;SVMETHOD=SURVIVOR1.0.7;CHR2=16;END=69824902;CIPOS=-2,0;CIEND=-2,0;STRANDS=+;Max_AF=0.001768;Max_Het=205;Max_HomAlt=1;Max_PopMax_AF=0.004054;CCDG_Count=0;Best_CCDG_Mismatch_ID=483870;Best_CCDG_Mismatch_OFP=0.452395;Best_CCDG_Mismatch_SVTYPE=DUP;Best_CCDG_Mismatch_AF=0.005368;Best_CCDG_Mismatch_Het=0;Best_CCDG_Mismatch_HomAlt=0;SV_Cov=1;CCDG_SV_Cov=0;TOPMed_Count=0;Best_TOPMed_ID=DEL_16:69819704-69827126;Best_TOPMed_OFP=0.602775;Best_TOPMed_AF=4e-06;Best_TOPMed_Het=1;Best_TOPMed_HomAlt=0;Best_TOPMed_PopMax_AF=0;Best_TOPMed_Mismatch_ID=DUP_16:69677028-69924914;Best_TOPMed_Mismatch_OFP=0.0180525;Best_TOPMed_Mismatch_SVTYPE=DUP;Best_TOPMed_Mismatch_AF=7e-06;Best_TOPMed_Mismatch_Het=2;Best_TOPMed_Mismatch_HomAlt=0;TOPMed_SV_Cov=1;ThousG_Count=0;Best_ThousG_Mismatch_ID=HGSV_57643;Best_ThousG_Mismatch_OFP=0.442054;Best_ThousG_Mismatch_SVTYPE=DUP;Best_ThousG_Mismatch_AF=0.004995;Best_ThousG_Mismatch_Het=30;Best_ThousG_Mismatch_HomAlt=1;ThousG_SV_Cov=0;gnomAD_Count=3;Best_gnomAD_ID=gnomAD-SV_v3_DEL_chr16_a548fb8d;Best_gnomAD_OFP=0.918212;Best_gnomAD_AF=0.001768;Best_gnomAD_Het=205;Best_gnomAD_HomAlt=1;Best_gnomAD_PopMax_AF=0.004054;gnomAD_Mismatches=gnomAD-SV_v3_DUP_chr16_713063eb;gnomAD_Mismatches_Count=1;gnomAD_Mismatch_SVTYPEs=DUP;Best_gnomAD_Mismatch_ID=gnomAD-SV_v3_DUP_chr16_713063eb;Best_gnomAD_Mismatch_OFP=0.87503;Best_gnomAD_Mismatch_SVTYPE=DUP;Best_gnomAD_Mismatch_AF=0.069756;Best_gnomAD_Mismatch_Het=2968;Best_gnomAD_Mismatch_HomAlt=1072;gnomAD_SV_Cov=1	GT:PSV:LN:DR:ST:QV:TY:ID:RAL:AAL:CO	0/1:NA:4474:15,18:+:1773:DEL:DEL00048414:NA:NA:chr16_69820426-chr16_69824900	0/1:NA:4474:0,0:+:999:DEL:MantaDEL_61841_0_1_0_0_0:NA:NA:chr16_69820428-chr16_69824902	0/1:NA:4474:0,0:+:983:DEL:9002:NA:NA:chr16_69820428-chr16_69824902	split	q22.1	WWP2				NM_001270455	2	69762331	69910405	4477	0	0	no	9	intron4-intron4	CDS	15223	3'	69820425	69824902																										dbVar	chr16:69677025-69924863;chr16:69762176-69924258;chr16:69815142-69824970	0.01	dbVar	chr16:69820414-69824905	0.01				IMH	16:15020811-70019806;16:29226964-88193867	0.4997																										16.36				1.36334	1.814698					11060						1	1.65E-01	9.78E-01	0.6812		Cleft lip;Cleft palate;Hypertelorism;Micrognathia;Microphthalmia;abnormal jaw morphology;abnormal snout morphology	Cleft palate;palate decreased size, abnormal			full=3	0/1
19_4005348_4159440_DEL_1	19	4005348	4159440	-154090	DEL	ILS_AP001,ILS_AP001_1,ILS_AP001_2	MantaDEL:79083:0:1:0:0:0	C		1740	PASS	SUPP=3;SUPP_VEC=111;SVLEN=-154090;SVTYPE=DEL;SVMETHOD=SURVIVOR1.0.7;CHR2=19;END=4159440;CIPOS=-2,0;CIEND=-5,0;STRANDS=+;Max_AF=0;Max_Het=0;Max_HomAlt=0;Max_PopMax_AF=0;CCDG_Count=0;Best_CCDG_ID=253950;Best_CCDG_OFP=0.113861;Best_CCDG_AF=6.839e-05;Best_CCDG_Het=0;Best_CCDG_HomAlt=0;Best_CCDG_PopMax_AF=0;Best_CCDG_Mismatch_ID=490213;Best_CCDG_Mismatch_OFP=0.150976;Best_CCDG_Mismatch_SVTYPE=DUP;Best_CCDG_Mismatch_AF=3.419e-05;Best_CCDG_Mismatch_Het=0;Best_CCDG_Mismatch_HomAlt=0;SV_Cov=0.735014;CCDG_SV_Cov=0.290815;TOPMed_Count=0;Best_TOPMed_ID=DEL_19:4130566-4146337;Best_TOPMed_OFP=0.102362;Best_TOPMed_AF=4e-06;Best_TOPMed_Het=1;Best_TOPMed_HomAlt=0;Best_TOPMed_PopMax_AF=0.000108719;Best_TOPMed_Mismatch_ID=INV_19:3851264-4364229;Best_TOPMed_Mismatch_OFP=0.300392;Best_TOPMed_Mismatch_SVTYPE=INV;Best_TOPMed_Mismatch_AF=4e-06;Best_TOPMed_Mismatch_Het=1;Best_TOPMed_Mismatch_HomAlt=0;TOPMed_SV_Cov=0.400108;ThousG_Count=0;Best_ThousG_ID=HGSV_70074;Best_ThousG_OFP=0.0204879;Best_ThousG_AF=0.000312;Best_ThousG_Het=2;Best_ThousG_HomAlt=0;Best_ThousG_PopMax_AF=0.00112;Best_ThousG_Mismatch_ID=HGSV_70075;Best_ThousG_Mismatch_OFP=0.148371;Best_ThousG_Mismatch_SVTYPE=DUP;Best_ThousG_Mismatch_AF=0.000312;Best_ThousG_Mismatch_Het=2;Best_ThousG_Mismatch_HomAlt=0;ThousG_SV_Cov=0.0925492;gnomAD_Count=0;Best_gnomAD_ID=gnomAD-SV_v3_DEL_chr19_5ba69aa0;Best_gnomAD_OFP=0.172119;Best_gnomAD_AF=6.3e-05;Best_gnomAD_Het=8;Best_gnomAD_HomAlt=0;Best_gnomAD_PopMax_AF=0.000493;Best_gnomAD_Mismatch_ID=gnomAD-SV_v3_DUP_chr19_9114b2c5;Best_gnomAD_Mismatch_OFP=0.367091;Best_gnomAD_Mismatch_SVTYPE=DUP;Best_gnomAD_Mismatch_AF=8e-06;Best_gnomAD_Mismatch_Het=1;Best_gnomAD_Mismatch_HomAlt=0;gnomAD_SV_Cov=0.733638	GT:PSV:LN:DR:ST:QV:TY:ID:RAL:AAL:CO	0/1:NA:154087:26,11:+:1740:DEL:DEL00054230:NA:NA:chr19_4005348-chr19_4159435	0/1:NA:154090:0,0:+:999:DEL:MantaDEL_79083_0_1_0_0_0:NA:NA:chr19_4005350-chr19_4159440	0/1:NA:154090:0,0:+:650:DEL:10046:NA:NA:chr19_4005350-chr19_4159440	split	p13.3	ZBTB7A				XM_005259570	6	4043302	4055521	12219	1905	100	no	3	txStart-txEnd	5'UTR-3'UTR			4043302	4055521																								2	Neurodevelopmental_delay	dbVar	chr19:4019786-4065415	0.01							IMH	19:1692676-41507766	0.0103																										46.6	0.025969	0.397011	0.402117			complex neurodevelopmental disorder;macrocephaly, neurodevelopmental delay, lymphoid hyperplasia, and persistent fetal hemoglobin	AD	Strong	27252013;28135719;29738522;31645653;34515416	51341	605878	Macrocephaly, neurodevelopmental delay, lymphoid hyperplasia, and persistent fetal hemoglobin, 619769 (3) AD	AD	yes		1	9.59E-01		0.3097	Dental crowding;Macrocephaly, neurodevelopmental delay, lymphoid hyperplasia, and persistent fetal hemoglobin;Micrognathia					full=5	0/1
19_4005230_4159441_DUP_1	19	4005230	4159441	154171	DUP	ILS_AP001,ILS_AP001_1,ILS_AP001_2	MantaDUP:TANDEM:79083:0:1:1:0:0	T		999	PASS	SUPP=3;SUPP_VEC=111;SVLEN=154171;SVTYPE=DUP;SVMETHOD=SURVIVOR1.0.7;CHR2=19;END=4159423;CIPOS=-22,1;CIEND=0,18;STRANDS=-;Max_AF=0;Max_Het=0;Max_HomAlt=0;Max_PopMax_AF=0;CCDG_Count=0;Best_CCDG_ID=490213;Best_CCDG_OFP=0.150896;Best_CCDG_AF=3.419e-05;Best_CCDG_Het=0;Best_CCDG_HomAlt=0;Best_CCDG_PopMax_AF=0;Best_CCDG_Mismatch_ID=253950;Best_CCDG_Mismatch_OFP=0.113801;Best_CCDG_Mismatch_SVTYPE=DEL;Best_CCDG_Mismatch_AF=6.839e-05;Best_CCDG_Mismatch_Het=0;Best_CCDG_Mismatch_HomAlt=0;SV_Cov=0.97591;CCDG_SV_Cov=0.202644;TOPMed_Count=0;Best_TOPMed_ID=DUP_19:4084740-4226078;Best_TOPMed_OFP=0.255974;Best_TOPMed_AF=4e-06;Best_TOPMed_Het=1;Best_TOPMed_HomAlt=0;Best_TOPMed_PopMax_AF=6.35898e-06;Best_TOPMed_Mismatch_ID=INV_19:3851264-4364229;Best_TOPMed_Mismatch_OFP=0.30055;Best_TOPMed_Mismatch_SVTYPE=INV;Best_TOPMed_Mismatch_AF=4e-06;Best_TOPMed_Mismatch_Het=1;Best_TOPMed_Mismatch_HomAlt=0;TOPMed_SV_Cov=0.741834;ThousG_Count=0;Best_ThousG_ID=HGSV_70075;Best_ThousG_OFP=0.14813;Best_ThousG_AF=0.000312;Best_ThousG_Het=2;Best_ThousG_HomAlt=0;Best_ThousG_PopMax_AF=0.002041;Best_ThousG_Mismatch_ID=HGSV_70074;Best_ThousG_Mismatch_OFP=0.0204771;Best_ThousG_Mismatch_SVTYPE=DEL;Best_ThousG_Mismatch_AF=0.000312;Best_ThousG_Mismatch_Het=2;Best_ThousG_Mismatch_HomAlt=0;ThousG_SV_Cov=0.285162;gnomAD_Count=0;Best_gnomAD_ID=gnomAD-SV_v3_DUP_chr19_9114b2c5;Best_gnomAD_OFP=0.36671;Best_gnomAD_AF=8e-06;Best_gnomAD_Het=1;Best_gnomAD_HomAlt=0;Best_gnomAD_PopMax_AF=7.9e-05;Best_gnomAD_Mismatch_ID=gnomAD-SV_v3_DEL_chr19_5ba69aa0;Best_gnomAD_Mismatch_OFP=0.172029;Best_gnomAD_Mismatch_SVTYPE=DEL;Best_gnomAD_Mismatch_AF=6.3e-05;Best_gnomAD_Mismatch_Het=8;Best_gnomAD_Mismatch_HomAlt=0;gnomAD_SV_Cov=0.960771	GT:PSV:LN:DR:ST:QV:TY:ID:RAL:AAL:CO	0/1:NA:154170:21,8:-:600:DUP:DUP00054229:NA:NA:chr19_4005253-chr19_4159423	0/1:NA:154171:0,0:-:999:DUP:MantaDUP_TANDEM_79083_0_1_1_0_0:NA:NA:chr19_4005252-chr19_4159423	0/1:NA:154211:0,0:-:106:DUP:10045:NA:NA:chr19_4005230-chr19_4159441	split	p13.3	ZBTB7A				XM_005259570	6	4043302	4055521	12219	1905	100	no	3	txStart-txEnd	5'UTR-3'UTR			4043302	4055521																								2	Neurodevelopmental_delay	dbVar	chr19:4019786-4065415	0.01							IMH	19:1692676-41507766	0.0103																										46.6	0.025969	0.397011	0.402117			complex neurodevelopmental disorder;macrocephaly, neurodevelopmental delay, lymphoid hyperplasia, and persistent fetal hemoglobin	AD	Strong	27252013;28135719;29738522;31645653;34515416	51341	605878	Macrocephaly, neurodevelopmental delay, lymphoid hyperplasia, and persistent fetal hemoglobin, 619769 (3) AD	AD	yes		1	9.59E-01		0.3097	Dental crowding;Macrocephaly, neurodevelopmental delay, lymphoid hyperplasia, and persistent fetal hemoglobin;Micrognathia					full=3	0/1
8_134624854_134624959_DEL_1	8	1.35E+08	1.35E+08	-104	DEL	ILS_AP001,ILS_AP001_1,ILS_AP001_2	MantaDEL:223528:0:1:0:0:0	TCATATGCATTCATTTATAATTAACACTGCACTAATATTCTAAAGTACCTATTCTAAATTGCCCAAGAACTAATTAAATCTCAGAATGCCCCAGAGAGTAAAGTG	T	660	PASS	SUPP=3;SUPP_VEC=111;SVLEN=-104;SVTYPE=DEL;SVMETHOD=SURVIVOR1.0.7;CHR2=8;END=134624958;CIPOS=0,0;CIEND=0,1;STRANDS=+;Max_AF=0.00641;Max_Het=1273;Max_HomAlt=108;Max_PopMax_AF=0.073634;CCDG_Count=0;Best_CCDG_Mismatch_ID=534379;Best_CCDG_Mismatch_OFP=0.0102971;Best_CCDG_Mismatch_SVTYPE=DUP;Best_CCDG_Mismatch_AF=0.0001026;Best_CCDG_Mismatch_Het=0;Best_CCDG_Mismatch_HomAlt=0;SV_Cov=1;CCDG_SV_Cov=0;TOPMed_Count=1;Best_TOPMed_ID=DEL_8:134624855-134624958;Best_TOPMed_OFP=1;Best_TOPMed_AF=0.005011;Best_TOPMed_Het=1273;Best_TOPMed_HomAlt=10;Best_TOPMed_PopMax_AF=0.00766061;Best_TOPMed_Mismatch_ID=DUP_8:134613175-134625901;Best_TOPMed_Mismatch_OFP=0.00824953;Best_TOPMed_Mismatch_SVTYPE=DUP;Best_TOPMed_Mismatch_AF=4e-06;Best_TOPMed_Mismatch_Het=1;Best_TOPMed_Mismatch_HomAlt=0;TOPMed_SV_Cov=1;ThousG_Count=1;Best_ThousG_ID=HGSV_158426;Best_ThousG_OFP=1;Best_ThousG_AF=0.002029;Best_ThousG_Het=9;Best_ThousG_HomAlt=2;Best_ThousG_PopMax_AF=0.003943;ThousG_SV_Cov=1;gnomAD_Count=1;Best_gnomAD_ID=gnomAD-SV_v3_DEL_chr8_1374fbf9;Best_gnomAD_OFP=1;Best_gnomAD_AF=0.00641;Best_gnomAD_Het=592;Best_gnomAD_HomAlt=108;Best_gnomAD_PopMax_AF=0.073634;Best_gnomAD_Mismatch_ID=gnomAD-SV_v3_DUP_chr8_5d030293;Best_gnomAD_Mismatch_OFP=0.0102971;Best_gnomAD_Mismatch_SVTYPE=DUP;Best_gnomAD_Mismatch_AF=8e-06;Best_gnomAD_Mismatch_Het=1;Best_gnomAD_Mismatch_HomAlt=0;gnomAD_SV_Cov=1	GT:PSV:LN:DR:ST:QV:TY:ID:RAL:AAL:CO	0/1:NA:105:0,0:+:660:DEL:DEL00027262:NA:NA:chr8_134624854-chr8_134624959	0/1:NA:104:0,0:+:457:DEL:MantaDEL_223528_0_1_0_0_0:TCATATGCATTCATTTATAATTAACACTGCACTAATATTCTAAAGTACCTATTCTAAATTGCCCAAGAACTAATTAAATCTCAGAATGCCCCAGAGAGTAAAGTG:T:chr8_134624854-chr8_134624958	0/1:NA:104:0,0:+:323:DEL:5373:NA:NA:chr8_134624854-chr8_134624958	split	q24.22	ZFAT				XM_011517204	3	1.34E+08	1.35E+08	106	0	0	no	15	intron2-intron2	CDS	12501	5'	1.35E+08	1.35E+08																										dbVar	chr8:134608227-134779707	0.01	gnomAD-SV_v3_DEL_chr8_d4501b6e	chr8:133866055-134670293	0.1984																																57.4	0.119428	-1.40202	-1.01818	-0.95443	1.113052					57623	610931	(Autoimmune thyroid disease, susceptibility to, 3), 608175 (3)				3	1.28E-08	2.93E-03	0.6411		Cleft lip;Cleft palate;Hypertelorism;Micrognathia;Microphthalmia;abnormal tooth morphology				full=3	0/1
1_35391781_35391915_DEL_1	1	35391781	35391915	-133	DEL	ILS_AP001,ILS_AP001_1,ILS_AP001_2	MantaDEL:191761:0:1:0:0:0	GCCTGTAATCTCAGCACTTTGGGAGGCTGAGGCAGGCGGATCACGAGGTCAGGAGTTTGAGACCATCCTGGCCAACATGGTGAAACCCCGTCTCTACTAAAATACAAAAAATTAGCTGGGTGTGGTGCTGCGCA	G	709	PASS	SUPP=3;SUPP_VEC=111;SVLEN=-133;SVTYPE=DEL;SVMETHOD=SURVIVOR1.0.7;CHR2=1;END=35391914;CIPOS=0,35;CIEND=0,1;STRANDS=+;Max_AF=0.00504;Max_Het=1296;Max_HomAlt=10;Max_PopMax_AF=0.037304;CCDG_Count=0;Best_CCDG_Mismatch_ID=124771_2;Best_CCDG_Mismatch_OFP=0.00746269;Best_CCDG_Mismatch_SVTYPE=BND;Best_CCDG_Mismatch_AF=0.003761;Best_CCDG_Mismatch_Het=0;Best_CCDG_Mismatch_HomAlt=0;SV_Cov=1;CCDG_SV_Cov=0;TOPMed_Count=1;Best_TOPMed_ID=DEL_1:35391782-35391914;Best_TOPMed_OFP=1;Best_TOPMed_AF=0.00504;Best_TOPMed_Het=1296;Best_TOPMed_HomAlt=10;Best_TOPMed_PopMax_AF=0.037304;Best_TOPMed_Mismatch_ID=INV_1:34936904-38057517;Best_TOPMed_Mismatch_OFP=4.29403e-05;Best_TOPMed_Mismatch_SVTYPE=INV;Best_TOPMed_Mismatch_AF=4e-06;Best_TOPMed_Mismatch_Het=1;Best_TOPMed_Mismatch_HomAlt=0;TOPMed_SV_Cov=1;ThousG_Count=0;Best_ThousG_ID=HGSV_2476;Best_ThousG_OFP=0.738806;Best_ThousG_AF=0.006712;Best_ThousG_Het=39;Best_ThousG_HomAlt=2;Best_ThousG_PopMax_AF=0.022463;ThousG_SV_Cov=0.738806;gnomAD_Count=1;Best_gnomAD_ID=gnomAD-SV_v3_DEL_chr1_2f5ed07a;Best_gnomAD_OFP=0.850746;Best_gnomAD_AF=0.003285;Best_gnomAD_Het=405;Best_gnomAD_HomAlt=4;Best_gnomAD_PopMax_AF=0.017792;Best_gnomAD_Mismatch_ID=gnomAD-SV_v3_INV_chr1_02e3c92a;Best_gnomAD_Mismatch_OFP=7.105e-06;Best_gnomAD_Mismatch_SVTYPE=INV;Best_gnomAD_Mismatch_AF=8e-06;Best_gnomAD_Mismatch_Het=1;Best_gnomAD_Mismatch_HomAlt=0;gnomAD_SV_Cov=1	GT:PSV:LN:DR:ST:QV:TY:ID:RAL:AAL:CO	0/1:NA:134:0,0:+:525:DEL:DEL00001573:NA:NA:chr1_35391781-chr1_35391915	0/1:NA:133:0,0:+:709:DEL:MantaDEL_191761_0_1_0_0_0:GCCTGTAATCTCAGCACTTTGGGAGGCTGAGGCAGGCGGATCACGAGGTCAGGAGTTTGAGACCATCCTGGCCAACATGGTGAAACCCCGTCTCTACTAAAATACAAAAAATTAGCTGGGTGTGGTGCTGCGCA:G:chr1_35391781-chr1_35391914	0/1:NA:98:0,0:+:314:DEL:329:NA:NA:chr1_35391816-chr1_35391914	split	p34.3	ZMYM4				XR_007064890	1	35268708	35397428	135	0	0	no	22	intron16-intron16	UTR	296	3'	35391780	35391915																													gnomAD-SV_v3_DEL_chr1_cf5f2df8	chr1:13203398-167621949	0.1676				IMH	1:7601938-50381771;1:10187873-186857210;1:16798094-234784887;1:31594523-94732184	0.4698																										13.84	1.343073	1.513139	1.782499	0.708669	2.831411					9202						0	1.0000e	1.0000e	0						full=1	0/1
9_112981593_113019974_DEL_1	9	1.13E+08	1.13E+08	-38381	DEL	ILS_AP001,ILS_AP001_1,ILS_AP001_2	MantaDEL:212049:1:3:0:0:0	T		809	PASS	SUPP=3;SUPP_VEC=111;SVLEN=-38381;SVTYPE=DEL;SVMETHOD=SURVIVOR1.0.7;CHR2=9;END=113019974;CIPOS=0,0;CIEND=-2,0;STRANDS=+;Max_AF=0;Max_Het=0;Max_HomAlt=0;Max_PopMax_AF=0;CCDG_Count=0;Best_CCDG_ID=435250;Best_CCDG_OFP=0.597161;Best_CCDG_AF=3.419e-05;Best_CCDG_Het=0;Best_CCDG_HomAlt=0;Best_CCDG_PopMax_AF=0;CCDG_Mismatches=537047;CCDG_Mismatches_Count=1;CCDG_Mismatch_SVTYPEs=DUP;Best_CCDG_Mismatch_ID=537047;Best_CCDG_Mismatch_OFP=0.903893;Best_CCDG_Mismatch_SVTYPE=DUP;Best_CCDG_Mismatch_AF=3.419e-05;Best_CCDG_Mismatch_Het=0;Best_CCDG_Mismatch_HomAlt=0;SV_Cov=1;CCDG_SV_Cov=1;TOPMed_Count=0;Best_TOPMed_ID=DEL_9:112952557-113025211;Best_TOPMed_OFP=0.52827;Best_TOPMed_AF=0.000114;Best_TOPMed_Het=32;Best_TOPMed_HomAlt=0;Best_TOPMed_PopMax_AF=0.000162979;TOPMed_Mismatches=DUP_9:112979208-113021665;TOPMed_Mismatches_Count=1;TOPMed_Mismatch_SVTYPEs=DUP;Best_TOPMed_Mismatch_ID=DUP_9:112979208-113021665;Best_TOPMed_Mismatch_OFP=0.903978;Best_TOPMed_Mismatch_SVTYPE=DUP;Best_TOPMed_Mismatch_AF=7e-06;Best_TOPMed_Mismatch_Het=2;Best_TOPMed_Mismatch_HomAlt=0;TOPMed_SV_Cov=1;ThousG_Count=0;Best_ThousG_ID=HGSV_164762;Best_ThousG_OFP=0.182065;Best_ThousG_AF=0.000156;Best_ThousG_Het=1;Best_ThousG_HomAlt=0;Best_ThousG_PopMax_AF=0.00056;Best_ThousG_Mismatch_ID=HGSV_164763;Best_ThousG_Mismatch_OFP=0.118415;Best_ThousG_Mismatch_SVTYPE=CPX;Best_ThousG_Mismatch_AF=0.039806;Best_ThousG_Mismatch_Het=231;Best_ThousG_Mismatch_HomAlt=12;ThousG_SV_Cov=0.299515;gnomAD_Count=0;Best_gnomAD_ID=gnomAD-SV_v3_DEL_chr9_21e41c3e;Best_gnomAD_OFP=0.597161;Best_gnomAD_AF=9.5e-05;Best_gnomAD_Het=12;Best_gnomAD_HomAlt=0;Best_gnomAD_PopMax_AF=0.001272;Best_gnomAD_Mismatch_ID=gnomAD-SV_v3_DUP_chr9_3bb074cb;Best_gnomAD_Mismatch_OFP=0.429264;Best_gnomAD_Mismatch_SVTYPE=DUP;Best_gnomAD_Mismatch_AF=8e-06;Best_gnomAD_Mismatch_Het=1;Best_gnomAD_Mismatch_HomAlt=0;gnomAD_SV_Cov=1	GT:PSV:LN:DR:ST:QV:TY:ID:RAL:AAL:CO	0/1:NA:38379:19,9:+:809:DEL:DEL00029120:NA:NA:chr9_112981593-chr9_113019972	0/1:NA:38381:0,0:+:557:DEL:MantaDEL_212049_1_3_0_0_0:NA:NA:chr9_112981593-chr9_113019974	0/1:NA:38381:0,0:+:281:DEL:5715:NA:NA:chr9_112981593-chr9_113019974	split	q32	ZNF883				NM_001101338	2	1.13E+08	1.13E+08	24064	1140	56	no	5	txStart-txEnd	5'UTR-3'UTR			1.13E+08	1.13E+08																										dbVar	chr9:112958718-113090720	0.01	dbVar	chr9:112952556-113025206	0.01				IMH	9:65437055-134286391;9:80897170-125694547;9:89671548-130954998	0.4714																																				169834									0						full=1	0/1
